# The early SARS-CoV-2 epidemic in Senegal was driven by the local emergence of B.1.416 and the introduction of B.1.1.420 from Europe

**DOI:** 10.1093/ve/veac025

**Published:** 2022-03-21

**Authors:** Lester J Perez, Gregory S Orf, Michael G Berg, Mary A Rodgers, Todd V Meyer, Aurash Mohaimani, Ana Olivo, Barbara Harris, Illya Mowerman, Abdou Padane, Agbogbenkou Tevi Dela-del Lawson, Aminata Mboup, Moustapha Mbow, Nafissatou Leye, Ndeye Coumba Touré-Kane, Ambroise D Ahouidi, Gavin A Cloherty, Souleymane Mboup

**Affiliations:** Infectious Disease Core Research, Abbott Diagnostics Division, Abbott Laboratories, 100 Abbott Park Rd, Lake Bluff, IL 60044, USA; Infectious Disease Core Research, Abbott Diagnostics Division, Abbott Laboratories, 100 Abbott Park Rd, Lake Bluff, IL 60044, USA; Infectious Disease Core Research, Abbott Diagnostics Division, Abbott Laboratories, 100 Abbott Park Rd, Lake Bluff, IL 60044, USA; Infectious Disease Core Research, Abbott Diagnostics Division, Abbott Laboratories, 100 Abbott Park Rd, Lake Bluff, IL 60044, USA; Infectious Disease Core Research, Abbott Diagnostics Division, Abbott Laboratories, 100 Abbott Park Rd, Lake Bluff, IL 60044, USA; Infectious Disease Core Research, Abbott Diagnostics Division, Abbott Laboratories, 100 Abbott Park Rd, Lake Bluff, IL 60044, USA; Infectious Disease Core Research, Abbott Diagnostics Division, Abbott Laboratories, 100 Abbott Park Rd, Lake Bluff, IL 60044, USA; Infectious Disease Core Research, Abbott Diagnostics Division, Abbott Laboratories, 100 Abbott Park Rd, Lake Bluff, IL 60044, USA; Infectious Disease Core Research, Abbott Diagnostics Division, Abbott Laboratories, 100 Abbott Park Rd, Lake Bluff, IL 60044, USA; Institut de Recherche en Santé de Surveillance Épidémiologique et de Formation, 4 Rue 2 D1 Pole Urbain de Diamniado, Dakar BP 7325, Senegal; Institut de Recherche en Santé de Surveillance Épidémiologique et de Formation, 4 Rue 2 D1 Pole Urbain de Diamniado, Dakar BP 7325, Senegal; Institut de Recherche en Santé de Surveillance Épidémiologique et de Formation, 4 Rue 2 D1 Pole Urbain de Diamniado, Dakar BP 7325, Senegal; Institut de Recherche en Santé de Surveillance Épidémiologique et de Formation, 4 Rue 2 D1 Pole Urbain de Diamniado, Dakar BP 7325, Senegal; Institut de Recherche en Santé de Surveillance Épidémiologique et de Formation, 4 Rue 2 D1 Pole Urbain de Diamniado, Dakar BP 7325, Senegal; Institut de Recherche en Santé de Surveillance Épidémiologique et de Formation, 4 Rue 2 D1 Pole Urbain de Diamniado, Dakar BP 7325, Senegal; Institut de Recherche en Santé de Surveillance Épidémiologique et de Formation, 4 Rue 2 D1 Pole Urbain de Diamniado, Dakar BP 7325, Senegal; Infectious Disease Core Research, Abbott Diagnostics Division, Abbott Laboratories, 100 Abbott Park Rd, Lake Bluff, IL 60044, USA; Institut de Recherche en Santé de Surveillance Épidémiologique et de Formation, 4 Rue 2 D1 Pole Urbain de Diamniado, Dakar BP 7325, Senegal

**Keywords:** SARS-CoV-2, viral surveillance, Bayesian inference of phylogeny, phylogeography, positive selection analysis, Senegal, variants of interest

## Abstract

Molecular surveillance of severe acute respiratory syndrome coronavirus 2 (SARS-CoV-2) is growing in west Africa, especially in the Republic of Senegal. Here, we present a molecular epidemiology study of the early waves of SARS-CoV-2 infections in this country based on Bayesian phylogeographic approaches. Whereas the first wave in mid-2020 was characterized by a significant diversification of lineages and predominance of B.1.416, the second wave in late 2020 was composed primarily of B.1.1.420. Our results indicate that B.1.416 originated in Senegal and was exported mainly to Europe. In contrast, B.1.1.420 was introduced from Italy, gained fitness in Senegal, and then spread worldwide. Since both B.1.416 and B.1.1.420 lineages carry several positive selected mutations in the spike and nucleocapsid genes, each of which may explain their local dominance, their mutation profiles should be carefully monitored. As the pandemic continues to evolve, molecular surveillance in all regions of Africa will play a key role in stemming its spread.

## 1. Introduction

The emergence of severe acute respiratory syndrome coronavirus 2 (SARS-CoV-2) in east Asia in late 2019 (Zhou et al. 2020) quickly became an international pandemic, spreading to five other continents by mid-2020. The first cases outside of China were linked to travel from China to Europe and the USA (Organization 2020a). By February 2020, cases began appearing in Africa, linked to travel from Europe (Organization 2020b). Although mitigation efforts and monitoring by the World Health Organization were put into place, local spread began to occur in many parts of the continent by April 2020 (Organization 2020c). Viral molecular surveillance efforts have continued since that time in order to inform public health authorities and ensure that diagnostic tests, vaccines, and therapeutics are not outpaced by emerging variants.

Although Africa contains about 16 per cent of the human population on Earth, it has only accounted for about 4 per cent of reported SARS-CoV-2 infections (Medicine 2021; Reuters 2021). This discrepancy may be due to a combination of inefficient diagnostic testing infrastructure in some areas and low population density in others (Wells et al. 2020). Additionally, there have been fewer sequenced SARS-CoV-2 genomes of African origin. As of September 2021, only ∼39,000 of the over 3.6 million genomes deposited in GISAID (Elbe and Buckland-Merrett 2017) (1.2 per cent) are of African origin, with the single largest share (∼17,600) deposited by investigators in South Africa. Recently, a prospective phylogenetics study was performed on the evolution of the pandemic generalized over the entire continent of Africa (Wilkinson et al. 2021). This study highlighted the heterogeneity of transmission patterns throughout Africa, although a unifying factor was the role of European travel on initial viral importation. Importantly, the unique influences that shaped the local epidemics of each African country should be evaluated in depth to track the emergence and relative fitness of variants that emerge.

Herein, we provide evidence and context for the local evolutionary processes and spread of SARS-CoV-2 in Senegal, which has one of the most robust viral surveillance programs in western Africa. Using Bayesian phylodynamic characterization, we identify the major events driving the first two waves of local infections during 2020. These events were characterized by the introduction of diverse lineages and mainly driven by local outbreaks of lineages B.1.416 and B.1.1.420. The differences in the rate of infection and spreading for these two lineages shaped the first two waves of the epidemic. Bayesian phylogeographic analysis identified the origin events of the epidemic within Senegal, the appearance of new viral lineages there, and their spatiotemporal dispersal to other countries. Importantly, during the second wave of the epidemic in mid-to-late 2020, the locally originated and entrenched lineages remained dominant, resulting in only a few cases of the Alpha variant that was prevalent in many other parts of the world at the time.

## 2. Results

### 2.1 First two waves in Senegal were dominated by lineages B.1.416 and B.1.1.420

**Figure 1.**
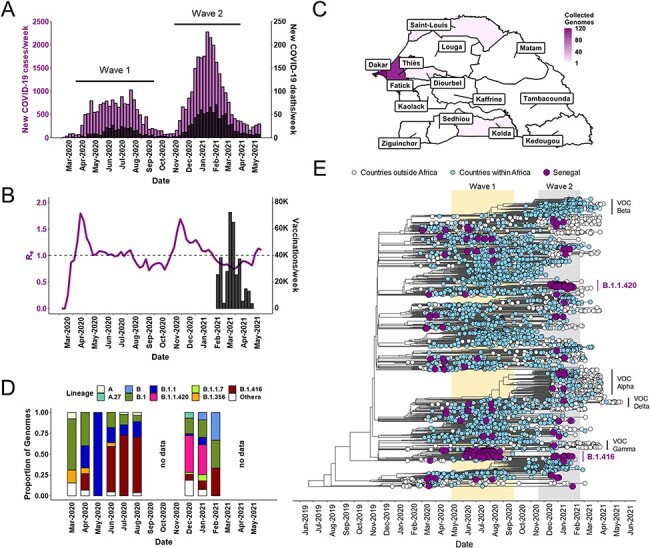
Dynamics and lineage diversity of the first two SARS-CoV-2 epidemic waves in Senegal. (A) The number of weekly cases of SARS-CoV-2 infection (purple bars) and associated deaths (black bars). The two epidemic waves are denoted. (B) changes in R_e_ estimations over time (purple line) and the progression of vaccination efforts (black bars). (C) Geographic representation of SARS-CoV-2 genome sampling in Senegal. (D) Frequency and distribution of the most common PANGOLIN lineages from March 2020 to February 2021. (E) The estimated time-resolved maximum clade credibility tree from 5,230 genomes containing a global representation (white dots), an African overrepresentation (blue dots), and all data available from Senegal (purple dots). The clades formed by variants of concern or interest are denoted. Data for Panels A and B are publicly available from Our World in Data (www.github.com/owid/covid-19-data/blob/master/public/data/). The column of panels containing A, B, and D are on the same time axis.

The SARS-CoV-2 epidemic in Senegal began in March 2020 with introductions from travel linked to Europe (Anonymous 2020; Hoije 2020). The temporal distribution of the Senegalese SARS-CoV-2 epidemic thereafter is summarized with weekly data obtained from Our World in Data (Fig. 1A and B). An initial peak in cases (purple) and SARS-CoV-2-associated deaths (black) was observed over mid-2020 (March 2020–August 2020: Wave 1), followed by a period of relative quiet when lockdown measures were put in place beginning in August (Fig. 1A and Supplementary Information, Fig. S1). Caseload began increasing once again in late 2020 as these restrictions were lifted and then subsided by April 2021 (November 2020–April 2021: Wave 2). The tally of case counts allows the calculation of the effective reproduction number (*R_e_*), which can be used to describe the expansion of the epidemic (Fig. 1B). The calculated rate of infections at >1 reflects these waves, with a spike in the *R_e_* value to ∼2 immediately preceding an increase in cases. For Wave 1, the *R_e_* value returns to ∼1 within a month’s time, hovering below 1 when cases were low and then sharply increasing to ∼2 again in December 2020. As *R_e_* leveled off again in February 2021, Senegal had begun vaccinating healthcare workers, peaking at roughly 75,000 vaccinations per week.

As a response to Wave 1, two institutions launched active genomic surveillance programs in Senegal: Institut de Recherche en Santé de Surveillance Epidemiologique et de Formation (IRESSEF) and the Institut Pasteur, both located in the Dakar Region. This allowed us to collect genetic information about the SARS-CoV-2 strains circulating in Senegal. SARS-CoV-2 sequences (*N* = 157 high quality) from IRESSEF were obtained from nasopharyngeal specimens collected from patients residing in five of the more populous regions in the country (Dakar, Thies, Diourbel, Saint Louis, and Kolda; Fig. 1C), with sampling evenly distributed between the first two waves (Supplementary Information, Figure S1 and Figure S2A-C). Additional sequences (*N* = 74) deposited in GISAID by the Institut Pasteur were also included in our analysis for a combined *N* = 231 Senegalese sequences representing collection dates before 5 February 2021. Although the relatively low number of sequences may suggest sampling bias, we observed a statistically supported correlation between the sequenced genomes and cases over time. Additionally, we observed a statistically supported association between sequenced genomes and cumulative cases by location (Supplementary Information, Figure S2D–E).

The frequency and distribution of PANGOLIN (*Phylogenetic Assignment of Named Global Outbreak Lineages*) classifications changed dramatically from March 2020 to February 2021 (Fig. 1D). Whereas Wave 1 consists of approximately 5 lineages, genetic diversity is much greater in Wave 2 with >10 lineages detected. A shift in distribution is evident, with the percentage of the B.1.1 (blue) and B.1.416 (red) lineages decreasing in prevalence over time, while B.1.1.420 (pink) is ascendant in Wave 2. During the time of decreased viral expansion between Waves 1 and 2, there was a corresponding lack of genomic data.

An estimated time-resolved maximum clade credibility (MCC) analysis (as implemented in BEAST), using a constrained model with a strict molecular clock, was performed to determine the correlation between the sequences and their time of local emergence or external introduction (Fig. 1E). A total of *N* = 5,230 sequences were evaluated, including *N* = 231 Senegalese (purple), all unique African (blue; *N* = 3,317), and representative global (white; *N* = 1,682) strains. Consistent with the PANGOLIN classifications (Fig. 1D), Senegalese strains were restricted to only a few lineages in Wave 1 and then expanded to several in Wave 2. After initial introduction or emergence, we observed the growth and predominance of lineage B.1.416 between June and August 2020. A similar trend was observed in Wave 2 from January to February 2021; however, the dominant lineage was B.1.1.420. It is notable that very few B.1.1.7 (Alpha) and no B.1.351 (Beta) variants of concern gripping Europe and southern Africa during this time were detected in Senegal during either wave. Gamma (P.1) and Delta (B.1.617.1) variants of concern were also not detected in Senegal during this time.

### 2.2 B.1.416 and B.1.1.420 exhibit relatively high genetic diversity and rates of infection

**Figure 2.**
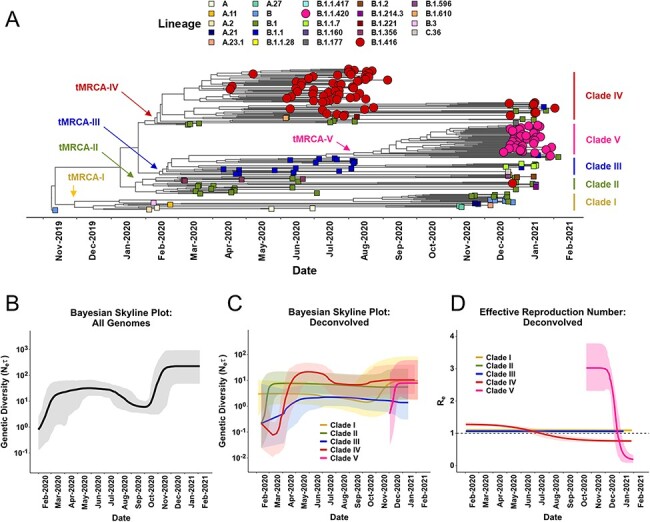
Evolutionary history, demographic dynamics, and effective rate of infection of SARS-CoV-2 strains circulating in Senegal. (A) The estimated time-resolved maximum clade credibility tree from 231 SARS-CoV-2 sequences collected in Senegal (plus Wuhan reference) with tip shapes colored by PANGOLIN lineage. The topological reconstruction revealed five major clades; the tMRCA for each clade is denoted (confidence intervals are noted in Supplementary Information, Table S1). (B) Demographic history of SARS-CoV-2 strains collected in Senegal inferred via a BSP with coalescent tree prior and an exponential, uncorrelated clock model. The shading represents the 95 per cent HPD of the product of generation time and effective population size (N_e_τ). The line tracks the inferred median of N_e_τ. (C) The deconvolved BSP (with 95 per cent HPD shading) estimated for each of the five major clades. (D) The reconstructed Re, also deconvolved for each of the five major clades. The median R_e_ profile for each clade (line), computed from the posterior birth–death rates and SIR trajectories, are framed by the 95 per cent HPD interval (shading).

Our next analysis focused exclusively on the Senegal sequences to understand the phylodynamics of SARS-CoV-2 in this country. We identified five main clades with distinct times of introduction or emergence (Fig. 2A). The emergence time of the most recent common ancestor (tMRCA) for Clade 1 was estimated to be sometime in November 2019. This clade is monophyletic and composed mostly of A, B, and B.1 lineages. Ancestors of Clades II, III, and IV emerged in late January to early February 2020, each containing a unique mixture of lineages. Notably, Clade IV (tMRCA = 14 March 2020, 95 per cent highest posterior density [HPD; 6 February 2020 to 19 April 2020]) was comprised primarily of the Wave 1-dominant lineage B.1.416 and related B.1 strains. Clade V emerged as a daughter of Clade III and consists almost entirely of the B.1.1.420 strains dominating Wave 2, with the tMRCA dating to October 2020 (tMRCA = 19 October 2020, 95 per cent HPD [13 September 2020 to 25 November 2020]), just before the end of Wave 1.

To obtain insight into the demographic composition of SARS-CoV-2 cases in Senegal, Bayesian skyline plots (BSPs) describing the genetic diversity present over time were calculated. Considering all Senegalese sequences (Fig. 2B), we observed a sharp increase in genetic diversity by more than an order of magnitude early in 2020. This led to the main phase of Wave 1, during which the diversity was relatively constant. As Wave 1 was subsiding in September 2020, genetic diversity temporarily decreased before a second increase that ushered in Wave 2. The rise in diversity leading to Wave 2 was elevated compared to that preceding Wave 1. This implies that newly emergent or imported strain(s) may have contributed to the overall upward trend in diversity preceding Wave 2.

We recomputed the BSPs using the Senegalese tree ungrouped by clade, allowing us to deconvolve the relative contributions of each clade to the overall profile (Fig. 2C). After their initial establishment, Clades II (green) and III (blue) exhibited very little change in their diversity, leading to their effective extinction by January 2021. Clade I experienced low genetic diversity for the majority of 2020, which increased near the end of the year as a mixture of A and B.1 daughter lineages emerged, likely due to external introductions. Clade IV displayed a different trend in genetic diversity, showing an initial decrease followed by an abrupt increase beginning in March 2020. Mostly consisting of lineage B.1.416, Clade IV maintained a high diversity throughout Wave 1 until July 2020, concomitant with the overall downward trend in cases. When Wave 2 began in October 2020, Clade IV’s diversity increased again. Lineage B.1.416 appeared to suppress the proliferation of its parent B.1, which persisted through all phases of Waves 1 and 2 but did not constitute a large share of cases. When Clade V was established in November 2020, it displayed a dramatic increase in genetic diversity, maintained through February 2021, although there is some uncertainty given that our sampling interval ends early that month. The evolutionary rates for Clades I–IV range from 4.6 to 8.2 ×10^−4^ nt substitutions site^−1^ year^−1^ (13–24 nt substitutions genome^−1^ year^−1^; Supplementary Table S1); accordingly, these clades in aggregate characterize the diversity of Wave 1 (Fig. 2B). Clade V, on the other hand, had an evolutionary rate of 1.5 × 10^−3^ nt substitutions site^−1^ year^−1^ (43 nt substitutions genome^−1^ year^−1^). Since Clade V drove Wave 2, this is reflected in the higher genetic diversity seen in the aggregate BSP from October 2020 onward (Fig. 2B).

Since genetic diversity is not necessarily an indication of a successful infection, we used the birth-death susceptible infectious recovery (BDSIR) model implemented in BEAST2 to calculate the *R_e_* for each clade independently (Fig. 2D). Clades I–III persist near *R_e_* = 1.0, suggesting that they neither propagate efficiently nor represent a major contribution to the epidemic in Senegal. By contrast, Clade IV, composed primarily of B.1.416, had an *R_e_* >1 early on, which dropped below 1 following the conclusion of Wave 1. Clade V, composed primarily of lineage B.1.1.420, quickly rose to the highest *R_e_* value in the dataset (R_e_ > 3), beginning in August 2020 and quickly falling below 1 after Wave 2 declined. The combined BSP and *R_e_* profiles for Clades IV and V (Fig. 2C and D) indicate that each clade may have been impacted by a mixture of fitness advantage or increased number of available susceptible individuals due to easing of government restrictions (Supplementary Information, Fig. S1). We attribute the early dominance of lineage B.1.416 to increased genetic fitness, while the later dominance of lineage B.1.1.420 could be due to both increased genetic fitness and relaxed restrictions.

Results obtained from the embedded genetic information interrogated by the BDSIR model (Supplementary Figure S3) revealed a steady upward trend in the prevalence and incidence for Clades I–III. This indicates that the sampling for those clades is not adequately representative of all susceptible individuals, suggesting these clades were the result of external introductions, which had already genetically diversified before arriving in Senegal. By contrast, Gaussian-shaped profiles were observed for Clades IV and V, which indicate adequately representative sampling from the susceptible individuals, suggesting either a local emergence or a local diversification for these two lineages (Supplementary Figure S3). Both clades had a high rate of infection (*R_e_* > 1) at some point during either Wave 1 or Wave 2.

### 2.3 Despite different origins, both B.1.416 and B.1.1.420 lineages gained fitness in Senegal and spread across the globe

**Figure 3.**
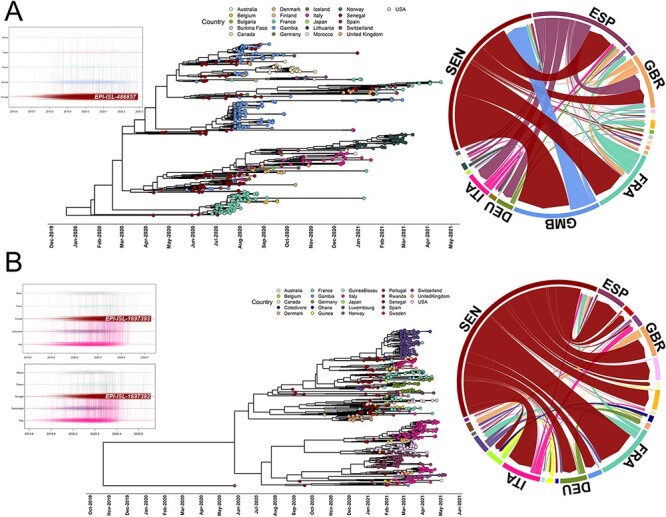
Discrete phylogeographical evaluations of SARS-CoV-2 PANGO lineages (A) B.1.416 and (B) B.1.1.420. Both A and B follow the same format from left to right. Left panel: Markov jump trajectory plot representing the ancestral transition history for the most ancestral genome in Senegal for each lineage. The trajectories are summarized from a posterior tree distribution with Markov jump history annotation using a sampling location and travel history model. Lines in a horizontal trajectory represents the time during which a particular location state is maintained in the spatiotemporal ancestry of the virus, and vertical lines represent Markov jumps between two locations in the trajectory. The most ancestral genomes for each lineage in Senegal location are denoted. Central panel: maximum clade credibility tree from Bayesian inference, colors of the tip shapes represent the sampling locations in the legend. Right panel: circular migration flow plot representing the origin and destination of international spreading events. The width of the links indicates the frequency of viral movements as estimated using *post hoc* summarized posterior expectations of the Markov jump events. The direction of the migration flow starts from the outer ring to the destination location denoted by an arrowhead.

We performed a discrete phylogeographic analysis using BEAST to reconstruct the entry and/or exit of different lineages to and from Senegal by attaching location, collection date, and available travel information for each sequence (Fig. 3 and companion Supplementary web applications). Starting with B.1.416 (Clade IV), the major lineage characterizing Wave 1, we constructed an MCC tree of all B.1.416 sequences reported across the globe to determine its origin and describe its change in spatiotemporal distribution (Fig. 3A). The root of the MCC tree identified the most ancestral B.1.416 sequence as originating from Senegal, which was confirmed by Markov jump analysis incorporating travel history (also see a time-dependent analysis in Supplementary Information, Fig. S4). From Senegal, the initial spreading events were to Spain, France, and the UK, with Spain and France constituting major secondary spreaders to other parts of Europe. There was also a substantial transmission of B.1.416 between Senegal and neighboring Gambia; however, these events did not contribute to the spread elsewhere. Following the same approach, we focused on B.1.1.420, which dominated Wave 2. Markov jump analysis incorporating travel history indicated the most ancestral B.1.1.420 sequence came from Italy (Fig. 3B and Supplementary Information, Fig. S4). After introduction to Senegal, this lineage gained fitness (i.e. increased genetic diversity) and transmissibility (i.e. increased *R_e_*) (Fig. 2) and then spread to several other countries, particularly in Europe (e.g. France, UK, and Germany). Thus, Senegal was the incubator for lineage B.1.1.420, but it did not originate there.

### 2.4 Wave 1 spread across Senegal, while Wave 2 was confined to Dakar and Thiés

**Figure 4.**
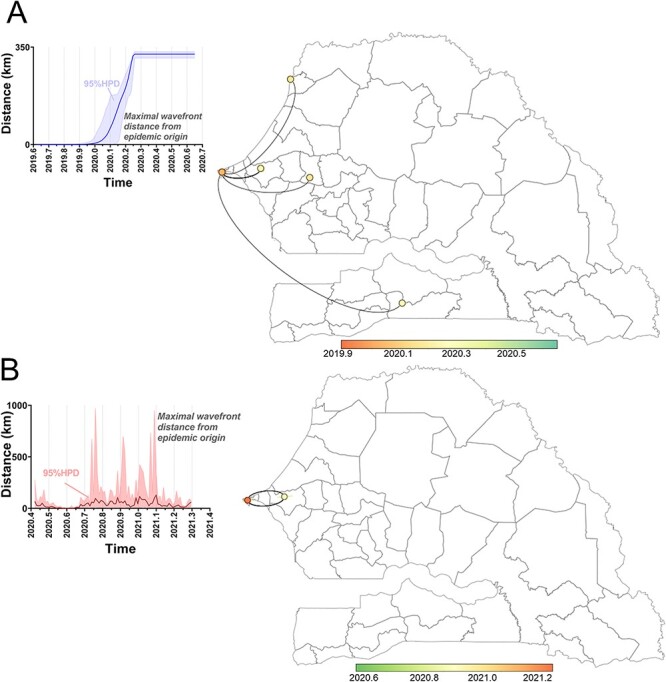
Spatiotemporal dynamics of the dispersal history of SARS-CoV-2 within Senegal during (A) Wave 1 and (B) Wave 2. Continuous phylogeographic reconstructions are based on 10^3^ post-burn-in posterior trees. Both panels represent the maximal wavefront distance from the epidemic origin in dark blue (A) and dark red (B) lines. The 95 per cent HPD intervals are represented in light blue (A) and light red (B) bracketing the median. The map borders represent Senegal at the Département level and display the maximal spread distance of the virus at various points in time.

Increased spatial resolution of SARS-CoV-2 spread was achieved by constraining the analysis to one geographic region and performing a continuous phylogeographic reconstruction on the Senegalese strains alone. We observed that Wave 1 originated in Dakar in early 2020 and spread to the rest of the country (Fig. 4A). By the end of March 2020, the virus had reached 350 km from Dakar, spreading to multiple regions, including Saint-Louis in the northwest, Thiés and Diourbel in the east, and Kolda in the south. By the end of Wave 1 in mid-2020, the virus had spread throughout the western half of the country. Due to the low population density and lack of positive tests, we could not resolve transmission to the eastern half of the country. Wave 2, by contrast, was confined to the regions of Dakar and Thiés (Fig. 4B). Here, we observed a sharing of strains within these neighboring regions, whereas Wave 1 was characterized by a unidirectional dispersal from Dakar.

### 2.5 The increased fitness of lineages B.1.416 and B.1.1.420 were driven by positively selected mutations

**Figure 5.**
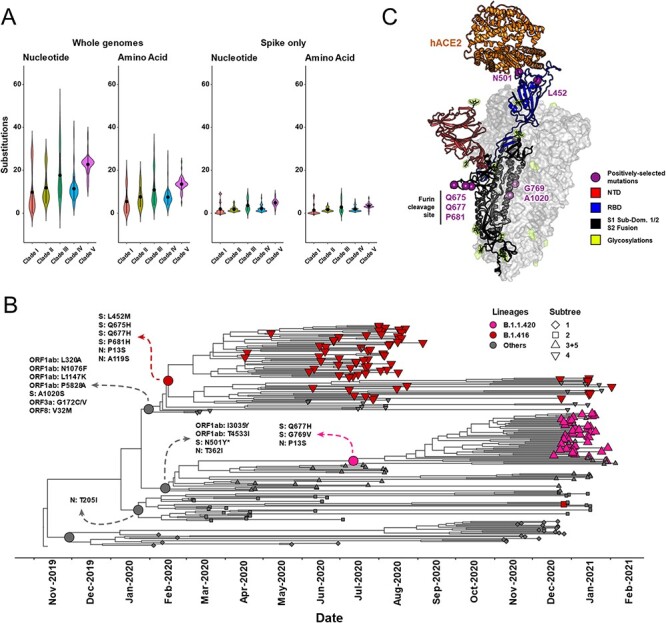
Positively selected mutation events in Senegalese SARS-CoV-2 strains. (A) Violin plots depicting the mutation counts in each of the five Senegalese clades at the nucleotide or amino acid level in either the whole genome or spike protein alone. Plots depict the full probability density of counting a certain number of mutations in the strains; black dots indicate the mutation count median; black vertical lines depict 1 SD. (B) The maximum clade credibility tree reproduced from Fig. 2A with emphasis on lineages B.1.416 and B.1.1.420, as well as positive selection events called out at the ancestral node of each clade or lineage of interest. Gray-dotted nodes are the clade ancestors and call out positively selected sites identified by PAML’s branch-site model, while red- or pink-dotted nodes are the B.1.416 or B.1.1.420 lineage ancestors and call out positively selected sites identified by PAML’s site model. (C) The three-dimensional structure of SARS-CoV-2 spike glycoprotein complexed with the hACE2 receptor, with positively selected amino acid sites and regions of interest identified. Mutation N501Y (shown with an asterisk in panel B) is a positively selected mutation; however, it does not occur in the lineages of interest, B.1.416 or B.1.1.420.

To assess the molecular basis for the increased *R_e_*, fitness, and dispersal velocity found in Clades IV and V (lineages B.1.416 and B.1.1.420, respectively), we compared the mutations in their protein-coding regions to those found in the other Senegalese sequences (Fig. 5). Violin plots illustrate the average number of accumulated mutations relative to the Wuhan reference (Fig. 5A). We observed an average of 23 nt substitutions genome^−1^ in Clade V, over the entire sampling window, compared to approximately 12–15 in the others. Correspondingly, an average of 18 amino acid substitutions genome^−1^ were found in Clade V compared to approximately 8–12 in the others. However, there was not a statistically significant difference between clades, so the question shifted to determining the efficacy of the mutations versus the quantity. Indeed, in the spike region, the number of nucleotide and amino acid changes were similar across all clades. To explain the phenotypic characteristics of B.1.416 and B.1.1.420, we performed a positive selection analysis using the *branch-site* model and *model-site* model implemented in the program PAML.

By merging the *branch-site* model results with the temporal analysis, we determined that the emergence of each clade resulted from distinct positive selection events. Foundational mutations of each clade were retained by daughter taxa (i.e. ω >1.0; Fig. 5B and Supplementary Information, Table S2). The *branch-site* model identified the following mutations in structural genes: spike A1020S (S2 fusion domain) found in Clade IV and nucleocapsid T205I found in Clade II. In the case of lineage B.1.416 specifically, additional mutations in spike and nucleocapsid were identified as positively selected using the separate *model-site* model; these included spike L452M, Q675H, Q677H, and P681H, as well as nucleocapsid P13S and A119S. For B.1.1.420, spike Q677H and G769V, as well as nucleocapsid P13S, were identified (Fig. 5 and Supplementary Information, Table S3). To visualize these mutations and their potential roles, we highlighted them on a 3D cryo-EM structure of the spike trimer complexed with the human ACE2 (hACE2) receptor (Fig. 5C). The signature mutations for B.1.416 fall in the receptor-binding domain (RBD; L452M) and the furin cleavage site (Q675H, Q677H, and P681H). The signature mutations in B.1.1.420 fall within the furin cleavage domain (Q677H) and the S2 fusion domain (G769V). Additionally, the N501Y mutation in spike is identified as a positively selected event in Clade III; this mutation is found in the few B.1.1.7 (Alpha variant) sequences identified during Wave 2, although, as mentioned previously, this lineage did not come to dominate in Senegal.

Putting these mutations in the context of the global pandemic, most of them are relatively rare in the GISAID database as of mid-September 2021. The nucleocapsid mutations P13S and A119S are found in 6,129 and 8,611 sequences, respectively. The spike RBD mutation L452M was found in 1,255 genomes, including some SARS-CoV-2 viruses found in mink. Other known lineages with mutations at L452 include variant of interest (VOI) Lambda (L452Q) and variant of concern (VOC) Delta (L452R). This mutation is situated on the face of the RBD opposite to that which binds hACE2; this mutation may confer a structural change to the RBD or could interfere with antibody binding to RBDs in the ‘open’ conformation. The S2 fusion domain mutations G769V and A1020S were found in 15,696 and 4,567 genomes, respectively. These mutations are in a similar region to the highly proliferated D614G mutation that has been hypothesized to confer stability to spike in the cleaved state.

The mutations in the furin cleavage site, on the other hand, are more commonly seen globally. Spike Q675H, Q677H, and P681H have been observed 24,749, 42,328, and 1,195,518 times, respectively. P681H is one of the signature mutations in the variants of concern Alpha and Omicron and the VOI Mu; however, in Senegal, it is found in the B.1.416 lineage. Q675H, also found in B.1.416, seems to have appeared sporadically in South America, Africa, and South Asia. Q677H, found in both B.1.416 and B.1.1.420, seems to have appeared multiple times, independently. Although it is beyond the temporal scope of this study, Q677H is now a signature mutation in more recent lineages, including the VOI Eta (prevalent during mid-2021 in central Africa) and the AY.34 lineage of the VOC Delta, the predominant Delta lineage in west Africa (including Senegal) during mid-to-late-2021.

## 3. Discussion

The global SARS-CoV-2 pandemic continues to strike in regional surges that cumulatively have led to over 245 million infections and 5 million deaths worldwide as of October 2021 (https://coronavirus.jhu.edu/map.html, last accessed 29 October 2021). Throughout the pandemic, global molecular surveillance has served as an early alert system to identify viral strains that may pose a new risk for diagnostic accuracy, transmission mitigation, vaccine efficacy, and therapeutic interventions. With transmission networks spanning borders and continents, the strength of the global surveillance system relies on strong specimen sampling and sequencing capacity in every country affected by the pandemic. Unequal distribution of sequencing capacity ultimately results in gaps in the SARS-CoV-2 evolutionary history and allows VOCs to circulate silently until they spread to countries with strong sequencing infrastructure (Dudas et al. 2021). In this context, our analysis of the first two waves (Fig. 1) of the local epidemic in Senegal provides some key insights into the relative fitness and evolutionary history of SARS-CoV-2.

The SARS-CoV-2 epidemic in Senegal began with multiple reported (Anonymous 2020; Hoije 2020) and calculated (Fig. 3) introductions between December 2019 and February 2020, which led to Wave 1. In response, Senegal launched molecular testing efforts, travel restrictions, and public gathering restrictions in March, with mask mandates following in April 2020 (Supplementary Information, Fig. S1). Midway through Wave 1, the B.1.416 lineage emerged in and spread through Senegal (Figs 2–4) and was subsequently exported to French-speaking countries (Fig. 3) where it has diversified further into the B.1.416.1 sub-lineage. Around this same time, restrictions were lifted and B.1.1.420 arrived from Italy, likely from a traveler visiting from one of these countries (Fig. 3 and Supplementary Information, Fig. S4). Over the next several months, B.1.1.420 diversity and infectivity increased at a pace on par with the Delta variant (Liu and Rocklov 2021) (i.e. *R_e_* ≥ 3; Clade V, Fig. 2) as Senegal settled into a new phase of relaxed public restrictions, air travel, and schools resuming toward the end of 2020. The combination of increased genetic diversity and increased close contact between people likely drove Wave 2 in December 2020–March 2021 (Fig. 4) while also allowing the exportation of B.1.1.420 back to Europe (Fig. 3). The combined phylodynamic and phylogeographic analyses (Figs 2 and 4) indicate that, despite the higher *R_e_* from lineage B.1.1.420 during Senegal’s Wave 2 in comparison with B.1.416 during Wave 1, the dispersal of Wave 2 was smaller in geographic space, potentially due to the mitigation measures in place in the country (Supplementary Information, Fig. S1).

Notably, the Alpha variant (B.1.1.7) was introduced to Senegal at the beginning of Wave 2 in December 2020 (Padane et al. 2021) (Fig. 2); yet, it did not become widespread, contrary to the pattern seen in other countries such as the USA, where it rapidly became the predominant strain after introduction from the UK (Paul et al. 2021). In fact, Alpha only constituted 6 of 113 sequenced genomes in Wave 2. This, along with the BDSIR model predictions suggests that B.1.1.420 (Clade V, Fig. 2) may have had a fitness advantage over B.1.1.7 (Clade III, Fig. 2). Some of the positively selected mutations found in B.1.1.420 are at positions also mutated in VOCs Alpha, Delta, and Omicron (e.g. L18, P26, L452, S477, Q677, N501, and P681; Fig. 5), suggesting that the mutations themselves could be an indicator of spread more so than the individual lineage designations themselves. Since known vaccine escape mutations are not present in B.1.416 and B.1.1.420, and prior work in cell culture has demonstrated that the L452M mutation does not lead to antibody escape (Greaney et al. 2021), vaccines would be expected to be effective against the B.1.416 and B.1.1.420 lineages.

The evolutionary patterns of B.1.416 and B.1.1.420 highlight some key concepts in the spread and surge patterns of SARS-CoV-2. The rise of each of these lineages in Wave 1 and Wave 2, respectively (Fig. 1), was preceded by a period of intralineage diversification (Fig. 2) that could have been identified with real-time surveillance as opposed to the retrospective surveillance presented here. Ideally, molecular surveillance and phylogenetics can be used as a *predictive* tool instead of just a retrospective tool. As the speed with which sophisticated phylogeographic analysis (e.g. Figs 3 and 4) can be undertaken increases, increased sampling, sequencing capacity, and travel monitoring should keep pace to enable rapid responses. Indeed, while our Senegalese sample size was limited, the sampling strategy whereby genomes sequenced were linked to case and population count helped mitigate the effects of temporal and geographic bias (Supplementary Information, Fig. S2). The combination of phylogeographic, BSP, and BDSIR techniques together gives a compelling picture of epidemic evolution. BSPs, the BDSIR model, and continuous phylogeographic analysis provide evidence for a lineage originating near the time of first detection in a country, giving an intracountry perspective. Discrete phylogeographic analysis with embedded travel history, on the other hand, helps to further limit sampling bias and provides evidence for a lineage’s origin from an intercountry perspective. Agreement of the two lends credibility to statements about origin and proliferation of a lineage.

In the current retrospective analysis, we observed the rise and decline of lineages B.1.416 and B.1.1.420, which illustrates the progression of an outbreak predominated by a single lineage, followed by exportation to other countries. Our analysis demonstrates that this process is not unique to strains designated as VOCs and is an expected outcome of natural selection in a naïve population. The introduction of vaccines added a new evolutionary bottleneck that was absent in the sampling period of the current study. In the time since this dataset was collected, the Delta variant drove a third wave in Senegal from July to September 2021 despite vaccination efforts, demonstrating that more prevention work is needed to avoid a fourth wave in the future. Additionally, seasonal cycles in endemic SARS-CoV-2 infection (Wilkinson et al. 2021) will further affect local epidemiological patterns, necessitating long-term surveillance like what is undertaken for influenza.

## 4. Methods

### 4.1 Data collection and genome sequencing

IRESSEF’s surveillance efforts in Senegal between June 2020 and February 2021 are the subject of this study. Nasopharyngeal swabs in viral transport media were collected from sites throughout Senegal and sent to Abbott Laboratories for analysis. The study was reviewed and approved by the Ethical Committee of the Ministry of Health of Senegal (000129/MSAS/CNERS). All participants gave informed written consent prior to specimen collection.

Total nucleic acid was extracted on the m2000sp (Abbott Molecular, Des Plaines, IL). Any RNA was converted into complementary DNA using SuperScript IV first-strand reagents and Sequenase v2.0 second-strand reagents. Complementary DNA was transformed into Illumina-compatible libraries using a Nextera-XT library kit and custom IDT-Nextera indexes. SARS-CoV-2 sequences were enriched using a custom xGen panel (IDTDNA, Coralville, IA) (Orf et al. 2021) and sequenced via NGS on a HiSeq instrument (Illumina) (Ahouidi et al. 2021). A total of *N* = 162 genomes were assembled using an in-house SARS-CoV-2 software pipeline (Ahouidi et al. 2021). These genomes have been deposited to GISAID. *N* = 74 previously released SARS-CoV-2 genomes from Senegal, deposited by both the Institut Pasteur and IRESSEF, were also retrieved from the GISAID database, resulting in a total of *N* = 236 Senegalese genomes to consider as of early February 2021.

### 4.2 Dataset collection, filtering, and subsampling

To avoid biases in the inference and comparative studies, different datasets were created. Initially, a list of headers for a globally representative, Africa-excluded, genome dataset was downloaded from the daily NextStrain update (along with 20 extra Delta variant genomes, totaling *N* = 3,283 sequences) on 23 April 2021 set as the final day for sequence collection in our study. In addition, *all* SARS-CoV-2 genome headers with their respective metadata with an origin in any African country *except Senegal* were downloaded from the same GISAID repository on 23 April 2021 (*N* = 9,629). The complete genome sequences and associated metadata corresponding to the header list were retrieved from a local copy of the complete GISAID repository.

All aforementioned sequences were combined with those from Senegal, resulting in a starting dataset of *N* = 13,148 sequences. Quality control, alignment, and clipping measures were then implemented. First, the sequences were run through a local sandboxed Docker deployment of NextClade (Orf GS 2021); those with ‘bad’ QC scores (using default scoring metrics in NextClade) or total mutations >40 were discarded. Sequence headers were renamed with relevant metadata information, including accession, country, PANGOLIN lineage, NextClade classification, and date of collection. The selected sequences were aligned using MAFFT with settings optimized for closely aligned viral genomes (https://mafft.cbrc.jp/alignment/software/closelyrelatedviralgenomes.html, last accessed 2 June 2021). The 5ʹ and 3ʹ UTRs were trimmed due to the general concentration of sequencing issues in these regions (Hong et al. 2021; Morel et al. 2021). Likewise, following the criteria described by Morel and coworkers (Morel et al. 2021), to avoid the effect induced by sequences with low quality, a bash script was used to further eliminate any sequences with more than 20 contiguous ambiguous bases, more than 300 total ambiguous bases, or less than 29,300 total nucleotides. Furthermore, a bash script was used in conjunction with a perl script to eliminate all 100 per cent identical sequences, retaining only the most ancestral sequence of an identical set.

Quality control and de-duplication measures removed *N* = 7,913 sequences from the analysis. Thus, an initial dataset containing *N* = 5,235 sequences, of which *N* = 3,317 were of African, non-Senegal origin, *N* = 236 were of Senegalese origin (*N* = 162 new Abbott-sequenced genomes plus *N* = 74 previously sequenced genomes), and *N* = 1,682 were of non-African origin, was obtained.

### 4.3 Phylogenetic and temporal-noise evaluation

The initial *N* = 5,235 dataset was used as the input for maximum likelihood (ML) phylogenetic inference using IQ-TREE version 1.6.12 (Nguyen et al. 2015). The algorithm ModelFinder (Kalyaanamoorthy et al. 2017) was initially used to find the best-fit nucleotide substitution model according to Bayesian information criterion, followed by ML tree reconstruction. One thousand replicates of both the Shimodaira–Hasegawa Approximate Likelihood Ratio Test (Anisimova and Gascuel 2006) and Ultrafast Bootstrapping (Hoang et al. 2018) were performed to provide branch supports for the ML tree. The yielded ML tree (with taxon labels containing parsable collection dates) was imported into TempEst (Rambaut et al. 2016) to assess its molecular clock. A root-to-tip regression was performed using the heuristic residual mean squared function to both calculate an underlying temporal signal and detect any outlier sequences. Five sequences were detected as potential outliers and removed; these were all from Senegal. The remaining *N* = 5,230 (of which now *N* = 231 were Senegalese) sequences were reprocessed through IQ-TREE using the same settings as before to produce a refined ML tree. From the refined *N* = 5,230 dataset (*Dataset G*), two additional subsequent datasets were further extracted: (i) *N* = 3,548 (*Dataset A*) containing all sequences from Africa (including Senegal) that passed the quality control filters and (ii) *N* = 232 (*Dataset S*) containing all (*N* = 231) remaining Senegalese sequences plus the Wuhan-Hu-1 reference sequence. The ML trees obtained were visualized using the *ggtree* (Yu et al. 2017) package in R (Supplementary Information, Fig. S5) and the root-to-tip regression was visualized using the *ggplot2* (Wickham 2016) package in R (Supplementary Information, Fig. S6).

### 4.4 Temporal and dynamic of the main lineages of SARS-CoV-2 in Senegal

The datasets were used for a time-scaled phylogenetic reconstruction using the Bayesian Markov Chain Monte Carlo (MCMC) framework. First, an initial time-scaled phylogeny was obtained by using the TreeTime (Sagulenko, Puller, and Neher 2018) algorithm, which employs the output ML tree from IQ-TREE as the starting point. The resulting topology (Supplementary Information, Fig. S7) was then used as the starting tree for a recently described alternative likelihood function, together with a constrained model implemented BEAST v.1.10.5pre_thorney_v0.1.1 (https://github.com/beast-dev/beast-mcmc/releases/tag/v1.10.5pre_thorney_v0.1.1, last accessed 23 June 2021) and a nonparametric Skygrid coalescent prior with a window of sampling of 86 points considering the time for the most recent sample (5 February 2021). The BEAGLE v.3.1.0 library was used to enhance computational speed (Ayres et al. 2012).

Additionally, *Dataset S* was also used to infer a time-scale phylogeny reconstruction focused only on Senegal. A GTR substitution model with a gamma distribution was assumed under three different clock models assessed, including (i) a Strict Clock (Ferreira and Suchard 2008), (ii) an Uncorrelated Relaxed Clock (Alexei J Drummond et al. 2006), and (iii) a Random Local Clock (Alexei J Drummond and Suchard 2010). In all cases, an exponential growth coalescent was assumed as a prior (A. J. Drummond et al. 2005). MCMC sampling was performed for 10^8^ iterations, sampled every 10^5^ steps for both log and tree files. After completion, the convergence of the chains for each Clock was inspected using Tracer v.1.7. The estimation of the Bayes factor (BF) available in Tracer was used to determine the best-fitting model for the dataset.

For both *Dataset G* and *Dataset S*, MCC trees were generated using TreeAnnotator after removing 25 per cent burn-in stages determined by Tracer. The reference sequence Wuhan-Hu-1 (Genbank accession NC_045512 or GISAID accession EPI_ISL_406798) was used as outgroup to infer an ancestor. The resulting MCC trees were visualized using *ggtree* in R (Yu et al. 2017).

BSPs were calculated for *Dataset S* to infer the population dynamics of SARS-CoV-2 in terms of changing levels of relative genetic diversity (*N_e_τ*) through time in Senegal. The BSPs were visualized using *ggplot2* in R (Wickham 2016).

### 4.5 Phylogeographic analysis

To distinguish between external introductions and local emergence of lineages, a *discrete trait phylogeographic inference* was performed using an incorporated travel history model described by Lemay and coworkers (Lemey et al. 2020) following recommendations suggested by Hong and coworkers (Hong et al. 2021). Briefly, the identified ancestor taxon for each main clade was analyzed; from all the lineages analyzed, two lineages were identified where the root of the tree yielded a sequence from Senegal suggesting a local emergence: B.1.416 and B.1.1.420. Thus, all sequences available at GISAID database belonging to those lineages were downloaded on 23 April 2021 to avoid any bias in the inference of the origin, and the Python script developed by Hong and coworkers (Hong et al. 2021) was used to incorporate the travel history included in the associated metadata.

Thus, for the lineages B.1.416 and B.1.1.420, two new datasets were created containing 577 and 711 sequences, respectively. For those lineages, a *post hoc* analysis to determine the MarkovJump estimates for transition histories were generated using the new BEAST tree sampling tools *TaxaMarkovJumpHistoryAnalyzer* and *TreeMarkovJumpHistoryAnalyzer* available from the BEAST codebase (https://github.com/beast-dev/beast-mcmc, last accessed 23 June 2021). The R packages *MarkovJumpR* (Hong et al. 2021), *circlize* (Gu et al. 2014), and home-built interactive Shiny apps incorporating *Mapdeck* (https://symbolixau.github.io/mapdeck/articles/mapdeck.html, last accessed 12 October 2021) were used for visualization purposes. The Shiny apps are deployed at https://gregory-orf-phd.shinyapps.io/B_1_416_Phylogeographic_Analysis/ and https://gregory-orf-phd.shinyapps.io/B_1_1_420_Phylogeographic_Analysis/ (both publicly available as of 23 November 2021).

To determine the phylogenetic diffusion of lineages across Senegal, we applied a flexible relaxed random walk model with a Cauchy distribution to determine the among-branch heterogeneity in diffusion velocity (Lemey et al. 2010) (also known as a *continuous phylogeographic inference*). We first divided *Dataset S* by epidemic wave. Then, for each sequence, the plaintext collection location was converted into a latitude and longitude using the Python script *loc_resolve.py*; due to the uncertainty, we added a jitter window with a size of 0.01, as suggested by Dellicour and coworkers (S. Dellicour et al. 2021). MCMC chains were run for 10^8^ generations and sampled every 10^6^ steps, with convergence assessed using Tracer v1.7. The R package *Seraphim* (Simon Dellicour et al. 2016) was used to extract and statically map spatiotemporal information embedded in the posterior trees. A tutorial for *Seraphim* is available at https://github.com/sdellicour/seraphim (last accessed 23 August 2021).

### 4.6 Estimation of the rate of infections from the different detected clade in Senegal using BDSIR model

To estimate the effective reproduction rate (*R_e_*) of the SARS-CoV-2 clades over time in Senegal, we used a BDSIR model (Kühnert et al. 2014) included in the software package BEAST 2 v.2.6.2 (Bouckaert et al. 2019). This model allows for the estimation of *R_e_*, rate of an infection being transmitted (*λ*) or becoming noninfectious (δ), and the probability that an infectious individual was sampled during the study (*s*). For the prior, a population size of susceptible individuals was fixed to a gamma distribution with α = 10^2^ and β = 4 ×10^4^, δ was set considering the duration of the infectious period of a SARS-CoV-2 infection (Byrne et al. 2020) (exponential, mean = 40), and standard deviation of 1.3. In addition, a Strict Clock model and substitution model HKY + G were selected, and MCMC chains were run for 8 × 10^7^ generations sampled every 8 × 10^4^ steps. Epidemiological information from the BDSIR model was extracted by applying the *plot_BDSIR.R* script described by the tutorial available at https://www.beast2.org/tutorials/ (last accessed 25 August 2021).

### 4.7 Positive selection analysis per site and among lineages

To perform a positive selection analysis, two additional datasets were constructed from the Senegal-only alignments (Dataset S). In the first (*whole genome dataset*), the UTRs, intergenic regions, and stop codons from the genomes were removed, leaving the coding regions for *all* ORFs in frame and contiguous with each other. In the second (*structural gene dataset*), the structural genes S, M, E, and N alone were extracted. A bash script (*pos_sel_cull_noambiguoustolerated.sh*) was used to remove all sequences containing any IUPAC ambiguous nucleotides (i.e. WSKMYRVHDBN). The resulting alignments were used to evaluate the action of positive selection pressure. The total number of nucleotide and amino acid mutations (relative to the Wuhan reference) for the whole genome and S protein alone were also calculated and visualized as violin plots using the *ggplot2* package in R.

The hypothesis of positive selection on the *structural gene dataset* was tested by the site models implemented in the CODEML program of the PAML v4.9 software package (Yang 2007), following the criteria reported by Rios and coworkers (Rios et al. 2017). Briefly, values of the nonsynonymous/synonymous dN/dS rate ratio (*ω* parameter) were assessed from the alignments, and false positives were avoided by contrasting the models used to detect sites under positive pressure with models used to detect neutral selection (Supplementary Information, Table S4) (Anisimova and Yang 2007). The Bayes Empirical Bayes calculation of posterior probabilities for site classes was used to estimate the probabilities of sites under positive selection. The structural genes were also tested for a discrete distribution using M3 and M0 models. Model M0 allows for a single value across the whole phylogenetic tree at all sites, and M3 assumes multiple categories of selection, not necessarily positive selection (Pikuła et al. 2021).

For the *whole-genome dataset*, *branch-site* models to evaluate the emergence of positive selected lineages were tested as described by Rios and coworkers (Rios et al. 2017). Briefly, *branch-site* tests, using prespecified branches, are hypothesized to have occurred (foreground branches) and were made with the null Model A1. The significance of the likelihood ratio tests was calculated assuming that twice the difference in the log maximum likelihood between the two models was distributed as a distribution with the degrees of freedom given by the difference in the number of parameters in the two types of models (Rios et al. 2017). Like for the *model site* model, the *branch-site* model was tested to avoid false-positive detection (Supplementary Information, Table S2); thus, the branch site Model A, which allows for *ω* >1 along foreground branches, was compared with the null hypothesis.

The identified positively selected nucleotide mutations in the S protein were related to the resulting amino acid mutations. To visualize those amino acid mutations, the three-dimensional cryo-EM structure of SARS-CoV-2 spike glycoprotein trimer in complex with the hACE2 receptor was downloaded from the Protein Data Bank (accession 7A94). The unresolved furin cleavage loop (which was of interest) was built *de novo* using PyMol 2.5.2 (Schrödinger, Inc., New York, NY) and loop-only refinement was performed using Modeller (Webb and Sali 2016), version 10.1. The final structure was visualized again in PyMol, with ray-tracing performed before image export.

### 4.8 Epidemiological data

Daily Senegalese cases of SARS-CoV-2, deaths, estimates of *R_e_*, and number of vaccinated individuals were retrieved from publicly released data provided by Our World In Data (OWID), accessible through the repository (https://ourworldindata.org/coronavirus, last accessed 12 October 2021)) (Hannah Ritchie et al. 2020). OWID builds its database using data supplied by public entities such as government health authorities.

## Supplementary Material

veac025_Supp
