# Supplementary material for: The early SARS-CoV-2 epidemic in Senegal was driven by the local emergence of B.1.416 and the introduction of B.1.1.420 from Europe
Source: Virus Evol. 2022 Mar 21;8(1):veac025. doi: 10.1093/ve/veac025 (PMC8971539; doi:10.1093/ve/veac025)
Supplement: veac025_Supp [file veac025_supp.zip › Supplementary_information.pdf]

Supplementary Information for:

The early SARS-CoV-2 epidemic in Senegal was driven by the local emergence of B.1.416 and the introduction of B.1.1.420 from Europe

Lester J. Perez[1†], Gregory S. Orf[1†], Michael G. Berg[1], Mary A. Rodgers[1], Todd V. Meyer[1], Aurash Mohaimani[1], Ana Olivo[1], Barbara Harris[1], Illya Mowerman[1], Abdou Padane[2], Agbogbenkou Tevi Dela-del Lawson[2], Aminata Mboup[2], Moustapha Mbow[2], Nafissatou Leye[2], Ndeye Coumba Touré Kane[2], Ambroise D. Ahouidi[2], Gavin A. Cloherty[1], Souleymane Mboup[2]

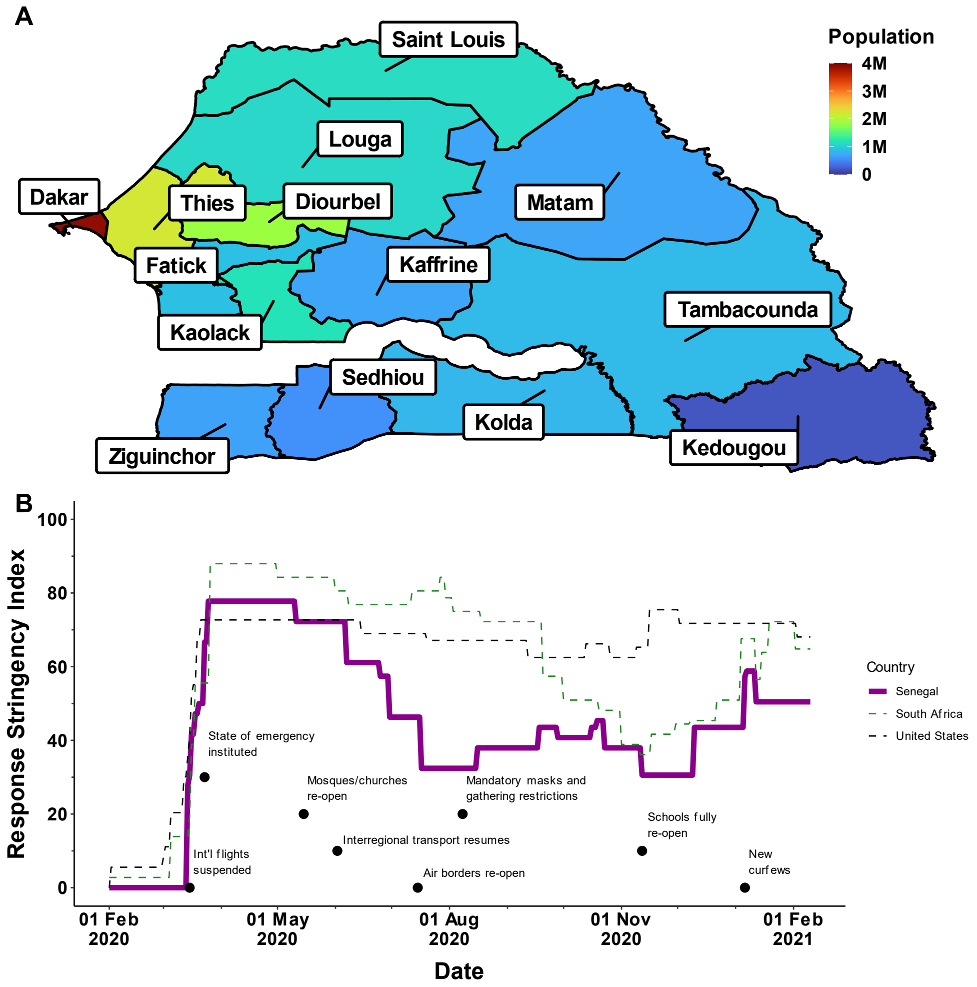

Figure S1. National data for Senegal. A) Population counts; the major regional divisions in Senegal are shown, with population scale at upper right. B) Response stringency index; a measure of the strictness of government actions for mitigating COVID-19 (available from Our World in Data, https://ourworldindata.org/covid-stringency-index). Various mitigation efforts announced by the Senegalese government are shown on the timeline. The stringency index for the United States and South Africa are shown with dashed lines for comparison.

A

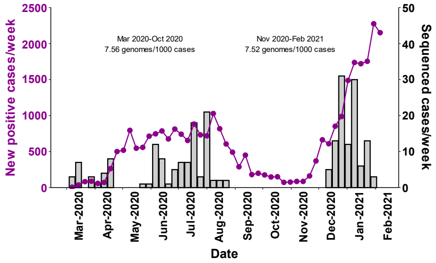

B

Region	Cumulative cases through 06 Feb 2021	Population at 2013 census	Cumulative cases per 1,000 residents
Dakar	18,250	3,137,196	5.82
Thiès	3,358	1,788,864	1.88
Diourbel	1,482	1,497,455	0.99
Kaolack	1,143	960,875	1.19
Saint-Louis	999	908,942	1.10
Louga	652	874,193	0.75
Fatick	574	835,352	0.69
Kolda	284	714,392	0.40
Tambacounda	246	681,310	0.36
Kaffrine	98	566,992	0.17
Matam	328	562,539	0.58
Ziguinchor	606	549,151	1.10
Sédhiou	156	452,944	0.34
Kédougou	313	152,357	2.05

C

Genomes collected cataloged by sampling location

Mar 2020-Oct 2020	Count
**Dakar**	**42**
Dakar	35
Guediawaye	1
KeurMassar	1
Mbao	2
Sangalkam	1
Yeumbeul	2
**Diourbel**	**2**
Touba	2
**Kolda**	**1**
Velingara	1
**Saint-Louis**	**2**
Saint-Louis	2
**Thiès**	**71**
Guereo	4
Joal-Fadiouth	1
Khombole	5
Mbour	17
Popenguine	6
Thiès	26
Tivaoune	12
**Grand Total**	**118**

Nov 2020-Feb 2021	Count
**Dakar**	**76**
Dakar	76
**Thiès**	**37**
Joal-Fadiouth	1
Khombole	1
Mbour	10
Mèckhè	1
Popenguine	1
Thiadiaye	1
Thiès	16
Tivaoune	6
**Grand Total**	**113**

D

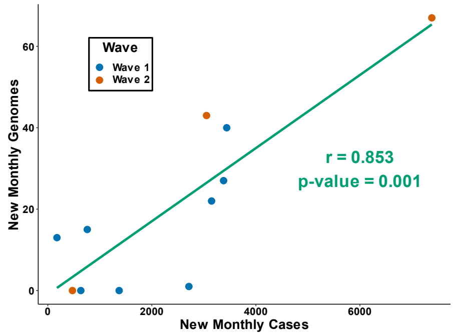

E

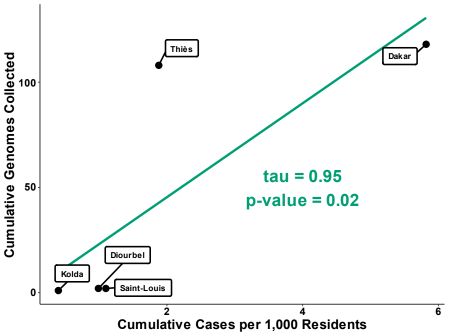

Figure S2. Spatiotemporal statistics of SARS-CoV-2 infections and bias analysis of genome collection. A) Genomes collected plotted against case count during the study timeframe, binned by week. B) Breakdown of case count per 1,000 residents of each of the 14 Regions of Senegal. C) Geographic distribution of collected genomes, separated by wave. D) New monthly genomes collected plotted against new monthly cases; February 2021 is omitted from the linear regression and Pearson bivariate correlation test because the study timeframe ended only 5 days into the month. E) Cumulative genomes collected plotted against cumulative cases per 1,000 residents in the five Senegalese regions sampled; Kendall's tau test and linear regression are also shown.

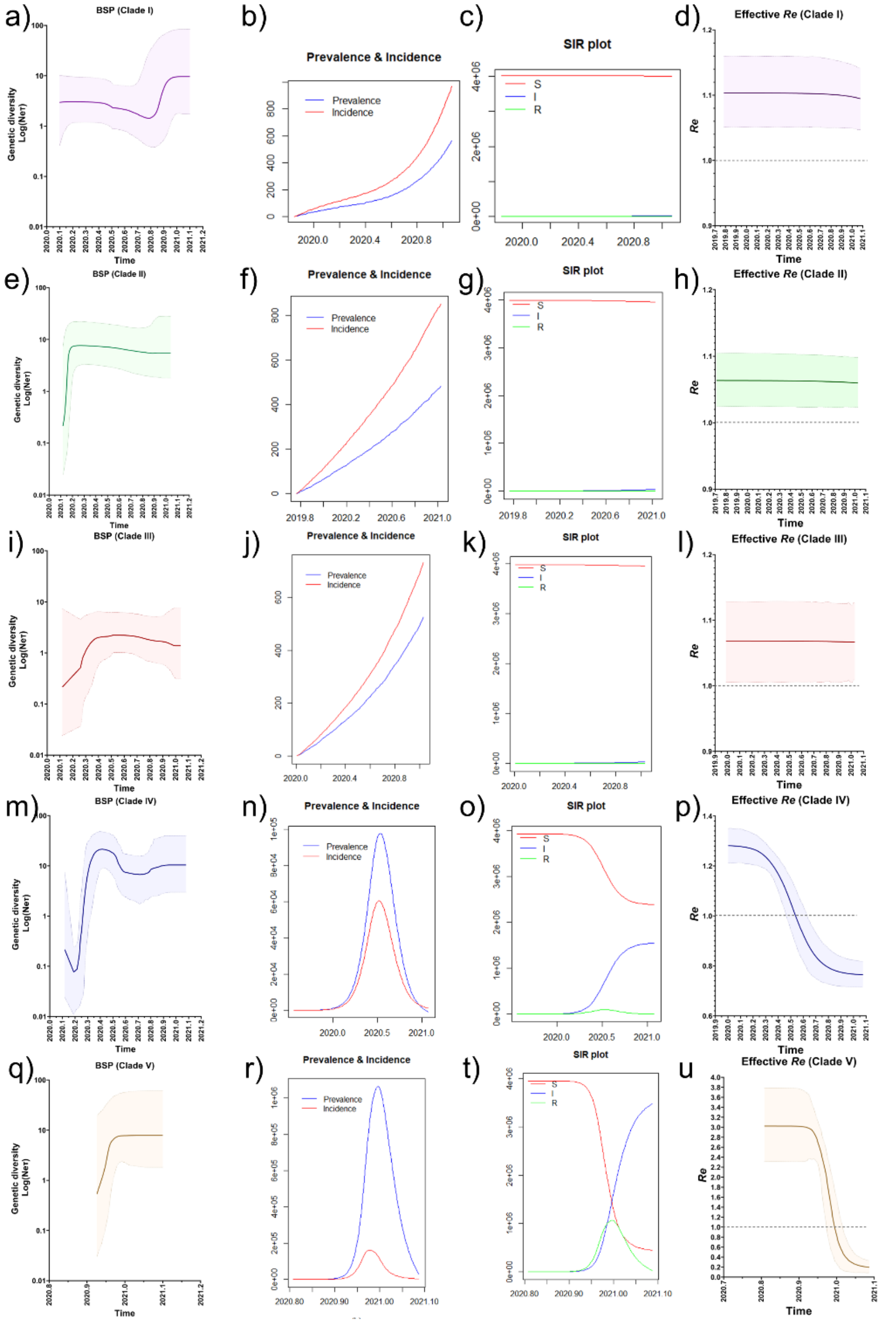

Figure S3. Demographic dynamics and SIR trajectories and incidence of SARS-CoV-2 clusters in Senegal. a,e,i,m,q) Bayesian skyline plot (with 95% HPD shading) estimated for each of the five major clades. The incidence (b,f,j,n,r) and SIR trajectories (c,g,k,o,t) reveal at what stage in the epidemic each cluster was sampled. d,h,l,p,u) Reconstructed effective reproduction ratio (R_e) obtained from the BDSIR model (with 95% HPD shading).

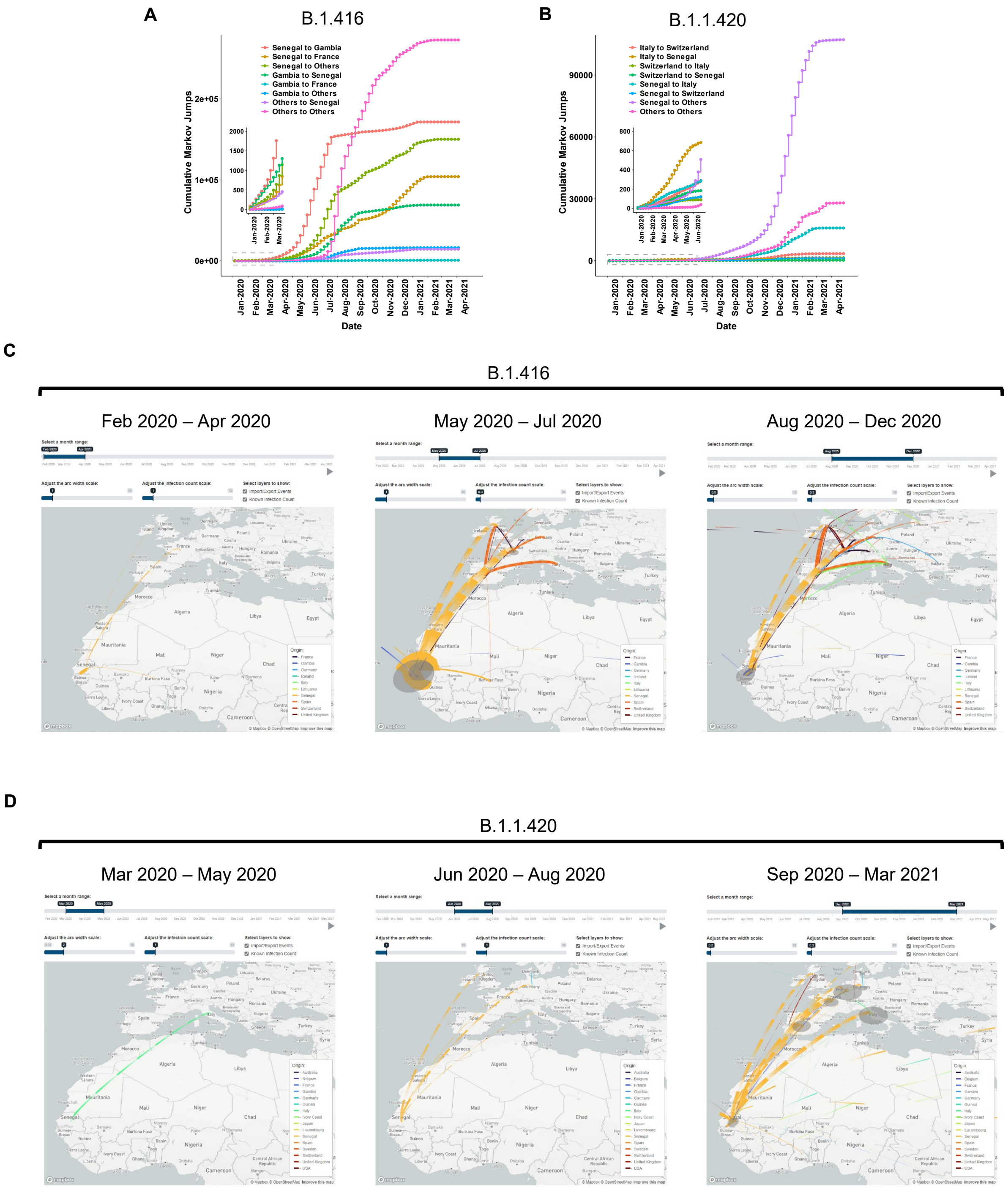

Figure S4. Supplementary analysis of the discrete character phylogeographic analysis. A) Cumulative Markov jumps for lineage B.1.416, B) cumulative Markov jumps for lineage B.1.1.420, C) maps of calculated international spreading events for lineage B.1.416, and D) maps of calculated international spreading events for lineage B.1.1.420. Panels C and D are extracted from our home-built Shiny app (access information available in the Main Text).

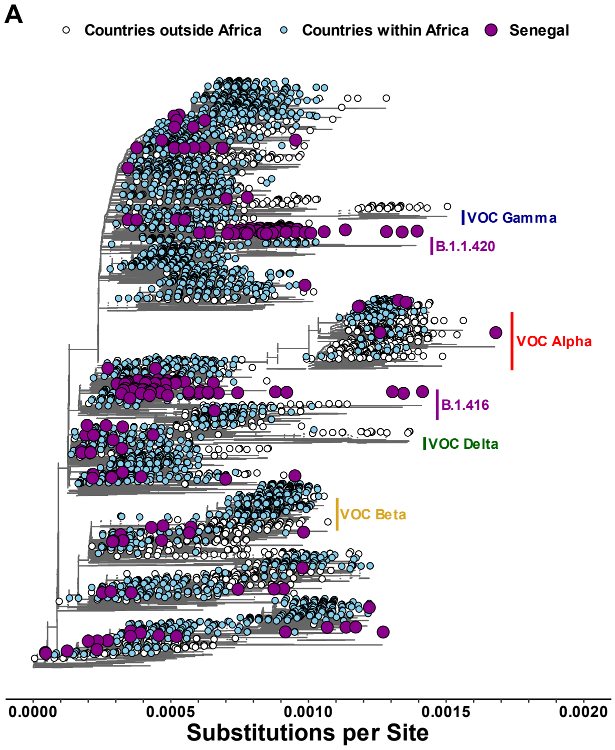

Figure S5. Maximum likelihood trees for (A) Dataset G and (B) Dataset S. Branches are drawn to scale and measured in average substitutions per site. Tips are color-coded based on sampling region. VOCs and lineages of interest are denoted.

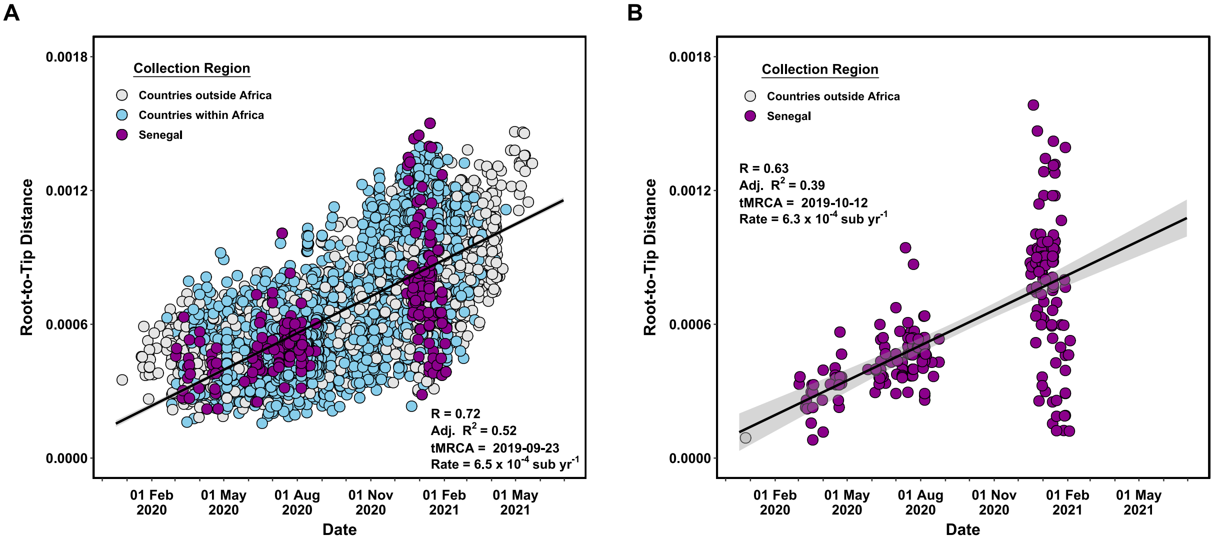

Figure S6. Root-to-tip regression of genetic distances and sampling dates for (A) Dataset G and (B) Dataset S. Regressions were estimated using TempEst v.1.5.3 (http://tree.bio.ed.ac.uk/software/tempest/). Points correspond to single tips of the ML phylogenetic trees, Timetrees, and MCC tree shown in **Figure S3**, **Figure S5,** and **Figure 1**, respectively.

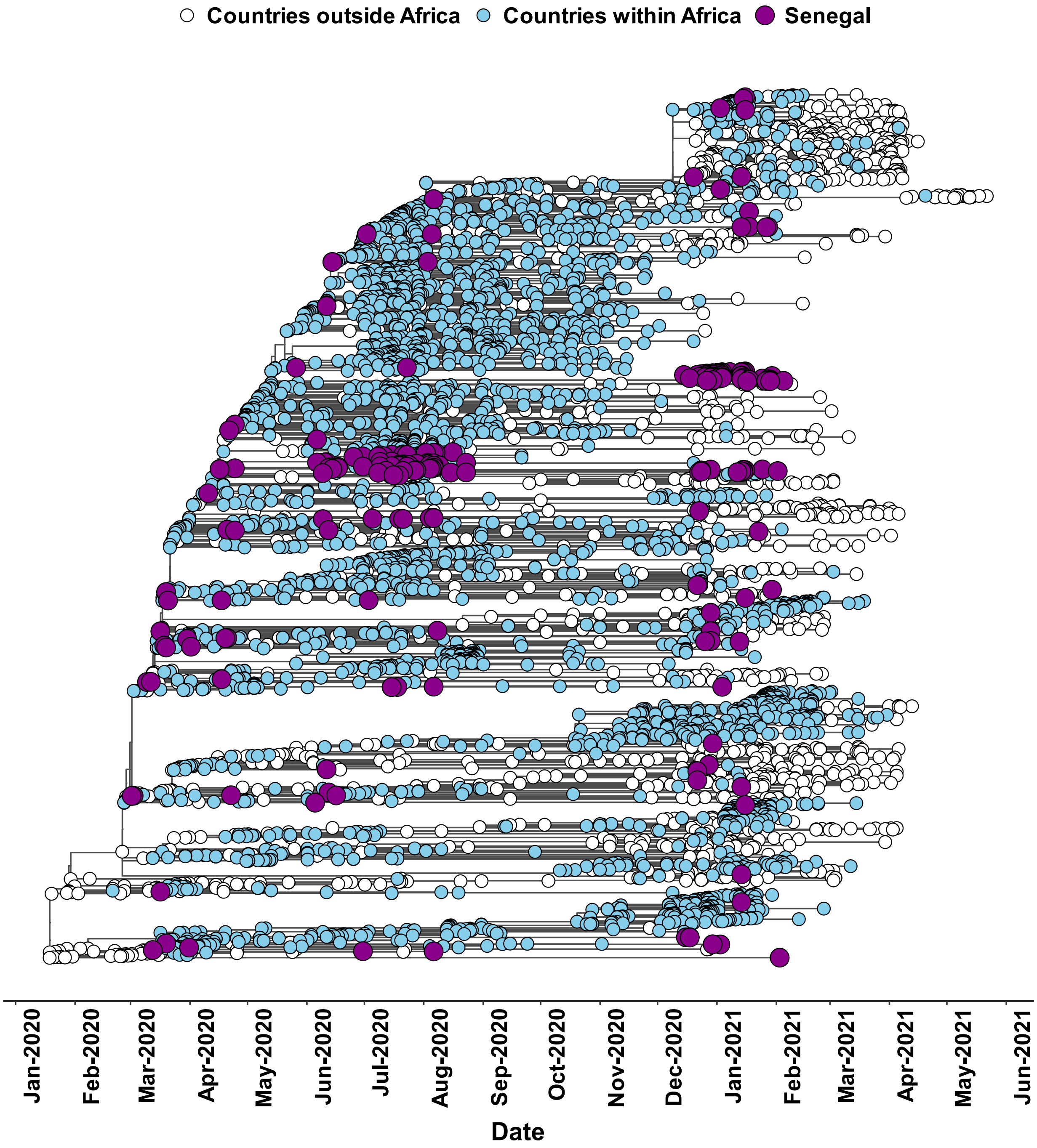

Figure S7. Timetree for Dataset G. The topology was obtained with TimeTree v.0.8.1 (https://github.com/neherlab/treetime) using the ML tree from **Figure S3A** as the starting tree. The tips are color-coded based on sampling region.

Table S1. Estimated substitution rates and tMRCA for the Senegalese SARS-CoV-2 main clades

**Clade**	**Evolutionary rates [HPD95%] (substitution/site/year)**	**tMRCA [HPD95%] (year)**
I	4.64×10^{-4} [2.51×10^{-4} - 6.87×10^{-4}]	2019.6 [2018.7-2020.1]
II	6.05×10^{-4} [4.80×10^{-4} - 7.22×10^{-4}]	2020.0 [2019.9-2020.1]
III	8.11×10^{-4} [3.48×10^{-4} - 1.39×10^{-3}]	2020.1 [2019.8-2020.3]
IV	5.78×10^{-4} [4.97×10^{-4} - 6.75×10^{-4}]	2020.2 [2020.1-2020.3]
V	1.46×10^{-3} [7.07×10^{-4} - 2.19×10^{-3}]	2020.8 [2020.7-2020.9]

Table S2. PAML branch-site model A analysis to identify branches under episodic positive selection in SARS-CoV-2 strains circulating in Senegal.

**Foreground branches**	**Parameters$^{n.m}$**	**-lnL$^{n.m}$**	**Parameters$^{a.m}$**	**-lnL$^{a.m}$**	**-2ΔlnL**	**Selected sites**
Subtree I	P_0= 0. 53742	26844.57478	P_0= 0.60602	26857.232634	**25.32****	-
	P_1= 0. 27880		P_1= 0.21727			
	P_{2a}= 0. 12100		P_{2a}= 0.13008			
	P_{2b}= 0. 06277		P_{2b}= 0.04663			
	ω_o= 0.0000		ω_o= 0.00807			
	ω_1= 1.00000		ω_1=1.00000			
	ω_2=1.00000		ω_2=**1.91572**			
Subtree II	P_0= 0.57246	26882.04608	P_0= 0.61988	26937.69825	**111.30****	**5215 (N:T205I)**
	P_1= 0.25389		P_1= 0.21670			
	P_{2a}= 0.12029		P_{2a}= 0.12109			
	P_{2b}= 0.05335		P_{2b}= 0.04233			
	ω_o= 0.0000		ω_o= 0.00000			
	ω_1= 1.00000		ω_1=1.00000			
	ω_2=1.00000		ω_2=**3.88496**			
Subtree III	P_0= 0.65023	26918.93029	P_0= 0.67374	26978.55674	**119.2529****	**1426 (ORF1ab:I3035Y, 2510**
	P_1= 0.20978		P_1= 0.18189			**(ORF1ab:T4533I), 5367**
	P_{2a}= 0.10585		P_{2a}= 0.9998			**(N:T362I)**
	P_{2b}= 0.03415		P_{2b}= 0.03069			
	ω_o= 0.0000		ω_o= 0.0001			
	ω_1= 1.00000		ω_1=1.0000			
	ω_2=1.00000		ω_2=**6.06861**			
Subtree IV		26927.34769		26953.34406	**51.99274****	**162 (ORF1ab:L320A, 543**
	P_0= 0.60307		P_0= 0.61403			**(ORF1ab:N1076F), 607**
	P_1= 0.22795		P_1= 0.19635			**(ORF1ab:L1147K), 1266**
	P_{2a}= 0.12263		P_{2a}= 0.14368			**(ORF1ab:V2715L),**
	P_{2b}= 0.04635		P_{2b}= 0.04594			**3463 (ORF1ab:P5828A),**
	ω_o= 0.00000		ω_o= 0.0000			**4427 (S:A1020S), 4700**
	ω_1= 1.00000		ω_1= 1.00000			**(ORF3a:G172C/V), 5005**
	ω_2=1.00000		ω_2= **7.36574**			**(ORF8:V32M)**

lnL: log-likelihood scores;
n.m: null model; a.m: alternative model
*$p<0.05$, χ^2= 3.84; **$p<0.01$, χ^2= 5.99

Table S3. Positively selected sites and parameters estimated by the CODEML program implemented in the PAML package.

Gene	Model	log-likelihood score						Positive site
S	M0	-6020.564638	ω0= 0.45413					-
	M1	-6000.276312	p0= 0.74936	p1=0.25064	ω= 1.000			-
	M2	-5975.434732	ω= 27.50058	p0=0.71326	p1=0.27859	p2=0.00816		**452L**, 501N*, 675Q*, 677Q**, 681P**, 769G****
	M3	-5971.681746	ω= 117.36444	p0=0.96949	p1=0.02889	p2=0.00162		18L*, 26P*, 452L**, 477S*, 501N**, 675Q*,677*Q, 681P*, 769G*, 1219G*
	M7	-6000.763162	p= 0.00576	q= 0.01350				-
	M8	-5976.179685	ω= 20.37060	p0= 0.97370	p= 0.80534	q= 5.09210	(p1= 0.01284)	18L*, 26P*, **452L****, 477S*, **501N****, 614G*, **675Q*,677*Q, 681P*, 769G*,** 1219G*
E	M0	-347.114591	ω0= 0.45413					-
	M1	-347.114592	p0= 0.99999	p1=0.00001	ω= 1.000			-
	M2	-347.114591	ω= 2.00672	p0=1.00000	p1=0.0	p2=0.0		-
	M3	-347.114591	ω= 0.59790	p0=0.29520	p1=0.23228	p2=0.47252		-
	M7	-347.115098	p= 99.00000	q= 66.51438				-
	M8	-347.115099	ω= 1.0000	p0= 0.97370	p= 99.00000	q= 66.51511	(p1= 0.00001)	-
M	M0	-1044.873955	ω0= 0.12122					-
	M1	-1044.873980	p0= 0.99999	p1= 0.00001	ω= 1.000			-
	M2	-1044.873955	ω= 2.00672	p0=0.93380	p1=0.06399	p2=0.00222		-
	M3	-1044.873955	ω= 0.12122	p0= 0.18455	p1= 0.44839	p2= 0.36706		-
	M7	-1044.877008	p= 13.70829	q= 99.00000				-
	M8	-1044.877033	ω= 1.29072	p0= 0.99999	p= 13.70723	q= 99.00000	(p1= 0.00001)	-
N	M0	-2369.337993	ω0= 0.49624					-
	M1	-2361.034593	p0= 0.65260	p1= 0.34740	ω= 1.000			-
	M2	-2356.976004	ω= 3.52805	p0=0.86712	p1=0.00000	p2=0.13288		**13P*, 119A****
	M3	-2356.488425	ω= 3.52802	p0= 0.76589	p1= 0.10123	p2= 0.13289		1M*, 13P**, 70Q**, 119A**, 187S**, 197S**, 220A**, 362T**, 377D**, 383P**
	M7	-2362.597921	p= 0.00500	q= 0.00725				-
	M8	-2356.488487	ω= 3.52947	p0= 0.97370	p= 6.92592	q= 99.00000	(p1= 0.13274)	**13P**, 119A****

*, codons with a posterior probability greater than 0.95 belonging to the positively selected class ($\omega > 1$); **, codons at which $P >$ 0.99.

l, log-likelihood score

Table S4. Statistical analysis to avoid false estimations of sites under positive pressure selection.

Gene	Models compared	-2Δl	d.f	dN/dS
S	M1 vs M2	49.68**	2	27.5
	M7 vs M8	49.17**	2	20.37
N	M1 vs M2	8.12**	2	3.53
	M7 vs M8	49.17**	2	3.53

Neutral models (M1 and M7) were compared to selection models (M2 and M8)
*, $P < 0.05$ **, $P < 0.01$ ($\chi^2_{0.05,2}$= 3.84, $\chi^2_{0.01,2}$= 6.63). Δl: likelihood-ratio statistic; d.f., degrees of freedom between nested models.

We gratefully acknowledge the following Authors from the Originating laboratories responsible for obtaining the specimens, as well as the Submitting laboratories where the genome data were generated and shared via GISAID, on which this research is based.

All Submitters of data may be contacted directly via www.gisaid.org

Authors are sorted alphabetically.

Accession ID	Originating Laboratory	Submitting Laboratory	Authors
EPI_ISL_1225874	"AK State Public Health Lab, State Health Department"	Genomics and Discovery, Respiratory Viruses Branch, Division of Viral Diseases, Centers for Disease Control and Prevention	Anna Montmayeur; Anna Uehara; Ben L. Rambo-Martin; Clinton R. Paden; Dhwani Batra; Haibin Wang; Jasmine Padilla; Jing Zhang; Justin Lee; Katie Dillon; Krista Queen; Kristen Knipe; Kristine Lacek; Lori Rowe; Mark Burroughs; Matthew Schmerer; Mili Sheth; Peter W. Cook; Rachel Marine; Sam Shepard; Sarah Nobles; Shoshona Le; Suxiang Tong; Yan Li; Ying Tao
EPI_ISL_1302479	"InMedica"	Lithuanian University of Health Sciences Hospital, Department of Genetics and Molecular Medicine	Astra Vitkauskiene; Darius Cereskevicius; Inga Nasvytiene; Mantas Sarauskas; Marius Sukys; Rasa Ugenskiene; Renaldas Jurkevicius; Zivile Zemeckiene
EPI_ISL_1081959	"Stefan S. Nicolau"Institute of Virology	"Stefan S. Nicolau"Institute of Virology	Adriana Plesa; Alina Nastasie; Ana Iulia Neagu; Anca Botezatu; Camelia Sultana; Carmen Cristina Diaconu; Coralia Bleotu; Cristina Mambet; Denisa Dragu; Gabriela Anton; Ioana Pitica; Iulia Virginia Iancu; Laura Grecu; Laura Necula; Lilia Matei; Marinela Bostan; Mihaela Economescu; Mirela Mihaila; Saviana Nedeianu; Simona Ruta
EPI_ISL_632310	1-Laboratory of Microbiology, National Reference Lab, Charles Nicolle Hospital; 2-University of Tunis ElManar, Faculty of Medicine of Tunis, LR99ES09, Tunis, Tunisia	1-Clinical and Experimental Pharmacology Lab, LR16SP02, National Center of Pharmacovigilance, University of Tunis El Manar, Tunis, Tunisia. 2-Neurodegenerative diseases and psychiatric troubles, LR18SP03, Razi Hospital, University of Tunis El Manar, Tunis, Tunisia. 3-Ministry of Health, National Observatory of New and Emerging Diseases, 1006, Tunis, Tunisia	Alia Ben Kahla; Gaies Emna; Ilhem Boutiba-Ben Boubaker; Imen Kacem; Imen Mkada; Jalila Ben Khelil; Maher Kharrat; Mouna Ben Sassi; Mouna Safer; Nissaf Ben Alaya; Riadh Daghfous; Riadh Gouider.; Salma Abid; Sameh Trabelsi; Sana Ferjani; Soumaya Rammeh
EPI_ISL_635061, EPI_ISL_635062, EPI_ISL_683329, EPI_ISL_707697, EPI_ISL_707698, EPI_ISL_707700, EPI_ISL_707792, EPI_ISL_707793, EPI_ISL_733499, EPI_ISL_733500, EPI_ISL_763065, EPI_ISL_794735, EPI_ISL_794736, EPI_ISL_794738			
see above	1-Laboratory of Microbiology, National Reference Lab, Charles Nicolle Hospital; 2-University of Tunis ElManar, Faculty of Medicine of Tunis, LR99ES09, Tunis, Tunisia	1-Clinical and Experimental Pharmacology Lab, LR16SP02, National Center of Pharmacovigilance, University of Tunis El Manar, Tunis, Tunisia. 2-Neurodegenerative diseases and psychiatric troubles, LR18SP03, Razi Hospital, University of Tunis El Manar, Tunis, Tunisia. 3-Ministry of Health, National Observatory of New and Emerging Diseases, 1006, Tunis, Tunisia	Alia Ben Kahla; Alia BenKahla; Asma Ferjani; Awatef El MOussi; Awatef El Moussi; Gaies Emna; Guedi Ali Barreh; Guedi Berrabeh; Habiba Ben Romdhane; Hanen El Jebari; Hanen ElJebari; Ilhem Boutiba-Ben Boubaker; Imen Kacem; Imen Mkada; Ines Mdini; Jalila Ben Khelil; Maher Kharrat; Mouna Ben Sassi; Mouna Safer; Nissaf Ben Alaya; Riadh Daghfous; Riadh Gouider.; Rouaa Ben Othman; Salma Abid; Salwa Mrabet; Sameh Trabelsi; Sana Ferjani; Sarra Chamman; Souissi Amira; Soumaya Rammeh; Zaineb Hamzaoui
EPI_ISL_1402509	1. Glowny Inspektorat Sanitarny. 2. Diagnostyka. Laboratoria Medyczne.	1. ViroGenetics - BSL3 Laboratory of Virology, Małopolska Centre of Biotechnology, Jagiellonian University; 2. genXone SA, Research & Development Laboratory	Aleksandra Gidlewicz; Anna Brylak; Gromowski, T.; Grzegorz Nowicki; Jakub Grabowski; Karol Szeszko; Kowalski, M.; Labaj; Maciej Sykulski; Mazur-Panasiuk, N.; Michał Kaszuba; Natalia Drweska-Matelska; P.P.; Pyrc, K.; Sylwia Januszczak; Szulc, P.; Łukasz Krych
EPI_ISL_882752	1.AO Universitaria 'S. Giovanni di Dio e Ruggi D'Aragona, Scuola Medica Salernitana' Hospital / 2.UOC di Virologia e Microbiologia, Università della Campania 'L. Vanvitelli' / 3.AO Universitaria 'Federico II' Napoli Hospital / 4.AORN 'San Giuseppe Moscati' Avellino Hospital / 5.AO 'San Pio - presidio G. Rummo' Benevento Hospital / 6.AO 'Sant'Anna e San Sebastiano' Caserta Hospital / 7.PO 'Maria Santissima Addolorata' Eboli Hospital / 8.Biogem Istituto di Ricerche Genetiche	1. Genome Research Center for Health (CRGS) / 2. Laboratory of Molecular Medicine and Genomics(LMMGe) / 3. Center for Research in Pure and Applied Mathematics (CRMPA)	Alessandro Weisz; Alessia Cossu; Aniello Gentile; Annamaria Salvati; Antonello Saccomanno; Arnolfo Petruzziello; Assunta Sellitto; Carlo Ferravante; Domenico Memoli; Domenico Palumbo; Elena Alexandrova; Emilia Vaccaro; Francesca Marciano; Francesca Rizzo; Gianluigi Franci; Giorgio Giurato; Giovanni Nassa; Giovanni Pecoraro; Giuseppe Fenza; Giuseppe Portella; Gregorio Goffredi; Ilaria Terenzi; Jessica Lamberti; Maddalena Schioppa; Maria Foti; Maria Landi; Marianna Scrima; Mariarosaria Ingino; Massimiliano Galdiero; Maurizio Fumi; Michele Caraglia; Michele Cennamo; Oriana Strianese; Pasquale Pagliano; Rita Greco; Roberta Tarallo; Sonia Amabile; Teresa Rocco; Valeria Mirici Cappa; Vincenzo Rocco; Viola Melone; Vittoria Letizia; Ylenia D'Agostino
EPI_ISL_1361523, EPI_ISL_1577235	1.AO Universitaria 'S. Giovanni di Dio e Ruggi D'Aragona, Scuola Medica Salernitana' Hospital / 2.UOC di Virologia e Microbiologia, Università della Campania 'L. Vanvitelli' / 3.AO Universitaria 'Federico II' Napoli Hospital / 4.AORN 'San Giuseppe Moscati' Avellino Hospital / 5.AO 'San Pio - presidio G. Rummo' Benevento Hospital / 6.AO 'Sant'Anna e San Sebastiano' Caserta Hospital / 7.PO 'Maria Santissima Addolorata' Eboli Hospital / 8.Biogem Istituto di Ricerche Genetiche / 9. U.O.C. di Genetica Medica e di Laboratorio A.O.R.N., Azienda Ospedaliera di Rilievo Nazionale Antonio Cardarelli, Napoli / 10. Centro di riferimento Oncologico della Basilicata (IRCCS-CROB), Rionero in Vulture (PZ) / 11. Presidio Ospedaliero di Agropoli, Agropoli (SA).	1. Genome Research Center for Health (CRGS) / 2. Laboratory of Molecular Medicine and Genomics(LMMGe) / 3. Center for Research in Pure and Applied Mathematics (CRMPA)	Alessandro Weisz (Corresponding Author); Alessia Cossu; Andreina Baj; Aniello Gentile; Annamaria Salvati; Antonello Saccomanno; Arnolfo Petruzziello; Assunta Sellitto; Carlo Ferravante; Domenico Memoli; Domenico Palumbo; Edmondo Adorisio; Elena Alexandrova; Emilia Vaccaro; Fausto Sessa.; Francesca Marciano; Francesca Rizzo (Corresponding Author); Francesco Curcio; Gianluigi Franci; Giorgio Dirani; Giorgio Giurato (Corresponding Author); Giovanni Nassa; Giovanni Pecoraro; Giuseppe Fenza; Giuseppe Portella; Gregorio Goffredi; Ilaria Terenzi; Jessica Lamberti; Maddalena Schioppa; Maria Grazia Foti; Maria Landi; Marianna Scrima; Mariarosaria Ingino; Massimiliano Galdiero; Maurizio Fumi; Michela Iacobellis; Michele Caraglia; Michele Cennamo; Morena D'Avenia; Oriana Strianese; Pasquale Pagliano; Rita Greco; Roberta Tarallo; Rosanna Piluscio; Silvia Zanoli; Simona Semprini; Sonia Amabile; Stefania Marzinotto; Teresa Rocco; Valeria Mirici Cappa; Vincenzo Rocco; Viola Melone; Vittoria Letizia; Vittorio Sambri; Ylenia D'Agostino
EPI_ISL_640107, EPI_ISL_640115, EPI_ISL_640127, EPI_ISL_700446, EPI_ISL_700555, EPI_ISL_1040770, EPI_ISL_1040773, EPI_ISL_1040790, EPI_ISL_1040792			
see above	2 Military Hospital wc MAA	NHLS/UCT	Arash Iranzadeh; Bruna Galvao; Carolyn Williamson; Deelan Doolabh; Diana Hardie; Innocent Mudau; Kruger Marais; Lynn Tyers; Marvin Hsiao; Stephen Korsman
EPI_ISL_960130	2 Military Hospital wc MAA	National Health Laboratory Service/UCT	Arash Iranzadeh; Bruna Galvao; Carolyn Williamson; Deelan Doolabh; Diana Hardie; Innocent Mudau; Kruger Marais; Lynn Tyers; Marvin Hsiao; Stephen Korsman
EPI_ISL_801518	ABC Algarve	Instituto Nacional de Saude (INSA)	Borges et al
EPI_ISL_498543, EPI_ISL_498546, EPI_ISL_498548	ACT Pathology	Schwessinger Lab	Ashley Jones; Benjamin Schwessinger; Craig Kennedy; Karina Kennedy; Kevin Murray; Megan McDonald; Ming-Dao Chia; Robert Lanfear; Robyn N Hall
EPI_ISL_602622, EPI_ISL_602624, EPI_ISL_602626, EPI_ISL_602627, EPI_ISL_602628	AHRI-Sigal	KRISP, KZN Research Innovation and Sequencing Platform	Cele S; Gazy I; Giandhari J; Karim F; Pillay S; Sigl A; Sigla; Tegally H; Wilkinson E; de Oliveira T
EPI_ISL_918437	AIID	Irish Coronavirus Sequencing Consortium-Teagasc Grange	Aljandro Abner Garcia Leon; Calum Walsh; Fiona Crispie; Gabriel Gonzalez; John Kenny; Matthew McCabe; Michael Carr; Patrick Mallon; Paul Cotter
EPI_ISL_467432, EPI_ISL_467435, EPI_ISL_467453, EPI_ISL_467462, EPI_ISL_467467, EPI_ISL_467470, EPI_ISL_467472			
see above	AMPATH-DBN	KRISP, KZN Research Innovation and Sequencing Platform	Chimukangara B; Giandhari J; Khan S; Lessells R; Mdlalose K; Pillay S; Tegally H; Wilkinson E; York D; de Oliveira T
EPI_ISL_2441206, EPI_ISL_2441213	AP SSO	CSIR-Centre for Cellular and Molecular Biology-INSACOG	Amareshwar Vodapalli; Ara Sreenivas; Archana Bharadwaj Siva; B Himasri; Divya Tej Sowpati; Karthik Bharadwaj Tallapaka; Lamuk Zaveri; Onkar Kulkarni; Payel Mukherjee; Priya Nurkurthy; Rakesh K Mishra; Shreekant Verma; Sofia Banu; Sumedha Avadhanula; Tulasi Nagabandi; Valli Nagalakshmi Undamatla; Vidhyadhari Methuku
EPI_ISL_1272857	AR Dept. of Health-PHL, Molecular Diagnostics	Centers for Disease Control and Prevention Division of Viral Diseases, Pathogen Discovery	Anna Montmayeur; Anna Uehara; Ben L. Rambo-Martin; Clinton R. Paden; Dhwani Batra; Haibin Wang; Jasmine Padilla; Jing Zhang; Justin Lee; Katie Dillon; Krista Queen; Kristen Knipe; Kristine Lacek; Lori Rowe; Mark Burroughs; Matthew Schmerer; Mili Sheth; Peter W. Cook; Rachel Marine; Sam Shepard; Sarah Nobles; Shoshona Le; Suxiang Tong; Yan Li; Ying Tao
EPI_ISL_527651	AR Dept. of Health-Public Health Lab	Pathogen Discovery, Respiratory Viruses Branch, Division of Viral Diseases, Centers for Disease Control and Prevention	Anna Uehara; Clinton Paden; Haibin Wang; Jing Zhang; Krista Queen; Suxiang Tong; Yan Li; Ying Tao
EPI_ISL_914828	AREA DE SALUD ALAJUELA SUR	Inciensa, Instituto Costarricense de Investigación y Enseñanza en Nutrición y Salud	Adriana Godínez; Claudio Soto-Garita; Estela Cordero; Francisco Duarte; Hebleen Porras; Melany Calderón & Mariel López
EPI_ISL_1067588	AREA DE SALUD BUENOS AIRES	Inciensa, Instituto Costarricense de Investigación y Enseñanza en Nutrición y Salud	Adriana Godínez; Claudio Soto-Garita; Estela Cordero; Francisco Duarte; Hebleen Porras; Melany Calderón & Mariel López
EPI_ISL_914806	AREA DE SALUD CORONADO	Inciensa, Instituto Costarricense de Investigación y Enseñanza en Nutrición y Salud	Adriana Godínez & Melany Calderón; Claudio Soto-Garita; Estela Cordero; Francisco Duarte; Hebleen Porras
EPI_ISL_914803	AREA DE SALUD LA UNION	Inciensa, Instituto Costarricense de Investigación y Enseñanza en Nutrición y Salud	Adriana Godínez; Claudio Soto-Garita; Estela Cordero; Francisco Duarte; Hebleen Porras; Melany Calderón & Mónica Charpentier-Artavia
EPI_ISL_914835	AREA DE SALUD PARAISO-CERVANTES	Inciensa, Instituto Costarricense de Investigación y Enseñanza en Nutrición y Salud	Adriana Godínez; Claudio Soto-Garita; Estela Cordero; Francisco Duarte; Hebleen Porras; Melany Calderón & Mariel López
EPI_ISL_796741	ARS Algarve - Laboratorio Laura Ayres	Instituto Nacional de Saude (INSA)	Borges et al
EPI_ISL_517644, EPI_ISL_517653, EPI_ISL_517658, EPI_ISL_518803, EPI_ISL_518812	Academic Hospital Paramaribo	Erasmus Medical Center	Bas Oude Munnink; Dion Gajadin; Ed Ijzerman; Emmanuelle Munger; Gary Gummels; Ingrid Krishnadath; Lycke Woittiez; Marion Koopmans; Mireille Van de Veer; Princes Wongsowidjojo; Radjesh Ori; Rohma Banwari; Stephen Vreden
EPI_ISL_1479600, EPI_ISL_1560109, EPI_ISL_1562815, EPI_ISL_1649583, EPI_ISL_1651003	Aegis Sciences Corporation	Centers for Disease Control and Prevention Division of Viral Diseases, Pathogen Discovery	Adrian Paskey; Alec Vest; Benjamin Rambo-Martin; Christopher Gulvick; Clinton R. Paden; Cyndi Clark; Dakota Howard; Darlene Wagner; Dhwani Batra; Dillon Nall; Duncan MacCannell; Ethan Sanders; Holly Houdeshell; Jason Caravas; Kara Moser; Matthew Hardison; Matthew Schmerer; Ola Kvalvaag; Patrick Campbell; Peter W. Cook; Rob Case; Scott Sammons; Shatavia Morrison; Shaun Westlund; Vikramsinha Ghorpade; Yvette Unoarumhi
EPI_ISL_739660	Al-Quds Nutrition and Health Research Institute, Al-Quds University	Al-Quds Nutrition and Health Research Institute, Al-Quds University	Al-Jawabreh, A.; Ereqat, S.; Nasereddin, A.; Rishmawi, C.
EPI_ISL_911696, EPI_ISL_1049508, EPI_ISL_1483588	Alaska State Virology Laboratory	Alaska State Virology Laboratory	Elva House; Jack Chen; Lisa Smith; Ph.D.; Stephanie DeRonde
EPI_ISL_805405	Alberta Precision Labs (APL)	Alberta Precision Labs (APL)	Berenger B; Bernier F; Chui L; Croxen M; Gordon P; Kellner J; Lam LG; Li V; Ma R; Melin A; Pabbaraju K; Tipples G; Wong A; Zelyas N
EPI_ISL_1385811	Alfa Diagnostica LLC	ONCOGENE LLC	ONCOGENE LLC
EPI_ISL_1385804	Alfa Diagnostica, Republic of Moldova	ONCOGENE LLC	ONCOGENE LLC
EPI_ISL_1591214	Algemeen Medisch Labo	Labo Klinische Biologie, UZA	Basil Britto Xavier; Christine Lammens; Herman Goossens; Jasmine Coppens; Marie Le Mercier; Veerle Matheeussen

EPI_ISL_700476, EPI_ISL_700521, EPI_ISL_700548, EPI_ISL_1040749	Alma CDC wc AHC	NHLS/UCT	Arash Iranzadeh; Bruna Galvao; Carolyn Williamson; Deelan Doolabh; Diana Hardie; Innocent Mudau; Kruger Marais; Lynn Tyers; Marvin Hsiao; Stephen Korsman
EPI_ISL_512664, EPI_ISL_527749	Area De Salud Alajuela Norte - Clinica Dr. Marcial Rodriguez	Inciensa, Instituto Costarricense de Investigación y Enseñanza en Nutrición y Salud	Adriana Godinez & Melany Calderon; Claudio Soto-Garita; Estela Cordero; Francisco Duarte; Hebleen Porras
EPI_ISL_770005	Area De Salud Moravia	Inciensa, Instituto Costarricense de Investigación y Enseñanza en Nutrición y Salud	Adriana Godínez; Claudio Soto-Garita; Estela Cordero; Francisco Duarte; Hebleen Porras; Melany Calderón & Mariel López
EPI_ISL_769996, EPI_ISL_770025	Area De Salud San Juan-San Diego-Concepcion 2	Inciensa, Instituto Costarricense de Investigación y Enseñanza en Nutrición y Salud	Adriana Godínez; Claudio Soto-Garita; Estela Cordero; Francisco Duarte; Hebleen Porras; Melany Calderón & Mariel López
EPI_ISL_1483700	Area of Virology, Serology and Virology Division (SAVID), New South Wales Health Pathology Randwick	Area of Virology, Serology and Virology Division (SAVID), New South Wales Health Pathology Randwick	Au, J.; Bull, R.; Deveson, I.; Foster, C.; Jean, T.; Rawlinson, W.; Ruiz Silva, M.; Van Haal, S.
EPI_ISL_527013, EPI_ISL_527018, EPI_ISL_527022	Area of Virology, Serology and Virology Division (SAViD), New South Wales Health Pathology Randwick	Area of Virology, Serology and Virology Division (SAViD), New South Wales Health Pathology Randwick	Rawlinson, W.
EPI_ISL_678371, EPI_ISL_1005540, EPI_ISL_1098798, EPI_ISL_1121976, EPI_ISL_1184504, EPI_ISL_1293048, EPI_ISL_1615592, EPI_ISL_1615598, EPI_ISL_1633352, EPI_ISL_1911190, EPI_ISL_2455220			
see above	Area of Virology, Serology and Virology Division (SAViD), New South Wales Health Pathology Randwick	Virology Research Laboratory; Area of Virology, Serology and Virology Division (SAViD), New South Wales Health Pathology Randwick	Au, J.; Bull, R.; Deveson, I.; Foster, C.; Rawlinson, W.; Ruiz Silva, M.; Van Hal, S.
EPI_ISL_1656801	Arlon	Plateforme de testing Namuroise	Céline Maschietto; Degosserie Jonathan; Denis Olivier; Mullier François; Otto Gaetan
EPI_ISL_1585942, EPI_ISL_1593858	Armed Forces Institute of Pathology (AFIP), Dhaka Cantonment	Genomic Research Lab, BCSIR	Abu Sayeed Mohammad Mahmud; Barna Goswami; Eshrar Osman; Iffat Jahan; Md. Ahasan Habib; Md. Kamrul Islam; Md. Murshed Hasan Sarkar; Md. Saddam Hossain; Md. Salim Khan; Mohammad Mizanur Rahman; Mohammad Mohi Uddin; Mohammad Samir Uzzaman; Shahina Akter; Susane Giti; Tanjina Akhter Banu
EPI_ISL_496540	Armed Forces Medical College	National Centre For Cell Science	Arvind Sahu; DBT's PAN-INDIA 1000 SARS-CoV2 RNA genome sequencing consortium; Dhiraj Paul; Girdhari Lal; Janesh Kumar; Kavita Bala Anand; Kunal Jani; Maharashtra COVID-19 Study Group; Manoj Kumar Bhat; Murlidhar Tambe; Radha Chauhan; Rajesh Karyakarte; Rajiv Mohan Gupta; Santosh Karade; Shelinder Pal Singh Shergill; Sourav Sen; Suvarna Joshi; Vasudevan Seshadri; Yogesh S Shouche
EPI_ISL_1315064	Armed Forces Medical Research Laboratories and Blood Bank, Egypt	Department of Microbial Biotechnology, Genetic Engineering Division, National Research Centre,	Ahmed Elsayed; Ahmed Taha; Ayman Farghaly; Mohamed Khalifa; Mohamed Shemis; Reham Dawood
EPI_ISL_413490	Auckland Hospital	Institute of Environmental Science and Research (ESR)	Erasmus Smit; Gary McAuliffe; Joep de Ligt; Lauren Jelly; Matt Storey; Matthew Blakiston; Sally Roberts; Xiaoyun Ren
EPI_ISL_767896	Australian Clinical Labs	NSW Health Pathology - Institute of Clinical Pathology and Medical Research; Westmead Hospital; University of Sydney	CIDM-PH et al.
EPI_ISL_1180788, EPI_ISL_1583466, EPI_ISL_1583500	Austrian Agency for Health and Food Safety (AGES)	Bergthaler laboratory, CeMM Research Center for Molecular Medicine of the Austrian Academy of Sciences	Andreas Bergthaler; Anna Schedl; Bekir Erguner; Benedikt Agerer; Christoph Bock; Fabian Amman; Jan Laine; Lukas Endler; Maelle Le Moing; Martin Senekowitsch; Michael Schuster; Petr Triska; Thomas Penz
EPI_ISL_882640, EPI_ISL_882642	Azerbaijan National Hematology Center Division of Medical Genetics	Azerbaijan National Hematology Center Division of Medical Genetics	Aghayev Agha Rza
EPI_ISL_968711, EPI_ISL_976760	BCCDC Public Health Laboratory	BCCDC Public Health Laboratory	Ana Pacagnella; Corrinne Ng; Dan Fornika; John Tyson; Kim Macdonald; Kimia Kamelian; Linda Hoang; Loretta Janz; Mel Krajden; Prystajecky Natalie; Robert Azana Terry Snutch; Shannon Russell
EPI_ISL_1628783, EPI_ISL_1628784, EPI_ISL_1628785, EPI_ISL_1628790, EPI_ISL_1628792, EPI_ISL_1628795, EPI_ISL_1628797, EPI_ISL_1628801, EPI_ISL_1628802, EPI_ISL_1629415, EPI_ISL_1629417			
see above	BIO AUSTRAL	UMR PIMIT	Dr Camille Lebarbenchon; Dr David A Wilkinson; Dr Patrick Mavingui; Magali Turpin
EPI_ISL_1219182	BIOR	Latvian Biomedical Research and Study Centre	Daina Pule; Davids Fridmanis; Guntars Zarins; Irena Meistere; Ivars Silamikelis; Janis Klovins; Janis Pjalkovskis; Jurijs Perevoscikovs; Kaspars Megnis; Laila Silamikele; Lauma Freimane; Laura Ansone; Liga Birzniece; Monta Ustinova; Nikita Zrelovs; Uga Dumpis; Una Krumina; Vita Rovite
EPI_ISL_859578, EPI_ISL_859582, EPI_ISL_859604, EPI_ISL_859673, EPI_ISL_859688, EPI_ISL_860013, EPI_ISL_860031, EPI_ISL_860048, EPI_ISL_860049, EPI_ISL_860050, EPI_ISL_860078, EPI_ISL_860090			
see above	BTC, Khalifa University	BTC, Khalifa University	Al Safar et al
EPI_ISL_1265445	BTKLPP Kelas I Makassar	Eijkman Institute for Molecular Biology, Ministry of Research and Technology/National Agency for Research and Innovation; National Institute of Health Research and Development	Amin Soebandrio; Edison Johar; Frilasita A Yudhaputri; Hana Apsari Pawestri; Hidayat Trimarsanto; Iskandar Adnan; Khin Saw Myint; Lydia V. Panggalo; Safarina G Malik; Slamet; Sukma Oktavianthi; Vivi Setiawaty; Willy Agustine
EPI_ISL_791978	Balai Labkes Lampung	National Institute of Health Research and Development	AA; HA; HD; Ikawati; KD; KNA; L; N; Nugraha; Pangesti; Pawestri; Puspa; Puspandari; Setiawaty; Soekarso; Subangkit; T; V; Yurina
EPI_ISL_1627441	Baylor Scott & White-Temple	Baylor Scott & White-Temple	Ari Rao; Caitlin Maloney; Kimberly Walker; Linden Morales; Marcus Volz; Shelby Hendrickson
EPI_ISL_640031, EPI_ISL_640047, EPI_ISL_700453	Beaufort West Hospital wc BWH	NHLS/UCT	Arash Iranzadeh; Bruna Galvao; Carolyn Williamson; Deelan Doolabh; Diana Hardie; Innocent Mudau; Kruger Marais; Lynn Tyers; Marvin Hsiao; Stephen Korsman
EPI_ISL_960141	Beaufort West Hospital wc BWH	National Health Laboratory Service/UCT	Arash Iranzadeh; Bruna Galvao; Carolyn Williamson; Deelan Doolabh; Diana Hardie; Innocent Mudau; Kruger Marais; Lynn Tyers; Marvin Hsiao; Stephen Korsman
EPI_ISL_429993, EPI_ISL_635779, EPI_ISL_730373, EPI_ISL_730559, EPI_ISL_755258, EPI_ISL_878452, EPI_ISL_878477, EPI_ISL_878503, EPI_ISL_878519, EPI_ISL_878571, EPI_ISL_878574			
see above	Biolab Diagnostic Laboratories	Andersen lab at Scripps Research	Ahmad Tibi; Amid Abdelnour with SEARCH Alliance San Diego; Issa Abu-Dayyeh; Lama Hussein; Lina Mohammad; Zein Naber
EPI_ISL_1404588, EPI_ISL_1405965, EPI_ISL_1406179, EPI_ISL_1406195	Biolab Diagnostic Laboratories	Biolab Diagnostic Laboratories	Ahmad Tibi; Amid Abdelnour; Badia Saddedin; Eiad Atwa; Issa Abu-Dayyeh; Lama Hussein; Shayma Ali
EPI_ISL_510526	Biological prevention, army	Biological prevention, army	A.F.; B.S.; Elhoseny; Harty; M.D.; M.G.; M.M. and Gad; Seadawy; Shamel
EPI_ISL_1524330, EPI_ISL_1524332, EPI_ISL_1524333, EPI_ISL_1524335, EPI_ISL_1524344, EPI_ISL_1524347, EPI_ISL_1524348, EPI_ISL_1524352, EPI_ISL_1524360, EPI_ISL_1524362			
see above	Biology Department, College of Science, Al Muthanna University and Public Health Laboratory, Al-Muthanna Health Directorate	Department of Virology, Faculty of Medicine, University of Helsinki, Helsinki, Finland	Alaa Hameed; Ali Jasim; Hussein Alburkat; Murad Munahi; Nihad Al-Rashedi; Olli Vapalahti; Tarja Sironen; Teemu Smura
EPI_ISL_1522838	BioneXt Lab	Laboratoire national de sante, Microbiology, Microbial Genomics Platform	Anke Wienecke-Baldacchino; Catherine Ragimbeau; Fatu Djabi; Jessica Tapp; Lise Pignon; Raoul Salmon; Tamir Abdelrahman; Thibault Ferrandon
EPI_ISL_985060, EPI_ISL_985063, EPI_ISL_985064, EPI_ISL_985068, EPI_ISL_985074, EPI_ISL_985078, EPI_ISL_985080, EPI_ISL_985081, EPI_ISL_985085, EPI_ISL_985088, EPI_ISL_985090, EPI_ISL_985091, EPI_ISL_985092, EPI_ISL_985095, EPI_ISL_985099, EPI_ISL_985102, EPI_ISL_985125, EPI_ISL_985128			
see above	Biorepository and Clinical Virology Laboratory	Ozer Lab	Adeola A. Fowotade; Babafemi O. Taiwo; Egon A. Ozer; Ewean C. Omoruyi; Johnson A. Adeniji; Judd F. Hultquist; Lacy M. Simons; Olubusuyi M. Adewumi; Ramon Lorenzo-Redondo
EPI_ISL_458287	Biosafety Department PCL3	Biosafety Department PCL3	A. and El Kabbaj, S.; Lemriss, S.; Souiri
EPI_ISL_745159, EPI_ISL_745168	Bishop Lavis CDC wc BLP	National Health Laboratory Service (NHLS), Tygerberg	Bronwyn Kleinhans; Eduan Wilkindon; Gert van Zyl; Houriiyah Tegally; Kayla Delaney; Susan Engelbrecht; Tulio de Oliveira; Wolfgang Preiser
EPI_ISL_1040762	Bredasdorp clinic	NHLS/UCT	Arash Iranzadeh; Bruna Galvao; Carolyn Williamson; Deelan Doolabh; Diana Hardie; Innocent Mudau; Kruger Marais; Lynn Tyers; Marvin Hsiao; Stephen Korsman
EPI_ISL_1000999, EPI_ISL_1034760	Bundeswehr Institute of Microbiology	Bundeswehr Institute of Microbiology	Alexandra Rehn; Enrico Georgi; Malena Bestehorn-Willmann; Markus Antwerpen; Mathias Walter; Roman Wölfel; Sabine Zange
EPI_ISL_1371898, EPI_ISL_1371899, EPI_ISL_1371900, EPI_ISL_1371902, EPI_ISL_1371904	C H DE LA POLYNESIE FRANCAISE	CNR Virus des Infections Respiratoires - France SUD	Antonin Bal; Bruno Lina; Gregory Destras; Gwendolyne Burfin; Hadrien Regue; Laurence Josset; Martine Valette; Quentin Semanas
EPI_ISL_1135056	C.H. PRINCESSE GRACE	CNR Virus des Infections Respiratoires - France SUD	Antonin Bal; Bruno Lina; Gregory Destras; Gwendolyne Burfin; Hadrien Regue; Laurence Josset; Martine Valette; Quentin Semanas
EPI_ISL_467516	CAPRISA	KRISP, KZN Research Innovation and Sequencing Platform	Chimukangara B; Giandhari J; Khan S; Lessells R; Mdlalose K; Pillay S; Tegally H; Wilkinson E; York D; de Oliveira T
EPI_ISL_605780	CEIRS Data Processing and Coordinating Center, St. Jude Center of Excellence for Influenza Research and Surveillance (CEIRS)	CEIRS Data Processing and Coordinating Center, St. Jude Center of Excellence for Influenza Research and Surveillance (CEIRS)	A.E.; Ali; El-Guindy; El-Sayes, M.; El-Shesheny, R.; El-Taweel, A.; Gomaa, M.; Kamel; Kandeil, A.; Kayali, G.; Kayed; Kutkat, O.; M.A.; M.N.; Mahmoud; Mahrous, N.; Moatasim, Y.; Mostafa, A.; N.M.; Naguib, A.; Roshdy; S.H.; Shehata, M.; Showky, S.; W.H.; Webby, R.
EPI_ISL_1628690, EPI_ISL_1628693, EPI_ISL_1628695, EPI_ISL_1628697, EPI_ISL_1628709, EPI_ISL_1628710, EPI_ISL_1628711, EPI_ISL_1628722, EPI_ISL_1628740, EPI_ISL_1628753, EPI_ISL_1628755, EPI_ISL_1628776, EPI_ISL_1628812, EPI_ISL_1628813, EPI_ISL_1628815, EPI_ISL_1628821, EPI_ISL_1628822, EPI_ISL_1628834, EPI_ISL_1628837, EPI_ISL_1628839, EPI_ISL_1628840, EPI_ISL_1628842, EPI_ISL_1628874, EPI_ISL_1628878, EPI_ISL_1628881, EPI_ISL_1628885, EPI_ISL_1628886, EPI_ISL_1628953, EPI_ISL_1628975, EPI_ISL_1628980, EPI_ISL_1628982, EPI_ISL_1628985, EPI_ISL_1628986, EPI_ISL_1628994, EPI_ISL_1628998, EPI_ISL_1629037, EPI_ISL_1629041, EPI_ISL_1629128, EPI_ISL_1629131, EPI_ISL_1629133, EPI_ISL_1629134, EPI_ISL_1629143, EPI_ISL_1629145, EPI_ISL_1629149, EPI_ISL_1629151, EPI_ISL_1629152, EPI_ISL_1629153, EPI_ISL_1629154, EPI_ISL_1629156, EPI_ISL_1629160, EPI_ISL_1629163, EPI_ISL_1629351, EPI_ISL_1629353, EPI_ISL_1629354, EPI_ISL_1629355, EPI_ISL_1629380			
see above	CERBALLIANCE	UMR PIMIT	Dr Camille Lebarbenchon; Dr David A Wilkinson; Dr Patrick Mavingui; Magali Turpin
EPI_ISL_1013100, EPI_ISL_1013105, EPI_ISL_1013107, EPI_ISL_1013112, EPI_ISL_1013117, EPI_ISL_1013123, EPI_ISL_1013124, EPI_ISL_1013125, EPI_ISL_1013128, EPI_ISL_1013137, EPI_ISL_1013141, EPI_ISL_1013142, EPI_ISL_1013144, EPI_ISL_1013152, EPI_ISL_1013165, EPI_ISL_1013167, EPI_ISL_1013227, EPI_ISL_1013232, EPI_ISL_1013238, EPI_ISL_1013239, EPI_ISL_1013252, EPI_ISL_1013266, EPI_ISL_1013269, EPI_ISL_1013274, EPI_ISL_1013282, EPI_ISL_1013285, EPI_ISL_1013302, EPI_ISL_1013310, EPI_ISL_1013312, EPI_ISL_1013313, EPI_ISL_1013322, EPI_ISL_1013328, EPI_ISL_1013330, EPI_ISL_1013355, EPI_ISL_1013356, EPI_ISL_1013362, EPI_ISL_1013366, EPI_ISL_1013379, EPI_ISL_1013387, EPI_ISL_1013391, EPI_ISL_1013393, EPI_ISL_1013395, EPI_ISL_1013396, EPI_ISL_1013401, EPI_ISL_1013402, EPI_ISL_1013403, EPI_ISL_1013406			
see above	CH de Mayotte	National Reference Center for Viruses of Respiratory Infections, Institut Pasteur, Paris	Angela Brisebarre; Camille Capel; Combe Patrice; Etienne Simon-Lorière; Marion Barbet; Maud Vanpeene; Méline Bizard; Sylvie Behillil; Sylvie van der Werf; Vincent Enouf

EPI_ISL_909708, EPI_ISL_909711, EPI_ISL_909713, EPI_ISL_909715, EPI_ISL_909717, EPI_ISL_909722	CH de Mayotte - Laboratoire de Biologie	National Reference Center for Viruses of Respiratory Infections, Institut Pasteur, Paris	Angela Brisebarre; Camille Capel; Combe Patrice; Etienne Simon-Lorière; Marion Barbet; Maud Vanpeene; Méline Bizard; Sylvie Behillil; Sylvie van der Werf; Vincent Enouf
EPI_ISL_861586	CHLO	Instituto Nacional de Saude (INSA)	Borges et al
EPI_ISL_1628860, EPI_ISL_1629032	CHOR	UMR PIMIT	Dr Camille Lebarbenchon; Dr David A Wilkinson; Dr Patrick Mavingui; Magali Turpin
EPI_ISL_1336575, EPI_ISL_1336576, EPI_ISL_1336580, EPI_ISL_1336583, EPI_ISL_1336586, EPI_ISL_1336587, EPI_ISL_1336588, EPI_ISL_1336589			
see above	CHR LA REUNION FELIX GUYON	CNR Virus des Infections Respiratoires - France SUD	Antonin Bal; Bruno Lina; Gregory Destras; Gwendolyne Burfin; Hadrien Regue; Laurence Josset; Martine Valette; Quentin Semanas
EPI_ISL_1628723, EPI_ISL_1628724, EPI_ISL_1628736, EPI_ISL_1628737, EPI_ISL_1628804, EPI_ISL_1628805, EPI_ISL_1628806, EPI_ISL_1628808, EPI_ISL_1628809, EPI_ISL_1628811, EPI_ISL_1628826, EPI_ISL_1628830, EPI_ISL_1628832, EPI_ISL_1628833, EPI_ISL_1628889, EPI_ISL_1628925, EPI_ISL_1629002, EPI_ISL_1629003, EPI_ISL_1629027, EPI_ISL_1629057, EPI_ISL_1629061, EPI_ISL_1629065, EPI_ISL_1629072, EPI_ISL_1629074, EPI_ISL_1629104, EPI_ISL_1629107, EPI_ISL_1629175, EPI_ISL_1629241, EPI_ISL_1629383, EPI_ISL_1629385, EPI_ISL_1629387, EPI_ISL_1629389, EPI_ISL_1629390, EPI_ISL_1629392, EPI_ISL_1629395, EPI_ISL_1629396, EPI_ISL_1629430, EPI_ISL_1629453, EPI_ISL_1629458, EPI_ISL_1629476			
see above	CHU	UMR PIMIT	Dr Camille Lebarbenchon; Dr David A Wilkinson; Dr Patrick Mavingui; Magali Turpin
EPI_ISL_1628747, EPI_ISL_1628751, EPI_ISL_1628756, EPI_ISL_1628757, EPI_ISL_1628758, EPI_ISL_1628760, EPI_ISL_1628761, EPI_ISL_1628762			
see above	CHU NORD	UMR PIMIT	Dr Camille Lebarbenchon; Dr David A Wilkinson; Dr Patrick Mavingui; Magali Turpin
EPI_ISL_852816	CHU Purpan - Laboratoire de Virologie - Institut Fédératif de Biologie	CHU Purpan - Laboratoire de Virologie - Institut Fédératif de Biologie	Boyer P.; Carcenac R.; Dubois M.; Harter A.; Izopet J.; Latour J.; Ranger N.; Tremeaux P.
EPI_ISL_1526233, EPI_ISL_1526235, EPI_ISL_1526236, EPI_ISL_1526237, EPI_ISL_1526241, EPI_ISL_1526246	CHU REUNION	CNR Virus des Infections Respiratoires - France SUD	Antonin Bal; Bruno Lina; Gregory Destras; Gwendolyne Burfin; Hadrien Regue; Laurence Josset; Martine Valette; Quentin Semanas
EPI_ISL_1370294	CHUV	Laboratory of genomics and metagenomics, Institute of Microbiology, University Hospital Centre and University of Lausanne, Switzerland	Claire Bertelli; Damien Jacot; Gilbert Greub; Sébastien Aeby; Trestan Pillonel
EPI_ISL_683835	CICM	Malaria Research and Training Center (MRTC-Parasito)	Abdoulaye Djimde; Antoine Dara
EPI_ISL_487446, EPI_ISL_487447, EPI_ISL_487448, EPI_ISL_487449, EPI_ISL_487450, EPI_ISL_487451, EPI_ISL_487452, EPI_ISL_487453, EPI_ISL_487454, EPI_ISL_487455, EPI_ISL_487456, EPI_ISL_487457, EPI_ISL_487459, EPI_ISL_487461, EPI_ISL_487462, EPI_ISL_487463, EPI_ISL_487464, EPI_ISL_487465, EPI_ISL_487466			
see above	CICM-Mali	Bundeswehr Institut of Microbiology	Antwerpen; Bestehorn-Willmann; Dürr; Heitzer; Kouriba; Maiga; Quedraogo; Rehn; Sangaré; Sogodogo; Traoré; Walter; Wölfel; Zimmermann
EPI_ISL_1299497	CMA Dano	Centre Muraz	Abdoul-Salam Ouedraogo; Ange Badjo; Armel Poda; Arsène Somé; Arsène Zongo; Essia Belarbi; Fabian Leendertz; Firmin Kaboré; Grit Schubert; Jasmin Schlotterbeck; Soumeya Ouangraoua; Thérèse Kagone; Yacouba Sawadogo
EPI_ISL_1371786	CNR Virus des Infections Respiratoires - France SUD	CNR Virus des Infections Respiratoires - France SUD	Antonin Bal; Bruno Lina; Gregory Destras; Gwendolyne Burfin; Hadrien Regue; Laurence Josset; Martine Valette; Quentin Semanas
EPI_ISL_1445115	COMPLEXO HOSPITALAR OURO VERDE DE CAMPINAS	Instituto Butantan / Mendelics	Antonio Jorge Martins; Bibiana Santos; Claudia Renata dos Santos Barros; David Schlesinger; Debora Botequio Moretti; Dimas Tadeu Covas; Elaine Cristina Marqueze; Elaine Vieira dos Santos; Erika Freitas; Evandra Strazza Rodrigues; Flavia Aburjaile; José Salvatore Leister Patané; João Paulo Kitajima; Luiz Carlos Junior de Alcantara; Maria Carolina Elias; Marta Giovanetti; Rafael dos Santos Bezerra; Raul Machado Neto; Ricardo Haddad; Rodrigo Tocantins Calado.; Sandra Coccuzzo Sampaio; Simone Kashima; Svetoslav Nanev Slavov; Vagner Fonseca; Vincent Louis Viala
EPI_ISL_1190751, EPI_ISL_1190754, EPI_ISL_1190761, EPI_ISL_1190763, EPI_ISL_1190767	CREMER(Centre de Rechercherches sur les Maladies Emergentes et Ré-émergentes)	TransVIHMI(Recherches Translationnelles sur le VIH et les Maladies Infectieuses)	Ahidjo Ayouba; Celestin Godwe; Christelle Butel; Dowbiss Meta Djomsi; Eitel Mpoudi Ngole; Eric Delaporte; Esemu Livo; Laetitia Serrano; Marcel Tongo; Marie Amougou; Martin Maidadi Foudi; Martine Peeters; Nicole Vidal; Rodrigue Kamga
EPI_ISL_636980	CS Xai Xai	KRISP, KZN Research Innovation and Sequencing Platform	Giandhari J; Ismael N; Nadia Sitoe; Nedio Mabunda; Paulo Arnaldo; Pillay S; Tegally H; Wilkinson E; de Oliveira T
EPI_ISL_447858, EPI_ISL_458072	CSIR-Centre for Cellular and Molecular Biology	CSIR-Centre for Cellular and Molecular Biology	Archana Bharadwaj Siva; Dhiviya Vedagiri; Divya Gupta; Divya Tej Sowpati; Karthik Bharadwaj Tallapaka; Krishnan Harinivas Harshan; Lamuk Zaveri; Namami Gaur; Payel Mukherjee; Priya Singh; Purushotham Vodnala; Rakesh K Mishra; Sakshi Shambhavi; Santosh Kumar Kuncha; Shagufta Khan; Sofia Banu; Tulasi Nagabandi; Vishal Sah
EPI_ISL_2373404, EPI_ISL_2373426, EPI_ISL_2441540, EPI_ISL_2441616	CSIR-Centre for Cellular and Molecular Biology	CSIR-Centre for Cellular and Molecular Biology-INSACOG	; Amareshwar Vodapalli; Ara Sreenivas; Archana Bharadwaj Siva; B Himasri; Blessy B John; Divya Tej Sowpati; Karthik Bharadwaj Tallapaka; Lamuk Zaveri; Onkar Kulkarni; Payel Mukherjee; Priya Nurkurthy; Rakesh K Mishra; Sharath Chandra Thota; Shreekant Verma; Sofia Banu; Sumedha Avadhanula; Tulasi Nagabandi; Valli Nagalakshmi Undamatla; Vidhyadhari Methuku; Viswagithe S L
EPI_ISL_2441409, EPI_ISL_2441443, EPI_ISL_2441454	CSIR-National Environmental Engineering Research Institute	CSIR-Centre for Cellular and Molecular Biology-INSACOG	Amareshwar Vodapalli; Ara Sreenivas; Archana Bharadwaj Siva; B Himasri; Divya Tej Sowpati; Karthik Bharadwaj Tallapaka; Krishna Khairnar; Lamuk Zaveri; Onkar Kulkarni; Payel Mukherjee; Priya Nurkurthy; Rakesh K Mishra; Shreekant Verma; Sofia Banu; Sumedha Avadhanula; Tulasi Nagabandi; Valli Nagalakshmi Undamatla; Vidhyadhari Methuku
EPI_ISL_1096140	Cambodian National Public Health Laboratory, National Institute of Public Health	Virology Unit, Institut Pasteur du Cambodge	Chau Darapheak; Chin Savuth; Erik A Karlsson; Kraing Sidonn; Ly Sovann; Sokhoun Yann; Veasna Duong; Yi Sengdoeurn
EPI_ISL_576371, EPI_ISL_576372, EPI_ISL_857315, EPI_ISL_857329, EPI_ISL_857333, EPI_ISL_857334, EPI_ISL_857337, EPI_ISL_857343, EPI_ISL_857345, EPI_ISL_862787, EPI_ISL_862788, EPI_ISL_862807, EPI_ISL_862808, EPI_ISL_862811, EPI_ISL_862813, EPI_ISL_890222, EPI_ISL_890228, EPI_ISL_890230, EPI_ISL_907087, EPI_ISL_907088, EPI_ISL_907089, EPI_ISL_907112			
see above	Cancer Biology Department, National Cancer Institute	Cancer Biology Department, National Cancer Institute	A.A.; A.E.; A.N.; Abouelhoda, M.; Ahmed; Bahnassy; Elhosieny; F.W.; Gad; H.K.; Hafez; Hamdy; M.G.; M.M.; M.S.; O.S.; Sedawy; Soliman; Soliman, L.; Zekri
EPI_ISL_579060, EPI_ISL_579066, EPI_ISL_579426, EPI_ISL_579448, EPI_ISL_579478, EPI_ISL_579496, EPI_ISL_622795, EPI_ISL_622799, EPI_ISL_622809, EPI_ISL_622810, EPI_ISL_622813, EPI_ISL_732963, EPI_ISL_755622, EPI_ISL_794627, EPI_ISL_843199			
see above	Canterbury Health Laboratories	Institute of Environmental Science and Research (ESR)	Anja Werno; Antje van der Linden; Arlo Upton; Chris Mansell; David Hammer; Dragana Drinkovic; Erasmus Smit; Gary McAuliffe; Hana Sofia Andersson; Hermes Perez; James Ussher; Jill Sherwood; Jing Wang; Joep de Ligt; Josh Freeman; Julia Howard; Juliet Elvy; Lauren Jelly; Mary DeAlmeida; Matt Blakiston; Matt Storey; Matthew Rogers; Max Bloomfield; Michael Addidle; Michelle Balm; Muhammad Faisal; Nikki Freed; Olin Silander; Sally Roberts; Sarah Jefferies; Sharmini Muttaiyah; Susan Morpeth; Susan Taylor; Timothy Blackmore; Vani Sathyendran; Veronica Playle; Virginia Hope; Xiaoyun Ren
EPI_ISL_459862	Center for Genome Regulation (CRG)	Center for Mathematical Modeling and Center for Genome Regulation. Santiago, Chile	Allende ML; Gaete A; González M.; Maass A; Palma R; Travisany D; Urra C; Varas M
EPI_ISL_1546086	Center for Laboratory Medicine St. Gallen	Center for Laboratory Medicine St. Gallen	Yannick Gerth
EPI_ISL_583692, EPI_ISL_853907	Center for Virology, Medical University of Vienna	Bergthaler laboratory, CeMM Research Center for Molecular Medicine of the Austrian Academy of Sciences	Adi Steinrigl; Alexander Lercher; Alexandra Popa; Andreas Bergthaler; Anna Schedl; Benedikt Agerer; Christian Paar; Christoph Bock; Christoph Bock; Daniela Schmid; Dorothee von Laer; Elisabeth Puchhammer-Stoeckl; Franz Allerberger; Gernot Walder; Gregor Hörmann; Guenter Weiss; Gunther Vogl; Henrique Colaco; Jakob-Wendelin Genger; Jan Laine; Judith Aberle; Kinga Rigler-Hohenwarter; Lukas Endler; Manfred Nairz; Mark Smyth; Martin Senekowitsch; Martin Senekowitsch; Michael Schuster; Michael Schuster; Peter Hufnagl; Peter Obrist; Rainer Gattringer; Sabine Sussitz-Rack; Stephan Aberle; Thomas Penz; Wegene Borena
EPI_ISL_678248	Center for public health - Skopje	Research Center for Genetic Engineering and Biotechnology "Georgi D. Efremov" , Macedonian Academy of Sciences and Arts	RCGEB - MASA
EPI_ISL_430819	Center of Scientific Excellence for Influenza Viruses,National Research Centre (NRC), Egypt.	Center of Scientific Excellence for Influenza Viruses,National Research Centre (NRC), Egypt.	Abo Shama; Ahmed E Kayed; Ahmed El-Taweel; Ahmed Kandeil; Ahmed Mostafa; Amal Naguib; M Noura; Mahmoud Shehata; Mina Kamel; Mohamed Ahmed Ali; Mohamed El Sayes; Mokhtar Gomaa; Nancy M. El Guindy; Omnia Kutkat; Rabeh El-Shesheny; Sara Mahmoud; Shymaa Showky Ahmed; Wael Roshdy; Yassmin Moatasim
EPI_ISL_940986, EPI_ISL_942011	Centers for Disease Control and Prevention, Dengue Branch	Centers for Disease Control and Prevention, Dengue Branch	Betzabel Flores; Gabriela Paz-Bailey; Gilberto A. Santiago; Glenda Gonzalez; Jorge L. Munoz-Jordan; Keyla Charriez
EPI_ISL_857314, EPI_ISL_1381386	Centers for Disease Control, R.O.C. (Taiwan)	Centers for Disease Control, R.O.C. (Taiwan)	Ji-Rong Yang; Jung-Jung Mu; Jung-Jung-Mu; Ming-Tsan Liu; Yu-Chi Lin
EPI_ISL_815255	Centogene	Centogene	Krishna Kumar Kandaswamy; Peter Bauer; Vivi Hue-Trang Lieu
EPI_ISL_794603, EPI_ISL_794604	Central Laboratories, Egyptian Ministry of Health and Population	Central Laboratories, Egyptian Ministry of Health and Population	A.E.; Ali; El Guindy; El Sayes; El Taweel, A.; El-Shesheny, R.; Gomaa, M.; Kamel; Kandeil, A.; Kayali, G.; Kayed; Khalifa; Kutkat, O.; M.A.; M.K.; M.N.; Mahmoud; Mahrous, N.; Moatasim, Y.; Mostafa, A.; N.M.; Naguib, A.; Roshdy; S.H.; Saleh, M.; Shawky, S.; Shehata, M.; W.H.
EPI_ISL_529032	Central Molecular Microbiology Laboratory and Next Generation Sequencing Reference Laboratory, Clinical and Chemical Pathology Department, Faculty of Medicine, CAIRO UNIVERSITY	Next Generation Sequencing Reference Laboratory, Faculty of Medicine, CAIRO UNIVERSITY and The Center for Genome and Microbiome Research, Faculty of Pharmacy, CAIRO UNIVERSITY	May Abdelfattah; May Sherif Soliman; Ramy Karam Aziz
EPI_ISL_529031	Central Molecular Microbiology Laboratory, Clinical and Chemical Pathology Department, Faculty of Medicine, CAIRO UNIVERSITY	Next Generation Sequencing Reference Laboratory, Faculty of Medicine, Cairo University and The Center for Genome and Microbiome Research, Faculty of Pharmacy, CAIRO UNIVERSITY	May Abdelfattah; May Sherif Soliman; Ramy Karam Aziz
EPI_ISL_1633291, EPI_ISL_1633293	Central Public Health Lab, National Public Health Organization	Central Public Health Lab, National Public Health Organization	Kyriaki Tryfinopoulou et al
EPI_ISL_693471, EPI_ISL_693472, EPI_ISL_693473, EPI_ISL_693475, EPI_ISL_693478, EPI_ISL_693482, EPI_ISL_1307670, EPI_ISL_1307674, EPI_ISL_1307678, EPI_ISL_1322321, EPI_ISL_1322322, EPI_ISL_1322324, EPI_ISL_1322325, EPI_ISL_1322326, EPI_ISL_1322328			
see above	Central Public Health Laboratory	National Public Health Laboratory, National Centre for Infectious Diseases	Esorom Daoni; Lin Cui; Raymond Tzer Pin Lin; Sophie Octavia; Theresa Palou; Tze Minn Mak; Zhenyang Zhou
EPI_ISL_978512	Central Public Health Laboratory - LACEN -Bahia, Salvador, Brazil	Central Public Health Laboratory - LACEN -Bahia, Salvador, Brazil	Arabela Leal; Breno Dominguez; Felicidade Pereira; Jaqueline Gomes; Luciana Oliveira; Luiz Alcantara; Marcela Gómez; Marta Giovanetti; Patrícia Cajado; Stephane Tosta; Vagner Fonseca; Vanessa Nardy
EPI_ISL_885143	Central public health laboratory	Molecular Diagnostics Department, Central public health laboratory	Dalia, F.; Dler, H.; Dlshad, H.; F.-A.; Fahmi, A.; Furat, S.; Hemdad, A.; Hemn; M. and Idrees, H.; Mohsen, A.; Sharmeen
EPI_ISL_961839, EPI_ISL_961840	Centrala laboratorija	Latvian Biomedical Research and Study Centre	Davids Fridmanis; Ivars Silamikelis; Jana Osite; Janis Pjalkovskis; Janis Klovins; Jurijs Perevoscikovs; Kaspars Megnis; Laila Silamikele; Lauma Freimane; Laura Ansone; Liga Birzniece; Marta Priedite; Monta Ustinova; Nikita Zrelovs; Uga Dumpis; Vita Rovite

EPI_ISL_1357601, EPI_ISL_1357603, EPI_ISL_1357604	Centre For Biotechnology Research And Development	THE AFRICA GENOMICS CENTRE AND CONSULTANCY	Adede Hawi; Cecilia Waruhiu; Damaris Matoke-Muhia; George Michuki; John Njuguna; Lilian Kanjau; Ravena Mubichi
EPI_ISL_1357597	Centre For Biotechnology Research And Development	THE AFRICA GENOMICS CENTRE AND CONSULTANCY LIMITED	Adede Hawi; Cecilia Waruhiu; Damaris Matoke-Muhia; George Michuki; John Njuguna; Lilian Kanjau; Ravena Mubichi
EPI_ISL_458000	Centre For Biotechnology Research and Development	Centre For Biotechnology Research and Development	C.N. and Michuki; D.K.; G.N.; J.O.; Kimotho, J.; Matoke-Muhia; Muuo; Ochwoto, M.; S.L.; S.N.; Symeker; Waruhiu; Zablon
EPI_ISL_1623055	Centre Hospitalier Universitaire Clermont-Ferrand	CHU Clermont-Ferrand, service de virologie	Bisseux Maxime; Combes Patricia; Henquell Cécile; Mirand Audrey
EPI_ISL_1299495	Centre Hospitalo Universitaire Souro Sanou	Centre Muraz	Abdoul-Salam Ouedraogo; Ange Badjo; Armel Poda; Arsène Somé; Arsène Zongo; Essia Belarbi; Fabian Leendertz; Firmin Kaboré; Grit Schubert; Jasmin Schlotterbeck; Soumeya Ouangraoua; Thérèse Kagone; Yacouba Sawadogo
EPI_ISL_788933	Centre de Recherche et de Formation en Infectiologie Guinée	TransVIHMI, IRD/INSERM/Monpellier University	Abdoul Karim SOUMAH; Abdoulaye TOURE; Ahidjo AYOUBA; Alimou CAMARA; Alpha Kabinet KEITA; Bouna Yatassaye; Christelle BUTEL; Eric DELAPORTE; Jean-louis MONEMOU; Joel KOIVOGUI; Kaba KOUROUMA; Laetitia SERRANO; Mamadou Bhoye KEITA; Mamadou Saliou BAH; Mamadou Saliou SOW; Mandiou DIAKITE; Martine PEETERS; Moriba POVOGUI; Penda Malhado DIALLO; Sakoba KEITA
EPI_ISL_539573, EPI_ISL_539574, EPI_ISL_539575, EPI_ISL_539576	Centre de Recherches Medicales de Lambarene (CERMEL)	Department of Emerging Infectious Diseases, Institute of Tropical Medicine, Nagasaki University	Akim A. Adegnika; Bertrand Lell; Haruka Abe; Jiro Yasuda; Rodrigue Bikangui; Yuri Ushijima
EPI_ISL_1615655	Centre de Recherches Médicales de Lambaréné (CERMEL)	Centre de Recherches Médicales de Lambaréné (CERMEL)	Armel MINTSA NDONG; Bertrand Lell and Ayola Akim Adegnika; Bénédicte NDEBOKO; Georgelin NGUEMA ONDO; Gédéon Prince Manouana; Jean Bernard LEKANA-DOUKI; Joël-Fleury DJOBA SIAWAYA; Rodrigue BIKANGUI; Samira ZOA ASSOUMOU; Srinivas reddy Pallerla; Steffen Borrmann; Thirumalaisamy P. Velavan
EPI_ISL_978534, EPI_ISL_978535, EPI_ISL_978537, EPI_ISL_978539, EPI_ISL_978540, EPI_ISL_978541, EPI_ISL_978542, EPI_ISL_978545, EPI_ISL_978546, EPI_ISL_978547, EPI_ISL_978548, EPI_ISL_978549, EPI_ISL_978550, EPI_ISL_978551			
see above	Centre de Virologie des Maladies infectueuses Tropicales	Functional Genomic Platform UATRS-biology, CNRST	; Abdelilah LARAQUI; Abderrazzak Rfaki; Elmostafa BENAISSA; Elmostafa EL FAHIME; Elmostafa EL FAHIME.; Farida HILALI; Hemlali Mouhssine; Khalid ENNIBI; Khalid ENNIBI.; Marouane MELLOUL; Mly Abdelaziz ELALAOUI; Mostafa ELOUENNASS; Nadia Touil; Safaa GHOULAM; Safaa Ghoulam; Sanaa ALAOUI-Amine; Taha Chouati; Tahar BAJJOU; Yassine SEKHSOKH
EPI_ISL_471456, EPI_ISL_471457, EPI_ISL_471458, EPI_ISL_471459, EPI_ISL_471460	Centre de Virologie des Maladies Tropicales	Functional Genomic Platform/Service Analyses Biologique/UATRS/ Centre National Pour la Recherche Scientifique Et Technique (CNRST)	Abdelillah LARAQUI; Abdelkader LAATIRIS; Ahmed REGGAD; Elmostafa EL FAHIME; Farida HILALI; Hicham EL ANNAZ; Idriss-Amine LAHLOU; Khalid ENNIBI; Marouane MELLOUL; Mly Abdelaziz ELALAOUI; Mostafa ELOUENNASS; Nadia TOUIL; Rachid ABI; Rida TAGAJDID; Safae ELKOCHRI; Sanaa ALAOUI-Amine; Tahar BAJJOU; Yassine SEKHSOKH; Youssef AKHOUAD; Zohour KASMY
EPI_ISL_428670, EPI_ISL_428672	Centre for Dengue Research	Centre for Dengue Research	Ananda Wijewickrama; Chandima Jeewandara; Damayanthi Idampitiya; Deshni Jayathilaka; Dinuka Ariyaratne; Diyanath Ranasinghe; Eranga Narangoda; Laksiri Gomes; Neelika Malaige; Neelika Malavige
EPI_ISL_1233092, EPI_ISL_1233095, EPI_ISL_1239364, EPI_ISL_1533830, EPI_ISL_1533834	Centre for Dengue Research and AICBU, Department of Immunology and Molecular Medicine	Centre for Dengue Research and AICBU, Department of Immunology and Molecular Medicine	Chandima Jeewandara; Deshan Madhusanka; Deshni Jayathilaka; Dinuka Ariyaratne; Diyanath Ranasinghe; Gathsaurie Neelika Malavige; Laksiri Gomes; Tibutius Thanesh Pramanayagam
EPI_ISL_654794	Centre for Human Virology & Genomics, Nigerian Institute of Medical Research	Centre for Human Virology & Genomics, Nigerian Institute of Medical Research	Shaibu, J.
EPI_ISL_413595	Centre for Infectious Diseases and Microbiology Laboratory Services	NSW Health Pathology - Institute of Clinical Pathology and Medical Research; Westmead Hospital; University of Sydney	Carter I; Chen SC; Eden J-S; Gall; Gray K; Holmes EC; Kok J and Dwyer DE for the 2019-nCoV Study Group*; Lam C; M; Maddocks S; O'Sullivan MV; Rahman H; Rockett R; Sintchenko V; Timms; V
EPI_ISL_1164310	Centro Hospitalar de Entre o Douro e Vouga (CHEDV)	Institute of Biomedicine (IBiMED), Universidade de Aveiro	Gabriela Moura; Miguel Pinheiro and Manuel Santos; Patricia Arinto; Sofia Marques
EPI_ISL_527742	Centro Nacional De Rehabilitacion Humberto Araya Rojas (Cenare)	Inciensa, Instituto Costarricense de Investigación y Enseñanza en Nutrición y Salud	Adriana Godinez & Melany Calderon; Claudio Soto-Garita; Estela Cordero; Francisco Duarte; Hebleen Porras
EPI_ISL_837554, EPI_ISL_837556, EPI_ISL_837558, EPI_ISL_837561, EPI_ISL_837564, EPI_ISL_837566, EPI_ISL_837567, EPI_ISL_837573, EPI_ISL_837576			
see above	Centro Nacional de Enfermedades Tropicales (CENETROP)	Laboratory of Respiratory Viruses and Measles, Oswaldo Cruz Institute, FIOCRUZ	Ana Carolina Mendonca; Anna Carolina Paixao; Cinthia Avila; Fernando Motta; Luciana Appolinario; Marilda Siqueira on behalf of the Fiocruz COVID-19 Genomic Surveillance Network; Paola Resende; Roxana Loayza
EPI_ISL_1531854, EPI_ISL_1531892	Centro de Diagnostico COVID-19 UABC Tijuana	Andersen lab at Scripps Research	German Ibarra; Jonathan Vincent Baena; Jorge Luis Jimenez Niebla; Manuel Sanchez Alavez; Oscar Efren Zazueta Fierro; SEARCH Alliance San Diego with Idanya Rubi Serafin Higuera
EPI_ISL_941160	Centro de Investigaciones en Microbiología y Biotecnología-UR (CIMBIUR), Facultad de Ciencias Naturales, Universidad del Rosario, Bogotá, Colombia	Centro de Investigaciones en Microbiología y Biotecnología-UR (CIMBIUR), Facultad de Ciencias Naturales, Universidad del Rosario, Bogotá, Colombia Icahn School of Medicine at Mount Sinai, New York, USA	Adriana van de Guchte; Alberto Paniz-Mondolfi; Alejandro Feged-Rivadeneira; Ana S. Gonzalez-Reiche; Andrés Angel; Carolina Flórez; Carolina Hernández; Emilia Mia Sordillo; Felipe González-Casabianca; Hala Alejel Alshammary; Harm van Bakel; Iván Carroll; Jaime Cascante; Jayeeta Dutta; Juan David Ramírez; Luz Helena Patiño; Marina Muñoz; Matthew M. Hernandez; Mauricio Santos-Vega; Mónica Palma-Cuero; Nathalia Ballesteros; Sergio Gomez; Viviana Simon; Zenab Khan
EPI_ISL_941974, EPI_ISL_941998	Centro de Investigaciones en Microbiología y Biotecnología-UR (CIMBIUR), Facultad de Ciencias Naturales, Universidad del Rosario, Bogotá, Colombia	Centro de Investigaciones en Microbiología y Biotecnología-UR (CIMBIUR), Facultad de Ciencias Naturales, Universidad del Rosario, Bogotá, Colombia Instituto Nacional de Salud, Bogotá, Colombia Icahn School of Medicine at Mount Sinai, New York, USA	Adriana van de Guchte; Alberto Paniz-Mondolfi; Ana S. Gonzalez-Reiche; Carolina Flórez; Carolina Hernández; Emilia Mia Sordillo; Hala Alejel Alshammary; Harm van Bakel; Jayeeta Dutta; Juan David Ramírez; Luz Helena Patiño; Marina Muñoz; Matthew M. Hernandez; Nathalia Ballesteros; Sergio Gomez; Viviana Simon; Zenab Khan
EPI_ISL_491934	Centro de Investigaciones, Universidad de Especialidades Espíritu Santo	Institute of Microbiology, Universidad San Francisco de Quito	Belén Prado-Vivar; Bernardo Gutiérrez; Derly Andrade; Edith Lopez; Fernando Espinoza; Gabriel Morey; Gabriel Trueba; Jose Pedro Barberan; Juan Carlos Fernandez; Juan José Guadalupe; Michelle Grunauer; Monica Becerra-Wong; Patricio Rojas-Silva; Paúl Cárdenas; Ruben Armas; Sully Márquez; Verónica Barragán
EPI_ISL_1585487	Centro de Investigación Biomédica de Occidente (CIBO)	Instituto Nacional de Enfermedades Respiratorias (INER): Centro de Investigación en Enfermedades Infecciosas (CIENI)	Alejandro Sanchez-Flores; Alfredo Herrera-Estrella; Alicia Ocaña-Mondragón; Angel Gustavo Salas-Lais; Bernardo Martínez-Miguel; Blanca Taboada; Brenda Irasema Maldonado-Meza; Carla Ivón Herrera-Najera; Carlos F. Arias; Celia Boukadida; Clara Esperanza Santacruz-Tinoco; Concepción Grajales-Muñiz; Consorcio Mexicano de Vigilancia Genómica (CoViGen-Mex). Authors (in alphabetical order): Julio Elias Alvarado-Yaah; Célida Duque Molina; Fernando Fontove-Herrera; Francisco Pulido; Gloria Elena Espinosa-Ayala; Gloria María Molina-Salinas; Gloria Vazquez; Hector Esteban Paz-Juárez; Hector Montoya-Fuentes; Helen Haydee Fernanda Ramírez-Plascencia; José Antonio Enciso-Moreno; José Esteban Muñoz-Medina; José de Jesús Nuñez-Contreras; Juan Bautista Chale-Dzul; Luis Alberto Ochoa-Carrera; Margarita Matías-Florentino; María Guadalupe Santiago-Mauricio; María Guadalupe de Jesús Mireles-Rivera; Nelly Sélem-Mojica; Pavel Isa; Ricardo Grande; Santiago Ávila-Ríos; Victor Eduardo Garcia-Arias; Víctor Hugo Borja-Aburto
EPI_ISL_1585401, EPI_ISL_1585412	Centro de Investigación Biomédica del Noreste (CIBIN)	Instituto Nacional de Enfermedades Respiratorias (INER): Centro de Investigación en Enfermedades Infecciosas (CIENI)	Alejandro Sanchez-Flores; Alfredo Herrera-Estrella; Alicia Ocaña-Mondragón; Angel Gustavo Salas-Lais; Bernardo Martínez-Miguel; Blanca Taboada; Brenda Irasema Maldonado-Meza; Carla Ivón Herrera-Najera; Carlos F. Arias; Celia Boukadida; Clara Esperanza Santacruz-Tinoco; Concepción Grajales-Muñiz; Consorcio Mexicano de Vigilancia Genómica (CoViGen-Mex). Authors (in alphabetical order): Julio Elias Alvarado-Yaah; Célida Duque Molina; Fernando Fontove-Herrera; Francisco Pulido; Gloria Elena Espinosa-Ayala; Gloria María Molina-Salinas; Gloria Vazquez; Hector Esteban Paz-Juárez; Hector Montoya-Fuentes; Helen Haydee Fernanda Ramírez-Plascencia; José Antonio Enciso-Moreno; José Esteban Muñoz-Medina; José de Jesús Nuñez-Contreras; Juan Bautista Chale-Dzul; Luis Alberto Ochoa-Carrera; Margarita Matías-Florentino; María Guadalupe Santiago-Mauricio; María Guadalupe de Jesús Mireles-Rivera; Nelly Sélem-Mojica; Pavel Isa; Ricardo Grande; Santiago Ávila-Ríos; Victor Eduardo Garcia-Arias; Víctor Hugo Borja-Aburto
EPI_ISL_1396507	Centro de Tecnología en Salud Pública de la Universidad Nacional de Rosario	Laboratorio Mixto de Biotecnología Acuática (LMBA) on behalf of 'Proyecto Argentino Interinstitucional de genomica de SARS-CoV-2' (PAIS Consortium)	Adriana Giri; Agustina Cerri; Ana Cavatorta; Ana Paletta; Diego Chouhy; Elisa Bolatti; Elizabeth Tapia (argenTAG); Federico Remes Lenicov; Flavio Spetale; Gastón Viarengo; Ignacio García Labari; Javier Murillo; Joaquín Ezpeleta; Julian Acosta; Laura Angelone; Leandro Ciappina; María Re; Pablo Casal; Pilar Bulacio; Silvana Spinelli; Silvia Arranz; Sofía Lavista Llanos; Vanina Villanova; Victoria Posner
EPI_ISL_735424	Centro de Vigilancia a Saude de Diadema	Instituto Adolfo Lutz, Interdiciplinary Procedures Center, Strategic Laboratory	Claudia Regina Gonçalves; Claudio Tavares Sacchi; Erica Valessa Ramos Gomes; Karoline Rodrigues Campos
EPI_ISL_1590359	Centrālā Laboratorija; Eurofins Genomics Europe Sequencing GmbH	Riga East University Hospital-National Microbiology Reference Laboratory; Eurofins Genomics Europe Sequencing GmbH	Arzu Algulieva; Diāna Dusacka; Dārta Pūpola; Ilva Pole; Jana Osīte; Reinis Vangravs; Reinis Zeltmatis; Sergejs Nikisins; Stella Lapiņa; Ģirts Šķenders
EPI_ISL_406862	Charité Universitätsmedizin Berlin, Institute of Virology; Institut für Mikrobiologie der Bundeswehr, Munich	Charité Universitätsmedizin Berlin, Institute of Virology	Barbara Mühlemann; Christian Drosten; Julia Schneider; Markus Antwerpen; Roman Wölfel; Talitha Veith; Victor M Corman
EPI_ISL_686818	Chiba Prefectural Institute of Public Health	Pathogen Genomics Center, National Institute of Infectious Diseases	Kentaro Itokawa; Makoto Kuroda; Masanori Hashino; Rina Tanaka; Tsuyoshi Sekizuka
EPI_ISL_2450551	City Hospital No 40	WHO National Influenza Centre Russian Federation	Andrey Komissarov; Artem Fadeev; Daria Danilenko; Dmitry Lioznov; Elena Nabieva; Georgii Bazykin; Kirill Varchenko; Ksenia Safina; Kseniya Komissarova; Maria Pisareva; Maria Timofeeva; Mikhail Bakaev; Nikita Yolshin; Olga Shneider; Oula Mansour; Sergey Scherbak; Tamila Musaeva; Veronika Eder
EPI_ISL_1278584, EPI_ISL_1278585, EPI_ISL_1278936, EPI_ISL_1425011	Clalit Health Services Laboratories, Israel	Stern Lab	Stern Lab
EPI_ISL_1250842	Clin & Gen Lab	Molecular Genetics Laboratory, Instituto de Investigaciones Químicas, Universidad Mayor de San Andrés	Aneth Vasquez Michel; Oscar M. Rollano-Peñaloza
EPI_ISL_700432, EPI_ISL_700519	Clinic-in-Asla	NHLS/UCT	Arash Iranzadeh; Bruna Galvao; Carolyn Williamson; Deelan Doolabh; Diana Hardie; Houriiyah Tegally; Innocent Mudau; Kruger Marais; Lynn Tyers; Marvin Hsiao; Stephen Korsman
EPI_ISL_462462, EPI_ISL_462476	Clinical Center, University of Sarajevo	Charite Universitatsmedizin Berlin, Institute of Virology	Almedina Hadzihasanovic-Moro; Amela Dedeic-Ljubovic; Barbara Muehlemann; Christian Drosten; Irma Salimovic-Besic; Jorn Beheim-Schwarzbach; Julia Schneider; Selma Mutevelic; Suzana Arapcic; Talitha Veith; Terry Jones; Victor M Corman
EPI_ISL_1300656, EPI_ISL_1300659	Clinical Center, University of Sarajevo; Unit for Clinical Microbiology	Clinical Center, University of Sarajevo; Unit for Clinical Microbiology	Amela Dedeić-Ljubović; Edina Zahirović; Golubinka Boshevska; Irma Salimović-Bešić; Maja Kuzmanovska; Sandra Vegar-Zubović; Sebija Izetbegović; Suzana Arapčić
EPI_ISL_677721	Clinical Hospital - Shtip	Research Center for Genetic Engineering and Biotechnology "Georgi D. Efremov" , Macedonian Academy of Sciences and Arts	RCGEB - MASA
EPI_ISL_447435	Clinical Microbiology Laboratory, Sheba Medical Center	Stern Lab	Stern Lab
EPI_ISL_581963	Clinical Virology	Clinical Bacteriology	Adrian Egli; Alexander Gensch; Alfredo Mari; Christian Nickel; Hans Hirsch; Hans Pargger; Helena MB Seth-Smith; Julia Bielicki; Karoline Leuzinger; Kirstine K. Soegaard; Madlen Stange; Manuel Battegay;

			Martin Siegemund; Michael Osthoff; Michael Schweitzer; Myrta Brunner; Rita Schneider-SliFemalea; Roland Bingisser; Sarah Tschudin-Sutter; Simon Fuchs; Stefano Bassetti; Tim Roloff
EPI_ISL_1539398, EPI_ISL_1540239	Colorado Department of Public Health and Environment	Colorado Department of Puplic Health and Environment	Diana Ir; Emily A. Travanty; Laura Bankers; Molly C. Hetherington-Rauth; Sarah Elizabeth Totten; Shannon Ely; Shannon R. Matzinger
EPI_ISL_771265, EPI_ISL_955042	Colorado Department of Public Health and Environment	Colorado Department of Puplic Health and Environment	Diana Ir; Emily A. Travanty; Laura Bankers; Molly C. Hetherington-Rauth; Sarah Elizabeth Totten; Shannon Ely; Shannon R. Matzinger
EPI_ISL_1516190	Commonwealth Healthcare Center	Centers for Disease Control and Prevention Division of Viral Diseases, Pathogen Discovery	Alison Laufer Halpin; Ben L. Rambo-Martin; Clinton R. Paden; Dakota Howard; Darlene Wagner; Dave Wentworth; Dhwani Batra; Jasmine Padilla; Justin Lee; Katie Dillon; Krista Queen; Kristen Knipe; Kristine Lacek; Mark Burroughs; Matthew Schmerer; Mili Sheth; Peter Cook; Sam Shepard; Sarah Nobles; Shoshona Le; Suxiang Tong; Vivien Dugan; Yvette Unoarumhi
EPI_ISL_632263, EPI_ISL_632285, EPI_ISL_681312	Communicable Disease Laboratory, Public Health Directorate	Communicable Disease Laboratory, Public Health Directorate	AlAbbas, Z.; AlHujairi, Z.; AlTaif, Z.; AlWasti, H.; Altaif, Z.; Alwasti, H.
EPI_ISL_537380	Complejo Hospitalario Universitario de Vigo	SeqCOVID-SPAIN consortium/IBV(CSIC)	Benito Regueiro and SeqCOVID-SPAIN consortium
EPI_ISL_640015, EPI_ISL_640118, EPI_ISL_700488, EPI_ISL_700506, EPI_ISL_700536, EPI_ISL_700556, EPI_ISL_700599			
see above	Conville CDC wc CVC	NHLS/UCT	Arash Iranzadeh; Bruna Galvao; Carolyn Williamson; Deelan Doolabh; Diana Hardie; Innocent Mudau; Kruger Marais; Lynn Tyers; Marvin Hsiao; Stephen Korsman
EPI_ISL_696508	Conville CDC wc CVC & NHLS/UCT	KRISP, KZN Research Innovation and Sequencing Platform	Arash Iranzadeh; Bruna Galvao; Carolyn Williamson; Deelan Doolabh; Diana Hardie; Emanuel James San; Houriiyah Tegally; Innocent Mudau; Jennifer Giandhari; Kruger Marais; Lynn Tyers; Marvin Hsiao; Stephen Korsman; Sureshnee Pillay; Tulio de Oliveira
EPI_ISL_1091196, EPI_ISL_1499569	Croatian Institute of Public Health	Croatian Institute of Public Health	Irena Tabain; Ivana Ferenčak
EPI_ISL_700448, EPI_ISL_700564, EPI_ISL_700569	D'Almeida Clinic wc DAL	NHLS/UCT	Arash Iranzadeh; Bruna Galvao; Carolyn Williamson; Deelan Doolabh; Diana Hardie; Innocent Mudau; Kruger Marais; Lynn Tyers; Marvin Hsiao; Stephen Korsman
EPI_ISL_1653815, EPI_ISL_1653926	DNA Solution Ltd.	Genomic Research Lab, BCSIR	Abu Sayeed Mohammad Mahmud; Barna Goswami; Eshrar Osman; Iffat Jahan; Kazi Nadim Hasan; Md Firoz Kabir; Md. Abdul Khaleque; Md. Ahasan Habib; Md. Mizanur Rahman; Md. Murshed Hasan Sarkar; Md. Saddam Hossain; Md. Salim Khan; Mohammad Fazle Alam Rabbi; Mohammad Mohi Uddin; Mohammad Samir Uzzaman; Shahina Akter; Sharif Akhteruzzamani; Tanjina Akhter Banu
EPI_ISL_1435789	DNALAB Sdn. Bhd.	Malaysia Genome Institute	Azrin Ahmad; Enizza Kasim; Irni Suhayu Sapian; Mohd Faizal Abu Bakar; Mohd Noor Mat Isa; Nor Azfa Johari.; Nurhezreen Md Iqbal; Shamsidar Sopie; Siti Noraini Othman; Wong Yong Wee; Yusuf Muhammad Noor
EPI_ISL_1583675	DNA Laboratory	Central Public Health Laboratory - LACEN -Bahia, Salvador, Brazil	Arabela Leal; Breno Dominguez; Felicidade Pereira; Jaqueline Gomes; Luciana Oliveira; Luiz Alcantara; Marcela Gómez; Marta Giovanetti; Patrícia Cajado; Stephane Tosta; Vagner Fonseca; Vanessa Nardy
EPI_ISL_416542, EPI_ISL_416543	Dasman Diabetes Institute	Dasman Diabetes Institute	Ebaa AlOzairi; Fahd Al-Mulla; Motasem Melhem; Qais Al-Duwairi; Rasheeba Iqbal; Sara Al-Qabandi; Sumi John
EPI_ISL_812299	Delaware Public Health Lab	Delaware Public Health Lab	Gregory Hovan
EPI_ISL_1423766	Departamento de Microbiología, CDB, Hospital Clínic, Barcelona	SeqCOVID-SPAIN consortium/IBV(CSIC)	Aida Peiró and SeqCOVID-SPAIN consortium; Andrea Vergara; Elisa Rubio; Jéssica Navero; Mikel Martínez
EPI_ISL_1340750, EPI_ISL_1340754, EPI_ISL_1340755, EPI_ISL_1340764	Departamento de Virologia, Laboratorio Central de Salud Pública, Avenida Venezuela y Teniente Escurra, Asunción, Paraguay	Laboratory of Respiratory Viruses and Measles, Oswaldo Cruz Institute, FIOCRUZ	Alice Sampaio Rocha; Ana Carolina Mendonca; Anna Carolina Paixao; Cynthia Vazquez; Fernando Motta; Luciana Appolinario; Marilda Siqueira on behalf of the Fiocruz COVID-19 Genomic Surveillance Network; Paola Resende; Renata Serrano Lopes
EPI_ISL_1013608, EPI_ISL_1013609	Department for Molecular Diagnostics, Centre for Medical Microbiology, Institute of Public Health, Montenegro	Charité Universitätsmedizin Berlin, Institut für Virologie	Barbara Mühlemann; Christian Drosten; Danijela Vujošević; Julia Schneider; Julia Tesch; Jörn Beheim-Schwarzbach; Marija Govedarica; Talitha Veith; Terry Jones; Tobias Bleicker; Victor M Corman
EPI_ISL_471529, EPI_ISL_481483, EPI_ISL_754180, EPI_ISL_754181, EPI_ISL_763062, EPI_ISL_1048367	Department for Virology, Molecular Biology and Genome Research, R. G. Lugar Center for Public Health Research, National Center for Disease Control and Public Health (NCDC) of Georgia.	Department for Virology, Molecular Biology and Genome Research, R. G. Lugar Center for Public Health Research, National Center for Disease Control and Public Health (NCDC) of Georgia.	Adam Kotorashvili; Amiran Gamkrelidze.; Ana Papkiauri; Ann Machablishvili; Anna Kasradze; Davit Tsaguria; Ekaterine Khmaladze; Ekaterine Zangaladze; Ekaterine Zhghenti; Giorgi Gogoladze; Giorgi Tomashvili; Gvantsa Brachveli; Gvantsa Chanturia; Irma Burjanadze; Ketevan Sidamonidze; Khatuna Zakhashvili; Lela Sabadze; Lela Urushadze; Magda Dgebuadze; Maia Alkhazashvili; Mari Gavashelidze; Mariam Zakalashvili; Marine Murtskhvaladze; Meri Pantsulaia; Nato Kotaria; Nino Berishvili; Paata Imnadze; Roena Sukhiashvili; Salome Javashvili; Tamar Jashiashvili; Tata Imnadze; Tea Tevdoradze
EPI_ISL_515084, EPI_ISL_515090, EPI_ISL_515096	Department of Biochemistry, Cell and Molecular Biology	WACCBIP, University of Ghana	A.K.; Adu, B.; Amenga-Etego; Ampofo, W.; Amuzu; Anang; Arjarquah, A.; Asante, I.; Awandare; Bediako, Y.; Boatemaa, L.; Bonney, E.; Bonney, K.; C.M.; D.S.; Eshun, M.; G.A.; G.B.; J.K.; J.M.; Kotey, E.; Kumordjie, S.; Kyei; L.N.; Magnussen, V.; Morang'a; Mutungi; Ngoi; Quashie, P.; Tei-Maya, F.
EPI_ISL_884826, EPI_ISL_884829, EPI_ISL_884831, EPI_ISL_884832, EPI_ISL_884834, EPI_ISL_884837, EPI_ISL_884840, EPI_ISL_884842, EPI_ISL_884844, EPI_ISL_884847, EPI_ISL_884848, EPI_ISL_884849, EPI_ISL_884850, EPI_ISL_884851, EPI_ISL_884852, EPI_ISL_884854, EPI_ISL_944646, EPI_ISL_944647, EPI_ISL_944649, EPI_ISL_944651, EPI_ISL_944652, EPI_ISL_944661, EPI_ISL_944666, EPI_ISL_944667, EPI_ISL_944668, EPI_ISL_944673, EPI_ISL_944677, EPI_ISL_944679, EPI_ISL_944683, EPI_ISL_944688, EPI_ISL_944689, EPI_ISL_944690, EPI_ISL_944691, EPI_ISL_944692, EPI_ISL_944693, EPI_ISL_944694, EPI_ISL_944695, EPI_ISL_944696, EPI_ISL_944697, EPI_ISL_944698, EPI_ISL_944699, EPI_ISL_944700, EPI_ISL_944701, EPI_ISL_944702, EPI_ISL_944706, EPI_ISL_944707, EPI_ISL_944708, EPI_ISL_944709, EPI_ISL_944711, EPI_ISL_944712, EPI_ISL_944713, EPI_ISL_944714, EPI_ISL_944716, EPI_ISL_944719, EPI_ISL_944721, EPI_ISL_944722, EPI_ISL_944724, EPI_ISL_944726			
see above	Department of Biochemistry, Cell and Molecular Biology, West African Centre for Cell Biology of Infectious Pathogens (WACCBIP), University of Ghana	Department of Biochemistry, Cell and Molecular Biology, West African Centre for Cell Biology of Infectious Pathogens (WACCBIP), University of Ghana	A.-K.; A.B.; Abass; Adusei-Poku, M.; Akoriyea; Amenga-Etego; Amoako, E.; Ampofo; Amuzu; Asante, I.; Awandare; Bediako, Y.; Boakye; Bonney; Bonney, E.; C.M.; D.S.; Diallo; E.B.; G.A.; J.H.; J.K.; J.M.; Kibinge, N.; Kumi-Ansah, F.; L.N.; Magnussen, V.; Mohammed, A.; Morang'a; N.T.; Ndam; Ngoi; O.D.; Odoom; Odoom, T.; Ofori-Boadu, L.; Quansah; Quashie, P.; S.K.; Saiid, S.; Tapela, K.; Tei-Maya, F.; W.K.
EPI_ISL_956332	Department of Biology, University of Basrah	Department of Biology, University of Basrah	A.I.; Abu-Ali; H.F. and Al-Badran
EPI_ISL_540494, EPI_ISL_737344	Department of Clinical Microbiology	GIGA Medical Genomics	Axelle Chaslain; Bouchra Boujemla; Cécile Meex; Céline Fombellida-Lopez; Keith Durkin; Maria Artesi; Marie-Pierre Hayette; Pierrette Melin; Raphaël Boreux; Sébastien Bontems; Vincent Bours
EPI_ISL_507209, EPI_ISL_507211	Department of Experimental Modeling and Pathogenesis of Infectious Diseases	WHO National Influenza Centre Russian Federation	Andrey Komissarov; Anna Ivanova; Artem Fadeev; Daria Danilenko; Mariia Sergeeva
EPI_ISL_1547365, EPI_ISL_1547372, EPI_ISL_1547828, EPI_ISL_1548044	Department of Genetic Engineering and Biotechnology, Shahjalal University of Science and Technology	Genomic Research Lab, BCSIR	Abu Sayeed Mohammad Mahmud; Ajit Ghosh; Barna Goswami; Eshrar Osman; G. M. Nurnabi Azad Jewel; G. M. Nurnabi Azad Jewel; Iffat Jahan; Md. Ahasan Habib; Md. Akkas Ali; Md. Asraful Jahan; Md. Fahmid Hossain Bhuiyan; Md. Hammadul Hoque; Md. Kamrul Islam; Md. Murshed Hasan Sarkar; Md. Nazmul Hasan; Md. Saddam Hossain; Md. Salim Khan; Md. Shamsul Haque Prodhan; Mohammad Mohi Uddin; Mohammad Samir Uzzaman; Shahina Akter; Tanjina Akhter Banu
EPI_ISL_610169, EPI_ISL_610210	Department of Health Technology and Informatics, The Hong Kong Polytechnic University	Department of Health Technology and Informatics, The Hong Kong Polytechnic University	A.K.-L.; A.Y.-M.; B.K.-C.; C.T.-M.; Chan; Chau; D.H.-K.; Fung; G.K.-H.; H.-Y.; Ho; J.S.-L.; K.-T.; K.K.-G.; K.S.-C.; K.S.-S.; L.-K.; Lai; Lao; Lee; Leung; Luk, K.; M.C.-Y.; Ng; Que; S.K.-Y.; S.P.; Shum; Siu; T.-L.; T.T.-L.; Tam; To; W.-K.; W.C.; Wong; Wu; Y.W.-M.; Yam; Yau; Yip
EPI_ISL_1020088, EPI_ISL_1020091, EPI_ISL_1020219, EPI_ISL_1020231, EPI_ISL_1020241, EPI_ISL_1020255, EPI_ISL_1020275, EPI_ISL_1472376			
see above	Department of Health Technology and Informatics, The Hong Kong Polytechnic University	Department of Health Technology and Informatics, The Hong Kong Polytechnic University	A.K.-L.; Alan Ka-Lun Wu; Alex Yat-Man Ho; Barry Kin-Chung Wong; C.T.-M.; Chan; Chloe Toi-Mei Chan; D.S.-H.; David Ho-Keung Shum; Denise Sze-Hang Wong; G.K.-H.; Gilman Kit-Hang Siu; H.-Y.; Hiu-Yin Lao; J.S.-L.; Jake Siu-Lun Leung; K.K.-G.; K.S.-S.; Kam-Tong Yip; Kenneth Siu-Sing Leung; Kingsley King-Gee Tam; Kitty Sau-Chun Fung; Kristine Luk; L.-K.; Lam-Kwong Lee; Lao; Lee; Leung; Lo; M.C.-Y. and Lai; Miranda Chong-Yee Yau; Ng; Sandy Ka-Yee Chau; Shea Ping Yip; Siu; T.T.-L.; Tak-Lun Que; Tam; Timothy Ting-Leung Ng; W.-H.; Wing Cheong Yam; Wing-Kin To; Wong; Wu; Y.W.-M.; Yau; Yvette Wai-Man Lai
EPI_ISL_1168864, EPI_ISL_1425501	Department of Infectious Diseases, Kobe Institute of Health	Department of Infectious Diseases, Kobe Institute of Health	Kentaro Itokawa; Makoto Kuroda; Masanori Hashino; Noriko Nakanishi; Rina Tanaka; Ryohei Nomoto; Tomotada Iwamoto; Tsuyoshi Sekizuka
EPI_ISL_410720	Department of Infectious and Tropical Diseases, Bichat Claude Bernard Hospital, Paris	National Reference Center for Viruses of Respiratory Infections, Institut Pasteur, Paris	Angela Brisebarre; Flora Donati; Marion Barbet; Maud Vanpeene; Mélanie Albert; Méline Bizard; Sylvie Behillil; Sylvie van der Werf; Vincent Enouf; Xavier Lescure.; Yazdan Yazdanpanah
EPI_ISL_479487	Department of Laboratory Medicine Tan Tock Seng Hospital	Department of Laboratory Medicine Tan Tock Seng Hospital	Barkham TMS; Chen YYC; Li C; Maurer-Stroh S; Nagarajan N; Sessions OM; Tang WY; Zair X
EPI_ISL_934584, EPI_ISL_934677, EPI_ISL_1008245, EPI_ISL_1209289, EPI_ISL_1495331, EPI_ISL_1495839	Department of Laboratory Medicine, Division of Clinical Virology, University of Medicine, Vienna	Bergthaler laboratory, CeMM Research Center for Molecular Medicine of the Austrian Academy of Sciences	Andreas Bergthaler; Anna Schedl; Bekir Erguner; Benedikt Agerer; Christoph Bock; Fabian Amman; Jan Laine; Lukas Endler; Maelle Le Moing; Martin Senekowitsch; Michael Schuster; Petr Triska; Thomas Penz
EPI_ISL_447618, EPI_ISL_534336, EPI_ISL_1020316, EPI_ISL_1039160, EPI_ISL_1041958	Department of Laboratory Medicine, National Taiwan University Hospital	Microbial Genomics Core Lab, National Taiwan University Centers of Genomic and Precision Medicine	Chiao-Ling Li; Pei-Jer Chen; Shan-Chwen Chang; Shiou-Hwei Yeh; Sui-Yuan Chang; Ya-Yun Lai; You-Yu Lin
EPI_ISL_648705, EPI_ISL_648734	Department of Laboratory Medicine, Tan Tock Seng Hospital	Department of Laboratory Medicine, Tan Tock Seng Hospital	Barkham TMS; Chen YYC; Li C; Lim JX; Maurer-Stroh S; Nagarajan N; Sessions OM; Tang WY; Zair X
EPI_ISL_501188, EPI_ISL_501220	Department of Medical Microbiology, University Malaya Medical Centre	Department of Medical Microbiology, Faculty of Medicine, University of Malaya	I-Ching SAM; Jennifer Chong; University Malaya Medical Centre COVID Team; Yoke Fun CHAN; Yoong Min CHONG
EPI_ISL_512844	Department of Medical Research	DMR_Myanmar	Aung Kyaw Kyaw; Aung Zaw Latt; Hlaing Myat Thu; Hnin Ohnmar Soe; Htin Lin; Kay Thi Aye; Lai Lai San; Myat Htut Nyunt; Nan Aye Thida Oo; Ni Ni Zaw; Phyu Win Ei; Su Mon Win; Theingi Win Myat; Wah Wah Aung; Yi Yi Kyaw; Zaw Than Htun
EPI_ISL_1027638, EPI_ISL_1027642	Department of Microbiology, National Institute for Public Health of Kosova	Charité Universitätsmedizin Berlin, Institut für Virologie	Barbara Mühlemann; Christian Drosten; Donjeta Hajdari; Julia Schneider; Jörn Beheim-Schwarzbach; Talitha Veith; Terry Jones; Victor M Corman; Xhevat Jakupi; Zana Deva
EPI_ISL_497770, EPI_ISL_497771, EPI_ISL_497823, EPI_ISL_498270, EPI_ISL_498271, EPI_ISL_1197083, EPI_ISL_1197099, EPI_ISL_1197100, EPI_ISL_1197102			
see above	Department of Microbiology, The University of Hong Kong	Department of Microbiology, The University of Hong Kong	Kelvin K.W. To; Kwok-Yung Yuen

EPI_ISL_1008285, EPI_ISL_1008286	Department of Microbiology, University Innsbruck	Bergthaler laboratory, CeMM Research Center for Molecular Medicine of the Austrian Academy of Sciences	Andreas Bergthaler; Anna Schedl; Bekir Erguner; Benedikt Agerer; Christoph Bock; Jan Laine; Lukas Endler; Maelle Le Moing; Martin Senekowitsch; Michael Schuster; Thomas Penz
EPI_ISL_463743, EPI_ISL_1164646, EPI_ISL_1164666, EPI_ISL_1164708, EPI_ISL_1164715, EPI_ISL_1164730, EPI_ISL_1164737, EPI_ISL_1164739, EPI_ISL_1164742, EPI_ISL_1164746			
see above	Department of Molecular Virology, Cyprus Institute of Neurology and Genetics	Department of Molecular Virology, Cyprus Institute of Neurology and Genetics	Anastasis Oulas; Andreas Hadjisavvas; Christina Christodoulou; Christina Tryfonos; Dana Koptides; Denise Alexandrou; George Krashias; George Spyrou; Jan Richter; Maria Loizidou; Mihalis Panayiotidis; Olga Kalakouta; Pavlos Fanis; Stavros Bashiardes
EPI_ISL_1247323	Department of Pathology, University of Cambridge	COVID-19 Genomics UK (COG-UK) Consortium	Aminu S. Jahun; Ian Goodfellow; Iliana Georgana; Martin D. Curran; Myra Hosmillo; Rhys Izuagbe; Surendra Parmar; William L. Hamilton; Yasmin Chaudhry
EPI_ISL_1657121	Department of Public Health Bucharest	National Institute of Infectious Diseases-Prof. Dr. Matei Bals Molecular Diagnostics Laboratory	Andreea Tudor; Corina Casangiu; Dan Otelea; Leontina Banica; Marius Surleac; Ovidiu Vlaicu; Simona Paraschiv
EPI_ISL_1289924	Department of Public Health Microbiology Ljubljana, National Laboratory for Health, Environment and Food	Department of Public Health Microbiology Ljubljana, National Laboratory for Health, Environment and Food	José Gonçalves; Katarina Prosenc; Martin Bosilj; Metka Paragi; Tom Koritnik
EPI_ISL_759899, EPI_ISL_902888, EPI_ISL_995918, EPI_ISL_995954, EPI_ISL_1279096, EPI_ISL_1279140, EPI_ISL_1496936, EPI_ISL_1497111, EPI_ISL_1497127			
see above	Department of Virology and Immunology, University of Helsinki and Helsinki University Hospital, Huslab Finland	Department of Virology, Faculty of Medicine, University of Helsinki, Helsinki, Finland	Essi Korhonen; Hanna Jarva; Hanna Liimatainen; Hannimari Kallio-Kokko; Harri Kangas; Hussein Alburkat; Jenni Virtanen; Maija Lappalainen; Maija Suvanto; Olli Vapalahti; Pekka Ellonen; Phuoc Truong; Ravi Kant; Sari Hannula; Satu Kurkela; Teemu Smura
EPI_ISL_1015320	Department of Virology, Pitié-Salpêtrière hospital	Department of Virology, Pitié-Salpêtrière hospital	Anne-Geneviève Marcelin; Aude Jary; Karen Zafilaza; Stéphane Marot; Valentin Leducq; Vincent Calvez
EPI_ISL_855558, EPI_ISL_855560, EPI_ISL_855561, EPI_ISL_855563, EPI_ISL_855566, EPI_ISL_855567, EPI_ISL_855568, EPI_ISL_855569, EPI_ISL_855570, EPI_ISL_855571, EPI_ISL_855572			
see above	Department of Virology, Principal Military Hospital of Instruction of Tunis	Bundeswehr Institute of Microbiology	Habiba Naija; Kilian Stoecker; Malena Bestehorn-Willmann; Markus H. Antwerpen; Mathias C. Walter; Roman Wölfel & Mohamed Ben Moussa; Simone Eckstein; Susann Handrick
EPI_ISL_757285, EPI_ISL_757286	Department of Virology, Public Health Laboratories Division	Department of Virology, Public Health Laboratories Division	Aamer Ikram; Massab Umair; Muhammad Salman
EPI_ISL_855832, EPI_ISL_1022427, EPI_ISL_1065881, EPI_ISL_1124896	Department of Virus and Microbiological Special Diagnostics, Statens Serum Institut, Copenhagen, Denmark	Aalborg University	Danish Covid-19 Genome Consortium
EPI_ISL_757926, EPI_ISL_759609, EPI_ISL_759696, EPI_ISL_795293, EPI_ISL_795786	Department of Virus and Microbiological Special Diagnostics, Statens Serum Institut, Copenhagen, Denmark	Albertsen Lab, Department of Chemistry and Bioscience, Aalborg University, Denmark	Danish Covid-19 Genome Consortium
EPI_ISL_614460, EPI_ISL_614708, EPI_ISL_614709, EPI_ISL_614786, EPI_ISL_615664, EPI_ISL_618631, EPI_ISL_619378, EPI_ISL_621368			
see above	Department of Virus and Microbiological Special Diagnostics, Statens Serum Institut, Denmark	Albertsen lab, Department of Chemistry and Bioscience, Aalborg University, Denmark	Danish Covid-19 Genome Consortia
EPI_ISL_1647233, EPI_ISL_1647234, EPI_ISL_1647307	Dept. of Microbiology and Infection Control, Akershus University Hospital HF	Dept. of Microbiology and Infection Control, Akershus University Hospital HF	Alexander Hesselberg Løvestad; Hege Vangstein Aamot
EPI_ISL_807154, EPI_ISL_807156	Deva County Emergency Hospital	National Institute of Infectious Diseases-Prof. Dr. Matei Bals Molecular Diagnostics Laboratory	Andreea Tudor; Corina Casangiu; Dan Otelea; Leontina Banica; Marius Surleac; Petre Milu; Simona Paraschiv
EPI_ISL_1533082	Diagnostic and Research Center of Infectious Diseases, Medical Faculty, Andalas University	Diagnostic and Research Center of Infectious Diseases, Medical Faculty, Andalas University	Andani Eka Putra; Ayu Novita Trisnawati; Dede Rahman Agustian; Desmawati; Dessy Arisanty; Fauzul Azhim; Gestina Aliska; Ikhwan R. Sudji; Juane Plantika Menra; Linosefa; Mutia Lailani; Nia Ayuni Putri; Nita Afriani; SM Rezvi; Sekar Asri Tresnaningtyas; Siskalil Fahma; Syafrizayanti; Syandrez Prima Putra; Yolani Syaputri
EPI_ISL_722897	Dipartimento di Scienze Biomediche e Oncologia Umana - Azienda Ospedaliero Universitaria Consorziale Policlinico	Istituto Zooprofilattico Sperimentale della Puglia e della Basilicata	Bianco A.; Capozzi L.; Chironna M.; Del Sambro L.; Loconsole D.; Parisi A.
EPI_ISL_1469669	Diretoria de Vigilância em Saúde	Epiclin	Ana Paula Muterle; Carolina Comerlato; Eliana Márcia Da Ros Wendland; Fernando Hayashi Sant'Anna; Janira Prichula; Juliana Comerlato
EPI_ISL_760074, EPI_ISL_955957, EPI_ISL_959535, EPI_ISL_994772, EPI_ISL_995244, EPI_ISL_995257, EPI_ISL_1063722, EPI_ISL_1063739, EPI_ISL_1138923, EPI_ISL_1138935, EPI_ISL_1165050, EPI_ISL_1252372, EPI_ISL_1252397, EPI_ISL_1252415, EPI_ISL_1319025, EPI_ISL_1489987, EPI_ISL_1490083, EPI_ISL_1587542, EPI_ISL_1587547, EPI_ISL_1587549			
see above	Division of Emerging Infectious Diseases, Bureau of Infectious Diseases Diagnosis Control, Korea Disease Control and Prevention Agency	Division of Emerging Infectious Diseases, Bureau of Infectious Diseases Diagnosis Control, Korea Disease Control and Prevention Agency	Ae Kyung Park; Chae Young Lee; Chaeyoung Lee; Eun-Jin Kim; Heui Man Kim; Il-Hwan Kim; Jeong-Ah Kim; Jeong-Ah Kimg; Jeong-Min Kim; Jin Sun No; Namjoo Lee; Sang Hee Woo
EPI_ISL_471455, EPI_ISL_498005, EPI_ISL_498017, EPI_ISL_498023, EPI_ISL_506984, EPI_ISL_510568, EPI_ISL_526724			
see above	Division of Viral Diseases, Center for Laboratory Control of Infectious Diseases, Korea Centers for Diseases Control and Prevention	Division of Viral Diseases, Center for Laboratory Control of Infectious Diseases, Korea Centers for Diseases Control and Prevention	Daesang Lee; Dong Hyun Song; Heui Man Kim; Hye-Jun Jo; Jeong-Min Kim; Jun-Sub Kim; Myung Guk Han; Namjoo Lee; Sang Hee Woo; Seong Tae Jeong; Yoon-Seok Chung
EPI_ISL_700513, EPI_ISL_700517, EPI_ISL_700547, EPI_ISL_700567, EPI_ISL_700568, EPI_ISL_700570, EPI_ISL_700582, EPI_ISL_700591, EPI_ISL_700592			
see above	Dr Abdurahman CDC wc DAC	NHLS/UCT	Arash Iranzadeh; Bruna Galvao; Carolyn Williamson; Deelan Doolabh; Diana Hardie; Innocent Mudau; Kruger Marais; Lynn Tyers; Marvin Hsiao; Stephen Korsman
EPI_ISL_960160	Dr Abdurahman CDC wc DAC	National Health Laboratory Service/UCT	Arash Iranzadeh; Bruna Galvao; Carolyn Williamson; Deelan Doolabh; Diana Hardie; Innocent Mudau; Kruger Marais; Lynn Tyers; Marvin Hsiao; Stephen Korsman
EPI_ISL_745154	Dr Ivan Toms Clinic wc IVT	National Health Laboratory Service (NHLS), Tygerberg	Bronwyn Kleinhans; Eduan Wilkindon; Gert van Zyl; Houriiyah Tegally; Kayla Delaney; Susan Engelbrecht; Tulio de Oliveira; Wolfgang Preiser
EPI_ISL_418811	Dr. Georges-L.-Dumont University Hospital Centre	National Microbiology Laboratory	Anna Majer; Grace Seo; Guillaume Desnoyers; Matthew Gilmour; Morag Graham; Natalie Knox; Nathalie Bastien; Philip Mabon; Richard Garceau; Shari Tyson; Timothy Booth; Yan Li
EPI_ISL_1055385, EPI_ISL_1055412, EPI_ISL_1055450	Dr. Leonard A. Miller Centre for Health Services	National Microbiology Laboratory (NML)	Adel Malek; Anna Majer; Anneliese Landgraff; CanCOGeN's metadata curation team; Darian Hole; Elsie Grudeski; Gary Van Domselaar; George Zahariadis; Grace Seo; Jennifer Tanner; Kerri Smith; Kirsten Biggar; Laura Gilbert; Madison Chapel; Morag Graham; Natalie Knox; Nathalie Bastien; Philip Mabon; Public Health Agency of Canada CanCOGeN team; Rhiannon Huzarewich; Robert Needle; Russell Mandes; Shari Tyson; Timothy Booth; Yan Li; Yang Yu
EPI_ISL_417211, EPI_ISL_417212	Dunedin Hospital	University of Otago	B. Lawley; J. Grant; J. Ussher; M.E. Quiñones-Mateu; R. Harfoot
EPI_ISL_414467, EPI_ISL_523336, EPI_ISL_523473, EPI_ISL_523784, EPI_ISL_578044, EPI_ISL_801433, EPI_ISL_904514			
see above	Dutch COVID-19 response team	Erasmus Medical Center	Anne van der Linden; Annemiek van der Eijk; Aura Timen; Bas Oude Munnink; Claudia Schapendonk; Corien Swaan; Corine GeurtsvanKessel; David Nieuwenhuijse; Emmanuelle Munger; Irina Chestakova; Jeroen van Kampen; Jolanda Voermans; Madelief Mollers; Manon Haverkate; Marion Koopmans; Marjan Boter; Mark Pronk; Mart Stein; Pascal Lexmond; Reina Sikkema; Richard Molenkamp; Sandra Kengne Kamga Mobou; Stefan van Nieuwkoop; Theo Bestebroer; on behalf of the Dutch national COVID-19 response team.
EPI_ISL_454754, EPI_ISL_547452, EPI_ISL_636493, EPI_ISL_636515, EPI_ISL_636516, EPI_ISL_905477, EPI_ISL_943231, EPI_ISL_1013811, EPI_ISL_1014269, EPI_ISL_1014550, EPI_ISL_1014552, EPI_ISL_1014558, EPI_ISL_1014561, EPI_ISL_1014571, EPI_ISL_1014572, EPI_ISL_1014580, EPI_ISL_1014585, EPI_ISL_1014603, EPI_ISL_1014614, EPI_ISL_1014617, EPI_ISL_1014619, EPI_ISL_1014630, EPI_ISL_1014655, EPI_ISL_1035770, EPI_ISL_1089895, EPI_ISL_1089992, EPI_ISL_1165484, EPI_ISL_1165525, EPI_ISL_1165532, EPI_ISL_1165538, EPI_ISL_1165618, EPI_ISL_1232257, EPI_ISL_1232258, EPI_ISL_1232293, EPI_ISL_1232307, EPI_ISL_1232327, EPI_ISL_1232540, EPI_ISL_1232552, EPI_ISL_1232736, EPI_ISL_1232749, EPI_ISL_1233007, EPI_ISL_1288143, EPI_ISL_1288147, EPI_ISL_1289400, EPI_ISL_1370629, EPI_ISL_1370674, EPI_ISL_1371327, EPI_ISL_1456283, EPI_ISL_1521080, EPI_ISL_1521087, EPI_ISL_1521169, EPI_ISL_1521321, EPI_ISL_1521546, EPI_ISL_1522178, EPI_ISL_1522181, EPI_ISL_1522183, EPI_ISL_1522185, EPI_ISL_1522192, EPI_ISL_1522199, EPI_ISL_1522200, EPI_ISL_1522208, EPI_ISL_1522211, EPI_ISL_1522214, EPI_ISL_1522215, EPI_ISL_1522216, EPI_ISL_1522223, EPI_ISL_1596135, EPI_ISL_1596137, EPI_ISL_1596148, EPI_ISL_1596230, EPI_ISL_1596270, EPI_ISL_1596435, EPI_ISL_1596766, EPI_ISL_1597327, EPI_ISL_1597407			
see above	Dutch COVID-19 response team	National Institute for Public Health and the Environment (RIVM)	Adam Meijer; AnneMarie van den Brandt; Annelies Kroneman; Bas van der Veer; Chantal Reusken; Dennis Schmitz; Dirk Eggink; Euníce Then; Florian Zwagemaker; Harry Vennema; James Groot; Jeroen Cremer; Jolienke Hardeman; Karim Hajji; Kim Freriks; Linda van de Nes; Lisa Wijsman; Lynn Aarts; Melissa van Tuil; Pieter Overduin; Robert Kohl; Ryanne Jaarsma; Sanne Bos; Sharon van den Brink; Sjoerd Kuiling; on behalf of the national COVID-19 response team
EPI_ISL_640024	Dysselsdorp Clinic wc DDC	NHLS/UCT	Arash Iranzadeh; Bruna Galvao; Carolyn Williamson; Deelan Doolabh; Diana Hardie; Innocent Mudau; Kruger Marais; Lynn Tyers; Marvin Hsiao; Stephen Korsman
EPI_ISL_534212, EPI_ISL_639660	E. Gulbja Laboratorija	Latvian Biomedical Research and Study Centre	Dmitrijs Perminovs; Ivars Silamiķelis; Jānis Kloviņš; Jānis Pjalkovskis; Kaspars Megnis; Mikus Gavars; Monta Ustinova; Uga Dumpis; Vita Rovīte; Ņikita Zrelovs
EPI_ISL_961873, EPI_ISL_1219668, EPI_ISL_1312455, EPI_ISL_1321920	E. Gulbja laboratorija	Latvian Biomedical Research and Study Centre	Davids Fridmanis; Davids Fridmanis; Dmitrijs Perminovs; Dmitrijs Perminovs; Elina Dimina; Guntars Zarins; Ivars Silamikelis; Ivars Silamikelis; Janis Klovins; Janis Pjalkovskis; Janis Klovins; Jurijs Perevoscikovs; Kaspars Megnis; Kaspars Megnis; Laila Silamikele; Laila Silamikele; Lauma Freimane; Lauma Freimane; Laura Ansone; Laura Ansone; Liga Birzniece; Liga Birzniece; Mikus Gavars; Mikus Gavars; Monta Ustinova; Monta Ustinova; Nikita Zrelovs; Nikita Zrelovs; Uga Dumpis; Uga Dumpis; Una Krumina; Vita Rovite; Vita Rovite
EPI_ISL_745173, EPI_ISL_745175, EPI_ISL_745176, EPI_ISL_745177, EPI_ISL_745178	Eerste River Hospital wc ERH	National Health Laboratory Service (NHLS), Tygerberg	Bronwyn Kleinhans; Eduan Wilkindon; Gert van Zyl; Houriiyah Tegally; Kayla Delaney; Susan Engelbrecht; Tulio de Oliveira; Wolfgang Preiser
EPI_ISL_468044, EPI_ISL_468046, EPI_ISL_468047, EPI_ISL_468048, EPI_ISL_468050, EPI_ISL_468051, EPI_ISL_468052, EPI_ISL_468055, EPI_ISL_468056, EPI_ISL_468059, EPI_ISL_475722, EPI_ISL_475723, EPI_ISL_475724, EPI_ISL_477161, EPI_ISL_478672, EPI_ISL_479686, EPI_ISL_479691, EPI_ISL_479696, EPI_ISL_479699, EPI_ISL_479700, EPI_ISL_479701, EPI_ISL_479702, EPI_ISL_479705, EPI_ISL_479706, EPI_ISL_479707, EPI_ISL_479709, EPI_ISL_479711, EPI_ISL_479713, EPI_ISL_479714, EPI_ISL_479715, EPI_ISL_479718, EPI_ISL_479719, EPI_ISL_479720, EPI_ISL_479723, EPI_ISL_479724, EPI_ISL_479725, EPI_ISL_479727, EPI_ISL_479728, EPI_ISL_479729, EPI_ISL_479731, EPI_ISL_479732, EPI_ISL_479733, EPI_ISL_479734, EPI_ISL_529141, EPI_ISL_529142, EPI_ISL_529145			
see above	Egyptian National Cancer Institute (ENCI)	Egyptian National Cancer Institute (ENCI)	A.A.; A.N.; Abdel Rahman N; Abdel Rahman N.; Abdelhamid, W.; Abouelhoda; Abouelhoda, M.; Ahmed; Ali; Ali, M.; Amer; Bahnassy; Elkhateeb; Elsissy; Ezzelarab; Gad, A.; H.K.; Hafez; Hamdy; Hassan; Hassan, W.; K.E.; Khattab; M.A.; M.H.; M.M.; M.S.; Mahmoud; Mohamed; O.S.; Raouf, A.; S.M.; Samir, M.; Soliman; W.A.; Zekri
EPI_ISL_979481, EPI_ISL_1404994	Eurofins Diatherix	Hudsonalpha Genome Sequencing Center	Ada Stewart; Jane Grimwood; Jenell Webber; Jennifer Cart; John Lovell; Joshua Stough; Leslie Malone; Lori H. Handley; Melissa Williams; Stefan Brzezinski; Teresa Jones; and Jeremy Schmutz
EPI_ISL_1571490	Eurofins MVZ Labor Gelsenkirchen	Robert Koch Institute	
EPI_ISL_1220060	FUNDACION VALLE DEL LILI	Instituto Nacional de Salud- Dirección de Investigación en Salud Pública	Carlos Franco-Muñoz; Diego A. Álvarez-Díaz; Diego Andrés Prada; Gerardo Santamaría; Hector Alejandro Ruiz-Moreno; Jhonnatan Reales-González; Julian Naizaque; Katherine Laiton-Donato; Magdalena Wiesner; Marcela Mercado-Reyes.; Maria T. Herrera-Sepúlveda; Martha Lucia Ospina Martínez; Sheryll Corchuelo
EPI_ISL_640121, EPI_ISL_1040682,	False Bay Hospital wc FBH	NHLS/UCT	Arash Iranzadeh; Bruna Galvao; Carolyn Williamson; Deelan Doolabh; Diana Hardie; Innocent Mudau; Kruger Marais; Lynn Tyers; Marvin Hsiao; Stephen Korsman

EPI_ISL_1040775, EPI_ISL_1040779, EPI_ISL_1040780			
EPI_ISL_1239940	Florida Bureau of Public Health Laboratories	Florida Bureau of Public Health Laboratories	Jason Blanton; Sarah Schmedes
EPI_ISL_476139	Folkhalsomyndigheten	The Public Health Agency of Sweden	Anna Risberg; Anna-Malin Linde; Karin Tegmark-Wisell; Maria Lind Karlberg; Mattias Haukland; Olov Svartstrom; Oskar Karlsson Lindsjo; Petra Edquist; Reza Advani; Sandra Broddesson; Shamam Muradrasoli
EPI_ISL_581490, EPI_ISL_581491	Fondation Congolaise pour la recherche medicale (FCRM)	NGS Competence Center Tübingen, Institut für Medizinische Mikrobiologie und Hygiene, Universitätsklinikum Tübingen	Angel Angelov
EPI_ISL_1654213, EPI_ISL_1654215, EPI_ISL_1654216, EPI_ISL_1654217	Fondation Congolaise pour la recherche medicale (FCRM), Francine Ntoumi	Institute of Tropical Medicine	Prof. Francine Ntoumi and Prof. Dr. Thirumalaisamy P. Velavan
EPI_ISL_912353, EPI_ISL_912356, EPI_ISL_912357, EPI_ISL_912362, EPI_ISL_912366, EPI_ISL_912372, EPI_ISL_912387, EPI_ISL_912388, EPI_ISL_912389			
see above	Fondation Congolaise pour la recherche medicale (FCRM), Francine Ntoumi	NGS Competence Center Tuebingen, Institut für Medizinische Mikrobiologie und Hygiene, Universitaetsklinikum Tübingen	Angel Angelov
EPI_ISL_491481	Functional Genomics Core University of South Carolina / Prisma Health–Midlands	Functional Genomics Core, Center For Targeted Therapeutics,	Alyssa Clay-Glimour; B.Celia Cui; Diego Altomare; Hao Ji; Helmut Albrecht; Mengqian Chen; Michael Shtutman; Michael Wyatt; Phillip Buckhaults
EPI_ISL_1182586	Fundação Ezequiel Dias (FUNED)	Coordenação Geral de Laboratórios de Saúde Pública (CGLAB/DAEVS/SVS/MS)	Vagner Fonseca; et al.
EPI_ISL_493375	Furst Medical Laboratory	Norwegian Institute of Public Health, Department of Virology	Hilde Elshaug; Kamilla Heddeland Instefjord; Karoline Bragstad; Kathrine Stene-Johansen; Olav Hungnes; Rasmus Riis Kopperud
EPI_ISL_522549, EPI_ISL_522550	Félix Guyon Hospital	UMR PIMIT Université de La Réunion	Camille Lebarbenchon; David Wilkinson; Patrick Mavingui
EPI_ISL_1262479	GA Department of Public Health	GA Department of Public Health	Aliyah Fields; Cynthia Dixey; Jonathan Edwards; Stacy Reeves; Taylor Smith; Tonia Parrott
EPI_ISL_1265820	GHE REUNION	CNR Virus des Infections Respiratoires - France SUD	Antonin Bal; Bruno Lina; Gregory Destras; Gwendolyne Burfin; Hadrien Regue; Laurence Josset; Martine Valette; Quentin Semanas
EPI_ISL_1628845, EPI_ISL_1628915, EPI_ISL_1628942, EPI_ISL_1629017, EPI_ISL_1629079, EPI_ISL_1629083, EPI_ISL_1629085, EPI_ISL_1629086, EPI_ISL_1629089, EPI_ISL_1629090, EPI_ISL_1629095, EPI_ISL_1629097, EPI_ISL_1629108, EPI_ISL_1629117, EPI_ISL_1629121, EPI_ISL_1629165, EPI_ISL_1629169, EPI_ISL_1629178, EPI_ISL_1629180			
see above	GHER	UMR PIMIT	Dr Camille Lebarbenchon; Dr David A Wilkinson; Dr Patrick Mavingui; Magali Turpin
EPI_ISL_825049, EPI_ISL_825054	GMERS Medical College and Hospital, Gotri, Vadodara	Gujarat Biotechnology Research Centre	Afzal Ansari; Apurvasinh Puvar; Bithika Duttaroy; Chaitanya Joshi; Dinesh Kumar; Janvi Raval; Kalpesh Mistry; Labdhi Pandya; Madhvi Joshi; Mitesh Kamothi; Nikha Trivedi; Nitin Savaliya; Ramesh Pandit; Zarna Patel; Zuber Saiyed
EPI_ISL_730568	Gazi University Faculty of Medicine, Medical Virology Laboratory	Gazi University Faculty of Medicine, Medical Virology Laboratory	Erdem Şahin; Gülendam Bozdayı; Hager Muftah; Işıl Fidan; Kayhan Çağlar; Murat Dizbay; Selin Yiğit; Shaknoza Sarzhanova; Özlem Güzel Tunçcan
EPI_ISL_1626610, EPI_ISL_1626611, EPI_ISL_1626613, EPI_ISL_1626616	Gencore - Universidad de los Andes	Gencore - Universidad de los Andes	Ana Maria Palacio; Cristian Barrera; David Gonzalez; Erica Salguero; Gabriela Ariza; Luisa Sacristan; Marcela Guevara; Silvia Restrepo
EPI_ISL_1184582	Genelabs Medical (Pvt) Ltd	Genelabs Medical (Pvt) Ltd	Chandanamali Punchihewa; K.Dasuki Hansana Perera; Ravithra Roshan Rajasegar; Tharmini Sundralingam; Viveka Selvarasa
EPI_ISL_959650	General Hospital "Abdulah Nakas" Sarajevo	Alea Genetic Centre	Dino Pecar; Lana Salihefendic; Rijad Konjhodzic; Sead Jazic
EPI_ISL_944786	General Hospital - Prilep	Research Center for Genetic Engineering and Biotechnology "Georgi D. Efremov" , Macedonian Academy of Sciences and Arts	Aleksandar J. Dimovski; Dijana Plasheska-Karanfilska; Gjorgji Bozinovski; Milena Jakimovska; Predrag Noveski
EPI_ISL_1014933	General Hospital - Strumica	Research Center for Genetic Engineering and Biotechnology "Georgi D. Efremov" , Macedonian Academy of Sciences and Arts	Aleksandar J. Dimovski; Dijana Plasheska-Karanfilska; Gjorgji Bozinovski; Milena Jakimovska; Predrag Noveski
EPI_ISL_943997, EPI_ISL_1142968	General Hospital - Tetovo	Research Center for Genetic Engineering and Biotechnology "Georgi D. Efremov" , Macedonian Academy of Sciences and Arts	Aleksandar J. Dimovski; Dijana Plasheska-Karanfilska; Gjorgji Bozinovski; Milena Jakimovska; Predrag Noveski
EPI_ISL_406798	General Hospital of Central Theater Command of People's Liberation Army of China	BGI & Institute of Microbiology, Chinese Academy of Sciences & Shandong First Medical University & Shandong Academy of Medical Sciences & General Hospital of Central Theater Command of People's Liberation Army of China	Weifeng Shi and Zhenhong Hu; Weijun Chen; Yuhai Bi
EPI_ISL_746518, EPI_ISL_746595, EPI_ISL_746691, EPI_ISL_746742, EPI_ISL_746770, EPI_ISL_754393, EPI_ISL_1167668, EPI_ISL_1167679, EPI_ISL_1167706, EPI_ISL_1167721, EPI_ISL_1167732, EPI_ISL_1167759, EPI_ISL_1167768, EPI_ISL_1167775, EPI_ISL_1167796, EPI_ISL_1167845, EPI_ISL_1167886, EPI_ISL_1167907, EPI_ISL_1300461, EPI_ISL_1300463, EPI_ISL_1300481, EPI_ISL_1300491, EPI_ISL_1300519, EPI_ISL_1321460, EPI_ISL_1321470, EPI_ISL_1321506, EPI_ISL_1321509, EPI_ISL_1321530, EPI_ISL_1321574, EPI_ISL_1470426, EPI_ISL_1470432, EPI_ISL_1470451, EPI_ISL_1470503, EPI_ISL_1534553, EPI_ISL_1633475			
see above	Genetica Molecular and Subdepartamento de Virologia ISP Chile	Instituto de Salud Publica de Chile	Andres Castillo; Barbara Parra; Gisselle Barra; Jaime Lagos; Javier Tognarelli; Jorge Fernandez; Karen Orostica; Loredana Arata; Patricia Bustos; Rodrigo Fasce
EPI_ISL_1578116, EPI_ISL_1595849	Genome Analysis Center, Yamanashi Central Hospital	Genome Analysis Center, Yamanashi Central Hospital	Yosuke Hirotsu
EPI_ISL_833143	Genomic Laboratory (GLAB) (Conjoint lab of Health Directorate of Istanbul and Istanbul Technical University)	Genomic Laboratory (GLAB), Istanbul Technical University	Gizem Dinler Doganay; Ilker Karacan; Levent Doganay; Nisan Denizce Can; Pari Sharifli; Payam Zolfagharian; Tugba Kizilboga Akgun
EPI_ISL_632908	Genomic Sciences, Rehman Medical Institute	Genomic Sciences, Rehman Medical Institute	Afridi; Ali, J.; H. and Jehanzeb, V.; Haider; Jan; S.A.; Sabiha, B.; U.K.
EPI_ISL_1366334	Genomica Lab Molecular, Mexico	Andersen lab at Scripps Research	Jose Horacio Reyna Verdugo; Jose Roman Chavez Mendez; Luis Alberto Rangel Gonzalez; Martin Gonzalez Ibarra; SEARCH Alliance San Diego with Jonathan Gonzalez Garcia
EPI_ISL_812783, EPI_ISL_812784, EPI_ISL_812785, EPI_ISL_812786, EPI_ISL_812787, EPI_ISL_812788, EPI_ISL_812789, EPI_ISL_812790, EPI_ISL_812791, EPI_ISL_812792, EPI_ISL_812793, EPI_ISL_812794, EPI_ISL_812795, EPI_ISL_812796, EPI_ISL_812797, EPI_ISL_812798, EPI_ISL_812799, EPI_ISL_812800, EPI_ISL_812801, EPI_ISL_812802, EPI_ISL_812803, EPI_ISL_812804, EPI_ISL_812805, EPI_ISL_812806, EPI_ISL_812807, EPI_ISL_812808, EPI_ISL_812809, EPI_ISL_812810, EPI_ISL_812811, EPI_ISL_812812, EPI_ISL_812813, EPI_ISL_812814, EPI_ISL_812816, EPI_ISL_812817, EPI_ISL_812818, EPI_ISL_812819, EPI_ISL_812820, EPI_ISL_812822, EPI_ISL_812823, EPI_ISL_812825, EPI_ISL_812826, EPI_ISL_812831, EPI_ISL_812833, EPI_ISL_812834, EPI_ISL_812835, EPI_ISL_812836, EPI_ISL_812837, EPI_ISL_812838, EPI_ISL_812839, EPI_ISL_812840, EPI_ISL_812841, EPI_ISL_812842, EPI_ISL_812843, EPI_ISL_812844, EPI_ISL_812845, EPI_ISL_812846, EPI_ISL_812847, EPI_ISL_812849, EPI_ISL_812850, EPI_ISL_812851, EPI_ISL_812852, EPI_ISL_812853, EPI_ISL_812854, EPI_ISL_812855, EPI_ISL_812856, EPI_ISL_812858, EPI_ISL_812859, EPI_ISL_812860, EPI_ISL_812861, EPI_ISL_812863, EPI_ISL_812864, EPI_ISL_812865, EPI_ISL_812867, EPI_ISL_812868, EPI_ISL_812869, EPI_ISL_812870, EPI_ISL_812871, EPI_ISL_812872			
see above	Genomics Program, Children Cancer Hospital	Genomics Program, Children Cancer Hospital	Abdo, I.; Abouelnaga, S.; Amer, K.; Bakry, U.; Diab, A.; El-Shaqnqery, H.; El-Zayat, M.; Farawyla, H.; Gomaa, C.; Hadad, A.; Halafawy, A.; Hammad, M.; Hassan, R.; Hassan, W.; Hatem, A.; Hussein, S.; Jalal, D.; Magdeldin, S.; Mansour, T.; Monuir, G.; Salah, H.; Samir, O.; Sayed, A.; Soliman, M.; Soliman, S.; shalaby, L.
EPI_ISL_640016, EPI_ISL_700497	George Hospital wc GRH	NHLS/UCT	Arash Iranzadeh; Bruna Galvao; Carolyn Williamson; Deelan Doolabh; Diana Hardie; Innocent Mudau; Kruger Marais; Lynn Tyers; Marvin Hsiao; Stephen Korsman
EPI_ISL_960118	George Hospital wc GRH	National Health Laboratory Service/UCT	Arash Iranzadeh; Bruna Galvao; Carolyn Williamson; Deelan Doolabh; Diana Hardie; Innocent Mudau; Kruger Marais; Lynn Tyers; Marvin Hsiao; Stephen Korsman
EPI_ISL_640049	George Road Sat Clinic wc GWM	NHLS/UCT	Arash Iranzadeh; Bruna Galvao; Carolyn Williamson; Deelan Doolabh; Diana Hardie; Innocent Mudau; Kruger Marais; Lynn Tyers; Marvin Hsiao; Stephen Korsman
EPI_ISL_960696	Germano de sousa	Instituto Gulbenkian de Ciencia	Cathy Paulino; João Costa; João Sobral; Maria Costa; Ricardo Leite; Susana Ladeiro
EPI_ISL_774976	Gonoshasthya-RNA Molecular Diagnostic and Research Center	Gonoshasthya-RNA Molecular Diagnostic and Research Center	Firoz Ahmed; Maha Jamiruddin; Mahfuza Marzan; Md. Ahsanul Haq; Mohd. Raeed Jamiruddin; Mohib Ullah Khondoker; Mousumi Chaity; Mumtarin Jannat Oishee; Nafisa Azmuda; Nihad Adnan; Nowshin Jahan; Salma Akter; Sayeda Moriam Liza; Shahad Saif Khandker; Shahana Sharmin; Tamanna Ali; Taslin Jahan Mou
EPI_ISL_1225434, EPI_ISL_1225519, EPI_ISL_1225544, EPI_ISL_1225545, EPI_ISL_1502934, EPI_ISL_1502937, EPI_ISL_1502943, EPI_ISL_1502998			
see above	Gorgas Memorial Laboratory of Health Studies	Gorgas Memorial Laboratory of Health Studies	Adriana Weeden; Alejandra Valoy; Alexander Martinez; Ambar Moreno; Anyuri Ortiz; Brechla Moreno; Castillo Jorge; Claudia Gonzalez; Daniel Castillo; Danilo Franco; Davis Beltran; Dimelza Arauz; Elimelec Valdespino; Franco Danilo; Gonzalez Claudia; Gretel Vasquez; Ilka Guerra; Isela Guerrero; Jessica Gondola; Jim Chang; Juan Miguel Pascale; Layda Abrego; Leyda Abrego; Lisseth Saenz; Lopez-Verges Sandra; Mabel Martinez-Montero; Maria Chen-German; Marlene Castillo; Marlenne Castillo; Martinez Alexander; Melissa Gaitan; Moreno Ambar; Moreno Brechla; Oris Chavarria; Ortiz Alma; Rita Corrales; Rita Rodriguez; Sandra Lopez-Verges; Yamilka Diaz; Yaneth Pitti; Zumara Chaverra
EPI_ISL_700543	Great Brak River Clinic wc GBC	NHLS/UCT	Arash Iranzadeh; Bruna Galvao; Carolyn Williamson; Deelan Doolabh; Diana Hardie; Innocent Mudau; Kruger Marais; Lynn Tyers; Marvin Hsiao; Stephen Korsman
EPI_ISL_960149	Great Brak River Clinic wc GBC	National Health Laboratory Service/UCT	Arash Iranzadeh; Bruna Galvao; Carolyn Williamson; Deelan Doolabh; Diana Hardie; Innocent Mudau; Kruger Marais; Lynn Tyers; Marvin Hsiao; Stephen Korsman
EPI_ISL_794818, EPI_ISL_794820	Greek Genome Center, Biomedical Research Foundation of the Academy of Athens (BRFAA)	Greek Genome Center, Biomedical Research Foundation of the Academy of Athens (BRFAA)	Christina Maria Kravvari; Dimitrios Thanos; Emmanouil Athanasiadis; Ioannis Vatsellas; Katerina Zoi; Thodoris Loupis
EPI_ISL_640040, EPI_ISL_640112, EPI_ISL_640129, EPI_ISL_700493, EPI_ISL_700507, EPI_ISL_700534, EPI_ISL_700577, EPI_ISL_1040648, EPI_ISL_1040706, EPI_ISL_1040747, EPI_ISL_1040750, EPI_ISL_1040751, EPI_ISL_1040753, EPI_ISL_1040756, EPI_ISL_1040781, EPI_ISL_1040782, EPI_ISL_1040785, EPI_ISL_1040786, EPI_ISL_1040789, EPI_ISL_1040795, EPI_ISL_1040800, EPI_ISL_1040801, EPI_ISL_1040802, EPI_ISL_1040807, EPI_ISL_1040808, EPI_ISL_1040811, EPI_ISL_1040812, EPI_ISL_1040813, EPI_ISL_1040815, EPI_ISL_1040816, EPI_ISL_1040818, EPI_ISL_1040822			
see above	Groote Schuur Hospital wc GSH	NHLS/UCT	Arash Iranzadeh; Bruna Galvao; Carolyn Williamson; Deelan Doolabh; Diana Hardie; Innocent Mudau; Kruger Marais; Lynn Tyers; Marvin Hsiao; Stephen Korsman
EPI_ISL_960117, EPI_ISL_960126, EPI_ISL_960132, EPI_ISL_960135	Groote Schuur Hospital wc GSH	National Health Laboratory Service/UCT	Arash Iranzadeh; Bruna Galvao; Carolyn Williamson; Deelan Doolabh; Diana Hardie; Innocent Mudau; Kruger Marais; Lynn Tyers; Marvin Hsiao; Stephen Korsman
EPI_ISL_699065	Group 42 (G42) Healthcare, Abu Dhabi, United Arab Emirates; Department of Health, The United Arab Emirates	G42 Healthcare	Ashish Koshy; Budoor Alqarni; Denghui Liu; Fang Chen; Hanif Khalak; Huanming Yang; Javier Quilez; Jian Wang; Junhua Li; Ke Liang; Long Lin; Mohammed Saifuddin Fasihuddin; Nan Qiao; Nawal Ahmed Mohamed Al Kaabi; Pauline Ogrodzki; Pei Wu; Peng Xiao; Pengjuan Liu; Rong Liu; Sally Mahmoud; Siyang Liu; Stephen S. Francis; Tao Ma; Vinay Kusuma; Walid Abbas Zaher; Weibin Liu; Wenjun He; Xavier Anton; Xin Jin; Xin Meng; Xinyu Huang; Xun Xu; Zhaorong Yuan
EPI_ISL_1273050, EPI_ISL_1273051, EPI_ISL_1273053, EPI_ISL_1273059, EPI_ISL_1273061, EPI_ISL_1273062, EPI_ISL_1273063, EPI_ISL_1273066, EPI_ISL_1273069, EPI_ISL_1273073			
see above	Guam Public Health Laboratory	Centers for Disease Control and Prevention Division of Viral Diseases, Pathogen Discovery	Anna Montmayeur; Anna Uehara; Ben L. Rambo-Martin; Clinton R. Paden; Dhwani Batra; Haibin Wang; Jasmine Padilla; Jing Zhang; Justin Lee; Katie Dillon; Krista Queen; Kristen Knipe; Kristine Lacek; Lori Rowe; Mark Burroughs; Matthew Schmerer; Mili Sheth; Peter W. Cook; Rachel Marine; Sam Shepard; Sarah Nobles; Shoshona Le; Suxiang Tong; Yan Li; Ying Tao
EPI_ISL_700481, EPI_ISL_700491,	Guguletu CHC wc GDH	NHLS/UCT	Arash Iranzadeh; Bruna Galvao; Carolyn Williamson; Deelan Doolabh; Diana Hardie; Innocent Mudau; Kruger Marais; Lynn Tyers; Marvin Hsiao; Stephen Korsman

EPI_ISL_700530, EPI_ISL_1040805			
EPI_ISL_960155	Guguletu CHC wc GDH	National Health Laboratory Service/UCT	Arash Iranzadeh; Bruna Galvao; Carolyn Williamson; Deelan Doolabh; Diana Hardie; Innocent Mudau; Kruger Marais; Lynn Tyers; Marvin Hsiao; Stephen Korsman
EPI_ISL_1239453	Gundersen Molecular Diagnostics Laboratory	Kabara Cancer Research Institute	Craig S. Richmond; Paraic A. Kenny
EPI_ISL_1116988, EPI_ISL_1117339	H Agostinho Ribeiro - Felgueiras	Instituto Nacional de Saude (INSA) and Instituto Gulbenkian de Ciencia (IGC)	Borges et al
EPI_ISL_765219	H Beatriz Angelo	Instituto Nacional de Saude (INSA)	Borges et al
EPI_ISL_941384, EPI_ISL_941395	H Divino Espirito Santo - Ponta Delgada	Instituto Nacional de Saude (INSA)	Borges et al
EPI_ISL_873032, EPI_ISL_1372379, EPI_ISL_1372386, EPI_ISL_1491694, EPI_ISL_1491724	HELIX LLC	WHO National Influenza Centre Russian Federation	Alexey Masharsky; Andrey Komissarov; Anna Ivanova; Artem Fadeev; Daria Danilenko; Dmitry Bazhenov; Dmitry Lioznov; Elena Nabieva; Georgii Bazykin; Ksenia Safina; Kseniya Komissarova; Maria Baturova; Maria Pisareva; Maria Timofeeva; Mikhail Bakaev; Tamila Musaeva; Veronika Eder
EPI_ISL_1583279	HG Pharma GmbH	Bergthaler laboratory, CeMM Research Center for Molecular Medicine of the Austrian Academy of Sciences	Andreas Bergthaler; Anna Schedl; Bekir Erguner; Benedikt Agerer; Christoph Bock; Fabian Amman; Jan Laine; Lukas Endler; Maelle Le Moing; Martin Senekowitsch; Michael Schuster; Petr Triska; Thomas Penz
EPI_ISL_955253	HGSMF 26 CABO SAN LUCAS	BIOBANCO / COCTI	Arias C; Borja-Aburto VH; Grajales-Muñiz C; Grande R; Isa P; López S; Muñoz-Medina JE; Ochoa Carrera LA; Rojas-Mendoza T; Santacruz Tinoco CE; Sánchez A; Taboada B
EPI_ISL_903583	HI Dept. of Health, State Laboratories Division	Genomics and Discovery, Respiratory Viruses Branch, Division of Viral Diseases, Centers for Disease Control and Prevention	Anna Montmayeur; Anna Uehara; Ben L. Rambo-Martin; Clinton R. Paden; Dhwani Batra; Haibin Wang; Jasmine Padilla; Jing Zhang; Justin Lee; Krista Queen; Lori Rowe; Mark Burroughs; Mili Sheth; Peter W. Cook; Rachel Marine; Sarah Nobles; Suxiang Tong; Yan Li; Ying Tao
EPI_ISL_1094774	HI Dept. of Health, State Laboratories Division	Respiratory Viruses Branch, Division of Viral Diseases, Centers for Disease Control and Prevention	Anna Montmayeur; Anna Uehara; Ben L. Rambo-Martin; Clinton R. Paden; Dhwani Batra; Haibin Wang; Jasmine Padilla; Jing Zhang; Justin Lee; Krista Queen; Lori Rowe; Mark Burroughs; Mili Sheth; Peter W. Cook; Rachel Marine; Sarah Nobles; Suxiang Tong; Yan Li; Ying Tao
EPI_ISL_914808	HLE - ASOCIACION HOGAR PARA ANCIANOS PRESBITERO JAFET JIMENEZ MORALES DE GRECIA	Inciensa, Instituto Costarricense de Investigación y Enseñanza en Nutrición y Salud	Adriana Godínez; Claudio Soto-Garita; Estela Cordero; Francisco Duarte; Hebleen Porras; Melany Calderón & Mariel López
EPI_ISL_914807	HLE - ASOCIACION HOGAR DE ANCIANOS SANTIAGO CRESPO CALVO	Inciensa, Instituto Costarricense de Investigación y Enseñanza en Nutrición y Salud	Adriana Godínez; Claudio Soto-Garita; Estela Cordero; Francisco Duarte; Hebleen Porras; Melany Calderón & Mariel López
EPI_ISL_1336649	HOPITAL PRINCESSE GRACE	CNR Virus des Infections Respiratoires - France SUD	Antonin Bal; Bruno Lina; Gregory Destras; Gwendolyne Burfin; Hadrien Regue; Laurence Josset; Martine Valette; Quentin Semanas
EPI_ISL_1265608, EPI_ISL_1359964, EPI_ISL_1359968, EPI_ISL_1359971, EPI_ISL_1359977	HOPITAL UNIVERSITAIRE DE FORT DE FRANCE	CNR Virus des Infections Respiratoires - France SUD	Antonin Bal; Bruno Lina; Gregory Destras; Gwendolyne Burfin; Hadrien Regue; Laurence Josset; Martine Valette; Quentin Semanas
EPI_ISL_914818, EPI_ISL_914819	HOSPITAL DE NIÑOS DR. CARLOS SAENZ HERRERA	Inciensa, Instituto Costarricense de Investigación y Enseñanza en Nutrición y Salud	Adriana Godínez; Claudio Soto-Garita; Estela Cordero; Francisco Duarte; Hebleen Porras; Melany Calderón & Cristian Pérez-Corrales
EPI_ISL_914821, EPI_ISL_914822	HOSPITAL DR. ENRIQUE BALTODANO BRICEÑO	Inciensa, Instituto Costarricense de Investigación y Enseñanza en Nutrición y Salud	Adriana Godínez; Claudio Soto-Garita; Estela Cordero; Francisco Duarte; Hebleen Porras; Melany Calderón & Mariel López
EPI_ISL_1067623, EPI_ISL_1067624	HOSPITAL MEXICO	Inciensa, Instituto Costarricense de Investigación y Enseñanza en Nutrición y Salud	Adriana Godínez; Caterina Guzmán; Claudio Soto-Garita; Estela Cordero; Francisco Duarte; Hebleen Porras; Melany Calderón; Nazareth Ruiz & Teresita Somogyi
EPI_ISL_682273	HOSPITAL SAN JUAN DE DIOS	Inciensa, Instituto Costarricense de Investigación y Enseñanza en Nutrición y Salud	Adriana Godinez & Melany Calderon; Claudio Soto-Garita; Estela Cordero; Francisco Duarte; Hebleen Porras
EPI_ISL_636977	HP Pemba	KRISP, KZN Research Innovation and Sequencing Platform	Giandhari J; Ismael N; Nadia Sitoe; Nedio Mabunda; Paulo Arnaldo; Pillay S; Tegally H; Wilkinson E; de Oliveira T
EPI_ISL_421234	Hangzhou Center for Diseases Control and Prevention	Hangzhou Center for Diseases Control and Prevention	Haoqiu Wang; Hua Yu; Jun Li; Junfang Chen; Lingfeng Mao; Shuchang Chen; Xin Qian; Xinfen Yu; Xuchu Wang; Zhou Sun
EPI_ISL_700457, EPI_ISL_700477, EPI_ISL_700480, EPI_ISL_700494, EPI_ISL_700515, EPI_ISL_700516, EPI_ISL_700561, EPI_ISL_700562, EPI_ISL_700573, EPI_ISL_700585, EPI_ISL_700593			
see above	Hanover Park CHC wc HPH	NHLS/UCT	Arash Iranzadeh; Bruna Galvao; Carolyn Williamson; Deelan Doolabh; Diana Hardie; Innocent Mudau; Kruger Marais; Lynn Tyers; Marvin Hsiao; Stephen Korsman
EPI_ISL_796013, EPI_ISL_796014, EPI_ISL_796018, EPI_ISL_796026	Hebei Provincial Center for Disease Control and Prevention, Shijiazhuang, Hebei Province; National Institute for Viral Disease Control and Prevention, China CDC	Hebei Provincial Center for Disease Control and Prevention, Shijiazhuang, Hebei Province; National Institute for Viral Disease Control and Prevention, China CDC	George F. Gao; Nankun Liu; Qi Li; Shunxiang Qi; Wenbo Xu; Xiang Zhao; Yang Song
EPI_ISL_640018	Heidelberg Clinic wc HBC	NHLS/UCT	Arash Iranzadeh; Bruna Galvao; Carolyn Williamson; Deelan Doolabh; Diana Hardie; Innocent Mudau; Kruger Marais; Lynn Tyers; Marvin Hsiao; Stephen Korsman
EPI_ISL_700473, EPI_ISL_700522, EPI_ISL_700549	Heideveld CDC wc HVP	NHLS/UCT	Arash Iranzadeh; Bruna Galvao; Carolyn Williamson; Deelan Doolabh; Diana Hardie; Innocent Mudau; Kruger Marais; Lynn Tyers; Marvin Hsiao; Stephen Korsman
EPI_ISL_960170	Heideveld CDC wc HVP	National Health Laboratory Service/UCT	Arash Iranzadeh; Bruna Galvao; Carolyn Williamson; Deelan Doolabh; Diana Hardie; Innocent Mudau; Kruger Marais; Lynn Tyers; Marvin Hsiao; Stephen Korsman
EPI_ISL_700559, EPI_ISL_700574, EPI_ISL_700595, EPI_ISL_700598, EPI_ISL_1040662, EPI_ISL_1040798, EPI_ISL_1040803, EPI_ISL_1040806			
see above	Heideveld Emergency Centre	NHLS/UCT	Arash Iranzadeh; Bruna Galvao; Carolyn Williamson; Deelan Doolabh; Diana Hardie; Innocent Mudau; Kruger Marais; Lynn Tyers; Marvin Hsiao; Stephen Korsman
EPI_ISL_960153	Heideveld Emergency Centre	National Health Laboratory Service/UCT	Arash Iranzadeh; Bruna Galvao; Carolyn Williamson; Deelan Doolabh; Diana Hardie; Innocent Mudau; Kruger Marais; Lynn Tyers; Marvin Hsiao; Stephen Korsman
EPI_ISL_1336999, EPI_ISL_1444007, EPI_ISL_1460980, EPI_ISL_1479278	Helix/Illumina	Centers for Disease Control and Prevention Division of Viral Diseases, Pathogen Discovery	Adrian Paskey; Alexandre Bolze; Ary Ascencio; Ben L. Rambo-Martin; Benjamin Rambo-Martin; Brad Sickler; Charlotte Rivera-Garcia; Christine Tran; Christopher Gulvick; Clinton R. Paden; Dakota Howard; Darlene Wagner; David Becker; Dhwani Batra; Duncan MacCannell; Efren Sandoval; Eileen de Feo; Elizabeth Cirulli; Eric Allen; Geraint Levan; James Lu; Jan Antico; Jason Caravas; Jason Nguyen; Jimmy Ramirez; Jingtao Liu; Kara Moser; Kelly Schiabor Barrett; Kim Gietzen; Magnus Isaksson; Marc Laurent; Matthew Schmerer; Matthew Tolentino; Nicole L. Washington; Peter W. Cook; Phil Febbo; Ryan Cho; Scott Sammons; Shannon Wickline; Shatavia Morrison; Sherry Wang; Simon White; Summer Galloway; Suxiang Tong; Tyler Cassens; William Lee; Yvette Unoarumhi
EPI_ISL_966962, EPI_ISL_967007	Helix/Illumina	Respiratory Viruses Branch, Division of Viral Diseases, Centers for Disease Control and Prevention	; Alexandre Bolze; Ary Ascencio; Ben L. Rambo-Martin; Brad Sickler; Charlotte Rivera-Garcia; Christine Tran; Clinton R. Paden; Dakota Howard; David Becker; Dhwani Batra; Duncan MacCannell; Efren Sandoval; Eileen de Feo; Elizabeth Cirulli; Eric Allen; Geraint Levan; James Lu; Jan Antico; Jason Nguyen; Jimmy Ramirez; Jingtao Liu; Kelly Schiabor Barrett; Kim Gietzen; Magnus Isaksson; Marc Laurent; Matthew Tolentino; Nicole L. Washington; Peter W. Cook; Phil Febbo; Ryan Cho; Shannon Wickline; Sherry Wang; Simon White; Summer Galloway; Suxiang Tong; Tyler Cassens; William Lee
EPI_ISL_699942, EPI_ISL_700258	Hematopathology Laboratory, ACTREC, TMC	Hematopathology Laboratory, ACTREC, TMC	ACTREC; Hematopathology Laboratory
EPI_ISL_700565, EPI_ISL_700571	Herbertsdale Sat Clinic wc HBD	NHLS/UCT	Arash Iranzadeh; Bruna Galvao; Carolyn Williamson; Deelan Doolabh; Diana Hardie; Innocent Mudau; Kruger Marais; Lynn Tyers; Marvin Hsiao; Stephen Korsman
EPI_ISL_591485, EPI_ISL_872580	Histopath	NSW Health Pathology - Institute of Clinical Pathology and Medical Research; Westmead Hospital; University of Sydney	CIDM-PH et al.
EPI_ISL_770006	Hle - Asociacion Hogar De Ancianos De Palmar Sur De Osa	Inciensa, Instituto Costarricense de Investigación y Enseñanza en Nutrición y Salud	Adriana Godínez; Claudio Soto-Garita; Estela Cordero; Francisco Duarte; Hebleen Porras; Melany Calderón & Mariel López
EPI_ISL_412028	Hong Kong Department of Health	School of Public Health, The University of Hon g Kong	Daniel K.W. Chu; Dominic N.C. Tsang; Leo L.M. Poon; Malik Peiris
EPI_ISL_414517	Hong Kong Department of Health	School of Public Health, The University of Hong Kong	Daniel K.W. Chu; Dominic N.C. Tsang; Leo L.M. Poon; Malik Peiris
EPI_ISL_1166892, EPI_ISL_1166895, EPI_ISL_1166898, EPI_ISL_1166900, EPI_ISL_1166903, EPI_ISL_1166904, EPI_ISL_1166907, EPI_ISL_1166908, EPI_ISL_1166911, EPI_ISL_1166912, EPI_ISL_1166913, EPI_ISL_1166916, EPI_ISL_1166921, EPI_ISL_1166924, EPI_ISL_1166925, EPI_ISL_1166929, EPI_ISL_1166930, EPI_ISL_1166931, EPI_ISL_1166932, EPI_ISL_1166933, EPI_ISL_1166935, EPI_ISL_1166940, EPI_ISL_1166944, EPI_ISL_1166955, EPI_ISL_1166957, EPI_ISL_1166960, EPI_ISL_1166961, EPI_ISL_1166963, EPI_ISL_1166964, EPI_ISL_1166967, EPI_ISL_1166971, EPI_ISL_1166972, EPI_ISL_1166974, EPI_ISL_1166980, EPI_ISL_1166981, EPI_ISL_1166984, EPI_ISL_1167004, EPI_ISL_1168194, EPI_ISL_1201540, EPI_ISL_1259295, EPI_ISL_1259296			
see above	Hopital	National Reference Center for Viruses of Respiratory Infections, Institut Pasteur, Paris	Angela Brisebarre; Camille Capel; Castelain Sandrine; Combe Patrice; Etienne Simon-Lorière; Hermann CéCile; Marion Barbet; Maud Vanpeene; Méline Bizard; Sylvie Behillil; Sylvie van der Werf; Vincent Enouf
EPI_ISL_700458, EPI_ISL_700558	Hornlee Clinic wc HLC	NHLS/UCT	Arash Iranzadeh; Bruna Galvao; Carolyn Williamson; Deelan Doolabh; Diana Hardie; Houriiyah Tegally; Innocent Mudau; Kruger Marais; Lynn Tyers; Marvin Hsiao; Stephen Korsman
EPI_ISL_855360, EPI_ISL_855361, EPI_ISL_855362, EPI_ISL_855366, EPI_ISL_855368, EPI_ISL_855370, EPI_ISL_855374, EPI_ISL_855376, EPI_ISL_855380, EPI_ISL_890357, EPI_ISL_1381163, EPI_ISL_1540447			
see above	Hospital	National Reference Center for Viruses of Respiratory Infections, Institut Pasteur, Paris	Angela Brisebarre; Bastian Sylvaine; Camille Capel; Christophe Malabat; Combe Patrice; Corinne Maufrais; Etienne Simon-Lorière; Frédéric Lemoine; Louise Lefrançois; Marion Barbet; Maud Vanpeene; Méline Bizard; Sylvaine Bastian; Sylvie Behillil; Sylvie van der Werf; Vincent Enouf
EPI_ISL_1629773, EPI_ISL_1629781	Hospital Carlos Alberto Seguín Escobedo - EsSalud	Laboratorio de Genómica Microbiana, Universidad Peruana Cayetano Heredia	Alejandra Dávila-Barclay; Fernando Sánchez Fragoso; Guillermo Salvatierra; Janet Huancachoque; Lenin Maturrano; Lin Zevallos Cuarite; Luis González; Pablo Tsukayama; Pedro E. Romero; Pool Marcos
EPI_ISL_1383837, EPI_ISL_1383864	Hospital Center Emile Mayrisch	Laboratoire national de sante, Microbiology, Microbial Genomics Platform	Anke Wienecke-Baldacchino; Catherine Ragimbeau; Cynthia Oxacelay; Fatu Djabi; Jessica Tapp; Lise Pignon; Raoul Salmon; Tamir Abdelrahman
EPI_ISL_1384077	Hospital Center Luxembourg	Laboratoire national de sante, Microbiology, Microbial Genomics Platform	Anke Wienecke-Baldacchino; Catherine Ragimbeau; Fatu Djabi; Jean-Hugues Francois; Jessica Tapp; Lise Pignon; Michel Kohnen; Raoul Salmon; Tamir Abdelrahman
EPI_ISL_1533710	Hospital Estadual de Vila Alpina Org Social Seconci Sao Paulo	Instituto Adolfo Lutz, Interdiciplinary Procedures Center, Strategic Laboratory	Caio Vinicius Dias Lopes; Claudia Regina Gonçalves; Claudio Tavares Sacchi; Erica Valessa Ramos Gomes; Karoline Rodrigues Campos; Leonardo Jose Tadeu de Araujo

EPI_ISL_1524846	Hospital General Universitario Gregorio Marañón	Hospital General Universitario Gregorio Marañón	Cristina Rodriguez-Grande; Darío García de Viedma; Laura Pérez-Lago; Patricia Muñoz; Pedro Sola Campoy; Pilar Catalán; Sergio Buenestado Serrano
EPI_ISL_1527021	Hospital Guápiles	Inciensa, Instituto Costarricense de Investigación y Enseñanza en Nutrición y Salud	Barboza-Arguedas E & Cerdas-Quesada C; Pérez-Corrales C
EPI_ISL_527739	Hospital Mexico [San Jose/San Jose]	Inciensa, Instituto Costarricense de Investigación y Enseñanza en Nutrición y Salud	Adriana Godinez & Melany Calderon; Claudio Soto-Garita; Estela Cordero; Francisco Duarte; Hebleen Porras
EPI_ISL_1303540	Hospital Municipal Cidade Tiradentes Carmen Prudente	Instituto Adolfo Lutz, Interdiciplinary Procedures Center, Strategic Laboratory	Caio Vinicius Dias Lopes; Claudia Regina Gonçalves; Claudio Tavares Sacchi; Erica Valessa Ramos Gomes; Karoline Rodrigues Campos
EPI_ISL_977490	Hospital Municipal Guido Guida	Instituto Adolfo Lutz, Interdiciplinary Procedures Center, Strategic Laboratory	Claudia Regina Gonçalves; Claudio Tavares Sacchi; Erica Valessa Ramos Gomes; Karoline Rodrigues Campos
EPI_ISL_1303539	Hospital Presidente	Instituto Adolfo Lutz, Interdiciplinary Procedures Center, Strategic Laboratory	Caio Vinicius Dias Lopes; Claudia Regina Gonçalves; Claudio Tavares Sacchi; Erica Valessa Ramos Gomes; Karoline Rodrigues Campos
EPI_ISL_491450	Hospital San Juan de Dios	Inciensa, Instituto Costarricense de Investigación y Enseñanza en Nutrición y Salud	Adriana Godinez & Melany Calderon; Claudio Soto-Garita; Estela Cordero; Francisco Duarte; Hebleen Brenes
EPI_ISL_861646	Hospital Santa Marcelina Sao Paulo	Instituto Adolfo Lutz, Interdiciplinary Procedures Center, Strategic Laboratory	Claudia Regina Gonçalves; Claudio Tavares Sacchi; Erica Valessa Ramos Gomes; Karoline Rodrigues Campos
EPI_ISL_693201	Hospital Sao Paulo de Ensino da Unifesp	Instituto Adolfo Lutz, Interdiciplinary Procedures Center, Strategic Laboratory	Claudia Regina Gonçalves; Claudio Tavares Sacchi; Erica Valessa Ramos Gomes; Karoline Rodrigues Campos
EPI_ISL_2249248, EPI_ISL_2249252	Hospital Sharp	Microbial Genomics Laboratory	Alejandra Garcia-Gasca; Bruno Gomez-Gil; Daniel Fregoso-Rueda; Julissa Enciso-Ibarra
EPI_ISL_819352, EPI_ISL_1020582	Hospital Universitari Vall d'Hebron - Vall d'Hebron Institut de Recerca	Hospital Universitari Vall d'Hebron - Vall d'Hebron Institut de Recerca	Andrés Antón; Ariadna Rando; Carla Castillo; Cristina Andrés; Damir Garcia-Cehic; Josep F Abril; Josep Quer; Juliana Esperalba; Maria Carmen Martin; Maria Gema Codina; Maria Piñana; Tomàs Pumarola
EPI_ISL_590915, EPI_ISL_1599407	Hospital of Southern Norway - Kristiansand, Department of Medical Microbiology	Norwegian Institute of Public Health, Department of Virology	Atiya R Ali; Debech Nadia; Engebretsen Serina Beate; Garcia Llorente Ignacio; Hilde Elshaug; Hilde Vollan; Jon Bråte; Kamilla Heddeland Instefjord; Karoline Bragstad; Kathrine Stene-Johansen; Marie Paulsen Madsen; Olav Hungnes; Pedersen Benedikte Nevjen; Rasmus Riis Kopperud
EPI_ISL_545111	Houston Methodist Hospital	Houston Methodist Hospital	Chia-Wei Chou; Concepcion C. Cantu; Daniel Boutz; David W. Bernard; Ghazaleh Eskandari; Heather Hendrickson; Hoang A. T. Nguyen; Hung-Che Kuo; Ilya J. Finkelstein; J. Hunter Long; James J. Davis; Jason S. McLellan; Jimmy Gollihar; Jule Goike; Kamyab Javanmardi; Layne Pruitt; Marcus Nguyen; Matthew Ojeda Saavedra; Maulik Shukla; Muthiah Kumaraswami; Paul A. Christensen; Prasanti Yerramilli; Randall J. Olsen; S. Wesley Long; Sishir Subedi; and James M. Musser
EPI_ISL_645030, EPI_ISL_645041	Human Genome Variation Research Group, Malopolska Centre of Biotechnology	Human Genome Variation Research Group, Malopolska Centre of Biotechnology	Botwina, P.; Branicki, W.; Dabrowska, A.; Foremny, J.; Gromowski, T.; Klajmon, A.; Kopera, K.; Kowalski, M.; Labaj; Marszalek, K.; Owczarek, K.; P.P.; Pisarek, A.; Pospiech, E.; Pyrc, K.; Sanak, M.; Swadzba, J.; Szczepanski, A.
EPI_ISL_759956	Husada Utama Hospital	Institute of Tropical Disease, Universitas Airlangga	Aldise M Nastri; Didi Dewanto; Gatot Soegiarto; Jezzy R Dewantari; Kazufumi Shimizu; Krisnoadi Rahardjo; Laksmi Wulandari; Maria I Lusida; Resti Yudhawati; Rima R Prasetya; Soetjipto; Yasuko Mori
EPI_ISL_710534, EPI_ISL_710541, EPI_ISL_712060	Hôpital Fattouma-Bourguiba de Monastir	Laboratoire des Procédés de Criblage Moléculaire et Cellulaire-Centre de Biotechnologie de Sfax	A. and Masmoudi, S.; Abdelmoulah, F.; Abid, N.; Ajili, F.; Aouni, M.; Ben Ayed, I.; Bensaid, M.; Chtourou, A.; Elargoubi, A.; Fki-berrajah, L.; Gaaloul, I.; Gargouri, S.; Hammami, A.; Kamoun, S.; Karray Hakim, H.; Kharat, N.; Mastouri, M.; Mhalla, S.; Nabli, A.; Rebai; Smeti, I.; Souissi, A.; Stambouli, N.; Turki, M.
EPI_ISL_961574, EPI_ISL_961615	Hôpital Georges L. Dumont	National Microbiology Laboratory (NML)	Anna Majer; Anneliese Landgraff; CanCOGeN's metadata curation team; Darian Hole; Elsie Grudeski; Gary Van Domselaar; Grace Seo; Guillaume Desnoyers; Jennifer Tanner; Kirsten Biggar; Madison Chapel; Morag Graham; Natalie Knox; Nathalie Bastien; Philip Mabon; Public Health Agency of Canada CanCOGeN team; Rhiannon Huzarewich; Richard Garceau; Russell Mandes; Shari Tyson; Timothy Booth; Yan Li
EPI_ISL_912975	Hôpital Henri Mondor	Department of Virology, Henri Mondor University Hospital, Assistance Publique Hôpitaux de Paris, Université Paris-Est Créteil, INSERM U955	Alexandre Soulier; Christophe Rodriguez; Elisabeth Trawinski; Guillaume Gricourt; Jean-Michel Pawlotsky; Melissa N'Debi; Slim Fourati; Vanessa Demontant
EPI_ISL_1094697	IA State Hygienic Laboratory	Respiratory Viruses Branch, Division of Viral Diseases, Centers for Disease Control and Prevention	Anna Montmayeur; Anna Uehara; Ben L. Rambo-Martin; Clinton R. Paden; Dhwani Batra; Haibin Wang; Jasmine Padilla; Jing Zhang; Justin Lee; Krista Queen; Lori Rowe; Mark Burroughs; Mili Sheth; Peter W. Cook; Rachel Marine; Sarah Nobles; Suxiang Tong; Yan Li; Ying Tao
EPI_ISL_1171619	IAL Regional de Presidente Prudente	Instituto Adolfo Lutz, Interdiciplinary Procedures Center, Strategic Laboratory	Claudia Regina Gonçalves; Claudio Tavares Sacchi; Erica Valessa Ramos Gomes; Karoline Rodrigues Campos
EPI_ISL_1171624	IAL Regional de Santos	Instituto Adolfo Lutz, Interdiciplinary Procedures Center, Strategic Laboratory	Caio Vinicius Dias Lopes; Claudia Regina Gonçalves; Claudio Tavares Sacchi; Erica Valessa Ramos Gomes; Karoline Rodrigues Campos
EPI_ISL_1400479, EPI_ISL_1400483, EPI_ISL_1626589, EPI_ISL_1648063, EPI_ISL_1648075	IN State Department of Health Laboratory Services	IN State Department of Health Laboratory Services	Ankita Kashikar; Brian Pope; Cassandra Campion; Jamie Yeadon; Kyle Brownlee; Lixia Liu; Mark Glazier; Melissa Hindenlang
EPI_ISL_1577817	INHRR	Laboratorio de Virología Molecular	Aguilar M; Alarcon V; D Angelo P; Delgado M; Garzaro D; Jaspe RC; Loureiro CL; Pujol FH; Rangel HR; Rodriguez L; Zambrano JL
EPI_ISL_410545	INMI Lazzaro Spallanzani IRCCS	Laboratory of Virology, INMI Lazzaro Spallanzani IRCCS	Barbara Bartolini; Cesare E. M. Gruber; Concetta Castilletti; Eleonora Lalle; Emanuela Giombini; Emanuele Nicastri; Fabrizio Carletti; Francesca Colavita; Francesco Messina; Giuseppe Ippolito.; Maria R. Capobianchi; Martina Rueca
EPI_ISL_1419264, EPI_ISL_1419447, EPI_ISL_1419513, EPI_ISL_1419623	INSACOG-WB	National Institute of Biomedical Genomics – INSACOG	Ajay Chakraborti; Arindam Maitra; Bhaswati Bandyopadhyay; Nidhan Kumar Biswas; Saumitra Das; Sreedhar Chinnaswamy; Tamal Ghosh
EPI_ISL_826822	INSPI-CRN DE INFLUENZA Y OTROS VIRUS RESPIRATORIOS	Instituto de Salud Publica de Chile	Alfredo Bruno; Andres Castillo; Barbara Parra; Domenica de Mora; Gisselle Barra; Jaime Lagos; Javier Tognarelli; Jimmy Garcez; Jorge Fernandez; Loredana Arata; Manuel Gonzalez; Martiza Olmedo; Michelle Paez; Patricia Bustos; Rodrigo Fasce; Solon Narvaez
EPI_ISL_940769, EPI_ISL_940773	INSPI-CRN de Influenza y otros virus respiratorios	INSPI-Centro de Investigación Multidisciplinaria de la DTIDI	Alberto Orlando.; Alfredo Bruno; Andrés Carrazco-Montalvo; Doménica de Mora; Leandro Patiño; Manuel González; Maritza Olmedo; Mary Regato-Arrata; Melissa Zambrano; Orson Mestanza
EPI_ISL_1399391	IPO Lisboa	Instituto Nacional de Saude (INSA) and Instituto Gulbenkian de Ciencia (IGC)	Borges et al
EPI_ISL_751425	IRCCS Sacro Cuore Don Calabria Hospital, Department of Infectious, Tropical Diseases & Microbiology	University of Verona, Department of Biotechnology	Antonio Mori; Chiara Degli Esposti; Chiara Piubelli; Cristina Beltrami; Elena Pomari; Emanuela Cosentino; Giulia Lopatriello; Luca Marcolungo; Massimo Delledonne; Michela Deiana
EPI_ISL_1164802	Idaho Bureau of Laboratories	IBL	"R. Beukelman; Aimee Ceniseros; Christopher Ball"; Matthew C. Burns; Robert L. Voermans
EPI_ISL_1063894	Immunogenomics lab, Institute of Life Sciences, Bhubaneswar	Immunogenomics lab, Institute of Life Sciences, Bhubaneswar	Ajay Parida; Amol M. Kanampalliwar; Arup Ghosh; Atimukta Jha; INSACOG Consortium; Kirtal Hansdah; Punit Prasad; Rajeeb Swain; Rupesh Dash; Safal Walia; Shifu Aggarwal; Sunil K. Raghav
EPI_ISL_1018071, EPI_ISL_1018073, EPI_ISL_1018074, EPI_ISL_1018076, EPI_ISL_1018077, EPI_ISL_1018079, EPI_ISL_1018083, EPI_ISL_1018084, EPI_ISL_1018085, EPI_ISL_1018088, EPI_ISL_1018089, EPI_ISL_1018090, EPI_ISL_1018092, EPI_ISL_1018095, EPI_ISL_1018097, EPI_ISL_1018099, EPI_ISL_1018100			
see above	Immunology, Noguchi Memorial Institute for Medical Research	Immunology, Noguchi Memorial Institute for Medical Research	Adu, B.; Adusei-Poku; Agbodzi, B.; Ampofo; Appiah-Kubi, J.; Asare; Bonney; Egyir, B.; J.K.; K.M.; Kumordjie, S.; M.A.; Mohktar, Q.; Odoom; Oteng, F.; Owusu-Nyantakyi, C.; W.K.; Yeboah, C.
EPI_ISL_1628624	InDRE	Instituto Nacional de Medicina Genomica	Alcaraz N; Canseco Mendez JC; Cedro-Tanda A; Garcia-Cardenas FJ; Gisela Barrera-Badillo; Gonzalez-Barrera D; Gonzalez-Woge MA; Herrera-Montalvo LA.; Hidalgo-Miranda A; Irma Lopez-Martinez; Jose Ernesto Ramirez González; Mendoza-Vargas A; Miranda-Ortiz H; Munguia-Garza P; Ramirez-Vega O; Rangel-DeLeon D; Reyes-Grajeda JP; Rosas-Escobar P
EPI_ISL_1517392, EPI_ISL_1517419, EPI_ISL_1517421, EPI_ISL_1517431	Inciensa, Instituto Costarricense de Investigación y Enseñanza en Nutrición y Salud	Inciensa, Instituto Costarricense de Investigación y Enseñanza en Nutrición y Salud	Barboza-Arguedas E & Blanco-Delgado C; Cristian Peréz-Corrales; Valeria Peralta-Barquero & César Cerdas Quesada; Valeria Peralta-Barquero & Gallegos-Carrillo B; Valeria Peralta-Barquero & Patricia Salas
EPI_ISL_476704	Incubadora Venezolana de Ciencia, Venezuela	Incubadora Venezolana de Ciencia, Venezuela / Instituto Nacional de Salud, Bogotá, Colombia / Grupo de Investigaciones Microbiológicas-UR (GIMUR), Departamento de Biología, Facultad de Ciencias Naturales, Universidad del Rosario, Bogotá, Colombia / Icahn School of Medicine at Mount Sinai, New York, USA	Alberto Paniz-Mondolfi; Ana S. Gonzalez-Reiche; Angelica Rico; Aníbal A. Teherán; Carolina Florez; Carolina Hernández; Emilia Mia Sordillo; Esther C. Barros; Harm van Bakel; Jesús E. Jaimes; Juan David Ramírez; Lisseth Pardo; Lourdes Delgado; Luis Perez-Garcia; Marina Muñoz; Matthew M. Hernandez; Sergio Gomez; Viviana Simon
EPI_ISL_413522	Indian Council of Medical Research - National Institute of Virology	National Influenza Center, Indian Council of Medical Research - National Institute of Virology	Choudhary ML; Potdar V; Shete-Aich A; Yadav PD
EPI_ISL_1588047	Infinity Biologix	Centers for Disease Control and Prevention Division of Viral Diseases, Pathogen Discovery	Adrian Paskey; Benjamin Rambo-Martin; Chirayu Goswami; Christian Bixby; Christopher Gulvick; Clinton R. Paden; Dakota Howard; Darlene Wagner; Dhwani Batra; Duncan MacCannell; Jason Caravas; Jonathan Schultz; Kara Moser; Matthew Schmerer; Peter W. Cook; Robin Grimwood; Russ Hager; Scott Sammons; Shatavia Morrison; Yihe Wang; Yvette Unoarumhi
EPI_ISL_1395831	Inmunología del Hospital Perrando e Instituto de Medicina Regional de la UNNE	Grupo de Genómica y Bioinformática del Instituto de Investigación de la Cadena Láctea CONICET-INTA on behalf of 'Proyecto Argentino Interinstitucional de genomica de SARS-CoV-2' (PAIS Consortium)	AF; Amadio; Antonieta Cayré; Eberhardt; Gerardo Deluca; Gustavo Giusiano; Horacio Lucero; Irazoqui; Laura Lescano; MF; Marcelo Marin; María Delia Foussal; María Verónica Gómez; Natalia Andrea Ayala
EPI_ISL_1404615, EPI_ISL_1435763, EPI_ISL_1436817, EPI_ISL_1439647, EPI_ISL_1440879	Institut National d'Hygiène	Unité Mixte Internationale TransVIHMI (UMI 233 IRD – U1175 INSERM - Université de Montpellier) IRD (Institut de recherche pour le développement)	Abla A. KONOU; Adodo SADJI; Ahidjo AYOUBA; Akoélé SILIADIN; Alassane OURO-MEDELI; Amivi EHLAN; Améyo DORKENOO; Anoumou DAGNRA; Christelle BUTEL; Déléma MABA; Eric DELAPORTE; Issaka Maman; Kokou TEGUENI; Laetitia SERRANO; Martine PEETERS; Messanh DOUFFAN; Mireille PRINCE-DAVID; Mounerou SALOU; Sidonie A.M.KAGNISSODE; Sika DOSSIM; Wembo A. HALATOKO
EPI_ISL_1415293, EPI_ISL_1415425, EPI_ISL_1415428, EPI_ISL_1442993, EPI_ISL_1443000, EPI_ISL_1443001, EPI_ISL_1443002, EPI_ISL_1443003			
see above	Institut National d'hygiène	"Unité Mixte Internationale TransVIHMI (UMI 233 IRD – U1175 INSERM - Université de Montpellier) IRD (Institut de recherche pour le	Abla A. KONOU; Adodo SADJI; Ahidjo AYOUBA; Akoélé SILIADIN; Alassane OURO-MEDELI; Amivi EHLAN; Améyo DORKENOO; Anoumou DAGNRA; Christelle BUTEL; Déléma MABA; Eric DELAPORTE; Issaka Maman; Kokou TEGUENI; Laetitia SERRANO; Martine PEETERS; Messanh DOUFFAN; Mireille PRINCE-DAVID; Mounerou SALOU; Sidonie A.M.KAGNISSODE; Sika DOSSIM; Wembo A. HALATOKO

		développement)"	
EPI_ISL_1508953, EPI_ISL_1508960, EPI_ISL_1508998, EPI_ISL_1509101	Institut National d'hygiène	Unité Mixte Internationale TransVIHMI (UMI 233 IRD – U1175 INSERM - Université de Montpellier) IRD (Institut de recherche pour le développement)	Abla A. KONOU; Adodo SADJI; Ahidjo AYOUBA; Akoélé SILIADIN; Alassane OURO-MEDELI; Amivi EHLAN; Améyo DORKENOO; Anoumou DAGNRA; Christelle BUTEL; Déléma MABA; Eric DELAPORTE; Issaka Maman; Kokou TEGUENI; Koku AGBODEKA; Laetitia SERRANO; Martine PEETERS; Messanh DOUFFAN; Mireille PRINCE-DAVID; Mounerou SALOU; Sidonie A.M.KAGNISSODE; Sika DOSSIM; Syntyche DEVATCHAGNI; Wembo A. HALATOKO
EPI_ISL_1293351, EPI_ISL_1303031, EPI_ISL_1321604, EPI_ISL_1406177, EPI_ISL_1445275, EPI_ISL_1500948	Institut National d'Hygiène (INH)	Unité Mixte Internationale TransVIHMI (UMI 233 IRD – U1175 INSERM - Université de Montpellier)IRD (Institut de recherche pour le développement)	Abla A. KONOU; Adodo SADJI; Ahidjo AYOUBA; Akoélé SILIADIN; Alassane OURO-MEDELI; Amivi EHLAN; Améyo DORKENOO; Anoumou DAGNRA; Christelle BUTEL; Déléma MABA; Eric DELAPORTE; Issaka Maman; Kokou TEGUENI; Laetitia SERRANO; Martine PEETERS; Messanh DOUFFAN; Mireille PRINCE-DAVID; Mounerou SALOU; Sidonie A.M.KAGNISSODE; Sika DOSSIM; Wembo A. HALATOKO
EPI_ISL_418207, EPI_ISL_418209, EPI_ISL_418210, EPI_ISL_418213, EPI_ISL_418216, EPI_ISL_420069, EPI_ISL_420070, EPI_ISL_420073, EPI_ISL_420074, EPI_ISL_420076, EPI_ISL_420077, EPI_ISL_476492, EPI_ISL_476493, EPI_ISL_476494, EPI_ISL_476514, EPI_ISL_476562, EPI_ISL_476564, EPI_ISL_476569, EPI_ISL_476572, EPI_ISL_480554, EPI_ISL_480783, EPI_ISL_480789, EPI_ISL_481236, EPI_ISL_481237, EPI_ISL_481239, EPI_ISL_481243, EPI_ISL_482874, EPI_ISL_482877, EPI_ISL_485635, EPI_ISL_485708, EPI_ISL_485710, EPI_ISL_485713, EPI_ISL_485715, EPI_ISL_485716, EPI_ISL_486860, EPI_ISL_486861, EPI_ISL_486862, EPI_ISL_486865, EPI_ISL_486866, EPI_ISL_486870, EPI_ISL_486871, EPI_ISL_486872, EPI_ISL_486873			
see above	Institut Pasteur Dakar	Institut Pasteur de Dakar	Amadou Alpha Sall; Amadou Alpha Sall.; Amadou alpha Sall; Mamadou Diop; Mamadou Malado Jallow; Mamadou diop; Mamadou malado Jallow; Marie Henriette Dior Ndione; Moussa Moise; Moussa Moise Diagne; Ndongo Dia; Ousmane Faye; Safietou Sanke; Safietou Sankhe
EPI_ISL_498230, EPI_ISL_498238, EPI_ISL_498242, EPI_ISL_498243	Institut Pasteur de Dakar	Institut Pasteur de Dakar	Amadou Alpha Sall.; Mamadou Diop; Mamadou Malado Jallow; Marie Henriette Dior Ndione; Moussa Moise Diagne; Ndongo Dia; Ousmane Faye; Safietou Sankhe Mbengue
EPI_ISL_1013424, EPI_ISL_1013430	Institut Pasteur de Guadeloupe	National Reference Center for Viruses of Respiratory Infections, Institut Pasteur, Paris	Angela Brisebarre; Camille Capel; Etienne Simon-Lorière; Marion Barbet; Maud Vanpeene; Méline Bizard; Sylvie Behillil; Sylvie van der Werf; Talarmin Antoine; Vincent Enouf
EPI_ISL_613421, EPI_ISL_613429, EPI_ISL_613430, EPI_ISL_613436	Institut Pasteur de la Guadeloupe	Institut Pasteur de la Guadeloupe	Angela Brisebarre; Antoine Talarmin; Camille Capel; Etienne Simon-Lorière; Marion Barbet; Maud Vanpeene; Méline Bizard; Stéphanie Guyomard; Sylvie Behillil; Sylvie van der Werf; Sébastien Breurec; Vincent Enouf
EPI_ISL_459965, EPI_ISL_459966, EPI_ISL_459972, EPI_ISL_459973, EPI_ISL_459975, EPI_ISL_459976, EPI_ISL_459977, EPI_ISL_459978, EPI_ISL_459980, EPI_ISL_459981, EPI_ISL_459982, EPI_ISL_459983			
see above	Institut Pasteur du Maroc	Institut Pasteur du Maroc	Abdellah Faouzi; Anass Abbad; Anderrahmane Maaroufi; Angela Brisebarre; Camille Capel; Etienne Simon-Lorière; Jalal Nourlil; Latifa Anga; Marion Barbet; Maud Vanpeene; Mjid Eloualid; Méline Bizard; Sylvie Behillil; Sylvie van der Werf; Vincent Enouf
EPI_ISL_955165	Institute for Biocides and Medical Ecology	Institute of microbiology and Immunology, Faculty of Medicine, University of Belgrade	Jankovic, M.; Jovanovic, T.; Knezevic, A.; Milicevic, O.; Sekler, M.; Tesovic, B.; Vidanovic, D.
EPI_ISL_678251	Institute for Lung Diseases in Children - Skopje	Research Center for Genetic Engineering and Biotechnology "Georgi D. Efremov" , Macedonian Academy of Sciences and Arts	RCGEB - MASA
EPI_ISL_490089, EPI_ISL_490093, EPI_ISL_490097, EPI_ISL_644672, EPI_ISL_718276, EPI_ISL_718282, EPI_ISL_944099, EPI_ISL_944102, EPI_ISL_944104, EPI_ISL_1114724, EPI_ISL_1263458, EPI_ISL_1406281, EPI_ISL_1424468			
see above	Institute for Medical Research, Infectious Disease Research Centre, National Institutes of Health, Ministry of Health Malaysia	Institute for Medical Research, Infectious Disease Research Centre, National Institutes of Health, Ministry of Health Malaysia	Azizan MA; Kalyanasundram J; Kamel K; Mohd Zawawi Z; Mohd-Zawawi Z; Suppiah J; Thayan R
EPI_ISL_1578530	Institute for Public Health of Zagreb County	Croatian Institute of Public Health	Irena Tabain; Ivana Ferenčak
EPI_ISL_891267	Institute of Biocides and Medical Ecology, Belgarde, Serbia	Virology department Institute of microbiology and immunology Faculty of Medicine University of Belgrade	Abazovic Dzihan; Banko Ana; Despot Dragana; Loncar Ana; Milicevic Ognjen; Miljanovic Danijela
EPI_ISL_2284889, EPI_ISL_2284892	Institute of Epidemiology, Disease Control and Research (IEDCR)	IEDCR-ideSHi-icddr,b	Firdausi Qadri; Hassan Afrad; Manjur Hossain Khan; Sadia Rahman; Tahmina Shirin
EPI_ISL_1499009, EPI_ISL_1499088, EPI_ISL_1647040	Institute of Microbiology and Immunology, Faculty of Medicine, University of Ljubljana	Institute of Microbiology and Immunology, Faculty of Medicine, University of Ljubljana	Alen Suljič; Andraž Celar; Dominika Šturm; Doroteja Vlaj; Mario Poljak; Matic Brvar; Miša Korva; Patricija Pozvek; Samo Zakotnik; Tatjana Avšič – Županc; Tomaž Mark Zorec; Špela Pleh
EPI_ISL_477015, EPI_ISL_486848, EPI_ISL_491938, EPI_ISL_491940, EPI_ISL_516648, EPI_ISL_516650, EPI_ISL_660536, EPI_ISL_660539, EPI_ISL_697800, EPI_ISL_824285, EPI_ISL_824286, EPI_ISL_877555, EPI_ISL_1656147			
see above	Institute of Microbiology, Universidad San Francisco de Quito	Institute of Microbiology, Universidad San Francisco de Quito	Alexandra Tino; Andrea Cunguan; Belén Prado-Vivar; Bernardo Gutiérrez; Carla Torres; Carlos Mena; Edmundo Encalada; Francisco Cordova; Freddy Saldarriaga-Mera; Gabriel Trueba; Hermelinda Paguay; Jonathan Araujo; Jorge Luis Velez; Jorge Montaño; Juan José Guadalupe; Kyllen Briones; Ligia Briceño; Maureen Mosquera; Mayra Perero Intriago; Michelle Grunauer; Miguel Sacoto Mazini; Monica Becerra-Wong; Nabih Dahik; Ninfa Hernandez; Patricio Reyes; Patricio Rojas-Silva; Paúl Cárdenas; Prado-Vivar; Rosario Erazo; Sully Márquez; Tania Guayasamin; Verónica Barragán
EPI_ISL_1443645, EPI_ISL_1443662, EPI_ISL_1443664	Institute of Microbiology, Universidad San Francisco de Quito	Omics Sciences Laboratory	; Andrea Cunguan; Belén Prado-Vivar; Bernardo Gutiérrez; Darlyn Amaya; Dayron Brossad; Derly Andrade Molina; Emily Sulay Saltos Montalvo; Fernanda Zurita; Gabriel Morey León; Gabriel Trueba; Juan Carlos Fernández Cadena; Juan José Guadalupe; Katheryn Sacheri Viteri; Michelle Grunauer; Monica Becerra-Wong; Nabih Dahik; Patricio Rojas-Silva; Paula Juliana Gavilanes Jarrín; Paúl Cárdenas; Rubén Armas González; Sully Márquez; Verónica Barragán
EPI_ISL_1499526, EPI_ISL_1499534	Institute of Public Health of Dubrovnik-Neretva County	Croatian Institute of Public Health	Irena Tabain; Ivana Ferenčak
EPI_ISL_1499505	Institute of Public Health of Međimurje county	Croatian Institute of Public Health	Irena Tabain; Ivana Ferenčak
EPI_ISL_1436002	Institute of Public Health of Varazdin county	Croatian Institute of Public Health	Irena Tabain; Ivana Ferenčak
EPI_ISL_577734, EPI_ISL_577738, EPI_ISL_577740, EPI_ISL_577741, EPI_ISL_583485, EPI_ISL_875523, EPI_ISL_875538, EPI_ISL_959608, EPI_ISL_959611, EPI_ISL_1234415			
see above	Institute of Virology, Biomedical Research Center of the Slovak Academy of Sciences, Bratislava	Faculty of Natural Sciences, Comenius University, Bratislava	Boris Klempa; Broňa Brejová; Dominika Fričová; Edita Staroňová; Elena Tichá; Jozef Nosek; Juraj Kopáček; Kristína Boršová; Martina Ličková; Martina Neboháčová; Monika Sláviková; Sabina Fumačová Havlíková; Tomáš Vinař; Viktória Hodorová; Viktória Čabanová; Ľubomíra Lukáčiková
EPI_ISL_1588667	Institute of Virology, Medical Center, University of Freiburg, Freiburg, Germany	Institute of Virology, Clinial Virus Genomics, Medical Center, University of Freiburg, Freiburg, Germany	Hajo Grundmann; Jonas Fuchs; Lena Jaki; Lisa Kern; Marcus Panning; Sandra Reuter
EPI_ISL_1654812, EPI_ISL_1654818, EPI_ISL_1654820, EPI_ISL_1654825, EPI_ISL_1654826, EPI_ISL_1654830, EPI_ISL_1654838, EPI_ISL_1654840			
see above	Institute of Virology, Vaccines and Sera "Torlak"	Institute of microbiology and Immunology, Faculty of Medicine, University of Belgrade	Jankovic, M.; Jovanovic, T.; Knezevic, A.; Milicevic, O.; Sekler, M.; Tesovic, B.; Vidanovic, D.
EPI_ISL_1017681, EPI_ISL_1017687, EPI_ISL_1017694, EPI_ISL_1017697	Institute of Virology, Vaccines and Sera ''Torlak''	Institute of microbiology and Immunology, Faculty of Medicine, University of Belgrade	Jankovic, M.; Jovanovic, T.; Knezevic, A.; Milicevic, O.; Sekler, M.; Tesovic, B.; Vidanovic, D.
EPI_ISL_1628347	Instituto Adolfo Lutz Central	Instituto Adolfo Lutz, Interdiciplinary Procedures Center, Strategic Laboratory	Caio Vinicius Dias Lopes; Claudia Regina Gonçalves; Claudio Tavares Sacchi; Erica Valessa Ramos Gomes; Karoline Rodrigues Campos; Katia Correa de Oliveira Santos; Leonardo Jose Tadeu de Araujo
EPI_ISL_837786	Instituto Nacional de Enfermedades Respiratorias (INER)	Instituto Nacional de Enfermedades Respiratorias (INER)	Alejandra Hernández-Terán; Alma Rincón-Rubio; Celia Boukadida; Edgar Sevilla-Reyes; Eduardo Becerril-Vargas; Fidencio Mejía-Nepomuceno; Hector Esteban Paz-Juárez; Joel Armando Vázquez-Pérez; Jorge Salas-Hernández; José Arturo Martínez-Orozco; Margarita Matías-Florentino; Mario Mújica-Sánchez; Olivia Briceño; Santiago Ávila-Ríos
EPI_ISL_1080444	Instituto Nacional de Medicina Genomica	Instituto Nacional de Medicina Genomica	Alcaraz N; Cedro-Tanda A; Cisneros-Villanueva M; Gonzalez-Barrera D; Herrera-Montalvo LA; Hidalgo-Miranda A; Mendoza-Vargas A; Ramirez-Vega O; Rangel-DeLeon D; Reyes-Grajeda JP; Sifuentes-Rojas C
EPI_ISL_536493, EPI_ISL_536515, EPI_ISL_536553	Instituto Nacional de Salud	Laboratorio de Infecciones Respiratorias Agudas	David Tarazona; Dennis Carhuaricra; Eduardo Juscamayta Lopez; Faviola Valdivia Guerrero; Lenin Maturrano Hernandez; Nancy Rojas Serrano; Ronnie Gavilan Chavez
EPI_ISL_791093	Instituto Nacional de Salud - Unidad de Secuenciación y Análisis Genómico	Instituto Nacional de Salud - Dirección de Investigación en Salud Pública	Carlos Franco-Muñoz; Diego A. Álvarez-Díaz; Diego Andrés Prada; Gerardo Santamaría; Jonathan Reales; Julian Naizaque; Katherine Laiton-Donato; Magdalena Wiesner; Marcela Mercado-Reyes; Maria T. Herrera; Martha Lucia Ospina Martinez; Mauricio Pacheco-Montealegre; Paola Muñoz-Laiton; Sheryl Corchuelo
EPI_ISL_941991	Instituto Nacional de Salud, Bogotá, Colombia	Centro de Investigaciones en Microbiología y Biotecnología-UR (CIMBIUR), Facultad de Ciencias Naturales, Universidad del Rosario, Bogotá, Colombia Instituto Nacional de Salud, Bogotá, Colombia Icahn School of Medicine at Mount Sinai, New York, USA	Adriana van de Guchte; Alberto Paniz-Mondolfi; Ana S. Gonzalez-Reiche; Carolina Flórez; Carolina Hernández; Emilia Mia Sordillo; Hala Alejel Alshammary; Harm van Bakel; Jayeeta Dutta; Juan David Ramírez; Luz Helena Patiño; Marina Muñoz; Matthew M. Hernandez; Nathalia Ballesteros; Sergio Gomez; Viviana Simon; Zenab Khan
EPI_ISL_447796	Instituto Nacional de Salud, Bogotá, Colombia	Grupo de Investigaciones Microbiológicas-UR (GIMUR), Departamento de Biología, Facultad de Ciencias Naturales, Universidad del Rosario, Bogotá, Colombia Instituto Nacional de Salud, Bogotá, Colombia Icahn School of Medicine at Mount Sinai, New York, USA	Adriana Castillo; Alberto Paniz-Mondolfi; Ana S. Gonzalez-Reiche; Angelica Rico; Aníbal A. Teherán; Carolina Florez; Carolina Hernandez; David Martínez; Emilia Mia Sordillo; Esther C. Barros; Harm van Bakel; Jesús E. Jaimes; Juan David Ramírez; Laura Vega; Lisseth Pardo; Marina Muñoz; Martha L. Ospina; Matthew M. Hernandez; Nathalia Ballesteros; Sergio Castañeda; Sergio Gomez; Viviana Simon
EPI_ISL_887428, EPI_ISL_887441, EPI_ISL_887457, EPI_ISL_887460, EPI_ISL_887461, EPI_ISL_887462, EPI_ISL_887463, EPI_ISL_887466, EPI_ISL_887467, EPI_ISL_887471, EPI_ISL_887474, EPI_ISL_887475, EPI_ISL_887478, EPI_ISL_887479, EPI_ISL_887481, EPI_ISL_887484, EPI_ISL_887487, EPI_ISL_887489, EPI_ISL_887490, EPI_ISL_887491, EPI_ISL_887495, EPI_ISL_887498, EPI_ISL_887499, EPI_ISL_887500, EPI_ISL_887502, EPI_ISL_887503, EPI_ISL_964922, EPI_ISL_964930, EPI_ISL_964932, EPI_ISL_964936, EPI_ISL_964939, EPI_ISL_964940			
see above	Instituto Nacional de Saude (INS), Mozambique	KRISP, KZN Research Innovation and Sequencing Platform	Emmanuel S; Giandhari J; Nadia Sitoe; Nalia Ismael; Nedio Mabunda; Paulo Arnaldo; Pillay S; Tegally H; Wilkinson E; de Oliveira T
EPI_ISL_576258, EPI_ISL_576276, EPI_ISL_658883, EPI_ISL_872089	Instituto de Diagnostico y Referencia Epidemiologicos (INDRE)	Instituto de Diagnostico y Referencia Epidemiologicos (INDRE)	Abril Rodriguez-Maldonado; Adnan Araiza-Rodriguez; Adnan-Araiza Rodríguez; Claudia Wong-Arambula; Dayanira Arellano-Suarez; Edgar Mendieta-Condado; Ernesto Ramirez-Gonzalez; Ernesto Ramirez-Gonzalez.; Fabiola Garces-Ayala; Gisela Barrera-Badillo; Gisela Barrera-Badillo.; Irma Lopez-Martinez; Lucia Hernandez-Rivas; Natividad Cruz-Ortiz; Tatiana Nunez-Garcia

EPI_ISL_1060728, EPI_ISL_1060747, EPI_ISL_1060751	Instituto de Diagnostico y Referencia Epidemiologicos (INDRE)_RNLSP	Instituto de Diagnostico y Referencia Epidemiologicos (INDRE)	Abril Rodriguez-Maldonado; Adnan Araiza-Rodriguez; Claudia Wong-Arambula; David Fragoso-Fonseca; Ernesto Ramirez-Gonzalez.; Fabiola Garces-Ayala; Gisela Barrera-Badillo; Irma Lopez-Martinez; Lucia Hernandez-Rivas; Mayra Jimenez-Morales; Nancy Munoz-Hernandez; Natividad Cruz-Ortiz; Sergio Rangel-Guerrero; Tatiana Nunez-Garcia
EPI_ISL_913921, EPI_ISL_913924, EPI_ISL_913929, EPI_ISL_933668, EPI_ISL_1054962, EPI_ISL_1054963, EPI_ISL_1054982, EPI_ISL_1168472, EPI_ISL_1168524, EPI_ISL_1168560			
see above	Instituto de Diagnostico y Referencia Epidemiologicos INDRE_RNLSP	Instituto de Diagnostico y Referencia Epidemiologicos (INDRE)	Abril Rodriguez-Maldonado; Adnan Araiza-Rodriguez; Ariadna Medina-Benitez; Claudia Wong-Arambula; David Fragoso-Fonseca; Ernesto Ramirez-Gonzalez.; Fabiola Garces-Ayala; Gisela Barrera-Badillo; Irma Lopez-Martinez; Joaquin Quiroz-Mercado; Lucia Hernandez-Rivas; Mayra Jimenez-Morales; Nancy Munoz-Hernandez; Natividad Cruz-Ortiz; Sergio Rangel-Guerrero; Tatiana Nunez-Garcia; Vanessa Rivero-Arredondo
EPI_ISL_1301690, EPI_ISL_1301698	Instituto de Diagnostico y Referencia Epidemiologicos InDRE_RNLSP	Instituto de Biotecnología de la UNAM	Alejandra Hernández-Terán; Alejandro Sanchez-Flores; Alma Rincón-Rubio; Andrea Santos Coy-Arechavaleta; Authors from IBT; Blanca Taboada; Celia Boukadida; Clara Esperanza Santacruz-Tinoco; Edgar Mendieta-Condado; Eduardo Becerril-Vargas; Fidencio Mejía-Nepomuceno; Francisco Pulido; Gisela Barrera-Badillo; Gloria Vazquez; Hector Esteban Paz-Juárez; IMSS; InDRE and INER (in alphabetical order): Carlos F. Arias; Irma Lopez-Martinez; Jerome Jean Verleyen; Joel Armando Vázquez-Pérez; Jorge Salas-Hernández; José Arturo Martínez-Orozco; José Ernesto Ramírez-González; José Esteban Muñoz-Medina; Larissa Fernandes-Matano; Lucia Hernandez-Rivas; Luis Alberto Ochoa-Carrera; Margarita Matías-Florentino; Mario Mújica-Sánchez; Natividad Cruz-Ortiz; Pavel Isa; Ricardo Grande; Santiago Ávila-Ríos; Tatiana Nunez-Garcia; Teresita Rojas-Mendoza
EPI_ISL_1395782, EPI_ISL_1395783, EPI_ISL_1395785	Instituto de Investigaciones Biomédicas en Retrovirus y SIDA (INBIRS)	Área de Secuenciación del Laboratorio de Virología del Hospital de Niños Dr. Ricardo Gutierrez on behalf of 'Proyecto Argentino Interinstitucional de genomica de SARS-CoV-2' (PAIS Consortium)	Acuña; Alexay; D; Federico Remes Lenicov; Goya; Horacio Salomón; LE; Lusso; M; MI; Nabaes Jodar; Natale; S; Valinotto; Vanesa Seery; Viegas, M.
EPI_ISL_1652066	Integrated Biorepository of H3Africa Uganda – IBRH3AU	Molecular Biology Laboratory	Ashaba Fred Katabazi; Bernard Ssentalo Bagaya; David Patrick Kateete; Edgar Kigozi; Emmanuel Nasinghe; Eric Katagirya; Gerald Mboowa; Lwanga Newton; Misaki Wayengera; Moses Joloba; Moses Luutu; Nsubuga Gideon; Rogers Kamulegeya; Samuel Kirimunda; Sarah Stanley; Savannah Mwesigwa
EPI_ISL_1167131, EPI_ISL_1167133, EPI_ISL_1167134, EPI_ISL_1167135, EPI_ISL_1167136, EPI_ISL_1167140, EPI_ISL_1167144, EPI_ISL_1167145, EPI_ISL_1167146, EPI_ISL_1167150, EPI_ISL_1167152, EPI_ISL_1167153, EPI_ISL_1167154, EPI_ISL_1167155, EPI_ISL_1167158, EPI_ISL_1167160, EPI_ISL_1167161, EPI_ISL_1167164, EPI_ISL_1167167, EPI_ISL_1167168, EPI_ISL_1167169, EPI_ISL_1167171, EPI_ISL_1167174, EPI_ISL_1167176, EPI_ISL_1167180, EPI_ISL_1167182, EPI_ISL_1167184			
see above	Iressef Genomics lab	L'institut de Recherche en Santé, de Surveillance Épidémiologique et de Formation (IRESSEF)	Abdou PADANE; Abdoulie KANTEH; Abdul Karim SESAY; Ambroise AHOUIDI; Aminata DIA; Aminata MBOUP; Astou Gaye GAYE; Barada CISSE; Birahim Piere NDIAYE; Gora LO; Khadim GUEYE; Moustapha MBOW; Nafisatou LEYE; Ndeye Coumba Toure KANE; Papa Alassane DIAW; Souleymane MBOUP; Yacine DIA
EPI_ISL_514283, EPI_ISL_514293, EPI_ISL_516885, EPI_ISL_516894, EPI_ISL_516897, EPI_ISL_745072, EPI_ISL_745084			
see above	Israel Central Virology laboratory	Israel Central Virology laboratory	Efrat Dahan Bucris; Ella Mendelson; Michal Mandelboim; Neta Zuckerman; Oran Erster; Orna Mor
EPI_ISL_889051, EPI_ISL_889144, EPI_ISL_944317, EPI_ISL_1209802, EPI_ISL_1210342, EPI_ISL_1358598	Israel Central Virology laboratory	Israel National Consortium for SARS-CoV-2 sequencing	Assaf Rokney; Dana Bar-Ilan; David A. Zeevi; Efrat Dahan Bucris; Efrat Glick-Saar; Efrat Rorman; Ella Mendelson; Ephraim Fass; Eva Nachum; Gal Zizelski Valenci; Gideon Rechavi; Israel Nissan; Joseph Jaffe; Maya Davidovich Cohen; Michal Mandelboim; Mor Rubinstein; Neta Zuckerman; Omer Murik; Omri Nayshool; Oran Erster; Orna Mor; Tzvia Mann
EPI_ISL_1424345, EPI_ISL_1424371, EPI_ISL_1629916	Istituto Zooprofilattico Sperimentale del Mezzogiorno	TIGEM	Andrea Ballabio; Anna Manfredi; Antonio Grimaldi; Antonio Grimaldi Patrizia Annunziata Francesco Panariello Biancamaria Pierri Claudia Tiberio Teresa Giuliano Valentina Bouche Chiara Colantuono Maria Concetta Cuomo Denise Di Concilio Lucio Di Filippo Anna Manfredi Marcello Salvi Antonio Limone Luigi Atripaldi Pellegrino Cerino Andrea Ballabio Davide Cacchiarelli; Antonio Limone Luigi Atripaldi Pellegrino Cerino; Biancamaria Pierri Claudia Tiberio Valentina Bouche; Chiara Colantuono; Davide Cacchiarelli; Denise Di Concilio; Francesco Panariello; Lucio Di Filippo; Marcello Salvi; Maria Concetta Cuomo; Patrizia Annunziata
EPI_ISL_1254794	Istituto Zooprofilattico Sperimentale del Mezzogiorno	Telethon Institute of Genetics and Medicine (TIGEM)	Antonio Grimaldi Patrizia Annunziata Francesco Panariello Biancamaria Pierri Claudia Tiberio Valentina Bouche Chiara Colantuono Maria Concetta Cuomo Denise Di Concilio Lucio Di Filippo Anna Manfredi Marcello Salvi Antonio Limone Luigi Atripaldi Pellegrino Cerino Andrea Ballabio Davide Cacchiarelli
EPI_ISL_1298477	Ivano-Frankivsk CCH#1	The Institute of Molecular Biology and Genetics of NASU	M.Tukalo et al.
EPI_ISL_1624950	J.W. Ruby Memorial Hospital	WVU and Marshall University Combined Genomics Core Facilities	James Denvir; Peter Perrotta; Peter Stoilov; Ryan Percifield; Wesley Kimble
EPI_ISL_779265	Jamil-ur-Rahman Center for Genome Research, Dr. Panjwani Center for Molecular Medicine and Drug Research	Jamil-ur-Rahman Center for Genome Research, Dr. Panjwani Center for Molecular Medicine and Drug Research	Ansari, S.; Irfan, M.; Khan, I.; Nisa, Z.; Rashid, M.; Shakeel, M.
EPI_ISL_451958	Jamil-ur-Rahman Center for Genome Research, Dr. Panjwani Center for Molecular Medicine and Drug Research, International Center for Chemical and Biological Sciences, University of Karachi	Jamil-ur-Rahman Center for Genome Research, Dr. Panjwani Center for Molecular Medicine and Drug Research, International Center for Chemical and Biological Sciences, University of Karachi	B.A.; I.A; Khan; Khan, S.; M.A.and Khan; Qureshi; Raza; S.A.; Shakeel, M.; Zahid, M.
EPI_ISL_1577775, EPI_ISL_1607982, EPI_ISL_1607988	Jessa	Jessa	Cruys et al. on behalf of the Jessa_cmdLab
EPI_ISL_421254	Jiangxi Province Center for Disease Control and Prevention	Jiangxi Province Center for Disease Control and Prevention	DaJin Xiao; Fang Xiao; Gang Xu; JianXiong Li; Jun Zhou; ShiWen Liu; Tian Gong; XiaoQing Liu; Xin Ran; YanNi Zhang; Ying Xiong; Yong Shi
EPI_ISL_1610613	KEMRI Center for Biotechnology Research and Development	KEMRI-Wellcome Trust Research Programme,Kilifi	Githinji G.; Matoke D.; Mburu M.W.; Mohamed K.S.; Thiongo K.; de Laurent Z.
EPI_ISL_457854, EPI_ISL_457868, EPI_ISL_457875, EPI_ISL_457884, EPI_ISL_457915, EPI_ISL_457920	KEMRI-CGMR-C	KEMRI-Wellcome Trust Research Programme/KEMRI-CGMR-C Kilifi	Githinji G. et al 2020
EPI_ISL_1440119	KEMRI-Wellcome Trust Research Programme,Kilifi	KEMRI-Wellcome Trust Research Programme,Kilifi	Githinji G.; Mburu M.W.; Mohamed K.S.; deLaurent Z.
EPI_ISL_568725, EPI_ISL_568727, EPI_ISL_568735, EPI_ISL_568764, EPI_ISL_568872	KEMRI-Wellcome Trust Research Programme/KEMRI-CGMR-C Kilifi	KEMRI-Wellcome Trust Research Programme/KEMRI-CGMR-C Kilifi	Githinji et al 2020
EPI_ISL_436686, EPI_ISL_455631, EPI_ISL_455632, EPI_ISL_455636, EPI_ISL_455639, EPI_ISL_660221, EPI_ISL_660232, EPI_ISL_660233			
see above	KRISP, KZN Research Innovation and Sequencing Platform	KRISP, KZN Research Innovation and Sequencing Platform	Chimukangara B; Deforche K; Giandhari J; Khan S; Lessells R; Mdlalose K; Pillay S; Tegally H; Wilkinson E; York D; de Oliveira T
EPI_ISL_888773, EPI_ISL_995629, EPI_ISL_1057093, EPI_ISL_1312210	KU Leuven, Rega Institute, Clinical and Epidemiological Virology	KU Leuven, Rega Institute, Clinical and Epidemiological Virology	Bert Vanmechelen; Joan Marti-Carerras; Piet Maes; Tony Wawina-Bokalanga
EPI_ISL_1094345	KY State Public Health Lab	Respiratory Viruses Branch, Division of Viral Diseases, Centers for Disease Control and Prevention	Anna Montmayeur; Anna Uehara; Ben L. Rambo-Martin; Clinton R. Paden; Dhwani Batra; Haibin Wang; Jasmine Padilla; Jing Zhang; Justin Lee; Krista Queen; Lori Rowe; Mark Burroughs; Mili Sheth; Peter W. Cook; Rachel Marine; Sarah Nobles; Suxiang Tong; Yan Li; Ying Tao
EPI_ISL_1054890	Kansas Health and Environmental Lab	Kansas Health and Environmental Lab	Ben Olsen; Carissa Robertson; Mike Grose; Paige Drury; and Phil Adam
EPI_ISL_2233093, EPI_ISL_2233097	Kantor Kesehatan Pelabuhan Kelas II Cilacap	National Institute of Health Research and Development	Arie Ardiansyah Nugraha; Hana Apsari Pawestri; Hartanti Dian Ikawati; Kartika Dewi Puspa; Krisna Pangesti; Nelly Puspandari; Subangkit; Triyani Soekarso; Vivi Setiawaty
EPI_ISL_475544, EPI_ISL_510862	Karolinska Universitetslaboratoriet	The Public Health Agency of Sweden	Anna Risberg; Anna-Malin Linde; Karin Tegmark-Wisell; Maria Lind Karlberg; Mattias Haukland; Mia Brytting; Olov Svartstrom; Oskar Karlsson Lindsjo; Petra Edquist; Reza Advani; Sandra Broddesson; Shaman Muradrasoli
EPI_ISL_512811, EPI_ISL_512813, EPI_ISL_512815, EPI_ISL_512816, EPI_ISL_512817, EPI_ISL_512818, EPI_ISL_512820, EPI_ISL_512821			
see above	Kenema Government Hospital, Ministry of Health and Sanitation	Kenema Government Hospital, Ministry of Health and Sanitation	Andersen, K.; Garry, R.; Goba, A.; Grant, D.; Happi, C.; Jalloh, S.; Mehta, S.; Momoh, M.; Olawoye, I.; Oluniyi, P.; Park, D.; Sandi, J.; Siddle, K.; Tomkins-Tinch, C.
EPI_ISL_490010	King Fahad Medical City	King Fahad Medical City	Alghoraibi, M.; Alosaimi, B.; Enani, M.; Naeem, A.
EPI_ISL_483544, EPI_ISL_483545, EPI_ISL_483547, EPI_ISL_483551	Kingdom of Bahrein Ministry of Health	Erasmus Medical Center	Amjad Ghanem Mohamed; Anne van der Linden; Bas Oude Munnink; Claudia Schapendonk; David Nieuwenhuijse; Ebrahim Shehad; Fatema; Hashmeya Al Wasti; Irina Chestakova; Marion Koopmans; Mark Pronk; Pascal Lexmond; Reina Sikkema; Richard Molenkamp; Stefan van Nieuwkoop; Theo Bestebroer; on behalf of the Dutch national COVID-19 response team.
EPI_ISL_717750	Kingston Health Sciences Centre and Queen's University	Ontario Institute for Cancer Research	Bernard Lam; Calvin Sjaarda; Ilinca Lungu; Jared T. Simpson; Katya Douchant; Lawrence E. Heisler; Michael Laszloffy; Paul Krzyzanowski; Prameet M. Sheth; Richard de Borja; Robert Colautti
EPI_ISL_1259313	Klinisch Laboratorium ZNA	Klinisch Laboratorium ZNA	Verstrepen et al.
EPI_ISL_700450, EPI_ISL_700526	Knysna CDC wc WLC	NHLS/UCT	Arash Iranzadeh; Bruna Galvao; Carolyn Williamson; Deelan Doolabh; Diana Hardie; Houriiyah Tegally; Innocent Mudau; Kruger Marais; Lynn Tyers; Marvin Hsiao; Stephen Korsman
EPI_ISL_640022, EPI_ISL_700429, EPI_ISL_700509	Knysna Hospital wc KNY	NHLS/UCT	Arash Iranzadeh; Bruna Galvao; Carolyn Williamson; Deelan Doolabh; Diana Hardie; Houriiyah Tegally; Innocent Mudau; Kruger Marais; Lynn Tyers; Marvin Hsiao; Stephen Korsman
EPI_ISL_1298475, EPI_ISL_1298479	Kosov CRH	The Institute of Molecular Biology and Genetics of NASU	M.Tukalo et al.
EPI_ISL_515181, EPI_ISL_515182	Kumasi Centre for Collaborative Research in Tropical Medicine, Kumasi.	Institute of Virology, Charité – Universitätsmedizin Berlin	Augustina Sylverken; Christian Drosten; Eric Adu; Jesse Addo Asamoah; Julia Schneider; Jörn Beheim-Schwarzbach; Michael Owusu; Philip El-Duah; Richard Phillips.; Richmond Gorman; Richmond Yeboah; Sherihane Aryeetey; Victor Max Corman
EPI_ISL_700532	Kwamandlenkosi Clinic wc KMN	NHLS/UCT	Arash Iranzadeh; Bruna Galvao; Carolyn Williamson; Deelan Doolabh; Diana Hardie; Innocent Mudau; Kruger Marais; Lynn Tyers; Marvin Hsiao; Stephen Korsman
EPI_ISL_700495,	Kwanokuthula CDC wc KWA	NHLS/UCT	Arash Iranzadeh; Bruna Galvao; Carolyn Williamson; Deelan Doolabh; Diana Hardie; Innocent Mudau; Kruger Marais; Lynn Tyers; Marvin Hsiao; Stephen Korsman

EPI_ISL_700537			
EPI_ISL_914832, EPI_ISL_1067620, EPI_ISL_1196432	LABORATORIO CLINICO LABIN	Inciensa, Instituto Costarricense de Investigación y Enseñanza en Nutrición y Salud	Adriana Godínez; Caterina Guzmán; Claudio Soto-Garita; Estela Cordero; Francisco Duarte; Hebleen Porras; Melany Calderón; Melany Calderón & David Rodríguez-Masís; Melany Calderón & Pei Ling Chan Ma; Nazareth Ruiz & Pei Ling Chan Ma
EPI_ISL_918556	LACEN - Laboratório Central de Saúde Pública do Amapa	Evandro Chagas Institute	A.M.; Barbagelata; E.C.; E.M.A.; Ferreira; J.A.; Junior; K.C.; L.C.; L.S.; M.C.; P.S.; Pinheiro; Santos; Silva; Sousa; Sousa Junior; W.D.C.; da Silva
EPI_ISL_925916	LACEN - Laboratório Central de Saúde Pública do Amazonas	Evandro Chagas Institute Virology	A.M.; Barbagelata; E.C.; E.M.A.; Ferreira; J.A.; Junior; K.C.; L.C.; L.S.; M.C.; P.S.; Pinheiro; Santos; Silva; Sousa; Sousa Junior; W.D.C.; da Silva
EPI_ISL_918503	LACEN - Laboratório Central de Saúde Pública do Amazonas	Evandro Chagas Institute	A.M.; Barbagelata; E.C.; E.M.A.; Ferreira; J.A.; Junior; K.C.; L.C.; L.S.; M.C.; P.S.; Pinheiro; Santos; Silva; Sousa; Sousa Junior; W.D.C.; da Silva
EPI_ISL_1293051	LACEN do Distrito Federal	Instituto Adolfo Lutz, Interdiciplinary Procedures Center, Strategic Laboratory	Caio Vinicius Dias Lopes; Claudia Regina Gonçalves; Claudio Tavares Sacchi; Erica Valessa Ramos Gomes; Karoline Rodrigues Campos
EPI_ISL_1468432	LACEN do Mato Grosso do Sul	Instituto Adolfo Lutz, Interdiciplinary Procedures Center, Strategic Laboratory	Caio Vinicius Dias Lopes; Claudia Regina Gonçalves; Claudio Tavares Sacchi; Erica Valessa Ramos Gomes; Karoline Rodrigues Campos
EPI_ISL_1213222	LAFEM/UESC	Bioinformatics Laboratory / LNCC	Alessandra P Lamarca; Alexandra L Gerber; Ana Paula Melo Mariano; Ana Paula de C Guimarães; Ana Tereza R Vasconcelos; Angela Maria Guimarães Santos; Bianca Mendes Maciel; Danielle Angst Secco; Eduardo Sérgio Soares Sousa; Eloiza Helena Campana; Francisco Paulo Freire Neto; George Rego Albuquerque; Kátia Castanho Scortecci; Lucymara Fassarella Agnez Lima; Luiz G P de Almeida; Luís Cristóvão Porto; Otavio J. Brustolini; Paulo Ricardo Nascimento; Ronaldo da Silva Francisco Jr; Sandra Rocha Gadelha; Selma Maria Bezerra Jeronimo; Vinícius Pietta Perez
EPI_ISL_1213201	LBM/UFPB	Bioinformatics Laboratory / LNCC	Alessandra P Lamarca; Alexandra L Gerber; Ana Paula Melo Mariano; Ana Paula de C Guimarães; Ana Tereza R Vasconcelos; Angela Maria Guimarães Santos; Bianca Mendes Maciel; Danielle Angst Secco; Eduardo Sérgio Soares Sousa; Eloiza Helena Campana; Francisco Paulo Freire Neto; George Rego Albuquerque; Kátia Castanho Scortecci; Lucymara Fassarella Agnez Lima; Luiz G P de Almeida; Luís Cristóvão Porto; Otavio J. Brustolini; Paulo Ricardo Nascimento; Ronaldo da Silva Francisco Jr; Sandra Rocha Gadelha; Selma Maria Bezerra Jeronimo; Vinícius Pietta Perez
EPI_ISL_1626809	LESP Guanajuato	Instituto de Diagnostico y Referencia Epidemiologicos (INDRE)	Abril Rodriguez-Maldonado; Ariadna Medina-Benitez; Claudia Wong-Arambula; Ernesto Ramirez-Gonzalez.; Gisela Barrera-Badillo; Irma Lopez-Martinez; Joaquin Quiroz-Mercado; Lucia Hernandez-Rivas; Natividad Cruz-Ortiz; Sergio Rangel-Guerrero; Tatiana Nunez-Garcia; Vanessa Rivero-Arredondo
EPI_ISL_1558830	LESP Jalisco/UdeG	Instituto de Diagnostico y Referencia Epidemiologicos (INDRE)	Abril Rodriguez-Maldonado; Ariadna Medina-Benitez; Claudia Wong-Arambula; Ernesto Ramirez-Gonzalez.; Gisela Barrera-Badillo; Irma Lopez-Martinez; Joaquin Quiroz-Mercado; Lucia Hernandez-Rivas; Natividad Cruz-Ortiz; Sergio Rangel-Guerrero; Tatiana Nunez-Garcia; Vanessa Rivero-Arredondo
EPI_ISL_1399277	LESP Tamaulipas	Instituto de Diagnostico y Referencia Epidemiologicos (INDRE)	Abril Rodriguez-Maldonado; Ariadna Medina-Benitez; Claudia Wong-Arambula; Ernesto Ramirez-Gonzalez.; Gisela Barrera-Badillo; Irma Lopez-Martinez; Joaquin Quiroz-Mercado; Lucia Hernandez-Rivas; Natividad Cruz-Ortiz; Sergio Rangel-Guerrero; Tatiana Nunez-Garcia; Vanessa Rivero-Arredondo
EPI_ISL_1219155, EPI_ISL_1219578	LIC	Latvian Biomedical Research and Study Centre	Davids Fridmanis; Diana Dusacka; Guntars Zarins; Ivars Silamikelis; Janis Klovins; Janis Pjalkovskis; Jurijs Perevoscikovs; Kaspars Megnis; Laila Silamikele; Lauma Freimane; Laura Ansone; Liga Birzniece; Monta Ustinova; Nikita Zrelovs; Reinis Zeltmatis; Uga Dumpis; Una Krumina; Vita Rovite
EPI_ISL_469049, EPI_ISL_469052, EPI_ISL_469054	LNR National Reference Laboratory, Mohammed VI University of Health Sciences	Medical Biotechnology Laboratory, Rabat Medical and Pharmacy School, Mohammed The Vth University in Rabat	Chakib NEJJARI; Houda BENRAHMA; Idrissa Diawara; Imane SMYEJ; Jalil El Atar; Jalila RAHOUI; Lahcen BELYAMANI and Azeddine IBRAHIMI; Laila SBABOU; Loubna ALLAM; M.W. CHEMAO-ELFIHRI; Meriem LAAMARTI; Mouna OUADGHIRI; Rachid EL JAOUDI; Rachid MENTAG; Rokaia LAAMRTI; Saaid AMZAZI; Souad KARTTI
EPI_ISL_451243, EPI_ISL_768502, EPI_ISL_889488, EPI_ISL_1324372	LSUHS Emerging Viral Threat Laboratory	Microbial Genome Sequencing Center	Abida Siddiqa; Adam Greer; Andrew D. Yurochko; Byeong-Jae Lee; Camille F. Abshire; Chan-ki Min; Christopher G. Kevil; Daniel J. Snyder; Edna Ondari; Jason M. Bodily; Jennifer L. Carroll; Jeremy P. Kamil; John A. Vanchiere; Katarzyna Zwolinska; Maarten Van Diest; Malgorzata Bienkowska-Haba; Martin J. Sapp; Md Maksudul Alam; Mohammed N.A. Siddiquey; Monica Gestal-Cartele; Paul M. Weinberger; Rona S. Scott; Vaughn S. Cooper
EPI_ISL_717998, EPI_ISL_855916	Lab voor klinische biologie	Onderzoeksgroep Virologie	Bruno Verhasselt; Hans Nauwynck; Laurens Lambrechts; Linos Vandekerckhove; Marthe Pauwels; Nick Vereecke; Sebastiaan Theuns
EPI_ISL_547969, EPI_ISL_579106, EPI_ISL_707804, EPI_ISL_755629, EPI_ISL_755630, EPI_ISL_755637, EPI_ISL_794616, EPI_ISL_794617, EPI_ISL_794620, EPI_ISL_794623, EPI_ISL_1016858, EPI_ISL_1172028, EPI_ISL_1250704, EPI_ISL_1469093, EPI_ISL_1621323			
see above	LabPLUS	Institute of Environmental Science and Research (ESR)	Anja Werno; Antje van der Linden; Arlo Upton; Chris Mansell; David Hammer; Dragana Drinkovic; Erasmus Smit; Gary McAuliffe; Hana Sofia Andersson; Hermes Perez; James Ussher; Jill Sherwood; Jing Wang; Joep de Ligt; Josh Freeman; Julia Howard; Juliet Elvy; Lauren Jelly; Mary DeAlmeida; Matt Blakiston; Matt Storey; Matthew Rogers; Max Bloomfield; Michael Addidle; Michelle Balm; Muhammad Faisal; Nikki Freed; Olin Silander; Olivia Stroeven; Rachel Boyle; Sally Roberts; SallyAnn Harbison; Sarah Jefferies; Sharmini Muttaiyah; Susan Morpeth; Susan Taylor; Timothy Blackmore; Vani Sathyendran; Veronica Playle; Virginia Hope; Xiaoyun Ren
EPI_ISL_886232, EPI_ISL_886293, EPI_ISL_886313, EPI_ISL_886375, EPI_ISL_888215, EPI_ISL_888471	Labcorp	Genomics and Discovery, Respiratory Viruses Branch, Division of Viral Diseases, Centers for Disease Control and Prevention	; Amanda Douglas; Amanda Suchanek; Andrea Throop; Ayla Burns; Ben L. Rambo-Martin; Bobbi Croy; Brian Krueger; Brian Norvell; Christos Petropoulos; Clinton R. Paden; Craig Lukasik; Debbie Boles; Dhwani Batra; Duncan MacCannell; Eyad Almasri; Goran Stevovic; Howard Engler; Hrushikesh Deshmukh; Jake Humphrey; Jana Schroth; Joe Voshell; John Pruitt; Jonathan Meltzer; Jonathan Williams; Kimberly Wagner; Lax Iyer; Lyndon Tilson; Manoj Jain; Marcia Eisenberg; Mary Ann Cristobal; Mary Williamson; Michael Levandoski; Mike Sapeta; Mindy Nye; Minoo Agarwal; Mohan Kolli; Nuthawin Charoensri; Oren Cohen; Peter W. Cook; Prashant Gupta; Qian Zeng; Rama Ghatti; Scott Parker; Scott Ryan; Stanley Letovsky; Steven Ragan; Summer Galloway; Suresh Babu Selvaraju; Susan Countryman; Susan Hicks; Suxiang Tong; Suzanne Dale; Thomas Urban; Tim Kuphal; Tricia Zwiefelhofer; Vincent Drouillon
EPI_ISL_1219949	Labo Analyses Med	National Reference Center for Viruses of Respiratory Infections, Institut Pasteur, Paris	Angela Brisebarre; Camille Capel; Durivault JéRôMe; Etienne Simon-Lorière; Marion Barbet; Maud Vanpeene; Méline Bizard; Sylvie Behillil; Sylvie van der Werf; Vincent Enouf
EPI_ISL_1140256	Labor ZOTZ|KLIMAS; MVZ Düsseldorf-Centrum	Robert Koch Institute	
EPI_ISL_768840, EPI_ISL_775220, EPI_ISL_775221, EPI_ISL_775225, EPI_ISL_775256, EPI_ISL_775260, EPI_ISL_775262, EPI_ISL_775263			
see above	Laboratoire Biolife	Laboratoire de Biotechnologie	Afaf Alaoui; Amina Benouda; Bouchra Belfquih; Hanae Dakka; Lahcen belyamani; Mohamed Chenaoui; Mohammed Walid Chemao Elfihri; Mouna Ouadghiri; Otmane Touzani; Saaid Amzazi and Azeddine Ibrahimi; Tarik Aanniz
EPI_ISL_1502567, EPI_ISL_1508991, EPI_ISL_1524720	Laboratoire Biolim/FSS/UL	Unité Mixte Internationale TransVIHMI (UMI 233 IRD – U1175 INSERM - Université de Montpellier) IRD (Institut de recherche pour le développement)	Abla A. KONOU; Adodo SADJI; Ahidjo AYOUBA; Akoélé SILIADIN; Alassane OURO-MEDELI; Amivi EHLAN; Améyo DORKENOO; Anoumou DAGNRA; Christelle BUTEL; Déléma MABA; Eric DELAPORTE; Issaka Maman; Kokou TEGUENI; Koku AGBODEKA; Laetitia SERRANO; Martine PEETERS; Messanh DOUFFAN; Mireille PRINCE-DAVID; Mounerou SALOU; Sidonie A.M.KAGNISSODE; Sika DOSSIM; Syntiche DEVATCHAGNI Adodo SADJI; Syntyche DEVATCHAGNI; Wembo A. HALATOKO
EPI_ISL_1103576, EPI_ISL_1109622, EPI_ISL_1116470, EPI_ISL_1116592, EPI_ISL_1120732, EPI_ISL_1137621, EPI_ISL_1159697, EPI_ISL_1159698, EPI_ISL_1159699			
see above	Laboratoire central de Virologie	Laboratoire de Biotechnologie	Abdelomunim Essabbar; Amal Zouaki; Ghizlane EL Amin; Hakima Kabbaj; Lahcen Belyamani and Azeddine Ibrahim; Lahcen Belyamani and Azeddine Ibrahimi; Mouna Ouadghiri; Myriam Seffar; Saaïd Amzazi; Tarik Aanniz
EPI_ISL_1103584	Laboratoire central de Virologie, Centre hospitalo-universitaire Ibn Sina, Hôpital des spécialités	Laboratoire de Biotechnologie	Abdelomunim Essabbar; Amal Zouaki; Ghizlane EL Amin; Hakima Kabbaj; Lahcen Belyamani and Azeddine Ibrahimi; Mouna Ouadghiri; Myriam Seffar; Saaïd Amzazi; Tarik Aanniz
EPI_ISL_660449, EPI_ISL_660450, EPI_ISL_660451, EPI_ISL_660453, EPI_ISL_660456, EPI_ISL_660460, EPI_ISL_660465, EPI_ISL_660466, EPI_ISL_660473, EPI_ISL_660475, EPI_ISL_660476, EPI_ISL_660478, EPI_ISL_660480, EPI_ISL_660481, EPI_ISL_660482, EPI_ISL_660483, EPI_ISL_660490, EPI_ISL_660492			
see above	Laboratoire de Microbiologie CHU Souro Sanou	Centre Muraz	Abdoul-Salam Ouedraogo; Arsène Zongo; Essia Belarbi; Fabian Leendertz; Grit Schubert; Halidou Tinto; Lassana Sangaré; Soumeya Ouangraoua; Yacouba Sawadogo; Zekiba Tarnagda
EPI_ISL_1116467, EPI_ISL_1116468, EPI_ISL_1118675, EPI_ISL_1118923, EPI_ISL_1138747	Laboratoire de Microbiologie- CHU Habib Bourguiba – Sfax	Laboratoire des Procédés de Criblage Moléculaire et Cellulaire-Centre de Biotechnologie de Sfax	A. and Masmoudi, S.; Abdelmoulah, F.; Abid, N.; Ajili, F.; Aouni, M.; Ben Ayed, I.; Bensaid, M.; Chtourou, A.; Elargoubi, A.; Fki-berrajah, L.; Gaaloul, I.; Gargouri, S.; Hammami, A.; Kamoun, S.; Karray Hakim, H.; Kharat, N.; Mastouri, M.; Mhalla, S.; Nabli, A.; Rebai; Smeti, I.; Souissi, A.; Stambouli, N.; Turki, M.
EPI_ISL_712065, EPI_ISL_712066	Laboratoire de Microbiologie- CHU Habib Bourguiba – Sfax adresse	Laboratoire des Procédés de Criblage Moléculaire et Cellulaire-Centre de Biotechnologie de Sfax	A. and Masmoudi, S.; Abdelmoulah, F.; Abid, N.; Ajili, F.; Aouni, M.; Ben Ayed, I.; Bensaid, M.; Chtourou, A.; Elargoubi, A.; Fki-berrajah, L.; Gaaloul, I.; Gargouri, S.; Hammami, A.; Kamoun, S.; Karray Hakim, H.; Kharat, N.; Mastouri, M.; Mhalla, S.; Nabli, A.; Rebai; Smeti, I.; Souissi, A.; Stambouli, N.; Turki, M.
EPI_ISL_476024, EPI_ISL_476025, EPI_ISL_476026	Laboratoire de Recherche et d'Analyses Médicales de la Gendarmerie Royale	Laboratoire de Recherche et d'Analyses Médicales de la Gendarmerie Royale	Amal SOUIRI; Amal Souiri; Ayoub Jibjibe; Mahmoud Yartaoui; Marouane El Rhouila; Mohamed Chahmi; Mohamed Labioui; Nabil Lemzaoui; Nabil Ouaariba; Nadia Kandoussi; Omar Mestoui; Samiha Sellak; Sanaâ LEMRISS; Sanaâ Lemriss; Saâd EL KABBAJ; Saâd El Kabbaj.
EPI_ISL_414020	Laboratoire de Virologie, HUG	Swiss National Reference Centre for Influenza	LAUBSCHER Florian et al.
EPI_ISL_825785, EPI_ISL_1400716, EPI_ISL_1523371, EPI_ISL_1523393	Laboratoire de santé publique du Québec	Laboratoire de santé publique du Québec	Guillaume Bourque; Ioannis Ragoussis; Jesse Shapiro; Mark Lathrop and Michel Roger on behalf of the CoVSeQ research group (http://covseq.ca/researchgroup); Sandrine Moreira
EPI_ISL_1197037	Laboratoire de virologie clinique - Institut Pasteur de Tunis	1-Laboratory of Microbiology, National Reference Lab, Charles Nicolle Hospital; 2-University of Tunis ElManar, Faculty of Medicine of Tunis, LR99ES09, Tunis, Tunisia	Alia BenKahla; Anissa Chouikha; Fares Wasfi; Henda Triki; Ilhem Boutiba-Ben Boubaker.; Imen Kacem; Ines Mdini; Jalila Ben Khelil; Maher Kharrat; Manel Ben Sassi; Mariem Gdoura; Mouna Ben Sassi; Mouna Safer; Nissaf Ben Alaya; Riadh Daghfous; Riadh Gouider; Roua Ben Othman; Salma Abid; Sameh Trabelsi; Sana Ferjani; Sara Chammam; Sondes Haddad
EPI_ISL_476823, EPI_ISL_476825, EPI_ISL_476826, EPI_ISL_476830, EPI_ISL_476831, EPI_ISL_476833	Laboratoire des Fièvres Hémorragiques Virales du Benin	Charité-Universitätsmedizin Berlin	Anges; Drexler; Jan Felix; Moreira-Soto Andres; Sander Anna-Lena; Yadouleton
EPI_ISL_910517, EPI_ISL_910580, EPI_ISL_910753, EPI_ISL_910823, EPI_ISL_911128, EPI_ISL_1383368	Laboratoire national de sante, Microbiology, Virology	Laboratoire national de sante, Microbiology, Microbial Genomics Platform	Anke Wienecke-Baldacchino; Catherine Ragimbeau; Fatu Djabi; Jessica Tapp; Lise Pignon; Raoul Salmon; Tamir Abdelrahman; Trung Nguyen Nguyen
EPI_ISL_740229, EPI_ISL_770889, EPI_ISL_771057	Laboratoire national de santé, Microbiology, Virology	Laboratoire national de santé, Microbiology, Microbial Genomics Platform	Anke Wienecke-Baldacchino; Catherine Ragimbeau; Fatu Djabi; Jessica Tapp; Lise Pignon; Raoul Salmon; Tamir Abdelrahman

EPI_ISL_1384363	Laboratoires d'analyses medicales - Ketterthill	Laboratoire national de sante, Microbiology, Microbial Genomics Platform	Anke Wienecke-Baldacchino; Caroline Scheiber; Catherine Ragimbeau; Fatu Djabi; Jessica Tapp; Lise Pignon; Raoul Salmon; Serge Vedy; Tamir Abdelrahman
EPI_ISL_831955	Laboratoriemedicin, Klinisk mikrobiologi	The Public Health Agency of Sweden	Department of Microbiology; The Public Health Agency of Sweden
EPI_ISL_1288393	Laboratorio Central de Epidemiología (LCE)	Instituto de Biotecnología de la UNAM	Alejandro Sanchez-Flores; Alfredo Herrera-Estrella; Alicia Ocaña-Mondragón; Angel Gustavo Salas-Lais; Bernardo Martínez-Miguel; Blanca Taboada; Brenda Irasema Maldonado-Meza; Carla Ivón Herrera-Najera; Carlos F. Arias; Celia Boukadida; Clara Esperanza Santacruz-Tinoco; Concepción Grajales-Muñiz; Consorcio Mexicano de Vigilancia Genómica (CoViGen-Mex). Authors (in alphabetical order): Julio Elias Alvarado-Yaah; Fernando Fontove-Herrera; Francisco Pulido; Gloria Elena Espinoza-Ayala; Gloria María Molina-Salinas; Gloria Vazquez; Hector Esteban Paz-Juárez; Hector Montoya-Fuentes; Helen Haydee Fernanda Ramírez-Plascencia; Jorge Ivan Salinal-Nevarez; José Antonio Enciso-Moreno; José Esteban Muñoz-Medina; José de Jesús Nuñez-Contreras; Juan Bautista Chale-Dzul; Luis Alberto Ochoa-Carrera; Margarita Matías-Florentino; María Guadalupe Santiago-Mauricio; María Guadalupe de Jesús Mireles-Rivera; Nelly Sélem-Mojica; Pavel Isa; Ricardo Grande; Santiago Ávila-Ríos; Víctor Hugo Borja-Aburto
EPI_ISL_1395939	Laboratorio Central de Salud Pública	Grupo de Genómica y Bioinformática del Instituto de Investigación de la Cadena Láctea CONICET-INTA on behalf of 'Proyecto Argentino Interinstitucional de genomica de SARS-CoV-2' (PAIS Consortium)	AF; Amadio; Antonieta Cayré; Eberhardt; Erica Struss; Esteban Paredes; Irazoqui; Laura Lescano; MF; Natalia Andrea Ayala y María Verónica Gómez
EPI_ISL_1534000	Laboratorio Central de Saude Publica do Estado do Parana (LACEN-PR)	Laboratory of Respiratory Viruses and Measles, Oswaldo Cruz Institute, FIOCRUZ	Alice Sampaio Rocha; Ana Carolina Mendonca; Anna Carolina Paixao; Fernando Motta; Irina Nastassja Riediger; Luciana Appolinario; Maria do Carmo Debur; Marilda Siqueira on behalf of the Fiocruz COVID-19 Genomic Surveillance Network; Paola Resende; Renata Serrano Lopes
EPI_ISL_1395881, EPI_ISL_1395917	Laboratorio Central de la Ciudad de Santa Fe	Grupo de Genómica y Bioinformática del Instituto de Investigación de la Cadena Láctea CONICET-INTA on behalf of 'Proyecto Argentino Interinstitucional de genomica de SARS-CoV-2' (PAIS Consortium)	AF; Amadio; C; Eberhardt; G; Irazoqui; JM; MF; Mugna; Ojeda; Pastor; Rompato; V
EPI_ISL_1395990, EPI_ISL_1395991, EPI_ISL_1396029, EPI_ISL_1396038, EPI_ISL_1396056	Laboratorio Central, Ministerio de Salud Cordoba	Instituto de Patologia Vegetal (CIAP-INTA) on behalf of 'Proyecto Argentino Interinstitucional de genomica de SARS-CoV-2' (PAIS Consortium)	Barbas, G.; Castro, G.; Debat, HJ.; FD; Fernández; M.B.; Marquez, N.; Pisano; Re, V.
EPI_ISL_792532	Laboratorio Central, Ministerio de Salud Córdoba	Instituto de Patologia Vegetal (CIAP-INTA) on behalf of 'Proyecto Argentino Interinstitucional de genomica de SARS-CoV-2' (PAIS Consortium)	Barbas, G.; Castro, G.; Debat, HJ.; FD; Fernández; MB; Pisano; Re; V
EPI_ISL_779193	Laboratorio Estatal de Salud Pública de Nuevo León	Laboratorio de Infectología Molecular, Departamento de Bioquímica y Medicina Molecular,Facultad de Medicina - Universidad Autónoma de Nuevo León	Ana M. Rivas-Estilla; Consuelo Treviño-Garza; Daniel Arellanos-Soto; Else del Carmen García-García; Gloria A. Jasso-de-la-Peña; Kame A. Galán-Huerta; Manuel E. de-la-O-Cavazos; María F. Herrera-Saldivar; Natalia Martínez-Acuña; Roberto Montes-de-Oca; Samuel Buentello-Wong; Sonia A. Lozano-Sepúlveda
EPI_ISL_794592	Laboratorio Estatal de Salud Pública de Tamaulipas	Instituto de diagnóstico y Referencia Epidemiologicos (INDRE)	Abril Rodriguez-Maldonado; Ana Maria Cortez-Calderon; Bernardita Reyes-Berrones; Celia Alpuche-Aranda; Claudia Wong-Arambula; Ernesto Ramirez-Gonzalez.; Fabiola Garces-Ayala; Gisela Barrera-Badillo; Gloria Molina-Gamboa; Hilda del Carmen-Selvera; Hugo Lopez Gatell-Ramirez; Irma Lopez-Martinez; Jose Luis Alomia-Zegarra; Lucia Hernandez-Rivas
EPI_ISL_837579	Laboratorio Nacional de Salud	Laboratory of Respiratory Viruses and Measles, Oswaldo Cruz Institute, FIOCRUZ	Ana Carolina Mendonca; Anna Carolina Paixao; Cesar Roberto Conde Pereira; Claudia Estrada; Fernando Motta; Luciana Appolinario; Marilda Siqueira on behalf of the Fiocruz COVID-19 Genomic Surveillance Network; Paola Resende
EPI_ISL_842652	Laboratorio de Biología Molecular Hospital Pedro de Elizalde	Grupo de Genómica y Bioinformática del Instituto de Investigación de la Cadena Láctea CONICET-INTA on behalf of 'Proyecto Argentino Interinstitucional de genomica de SARS-CoV-2' (PAIS Consortium)	A; AF; Alegre; Alexay; Amadio; Aulicino; B; Bressan; C; Chamorro; Claps; D; Diaz; E; Eberhardt; F; FJ; G; Gondolessi; Goya; Gómez; Indart; Irazoqui; J; König; L; Lorenzo; Lusso; M; ME; MF; ML; MS; Marchetti; Martín; Montoto Piazza; Morandi; N; Nabaes Jodar; Natale; Osaba; P; Paez; Rocovich; Rosales; S; Sanchez; Sueiro; Torres; Valinotto; Viegas, M.; Wenk; Zamora
EPI_ISL_1068113, EPI_ISL_1068154, EPI_ISL_1068159	Laboratorio de Ecologia de Doencas Transmissiveis na Amazonia, Instituto Leonidas e Maria Deane - Fiocruz Amazonia	Laboratorio de Ecologia de Doencas Transmissiveis na Amazonia, Instituto Leonidas e Maria Deane - Fiocruz Amazonia	André Corado; Debora Duarte; Felipe Naveca on behalf of the Fiocruz COVID-19 Genomic Surveillance Network; Fernanda Nascimento; George Silva; Karina Pessoa; Luciana Gonçalves; Maria Júlia Brandão; Matilde Mejía; Michele Jesus; Valdinete Nascimento; Victor Souza; Ágatha Costa
EPI_ISL_1629798	Laboratorio de Genómica Microbiana, Universidad Peruana Cayetano Heredia	Laboratorio de Genómica Microbiana, Universidad Peruana Cayetano Heredia	Alejandra Dávila-Barclay; Diego Cuicapuza; Guillermo Salvatierra; Janet Huancachoque; Lenin Maturrano; Luis González; Pablo Tsukayama; Pedro E. Romero; Pool Marcos
EPI_ISL_591533	Laboratorio de Infectologia y virologia molecular	Center for Mathematical Modeling and Center for Genome Regulation. Santiago, Chile	Allende ML; Ferres M.; Gaete A; González M; Maass A; Palma R; Travisany D; Urra C; Valiente F; Varas M
EPI_ISL_953415, EPI_ISL_953420, EPI_ISL_953423, EPI_ISL_953424	Laboratorio de Investigaciones de Baney	"Swiss Tropical and Public Health Institute"	"Carlos Cortes; Bonifacio Manguire Nlavo; Claudia Daubenberger; Diosdado Odjama Nseng Ada; Elizabeth Nyakarungu; Guillermo Garcia; Maximilian Mpina; Mitoha Ondo O Ayekaba; Philip Wonder Phiri"; Salome Hosch; Tobias Schindler
EPI_ISL_648312, EPI_ISL_648315, EPI_ISL_648326, EPI_ISL_648340, EPI_ISL_648341, EPI_ISL_648344, EPI_ISL_648353, EPI_ISL_648354, EPI_ISL_649158, EPI_ISL_649166			
see above	Laboratorio de Investigaciones de Baney	University Hospital Basel, Clinical Bacteriology	Adrian Egli; Alfredo Mari; Bonifacio Manguire Nlavo; Carlos Cortes; Claudia Daubenberger; Diosdado Odjama Nseng Ada; Elizabeth Nyakarungu; Guillermo Garcia; Helena Seth-Smith; Madlen Stange; Maximilian Mpina; Mitoha Ondo O Ayekaba; Philip Wonder Phiri; Salome Hosch; Tim Roloff; Tobias Schindler
EPI_ISL_1111418	Laboratorio de Referencia Nacional de Enteropatógenos. Instituto Nacional de Salud del Perú	Laboratorio de Referencia Nacional de Enteropatógenos. Instituto Nacional de Salud del Perú	Fiorella Orellana Peralta; Iris Silva Molina; Junior Caro Castro; Ronnie Gavilan Chavez; Veronica Hurtado Vela; Willi Quino Sifuentes
EPI_ISL_491435, EPI_ISL_1092332, EPI_ISL_1092358, EPI_ISL_1532182, EPI_ISL_1532212, EPI_ISL_1532216, EPI_ISL_1534629, EPI_ISL_1534670			
see above	Laboratorio de Referencia Nacional de Virus Respiratorio. Instituto Nacional de Salud Perú	Laboratorio de Referencia Nacional de Biotecnología y Biología Molecular. Instituto Nacional de Salud Perú	Carlos Padilla Rojas; Henri Bailon Calderon; Johanna Balbuena Torres; Johanna Balbuena Torrez; Karolyn Vega Chozo; Luis Barcena; Marco Galarza Perez; Maribel Huaringa Nuñez; Nancy Rojas Serrano; Nancy Rojas Serrano.; Omar Caceres Rey; Priscila Lope Pari
EPI_ISL_1111137, EPI_ISL_1111139, EPI_ISL_1111232, EPI_ISL_1111258, EPI_ISL_1111298, EPI_ISL_1111303, EPI_ISL_1111372, EPI_ISL_1111451, EPI_ISL_1111452			
see above	Laboratorio de Referencia Nacional de Virus Respiratorio. Instituto Nacional de Salud Perú	Laboratorio de Referencia Nacional de Enteropatógenos. Instituto Nacional de Salud del Perú	Fiorella Orellana Peralta; Iris Silva Molina; Junior Caro Castro; Ronnie Gavilan Chavez; Veronica Hurtado Vela; Willi Quino Sifuentes
EPI_ISL_529074, EPI_ISL_540929, EPI_ISL_812458	Laboratorio de Referencia Nacional de Virus Respiratorios, Instituto Nacional de Salud Peru	Laboratorio de Genómica Microbiana, Universidad Peruana Cayetano Heredia	Alejandra Dávila-Barclay; Brenda Ayzanoa; Camila Castillo-Vilcahuaman; Camila Castillo-Vilcahuamán; Guillermo Salvatierra; Janet Huancachoque; Luis González; Maribel Huaringa; Nancy Rojas; Oscar Escalante; Pablo Tsukayama; Pedro E. Romero; Pool Marcos; Priscila Lope
EPI_ISL_1396068	Laboratorio de Salud Pública	Instituto de Patologia Vegetal (CIAP-INTA) on behalf of 'Proyecto Argentino Interinstitucional de genomica de SARS-CoV-2' (PAIS Consortium)	A. Mariana B. Salmerón; Amadio; Ana Maria Zamora; Dardo E. Costas; Debat, HJ.; FD; Fernández; Graciela Alabarse.; Gustavo Ruìz de Huidobro; Irazoqui, M.; Marquez, N.
EPI_ISL_1626618, EPI_ISL_1626619, EPI_ISL_1626621	Laboratorio de Salud Pública Bogota	Gencore - Universidad de los Andes	Alejandro Gomez; Ana Maria Palacio; David Gonzalez; Gabriela Delgado; Johana Hernandez; Luisa Sacristan; Marcela Guevara; Silvia Restrepo
EPI_ISL_717855	Laboratorio de Virologia Molecular / UFRJ	Bioinformatics Laboratory / LNCC	Alexandra L Gerber; Amilcar Tanuri; Ana Paula de C Guimarães; Ana Tereza R de Vasconcelos; Andréa Cony Cavalcanti; Carolina M Voloch; Claudia dos Santos Rodrigues; Cynthia C Cardoso; Diana Mariani; Luiz G P de Almeida; Otavio Bustrolini; Ronaldo da Silva F Jr; Terezinha M P P Castiñeira
EPI_ISL_1396254	Laboratorio de Virología del Hospital de Niños Dr. Ricardo Gutierrez	Biocódices SA. on behalf of 'Proyecto Argentino Interinstitucional de genomica de SARS-CoV-2' (PAIS Consortium)	A; Acevedo; Alexay; Alvarez Lopez; Barreda Frank; Berros; C; Dopazo, H.; E; G; Grandis; J; JM; Jacques; Labarta; M; ME; Medina; Mistchenko; N; O; S; Streitenberger; Thomas; Villegas; Zubrzycki J
EPI_ISL_792171, EPI_ISL_792247, EPI_ISL_1395779, EPI_ISL_1395780, EPI_ISL_1395792, EPI_ISL_1396191, EPI_ISL_1396224, EPI_ISL_1396225, EPI_ISL_1396228			
see above	Laboratorio de Virología del Hospital de Niños Dr. Ricardo Gutierrez	Área de Secuenciación del Laboratorio de Virología del Hospital de Niños Dr. Ricardo Gutierrez on behalf of 'Proyecto Argentino Interinstitucional de genomica de SARS-CoV-2' (PAIS Consortium)	A; AS; Acevedo; Acuña; Alexay; Alvarez Lopez; Barreda Frank; C; D; E; G; Goya; Grandis; Gravis; Jacques; LE; Labarta; Lusso; M; ME; MI; MS; Medina; Mistchenko; N; Nabaes Jodar; Natale; O; S; Streitenberger; Thomas; Valinotto; Viegas, M.; Villegas
EPI_ISL_1395789	Laboratorio del Hospital Interzonal General de Agudos "Evita"	Área de Secuenciación del Laboratorio de Virología del Hospital de Niños Dr. Ricardo Gutierrez on behalf of 'Proyecto Argentino Interinstitucional de genomica de SARS-CoV-2' (PAIS Consortium)	Acuña; Alejandra Musto; Alexay; D; Erica Luczak; Goya; Isabel Desimone; LE; Lorena Serrano; Lusso; M; MI; Nabaes Jodar; Natale; Omar Grossi; Rubén Pelagamos; S; Valinotto; Viegas, M.
EPI_ISL_1396355, EPI_ISL_1396359, EPI_ISL_1396363	Laboratorio del Hospital Regional Ushuaia Gdor. Ernesto Campos	Nodo de Secuenciación Tierra del Fuego - Hospital Regional Ushuaia - Centro Austral De Investigaciones Científicas - Universidad Nacional De Tierra Del Fuego on behalf of 'Proyecto Argentino Interinstitucional de genomica de SARS-CoV-2' (PAIS Consortium)	Alejandro Ezequiel Rojas; Carina Andrea De Roccis; Carolina Beatriz Yulan; Cristina Fernanda Nardi; Fernando Gallego; Gabriel Alejandro Castro; Ivan Dario Gramundi; Manuel Fabian Boutureira; Santiago Guillermo Ceballos; Silvana Beatriz Cáceres
EPI_ISL_961466	Laboratorios Lister	Instituto de Diagnostico y Referencia Epidemiologicos (INDRE)	Abril Rodriguez-Maldonado; Claudia Wong-Arambula; Ernesto Ramirez-Gonzalez.; Fabiola Garces-Ayala; Gisela Barrera-Badillo; Irma Lopez-Martinez; Lucia Hernandez-Rivas; Natividad Cruz-Ortiz; Tatiana Nunez-Garcia
EPI_ISL_882960	Laboratorium Diagnostyi Mikrobiologicznej z Pracownią Prątka Gruźlicy SPSzW im. Jana Bożego w Lublinie	National Institute of Public Health - National Institute of Hygiene	Gawor Jan; Wołkowicz Tomasz; Zacharczuk Katarzyna
EPI_ISL_1221253, EPI_ISL_1221448, EPI_ISL_1319051, EPI_ISL_1339107, EPI_ISL_1421963, EPI_ISL_1462725, EPI_ISL_1481208, EPI_ISL_1515565, EPI_ISL_1548200, EPI_ISL_1611694, EPI_ISL_1612177, EPI_ISL_1612202, EPI_ISL_1612251, EPI_ISL_1612254, EPI_ISL_1612429, EPI_ISL_1612767, EPI_ISL_1612819, EPI_ISL_1612820			
see above	Laboratory Corporation of America	Centers for Disease Control and Prevention Division of Viral Diseases, Pathogen Discovery	Adrian Paskey; Amanda Douglas; Amanda Suchanek; Andrea Throop; Ayla Burns; Ben L. Rambo-Martin; Benjamin Rambo-Martin; Bobbi Croy; Brian Krueger; Brian Norvell; Christopher Gulvick; Christos Petropoulos; Clinton R. Paden; Craig Lukasik; Dakota Howard; Darlene Wagner; Debbie Boles; Dhwani Batra; Duncan MacCannell; Eyad Almasri; Goran Stevovic; Howard Engler; Hrushikesh Deshmukh; Jake Humphrey; Jana Schroth; Jason Caravas; Joe Voshell; John Pruitt; Jonathan Meltzer; Jonathan Williams; Kara Moser; Kimberly Wagner; Lax Iyer; Lyndon Tilson; Manoj Jain; Marcia Eisenberg; Mary Ann Cristobal; Mary Williamson; Matthew Schmerer; Michael Levandoski; Mike Sapeta; Mindy Nye; Minoo Agarwal; Mohan Kolli; Nuthawin Charoensri; Oren Cohen; Peter W. Cook; Prashant Gupta; Qian Zeng; Rama Ghatti; Scott Parker; Scott Ryan; Scott Sammons; Shatavia Morrison; Stanley Letovsky; Steven Ragan; Suresh Babu Selvaraju; Susan Countryman; Susan Hicks; Suxiang Tong; Suzanne Dale; Thomas Urban; Tim Kuphal; Tricia Zwiefelhofer; Vincent Drouillon; Yvette Unoarumhi
EPI_ISL_1029548, EPI_ISL_1029554, EPI_ISL_1029740, EPI_ISL_1032075, EPI_ISL_1038457, EPI_ISL_1081277, EPI_ISL_1163080			
see above	Laboratory Corporation of America	Respiratory Viruses Branch, Division of Viral Diseases, Centers for Disease Control and Prevention	Alexandre Bolze; Amanda Douglas; Amanda Suchanek; Andrea Throop; Ary Ascencio; Ayla Burns; Ben L. Rambo-Martin; Bobbi Croy; Brad Sickler; Brian Krueger; Brian Norvell; Charlotte Rivera-Garcia; Christine Tran; Christos Petropoulos; Clinton R. Paden; Craig Lukasik; Dakota Howard; David Becker; Dhwani Batra; Duncan MacCannell; Efren Sandoval; Eileen de Feo; Elizabeth Cirulli; Eric Allen; Eyad

			Almasri Debbie Boles; Geraint Levan; Goran Stevovic; Howard Engler; Hrushikesh Deshmukh; Jake Humphrey; James Lu; Jan Antico; Jana Schroth; Jason Nguyen; Jimmy Ramirez; Jingtao Liu; Joe Voshell; John Pruitt; Jonathan Meltzer; Jonathan Williams; Kelly Schiabor Barrett; Kim Gietzen; Kimberly Wagner; Lax Iyer; Lyndon Tilson; Magnus Isaksson; Manoj Jain; Marc Laurent; Marcia Eisenberg; Mary Ann Cristobal; Mary Williamson; Matthew Tolentino; Michael Levandoski; Mike Sapeta; Mindy Nye; Minoo Agarwal; Mohan Kolli; Nicole L. Washington; Nuthawin Charoensri; Oren Cohen; Peter W. Cook; Phil Febbo; Prashant Gupta; Qian Zeng; Rama Ghatti; Ryan Cho; Scott Parker; Scott Ryan; Shannon Wickline; Sherry Wang; Simon White; Stanley Letovsky; Steven Ragan; Summer Galloway; Suresh Babu Selvaraju; Susan Countryman; Susan Hicks; Suxiang Tong; Suzanne Dale; Thomas Urban; Tim Kuphal; Tricia Zwiefelhofer; Tyler Cassens; Vincent Drouillon; William Lee
EPI_ISL_644565	Laboratory Diagnostic, Veterinary Specialized Institute Kraljevo	Laboratory Diagnostic, Veterinary Specialized Institute Kraljevo	Afonso, C.; Banovic Djeri, B.; Jankovic, M.; Jovanovic, T.; Knezevic, A.; Petrovic, T.; Sekler, M.; Tesovic, B.; Vidanovic, D.; Volkening, J.
EPI_ISL_660543, EPI_ISL_660544, EPI_ISL_956328, EPI_ISL_956330	Laboratory Medicine	Department of Laboratory Medicine, Lin-Kou Chang Gung Memorial Hospital, Taoyuan, Taiwan	Cheng-Hsun Chiu; Cheng-Ta Yang; Chung-Guei Huang; Guang-Wu Chen; Kuo-Chien Tsao; Kuo-Ming Lee; Mei-Jen Hsiao; Peng-Nien Huang; Po-Wei Huang; Shin-Ru Shih; Shu-Li Yang; Yi-Chun Liu; Yu-Nong Gong
EPI_ISL_1138899, EPI_ISL_1138969, EPI_ISL_1209407, EPI_ISL_1508895	Laboratory for HIV and opportunistic infections diagnosis The Republican Research and Practical Center for Epidemiology and Microbiology (RRPCEM)	Laboratory for HIV and opportunistic infections diagnosis The Republican Research and Practical Center for Epidemiology and Microbiology (RRPCEM)	Alena Mikhalenka; Alexander Kilchevcky; Alina Drozd; Analoly Krasko; Anatoly Krasko; Artur Akhremchuk; Elena Gasich; Kirill Bulda; Leonid Valentovich; Nastassia Kabankova; Vladimir Gorbunov; Yauhen Sysaliatsin
EPI_ISL_754238, EPI_ISL_910336, EPI_ISL_1224912, EPI_ISL_1381323	Laboratory for Respiratory Viruses, Cantacuzino National Military-Medical Institute for Research and Development	Cantacuzino Institute Virology	Catalina Pascu; Luiza Ustea; Mihaela Lazar; Mühlemann Barbara; Nicoleta Paraschiv; Sorin Dinu
EPI_ISL_1138520, EPI_ISL_1138522, EPI_ISL_1138539, EPI_ISL_1138552	Laboratory of Communicable Diseases	1. Laboratory of Communicable Diseases (Estonia); 2. Eurofins Genomics Europe Sequencing GmbH	Liidia Dotsenko
EPI_ISL_717979	Laboratory of Microbiology and Infectious Diseases, Faculty of Veterinary Medicine, Aristotle University of Thessaloniki, University Campus, 541 24, Thessaloniki, Greece.	Laboratory of Biology, Department of Medicine, Democritus University of Thrace, Alexandroupolis, Greece	Bampali M.; Chaintoutis S.; Dimitriou M.; Dovas C.; Dovrolis N.; Karakasiliotis I.
EPI_ISL_654016, EPI_ISL_654017, EPI_ISL_654018, EPI_ISL_654020	Laboratory of Microbiology, National Reference Lab, Charles Nicolle Hospital; 2-University of Tunis ElManar, Faculty of Medicine of Tunis, LR99ES09, Tunis, Tunisia	1-Clinical and Experimental Pharmacology Lab, LR16SP02, National Center of Pharmacovigilance, University of Tunis El Manar, Tunis, Tunisia. 2-Neurodegenerative diseases and psychiatric troubles, LR18SP03, Razi Hospital, University of Tunis El Manar, Tunis, Tunisia. 3-Ministry of Health, National Observatory of New and Emerging Diseases, 1006, Tunis, Tunisia	Alia Ben Kahla; Gaies Emna; Ilhem Boutiba-Ben Boubaker; Imen Kacem; Imen Mkada; Jalila Ben Khelil; Maher Kharrat; Mouna Ben Sassi; Mouna Safer; Nissaf Ben Alaya; Riadh Daghfous; Riadh Gouider.; Salma Abid; Sameh Trabelsi; Sana Ferjani; Soumaya Rammeh
EPI_ISL_796782, EPI_ISL_803119, EPI_ISL_803120, EPI_ISL_803851	Laboratory of Microbiology, National Reference Lab, Charles Nicolle Hospital; 2-University of Tunis ElManar, Faculty of Medicine of Tunis, LR99ES09, Tunis, Tunisia	Clinical and Experimental Pharmacology Lab, LR16SP02, National Center of Pharmacovigilance, University of Tunis El Manar, Tunis, Tunisia. 2-Neurodegenerative diseases and psychiatric troubles, LR18SP03, Razi Hospital, University of Tunis El Manar, Tunis, Tunisia. 3-Ministry of Health, National Observatory of New and Emerging Diseases, 1006, Tunis, Tunisia	Ahmed Fakhfakh; Alia BenKahla; Gaies Emna; Guedi Ali Barreh; Habiba Ben Romdhane; Hanen El Jebari; Ilhem Boutiba-Ben Boubaker; Jalila Ben Khelil; Maher Kharrat; Mouna Ben Sassi; Mouna Safer; Nissaf Ben Alaya; Riadh Daghfous; Riadh Gouider.; Salma Abid; Sameh Trabelsi; Sana Ferjani; Sarra Chamman; Souissi Amira; Zaineb Hamzaoui
EPI_ISL_876011	Laboratory of Molecular Biology, Diagnostyka sp. z o.o.	genXone SA, Research & Development Laboratory	Grzegorz Nowicki; Jakub Grabowski; Maciej Sykulski; Michał Kaszuba; Monika Mańkowska-Woźniak; Natalia Drwęska-Matelska; Łukasz Krych
EPI_ISL_625677	Laboratory of Molecular Medicine, University of Magallanes	Centro Asistencial Docente y de Investigacion, Universidad de Magallanes	Diego Alvarez; Jacqueline Aldridge; Jorge Gonzalez; Marcelo Navarrete
EPI_ISL_1181512	Laboratory of Respiratory Viruses and Measles, Oswaldo Cruz Institute, FIOCRUZ	Laboratory of Respiratory Viruses and Measles, Oswaldo Cruz Institute, FIOCRUZ	Alice Sampaio Rocha; Ana Carolina Mendonca; Anna Carolina Paixao; Fernando Motta; Luciana Appolinario; Marilda Siqueira on behalf of the Fiocruz COVID-19 Genomic Surveillance Network; Paola Resende; Renata Serrano Lopes
EPI_ISL_1527383	Laboratory of Virology of Federal Budget Health Care Institution "Center of Hygiene and Epidemiology in Arkhangelsk region"	Group of Genomics and Postgenomic Technologies of Central Research Institute of Epidemilology	Akimkin VG; Bulanenko VP; Cherkashina AS; Golubeva AG; Kaptelova VV; Kondrasheva LY; Korneenko EV; Ponomareva YA; Saenko SS; Samoilov AE; Semyina LV; Shishko LA; Speranskaya AS; Tivanova EV; Valdokhina AV
EPI_ISL_1341380, EPI_ISL_1341501, EPI_ISL_1341504, EPI_ISL_1341643, EPI_ISL_1365742, EPI_ISL_1489932	Laboratory of Virology, National center of expertise	RSE "National Center of Expertise" and RSE "National center for Biotechnology"	Abdaliyev Askar; Amirgazin Asylulan; Balykbaev Kanat; Kamalova Dinara; Ramankulov Erlan; Sharipova Saule; Shevtsov Alexandr; Tungushbayev Talgat
EPI_ISL_1191753	Laboratory of virology and molecular diagnostics	Laboratory of virology and molecular diagnostics	Boshevska Golubinka; Janchevska Elizabeta; Kuzmanovska Maja
EPI_ISL_454575	Laboratory of virology, National Center of Expertise	Laboratory of molecular-genetic research, National Center for Expertise, Kazakhstan National Center for Biotechnology, Kazakhstan	; Abdaliyev Askar; Akhmetollayev Ilyas; Amirgazin Asylulan; Aushakhmetova Zabira; Kalendar Ruslan; Lutsay Viktoriya; Rakhmetova Akbota; Ramankulov Yerlan; Shevtsov Alexandr
EPI_ISL_454596	Laboratory of virology, National Center of Expertise	Laboratory of molecular-genetic research, National Center for Expertise, Kazakhstan National Center for Biotechnology, Kazakhstan	; Abdaliyev Askar; Akhmetollayev Ilyas; Amirgazin Asylulan; Aushakhmetova Zabira; Kalendar Ruslan; Lutsay Viktoriya; Rakhmetova Akbota; Ramankulov Yerlan; Shevtsov Alexandr
EPI_ISL_1334517, EPI_ISL_1335044, EPI_ISL_1365365, EPI_ISL_1448017, EPI_ISL_1448018, EPI_ISL_1448020	Laboratory of virology, National center of expertise	RSE "National Center for Biotechnology" and RSE "National Center of Expertise"	Abdaliyev Askar; Amirgazin Asylulan; Balykbaev Kanat; Kamalova Dinara; Ramankulov Yerlan; Sharipova Saule; Shevtsov Alexandr; Tungushbayev Talgat
EPI_ISL_792616	Laboratório Central de Saúde Pública do Estado da Paraíba (LACEN-PB)	Laboratory of Respiratory Viruses and Measles, Oswaldo Cruz Institute, FIOCRUZ	Ana Carolina Mendonca; Anna Carolina Paixao; Dalane Loudal Florentino Teixeira; Fernando Motta; João Felipe Bezerra; Luciana Appolinario; Marilda Siqueira on behalf of the Fiocruz COVID-19 Genomic Surveillance Network; Paola Resende; Romero Henrique Teixeira de Vasconcelos; Thiago Franco de Oliveira Carneiro
EPI_ISL_729842	Laboratório Central de Saúde Pública do Estado do Rio Grande do Sul (LACEN-RS)	Laboratory of Respiratory Viruses and Measles, Oswaldo Cruz Institute, FIOCRUZ	Ana Carolina Mendonça; Anna Carolina Paixão; Fernando Motta; Luciana Appolinario; Marilda Siqueira on behalf of the Fiocruz COVID-19 Genomic Surveillance Network; Marilda Tereza Mar da Rosa; Paola Resende; Tatiana Schaffer Gregianini
EPI_ISL_1182582	Laboratório Central do Estado do Paraná	Coordenação Geral de Laboratórios de Saúde Pública (CGLAB/DAEVS/SVS/MS)	Vagner Fonseca; et al.
EPI_ISL_1494995, EPI_ISL_1495026	Laboratório de Biologia Integrativa	Laboratório de Biologia Integrativa	Alessandro Clayton de Souza Ferreira; Aline Brito de Lima; Carolina Moreira Voloch; Daniel Costa Queiroz; Danielle Alves Gomes Zauli; Diego Menezes Bonfim; Filipe Romero Rebello Moreira; Frederico Scott Varella Malta; Joice do Prado Silva; Lucyene Miguita Luiz; Nuno Rodrigues Faria; Paula Luize Camargos Fonseca; Rafael Marques de Souza; Renan Pedra de Souza; Renato Santana Aguiar; Rennan Garcias Moreira; Victor Cavalcanti Pardini; Victor Emmanuel Viana Geddes
EPI_ISL_1299913, EPI_ISL_1299923	Labormedizinisches Zentrum Dr Risch	Clinical Bacteriology	Adrian Egli; Alfredo Mari; Hans Hirsch; Helena MB Seth-Smith; Julia Bielicki; Karoline Leuzinger; Lorenz Risch; Madlen Stange; Manuel Battegay; Martin Risch; Nadia Wohlwend; Tim Roloff
EPI_ISL_1585270	Labuan Hospital	Institute for Medical Research, Infectious Disease Research Centre, National Institutes of Health, Ministry of Health Malaysia	Kamel K; Mohd Zawawi Z; Ramly N; Robert F; Suppiah J; Thayan R
EPI_ISL_700466	Ladismith (Nissenville) Clinic wc LAF	NHLS/UCT	Arash Iranzadeh; Bruna Galvao; Carolyn Williamson; Deelan Doolabh; Diana Hardie; Innocent Mudau; Kruger Marais; Lynn Tyers; Marvin Hsiao; Stephen Korsman
EPI_ISL_745152	Laetitia Bam CHC	National Health Laboratory Service (NHLS), Tygerberg	Bronwyn Kleinhans; Eduan Wilkindon; Gert van Zyl; Houriiyah Tegally; Kayla Delaney; Susan Engelbrecht; Tulio de Oliveira; Wolfgang Preiser
EPI_ISL_623073	Lancet Laboratories	National Institute for Communicable Diseases of the National Health Laboratory Service	Allam M; Bhiman JN; Ismail A; Khumalo Z; Kwenda S; Mnyameni F; Mohale T; Mtshali P; Subramoney K
EPI_ISL_767878	Laverty Pathology	NSW Health Pathology - Institute of Clinical Pathology and Medical Research; Westmead Hospital; University of Sydney	CIDM-PH et al.
EPI_ISL_760644, EPI_ISL_796857, EPI_ISL_874422, EPI_ISL_885871, EPI_ISL_1594881	Lighthouse Lab in Alderley Park	Wellcome Sanger Institute for the COVID-19 Genomics UK (COG-UK) Consortium	Cordelia Langford; David K. Jackson; Dominic Kwiatkowski; Ewan Harrison; Ian Johnston; Jacquelyn Wynn; Jeffrey Barrett; John Sillitoe on behalf of the Wellcome Sanger Institute COVID-19 Surveillance Team; Mairead Hyland; Roberto Amato; Sonia Goncalves; The Lighthouse Lab in Alderley Park and Alex Alderton
EPI_ISL_933172, EPI_ISL_1242585	Lighthouse Lab in Cambridge	Wellcome Sanger Institute for the COVID-19 Genomics UK (COG-UK) Consortium	Cordelia Langford; David K. Jackson; Dominic Kwiatkowski; Ewan Harrison; Ian Johnston; Jeffrey Barrett; John Sillitoe on behalf of the Wellcome Sanger Institute COVID-19 Surveillance Team; Rob Howes; Roberto Amato; Sonia Goncalves; The Lighthouse Lab in Cambridge and Alex Alderton
EPI_ISL_760310, EPI_ISL_761991, EPI_ISL_799192, EPI_ISL_1544757, EPI_ISL_1544824	Lighthouse Lab in Glasgow	Wellcome Sanger Institute for the COVID-19 Genomics UK (COG-UK) Consortium	Anna Dominiczak and Alex Alderton; Carol Clugston; Cordelia Langford; David Gray; David K. Jackson; Dominic Kwiatkowski; Ewan Harrison; Harper VanSteenhouse; Ian Johnston; Jeffrey Barrett; John Sillitoe on behalf of the Wellcome Sanger Institute COVID-19 Surveillance Team; Roberto Amato; Sonia Goncalves; Yumi Kasai
EPI_ISL_589829	Lighthouse Lab in Glasgow	Wellcome Sanger Institute for the COVID-19 Genomics UK (COG-UK) consortium	Anna Dominiczak and Alex Alderton; Carol Clugston; Cordelia Langford; David Gray; David K. Jackson; Dominic Kwiatkowski; Ewan Harrison; Harper VanSteenhouse; Ian Johnston; John Sillitoe on behalf of the Wellcome Sanger Institute COVID-19 Surveillance Team (http://www.sanger.ac.uk/covid-team); Roberto Amato; Sonia Goncalves; Yumi Kasai
EPI_ISL_1224364	Lighthouse Lab in Milton Keynes	Wellcome Sanger Institute for the COVID-19 Genomics UK (COG-UK) Consortium	Cordelia Langford; David K. Jackson; Dominic Kwiatkowski; Ewan Harrison; Ian Johnston; Jeffrey Barrett; John Sillitoe on behalf of the Wellcome Sanger Institute COVID-19 Surveillance Team; Roberto Amato; Sonia Goncalves; The Lighthouse Lab in Milton Keynes and Alex Alderton

EPI_ISL_557248, EPI_ISL_557377	Lighthouse Lab in Milton Keynes	Wellcome Sanger Institute for the COVID-19 Genomics UK (COG-UK) consortium	Cordelia Langford; David K. Jackson; Dominic Kwiatkowski; Ewan Harrison; Ian Johnston; John Sillitoe on behalf of the Wellcome Sanger Institute COVID-19 Surveillance Team (http://www.sanger.ac.uk/covid-team); Roberto Amato; Sonia Goncalves; The Lighthouse Lab in Milton Keynes and Alex Alderton
EPI_ISL_603110, EPI_ISL_636839, EPI_ISL_770503, EPI_ISL_770511, EPI_ISL_770527	Lithuanian University of Health Sciences Hospital, Department of Laboratory Medicine	Lithuanian University of Health Sciences, Molecular cardiology lab.	Arnoldas Pautienius; Astra Vitkauskiene; Dovydas Gecys; Ingrida Olendraitė; Kamile Tamusauskaite; Laura Pareckaite; Lukas Zemaitis; Vaiva Lesauskaite
EPI_ISL_1502048	Lurie Children's Hospital of Chicago	Northwestern University - Ozer Lab	Egon A. Ozer; Judd F. Hultquist; Lacy M. Simons; Larry K. Kociolek; Michael G. Ison; Ramon Lorenzo-Redondo; Taylor J. Dean; William J. Muller; Xiaotian; Zheng
EPI_ISL_1654513	M Health Fairview	Minnesota Department of Health, Public Health Laboratory	Alexandra Lorentz; Jacob Garfin; Matt Plumb; and Xiong Wang
EPI_ISL_1258333, EPI_ISL_1258402, EPI_ISL_1594128	MB-Cadham Provincial laboratory	National Microbiology Laboratory (NML)	Anna Majer; Anneliese Landgraff; CanCOGeN's metadata curation team; Darian Hole; David Alexander; Elsie Grudeski; Gary Van Domselaar; Grace Seo; Jared Bullard; Jennifer Tanner; Kerry Dust; Kirsten Biggar; Madison Chapel; Morag Graham; Natalie Knox; Nathalie Bastien; Paul Van Caeseele; Philip Mabon; Public Health Agency of Canada CanCOGeN team; Rhiannon Huzarewich; Russell Mandes; Shari Tyson; Timothy Booth; Yan Li
EPI_ISL_736966, EPI_ISL_736995	MDS	KRISP, KZN Research Innovation and Sequencing Platform	ChimukangaraB; Giandhari J; Khan S; Lessells R; Mdlalose K; Pillay S; Tegally H; Wilkinson E; York D; de Oliveira T
EPI_ISL_804571	MEPHI, Aix Marseille University	MEPHI, Aix Marseille University	Anthony LEVASSEUR
EPI_ISL_903857	MN PHL Division, Minnesota Department of Health	Genomics and Discovery, Respiratory Viruses Branch, Division of Viral Diseases, Centers for Disease Control and Prevention	Anna Montmayeur; Anna Uehara; Ben L. Rambo-Martin; Clinton R. Paden; Dhwani Batra; Haibin Wang; Jasmine Padilla; Jing Zhang; Justin Lee; Krista Queen; Lori Rowe; Mark Burroughs; Mili Sheth; Peter W. Cook; Rachel Marine; Sarah Nobles; Suxiang Tong; Yan Li; Ying Tao
EPI_ISL_1516400	MO State Public Health Laboratory	Centers for Disease Control and Prevention Division of Viral Diseases, Pathogen Discovery	Alison Laufer Halpin; Ben L. Rambo-Martin; Clinton R. Paden; Dakota Howard; Darlene Wagner; Dave Wentworth; Dhwani Batra; Jasmine Padilla; Justin Lee; Katie Dillon; Krista Queen; Kristen Knipe; Kristine Lacek; Mark Burroughs; Matthew Schmerer; Mili Sheth; Peter Cook; Sam Shepard; Sarah Nobles; Shoshona Le; Suxiang Tong; Vivien Dugan; Yvette Unoarumhi
EPI_ISL_954226, EPI_ISL_954227, EPI_ISL_954229, EPI_ISL_954234, EPI_ISL_954237, EPI_ISL_954239, EPI_ISL_954240, EPI_ISL_954241, EPI_ISL_954242, EPI_ISL_954246, EPI_ISL_954247, EPI_ISL_954256, EPI_ISL_954257, EPI_ISL_954258, EPI_ISL_954260, EPI_ISL_954261, EPI_ISL_954262, EPI_ISL_954263, EPI_ISL_954264, EPI_ISL_954266, EPI_ISL_954267, EPI_ISL_954268, EPI_ISL_954269, EPI_ISL_954270, EPI_ISL_954271, EPI_ISL_954272, EPI_ISL_954273, EPI_ISL_954274, EPI_ISL_954276, EPI_ISL_954277, EPI_ISL_954278, EPI_ISL_954279, EPI_ISL_954280, EPI_ISL_954281, EPI_ISL_954282, EPI_ISL_954283, EPI_ISL_954285, EPI_ISL_954286, EPI_ISL_954287, EPI_ISL_954288, EPI_ISL_954289, EPI_ISL_954290, EPI_ISL_954291, EPI_ISL_954292, EPI_ISL_954294, EPI_ISL_954295, EPI_ISL_954296, EPI_ISL_954297, EPI_ISL_954298, EPI_ISL_954299, EPI_ISL_954300, EPI_ISL_955136, EPI_ISL_1469314, EPI_ISL_1469315, EPI_ISL_1469316, EPI_ISL_1469317, EPI_ISL_1469318, EPI_ISL_1469319, EPI_ISL_1469320, EPI_ISL_1469321, EPI_ISL_1469322, EPI_ISL_1469323, EPI_ISL_1469325, EPI_ISL_1469326, EPI_ISL_1469329, EPI_ISL_1469330, EPI_ISL_1469331, EPI_ISL_1469332, EPI_ISL_1469333, EPI_ISL_1469336, EPI_ISL_1469337, EPI_ISL_1469338, EPI_ISL_1469339, EPI_ISL_1469340, EPI_ISL_1469343, EPI_ISL_1469344, EPI_ISL_1469346, EPI_ISL_1469347, EPI_ISL_1469349, EPI_ISL_1469350, EPI_ISL_1469352, EPI_ISL_1469353, EPI_ISL_1469354, EPI_ISL_1469355, EPI_ISL_1469356, EPI_ISL_1469357, EPI_ISL_1469358, EPI_ISL_1469359, EPI_ISL_1469360, EPI_ISL_1469361, EPI_ISL_1469362, EPI_ISL_1469363, EPI_ISL_1469365, EPI_ISL_1469366, EPI_ISL_1469367, EPI_ISL_1469368, EPI_ISL_1469369, EPI_ISL_1469370, EPI_ISL_1469372, EPI_ISL_1469373, EPI_ISL_1469375, EPI_ISL_1469376, EPI_ISL_1469377, EPI_ISL_1469378, EPI_ISL_1469379, EPI_ISL_1469380, EPI_ISL_1469381, EPI_ISL_1469383, EPI_ISL_1469384, EPI_ISL_1469385, EPI_ISL_1469386, EPI_ISL_1469387, EPI_ISL_1469388, EPI_ISL_1469389, EPI_ISL_1469390, EPI_ISL_1469391, EPI_ISL_1469392, EPI_ISL_1469393, EPI_ISL_1469394, EPI_ISL_1469395, EPI_ISL_1469397, EPI_ISL_1469398, EPI_ISL_1469399, EPI_ISL_1469400, EPI_ISL_1469401, EPI_ISL_1469402, EPI_ISL_1469403, EPI_ISL_1469404, EPI_ISL_1469406, EPI_ISL_1469407, EPI_ISL_1469408, EPI_ISL_1469409, EPI_ISL_1469410, EPI_ISL_1469413, EPI_ISL_1469414, EPI_ISL_1469415, EPI_ISL_1469416, EPI_ISL_1469417, EPI_ISL_1469418, EPI_ISL_1469420, EPI_ISL_1469421, EPI_ISL_1469422, EPI_ISL_1469423, EPI_ISL_1469424, EPI_ISL_1469425, EPI_ISL_1469426, EPI_ISL_1469427, EPI_ISL_1469428, EPI_ISL_1469430, EPI_ISL_1469431, EPI_ISL_1469432			
see above	MRC/UVRI & LSHTM Uganda Research Unit	Where sequence data have been generated and submitted to GISAID	Dan Lule Bugembe; Isaac Sseeewanyana; Matthew Cotten; My V.T. Phan; Patrick Semanda; Pontiano Kaleebu; Susan Nabadda
EPI_ISL_428856	MRCG at LSHTM Genomics Lab	MRCG at LSHTM Genomics lab	Sesay et al
EPI_ISL_471163, EPI_ISL_560997, EPI_ISL_561000, EPI_ISL_561001, EPI_ISL_561003, EPI_ISL_561004, EPI_ISL_561006, EPI_ISL_561009, EPI_ISL_561011, EPI_ISL_561012, EPI_ISL_561013, EPI_ISL_561021, EPI_ISL_561022, EPI_ISL_561034, EPI_ISL_561038, EPI_ISL_561041, EPI_ISL_561042, EPI_ISL_561052, EPI_ISL_561060, EPI_ISL_561062, EPI_ISL_561068, EPI_ISL_561069, EPI_ISL_561070, EPI_ISL_561073, EPI_ISL_561079, EPI_ISL_561081, EPI_ISL_561082, EPI_ISL_561083, EPI_ISL_561084, EPI_ISL_561085, EPI_ISL_561087, EPI_ISL_561088, EPI_ISL_561090, EPI_ISL_561091, EPI_ISL_561093, EPI_ISL_561096, EPI_ISL_561097, EPI_ISL_561098, EPI_ISL_561099, EPI_ISL_561100, EPI_ISL_561102, EPI_ISL_561105, EPI_ISL_561107, EPI_ISL_561110, EPI_ISL_561111, EPI_ISL_561113, EPI_ISL_561114, EPI_ISL_561115, EPI_ISL_561120, EPI_ISL_561121, EPI_ISL_561128, EPI_ISL_561130, EPI_ISL_561133, EPI_ISL_561137, EPI_ISL_561138, EPI_ISL_561141, EPI_ISL_561142, EPI_ISL_561143, EPI_ISL_561144, EPI_ISL_561145, EPI_ISL_561148, EPI_ISL_561150, EPI_ISL_561151, EPI_ISL_561153, EPI_ISL_561155, EPI_ISL_561157, EPI_ISL_561158, EPI_ISL_561159, EPI_ISL_561160, EPI_ISL_561162, EPI_ISL_561164, EPI_ISL_561165, EPI_ISL_561166, EPI_ISL_561172, EPI_ISL_561173, EPI_ISL_561174, EPI_ISL_561176, EPI_ISL_561180, EPI_ISL_561181, EPI_ISL_561184, EPI_ISL_561188, EPI_ISL_561193, EPI_ISL_561194, EPI_ISL_561195, EPI_ISL_561196, EPI_ISL_561197, EPI_ISL_561199, EPI_ISL_561201, EPI_ISL_561202, EPI_ISL_561212, EPI_ISL_561215, EPI_ISL_561223, EPI_ISL_561229, EPI_ISL_561236, EPI_ISL_561238, EPI_ISL_561240, EPI_ISL_561241, EPI_ISL_561249, EPI_ISL_561260, EPI_ISL_561268, EPI_ISL_561285, EPI_ISL_561289, EPI_ISL_561291, EPI_ISL_561292, EPI_ISL_561294, EPI_ISL_561304, EPI_ISL_561310, EPI_ISL_811016, EPI_ISL_811018, EPI_ISL_811021, EPI_ISL_811022, EPI_ISL_811023, EPI_ISL_811025, EPI_ISL_811026, EPI_ISL_811028, EPI_ISL_811034, EPI_ISL_811035			
see above	MRCG at LSHTM Genomics lab	MRCG at LSHTM Genomics lab	Abdoulie Kanteh; Abdul Karim sesay; Bakary Sanyang; Jarra Manneh; Mariama Kujabi; Sesay et al
EPI_ISL_428855	MRCG at LSHTM Geomics lab	MRCG at LSHTM Genomics lab	Sesay et al
EPI_ISL_802103, EPI_ISL_802148, EPI_ISL_802258	MSHS Clinical Microbiology Laboratories	MSHS Pathogen Surveillance Program	Adolfo García-Sastre; Adriana van de Guchte; Ajay Obla; Alberto Paniz-Mondolfi; Ana S. Gonzalez-Reiche; Andrew Kasarskis; Angela Amoako; Ashley S. Salimbangon; Betsaida Salom Melo; Bremy Alburquerque; Brianne Ciferri; Charles Gleason; Deena R. Altman; Denise Jurczyszak; Elena Hirsch; Emilia Mia Sordillo; Emily Ferreri; Gintaras Deikus; Giulio Kleiner; Gopi Patel; Hala Alshammary; Harm van Bakel; Irina Oussenko; Jayeeta Dutta; Juan Soto; Katherine Beach; Kathryn Twyman; Kayla Russo; Komal Srivastava; Levy Sominsky; Mahmoud Awawda; Marta Luksza; Matthew M. Hernandez; Melissa Gitman; Michael D. Nowak; Mitchell J. Sullivan; Nancy Francoeur; Rachel Chernet; Robert Sebra; Sarah Schaefer; Shelcie Fabre; Shwetha Hara Sridhar; Viviana Simon; Ying-Chih Wang; Zenab Khan
EPI_ISL_1225887	MT Public Health Laboratory	Genomics and Discovery, Respiratory Viruses Branch, Division of Viral Diseases, Centers for Disease Control and Prevention	Anna Montmayeur; Anna Uehara; Ben L. Rambo-Martin; Clinton R. Paden; Dhwani Batra; Haibin Wang; Jasmine Padilla; Jing Zhang; Justin Lee; Katie Dillon; Krista Queen; Kristen Knipe; Kristine Lacek; Lori Rowe; Mark Burroughs; Matthew Schmerer; Mili Sheth; Peter W. Cook; Rachel Marine; Sam Shepard; Sarah Nobles; Shoshona Le; Suxiang Tong; Yan Li; Ying Tao
EPI_ISL_1094413	MT Public Health Laboratory	Respiratory Viruses Branch, Division of Viral Diseases, Centers for Disease Control and Prevention	Anna Montmayeur; Anna Uehara; Ben L. Rambo-Martin; Clinton R. Paden; Dhwani Batra; Haibin Wang; Jasmine Padilla; Jing Zhang; Justin Lee; Krista Queen; Lori Rowe; Mark Burroughs; Mili Sheth; Peter W. Cook; Rachel Marine; Sarah Nobles; Suxiang Tong; Yan Li; Ying Tao
EPI_ISL_832931, EPI_ISL_1048662	Maine HETL	Tewhey Lab, The Jackson Laboratory	Barter, M.; Dewey, H.; H. and Tewhey, R.; Iosue, F.; Lynch, R.; Matluk, N.; Munger
EPI_ISL_1372872, EPI_ISL_1523782	Maine Health and Environmental Testing Laboratory	Tewhey Lab, The Jackson Laboratory	Barter, M.; Dewey, H.; H. and Tewhey, R.; Iosue, F.; Lynch, R.; Matluk, N.; Munger
EPI_ISL_528738, EPI_ISL_1435820	Malaysia Genome Institute	Malaysia Genome Institute	Azrin Ahmad; Enizza Kasim; Irni Suhayu Sapian; Mohd Faizal Abu Bakar; Mohd Noor Mat Isa; Nor Azfa Johari; Nor Azfa Johari.; Nurhezreen Md Iqbal; Shahrul Hisham Zainal Ariffin; Shamsidar Sopie; Siti Noraini Othman; Yusuf Muhammad Noor
EPI_ISL_1335888	Martha Friska Multatuli Hospital Medan	Faculty of Medicine, Universitas Sumatera Utara; Institute of Tropical Disease, Universitas Airlangga	Aldise M Nastri; Franciscus Ginting; Inke N D Lubis; Irbah R Nainggolan; Jezzy R Dewantari; Kazufumi Shimizu; Krisnoadi Rahardjo; Maria I Lusida; Meliani; Mirzan Hasibuan; Muhammad Ichwan; R Andika D Cahyadi; R Lia Kusumawati; Ramadhan Bestari; Rima R Prasetya; Soetjipto; Yasuko Mori
EPI_ISL_1196007	Mbabane Gov Hospital	National Institute for Communicable Diseases of the National Health Laboratory Service	Amoako DG; Bhiman JN; Ismail A; Mahlangu B; Maphalala GP; Mohale T; Ntuli N; Scheepers C
EPI_ISL_456377	MedLab Central Ltd	Institute of Environmental Science and Research (ESR)	Anja Werno; Antje van der Linden; Arlo Upton; Chris Mansell; David Hammer; Dragana Drinkovic; Erasmus Smit; Gary McAuliffe; Hana Sofia Andersson; James Ussher; Jill Sherwood; Joep de Ligt; Josh Freeman; Julia Howard; Juliet Elvy; Lauren Jelly; Mary DeAlmeida; Matt Blakiston; Matt Storey; Matthew Rogers; Max Bloomfield; Michael Addidle; Michelle Balm; Sally Roberts; Sarah Jefferies; Sharmini Muttaiyah; Susan Morpeth; Susan Taylor; Timothy Blackmore; Vani Sathyendran; Veronica Playle; Virginia Hope; Xiaoyun Ren
EPI_ISL_482759, EPI_ISL_482760, EPI_ISL_482762, EPI_ISL_482763, EPI_ISL_482765, EPI_ISL_482766, EPI_ISL_482770, EPI_ISL_482771, EPI_ISL_482772, EPI_ISL_482773, EPI_ISL_482774, EPI_ISL_483035, EPI_ISL_483038, EPI_ISL_1109484, EPI_ISL_1109486, EPI_ISL_1109625, EPI_ISL_1109626, EPI_ISL_1109628, EPI_ISL_1109629, EPI_ISL_1109630, EPI_ISL_1165078, EPI_ISL_1165080, EPI_ISL_1165082, EPI_ISL_1167193			
see above	Medical Ain Shams Research Institute (MASRI), Ain Shams University	Medical Ain Shams Research Institute (MASRI), Ain Shams University	Ahmad Moustafa; Ashraf Omar; Aya Mohamed; Fatma Ebied; Ghada Ismael; Hagar Elshora; Hala Hafez; Hesham Elghazaly; Hoda Ezz Elarab; Iman Foda; Mahmoud Elmeitini; Manal Hamdy Elsaid; Mohamed Elhadidi; Osama Mansour; Osama Mansour.; Reham Kassab; Reham Mamdouh; Samia Abdou Girgis; Sara Elnakeep; Sara Hassan Agwa; Shaimaa Moustafa; Shimaa Moustafa
EPI_ISL_495535, EPI_ISL_495536, EPI_ISL_495537, EPI_ISL_495538, EPI_ISL_495539, EPI_ISL_495540, EPI_ISL_495541, EPI_ISL_495542, EPI_ISL_515802, EPI_ISL_515803, EPI_ISL_515804, EPI_ISL_515805, EPI_ISL_515806, EPI_ISL_515807, EPI_ISL_515808, EPI_ISL_515809, EPI_ISL_515810, EPI_ISL_515811, EPI_ISL_515812, EPI_ISL_515813, EPI_ISL_515814, EPI_ISL_515815, EPI_ISL_515816, EPI_ISL_515817, EPI_ISL_515818, EPI_ISL_515819, EPI_ISL_515820, EPI_ISL_515822, EPI_ISL_515823, EPI_ISL_515825, EPI_ISL_515826, EPI_ISL_515829, EPI_ISL_515830, EPI_ISL_515831, EPI_ISL_515832, EPI_ISL_515833, EPI_ISL_515835, EPI_ISL_515836, EPI_ISL_515837, EPI_ISL_515838, EPI_ISL_515839, EPI_ISL_515840, EPI_ISL_515841, EPI_ISL_515842, EPI_ISL_515843, EPI_ISL_515844, EPI_ISL_515845, EPI_ISL_515846, EPI_ISL_515847, EPI_ISL_515848, EPI_ISL_515849, EPI_ISL_515850, EPI_ISL_515851, EPI_ISL_515852, EPI_ISL_515853, EPI_ISL_515858, EPI_ISL_515859, EPI_ISL_515860, EPI_ISL_515862, EPI_ISL_515863, EPI_ISL_515865, EPI_ISL_515866, EPI_ISL_515867, EPI_ISL_515868, EPI_ISL_515870, EPI_ISL_515872, EPI_ISL_515874, EPI_ISL_515875, EPI_ISL_515877, EPI_ISL_515878, EPI_ISL_515881, EPI_ISL_515882, EPI_ISL_515883, EPI_ISL_515885, EPI_ISL_515886, EPI_ISL_515888, EPI_ISL_515890, EPI_ISL_515891, EPI_ISL_515892, EPI_ISL_515893			
see above	Medical Disagnostics Services (MDS)	KRISP, KZN Research Innovation and Sequencing Platform	Chimukangara B; ChimukangaraB; Giandhari J; Khan S; Lessells R; Mdlalose K; Pillay S; Tegally H; Wilkinson E; York D; de Oliveira T
EPI_ISL_873164, EPI_ISL_873165	Medical Laboratory Sciences, Arab American University	Medical Laboratory Sciences, Arab American University	Al-Jawabreh, A.; Al-Jawabreh, H.; Dumaidi, k.; Ereqat, S.; Nasereddin, A.
EPI_ISL_1251601	Medical Microbiology Unit, Department for Laboratory Medicine, Drammen Hospital, Vestre Viken Health Trust,	Norwegian Institute of Public Health, Department of Virology	Atiya R Ali; Debech Nadia; Engebretsen Serina Beate; Garcia Llorente Ignacio; Hilde Elshaug; Hilde Vollan; Jon Bråte; Kamilla Heddeland Instefjord; Karoline Bragstad; Kathrine Stene-Johansen; Marie Paulsen Madsen; Olav Hungnes; Pedersen Benedikte Nevjen; Rasmus Riis Kopperud
EPI_ISL_806725	Medlab Pathology	NSW Health Pathology - Institute of Clinical Pathology and Medical Research; Westmead Hospital; University of Sydney	CIDM-PH et al.
EPI_ISL_1048371	Megalab, Molecular and Cytogenetics Diagnostics	Department for Virology, Molecular Biology and Genome Research, R. G. Lugar Center for Public Health Research, National Center for Disease Control and Public Health (NCDC) of Georgia.	Adam Kotorashvili; Amiran Gamkrelidze. Submitter information; Ana Papkiauri; Ann Machablishvili; Anna Kasradze; Davit Tsaguria; Ekaterine Khmaladze; Ekaterine Zangaladze; Ekaterine Zhghenti; Giorgi Gogoladze; Giorgi Tomashvili; Gvantsa Brachveli; Gvantsa Chanturia; Irma Burjanadze; Ketevan Sidamonidze; Khatuna Zakhashvili; Lela Sabadze; Lela Urushadze; Magda Dgebuadze; Maia Alkhazashvili; Mari Gavashelidze; Mariam Zakalashvili; Marine Murtskhvaladze; Meri Pantsulaia; Nato Kotaria; Nino Berishvili; Paata Imnadze; Roena Sukhiashvili; Tamar Jashiashvili; Tata Imnadze; Tea Tevdoradze
EPI_ISL_593186, EPI_ISL_593457, EPI_ISL_663785, EPI_ISL_779642, EPI_ISL_812426, EPI_ISL_877581, EPI_ISL_962827, EPI_ISL_979361, EPI_ISL_1033151			
see above	Microbiological Diagnostic Unit - Public Health Laboratory (MDU-PHL)	MDU-PHL	M. B.; M.L.; N.L.; Sait; Sait, M.; Schultz; Schultz M.B.; Seemann T.; Sherry; Sherry, N.
EPI_ISL_1208558	Microbiology Department, Laboratori Clínic Metropolitana Nord. Hospital Universitari Germans Trias i Pujol.	Can Ruti SARS-CoV-2 Sequencing Hub (HUGTiP/IrsiCaixa/IGTP)	Alba Sánchez; Anna Not; Antoni E Bordoy; Bonaventura Clotet; Carol Galvez Maria Casadellà; Cristina Casañ; Cristina Esteban; Francesc Catala-Moll; Gemma Clara; Ignacio Blanco; Irina Pey; Jordi Barretina; Julia G Prado; Marc Noguera-Julian; Mariona Parera; Mercedes Guerrero; Montserrat Giménez; Pere-Joan Cardona; Pilar Armengol; Roger Paredes; Verónica Saludes; and Elisa Martró on behalf of the Can Ruti SARS-CoV-2 Sequencing Hub.
EPI_ISL_447282	Microbiology Division, Barzilai University Medical Center	Stern Lab	Stern Lab
EPI_ISL_548132, EPI_ISL_755624, EPI_ISL_794624, EPI_ISL_1016857, EPI_ISL_1016865, EPI_ISL_1250702	Middlemore Hospital	Institute of Environmental Science and Research (ESR)	Anja Werno; Antje van der Linden; Arlo Upton; Chris Mansell; David Hammer; Dragana Drinkovic; Erasmus Smit; Gary McAuliffe; Hana Sofia Andersson; Hermes Perez; James Ussher; Jill Sherwood; Jing Wang; Joep de Ligt; Josh Freeman; Julia Howard; Juliet Elvy; Lauren Jelly; Mary DeAlmeida; Matt Blakiston; Matt Storey; Matthew Rogers; Max Bloomfield; Michael Addidle; Michelle Balm; Muhammad Faisal; Nikki Freed; Olin Silander; Olivia Stroeven; Rachel Boyle; Sally Roberts; SallyAnn Harbison; Sarah Jefferies; Sharmini Muttaiyah; Susan Morpeth; Susan Taylor; Timothy Blackmore; Vani Sathyendran; Veronica Playle; Virginia Hope; Xiaoyun Ren
EPI_ISL_1318211, EPI_ISL_1318217	Ministry of Health Hospitals	Institute of Health and Community Medicine	Chan Chia Jui; Chua Hock Hin; David Perera; Ooi Mong How; Tonnii Sia Loong Loong; Wong Jyn Shan; Wong Kiing Aik
EPI_ISL_429861, EPI_ISL_812876, EPI_ISL_812878,	Ministry of Health Turkey	Ministry of Health Turkey	Ayşe Başak Altaş; Fatma Bayrakdar; Gulay Korukluoglu; Gülay Korukluoğlu; Selçuk Kılıç; Suleyman Yalcin; Süleyman Yalcin; Yasemin Cosgun; Yasemin Coşgun

EPI_ISL_812895, EPI_ISL_812907, EPI_ISL_1534466			
EPI_ISL_530191, EPI_ISL_576234, EPI_ISL_1593689, EPI_ISL_1593702	Minnesota Department of Health, Public Health Laboratory	Minnesota Department of Health, Public Health Laboratory	Alexandra Lorentz; Jacob Garfin; Matt Plumb; and Xiong Wang
EPI_ISL_1362729, EPI_ISL_1362871, EPI_ISL_1489851	Missouri State Public Health Laboratory	Missouri State Public Health Laboratory	Ashley New; Joshua Barry; Matthew Sinn
EPI_ISL_640020, EPI_ISL_640028, EPI_ISL_640065, EPI_ISL_700489, EPI_ISL_700597, EPI_ISL_1040794	Mitchells Plain Hospital wc MPH	NHLS/UCT	Arash Iranzadeh; Bruna Galvao; Carolyn Williamson; Deelan Doolabh; Diana Hardie; Innocent Mudau; Kruger Marais; Lynn Tyers; Marvin Hsiao; Stephen Korsman
EPI_ISL_1634442	Mitra Kasih Hospital	West Java Health Laboratory; School of Life Sciences and Technology, Institut Teknologi Bandung	Aulia Saraswati Wicaksono; Azzania Fibriani; Cut Nur Cinthia Alamanda; Ema Rahmawati; Karimatu Khoirunnisa; Miftahul Faridl; Rifky Waluyajati Rachman; Rini Robiani; Ryan Bayusantika Ristandi
EPI_ISL_435121	Mohammed Bin Rashid University of Medicine and Health Sciences	Al Jalila Genomics Center	Abdulmajeed Alkhaja; Abiola Catherine Senok; Ahmad Abou Tayoun; Alawi Alsheikh-Ali; Divinlal Harilal; Hamda Khansaheb; Hanan Al Suwaidi; Mohammed Uddin; Norbert Nowotny; Qutayba Hamid; Rabih Halwani; Rifat Hamoudi; Rupa Murthy Varghese; Sathishkumar Ramaswamy; Tom Loney; Zulfa Omar Deesi
EPI_ISL_660259, EPI_ISL_660261, EPI_ISL_660262, EPI_ISL_660263	Molecualr Diagnostic Services (MDS)	KRISP, KZN Research Innovation and Sequencing Platform	Giandhari J; Khan S; Lessells R; Mdlalose K; Pillay S; Tegally H; Wilkinson E; York D; de Oliveira T
EPI_ISL_467445, EPI_ISL_467475, EPI_ISL_467477, EPI_ISL_467478, EPI_ISL_467479, EPI_ISL_467481, EPI_ISL_467482, EPI_ISL_467483, EPI_ISL_467484, EPI_ISL_467485, EPI_ISL_467486, EPI_ISL_467488, EPI_ISL_467490, EPI_ISL_467491, EPI_ISL_467494, EPI_ISL_467498, EPI_ISL_467499, EPI_ISL_467500, EPI_ISL_467502, EPI_ISL_467503, EPI_ISL_482705, EPI_ISL_482708, EPI_ISL_482709, EPI_ISL_482720, EPI_ISL_482722, EPI_ISL_482852, EPI_ISL_482853, EPI_ISL_482867, EPI_ISL_487332, EPI_ISL_487338, EPI_ISL_487340, EPI_ISL_1250542			
see above	Molecular Diagnostics Services (MDS)	KRISP, KZN Research Innovation and Sequencing Platform	Chimukangara B; Emmanuel SJ; Giandhari J; Khan S; Lessells R; Maslo C; Mdlalose K; Pillay S; Sitharam L; Tegally H; Wilkinson E; York D; de Oliveira T
EPI_ISL_895831	Molecular biology division, Institute of Clinical Biochemistry and Diagnostics, Charles University, Faculty of Medicine in Hradec Králové and University Hospital Hradec Králové	Molecular biology division, Institute of Clinical Biochemistry and Diagnostics, Charles University, Faculty of Medicine in Hradec Králové and University Hospital Hradec Králové	Helena Kovaříková; Ivana Baranová; Jitka Novotná; Kateřina Hrochová; Kateřina Pehlíková; Petr Brož; Tereza Baťková; Vladimír Palička. Cooperation project with BioVendor-R&D and bioinformatics company BIOXSYS s r.o.
EPI_ISL_1510612	Molecular diagnostic laboratory of Federal Budget Institution of Science "Central Research Institute of Epidemiology" of The Federal Service on Customers' Rights Protection and Human Well-being Surveillance	Group of Genomics and Postgenomic Technologies of Central Research Institute of Epidemiology	Akimkin VG; Berlina YY; Bulanenko VP; Cherkashina AS; Golubeva AG; Kaptelova VV; Kondrasheva LY; Korneenko EV; Saenko SS; Samoilov AE; Shipulina OY; Solovyeva ED; Speranskaya AS; Tivanova EV; Valdokhina AV; Zotova MI
EPI_ISL_1363114	Molecular diagnostic laboratory of Federal Budget Institution of Science "Central Research Institute of Epidemiology" of The Federal Service on Customers' Rights Protection and Human Well-being Surveillance	Group of Genomics and Postgenomic Technologies of Central Research Institute of Epidemiology	Akimkin VG; Berlina YY; Bulanenko VP; Cherkashina AS; Golubeva AG; Kaptelova VV; Kondrasheva LY; Korneenko EV; Saenko SS; Samoilov AE; Shipulina OY; Solovyeva ED; Speranskaya AS; Tivanova EV; Valdokhina AV; Zotova MI
EPI_ISL_1365024, EPI_ISL_1365025, EPI_ISL_1367678, EPI_ISL_1367692	Molecular diagnostic unit for viral haemorrhagic fevers and emerging viruses, Bouaké CHU Laboratory	Molecular diagnostic unit for viral haemorrhagic fevers and emerging viruses, Bouaké CHU Laboratory	Adjaratou Traoré; Bamba Fatoumata Touré; Chantal Akoua-Koffi; Coulibaly Mbegnan; Diané Bamourou; Essia Belarbi; Etilé Anoh; Fabian Leendertz; Grit Schubert; Kra Ouffoué; Monemo Pacome; Oby Wayoro; Safiatou Karidioula; Soundélé Maïté
EPI_ISL_614347, EPI_ISL_614348, EPI_ISL_614349, EPI_ISL_614351, EPI_ISL_614352, EPI_ISL_614353, EPI_ISL_614354, EPI_ISL_614355, EPI_ISL_614356, EPI_ISL_614357, EPI_ISL_614358, EPI_ISL_614359, EPI_ISL_614362, EPI_ISL_614363, EPI_ISL_614364, EPI_ISL_614365, EPI_ISL_614366, EPI_ISL_614368, EPI_ISL_614371, EPI_ISL_614376, EPI_ISL_614379, EPI_ISL_614384, EPI_ISL_614386, EPI_ISL_614388, EPI_ISL_614391, EPI_ISL_614393, EPI_ISL_681829, EPI_ISL_681834, EPI_ISL_681835, EPI_ISL_681836, EPI_ISL_681840			
see above	Molecular diagnostic unit for viral haemorrhagic fevers and emerging viruses, Bouaké CHU Laboratory	Project group Epidemiology of Highly Pathogenic Microorganisms, Robert Koch-Institute	Adjaratou Traoré; Bamba Fatoumata Touré; Chantal Akoua-Koffi; Coulibaly Mbegnan; Diané Bamourou; Essia Belarbi; Etilé Anoh; Fabian Leendertz; Grit Schubert; Kra Ouffoué; Monemo Pacome; Safiatou Karidioula; Soundélé Maïté
EPI_ISL_406844	Monash Medical Centre	Collaboration between the University of Melbourne at The Peter Doherty Institute for Infection and Immunity, and the Victorian Infectious Disease Reference Laboratory	Caly, L.; Druce; G; J. and Taiaroa; Schultz, M.; Seemann, T.
EPI_ISL_1291752, EPI_ISL_1576811, EPI_ISL_1577329	Montana Public Health Laboratory	Montana Public Health Laboratory	Carrie Biskupiak; Deborah Gibson; Joy Ritter; Michelle Mozer
EPI_ISL_700479	Mowbray Maternity Hospital wc MMH	NHLS/UCT	Arash Iranzadeh; Bruna Galvao; Carolyn Williamson; Deelan Doolabh; Diana Hardie; Innocent Mudau; Kruger Marais; Lynn Tyers; Marvin Hsiao; Stephen Korsman
EPI_ISL_1591098, EPI_ISL_1593728	NAMRU-6	Pathogen Discovery, Respiratory Viruses Branch, Division of Viral Diseases, Centers for Disease Control and Prevention	Adam Retchless; Anna Kelleher; Anna Montmayeur; Anna Uehara; Brian Lynch; Clinton R. Paden; Haibin Wang; Han Jia Justin Ng; Jing Zhang; Justin Lee; Krista Queen; Mark Burroughs; Peter Cook; Rachel Marine; Suxiang Tong; Yan Li; Ying Tao
EPI_ISL_1272260, EPI_ISL_1272263	NB-Hôpital Georges L. Dumont	National Microbiology Laboratory (NML)	Anna Majer; Anneliese Landgraff; CanCOGeN's metadata curation team; Darian Hole; Elsie Grudeski; Gary Van Domselaar; Grace Seo; Guillaume Desnoyers; Jennifer Tanner; Kirsten Biggar; Madison Chapel; Morag Graham; Natalie Knox; Nathalie Bastien; Philip Mabon; Public Health Agency of Canada CanCOGeN team; Rhiannon Huzarewich; Richard Garceau; Russell Mandes; Shari Tyson; Timothy Booth; Yan Li
EPI_ISL_424871	NC State Laboratory of Public Health	Pathogen Discovery, Respiratory Viruses Branch, Division of Viral Diseases, Centers for Disease Control and Prevention	Alison S. Laufer Halpin; Anna Uehara; Christopher A. Elkins; Clinton R. Paden; Haibin Wang; Jing Zhang; Krista Queen; Mary S. Keckler; Rachel Marine; Suxiang Tong; Yan Li; Ying Tao
EPI_ISL_1446985	ND Dept. of Health Laboratory Services-Microbiology	Centers for Disease Control and Prevention Division of Viral Diseases, Pathogen Discovery	Alison Laufer Halpin; Ben L. Rambo-Martin; Clinton R. Paden; Dakota Howard; Darlene Wagner; Dave Wentworth; Dhwani Batra; Jasmine Padilla; Justin Lee; Katie Dillon; Krista Queen; Kristen Knipe; Kristine Lacek; Mark Burroughs; Matthew Schmerer; Mili Sheth; Peter Cook; Sam Shepard; Sarah Nobles; Shoshona Le; Suxiang Tong; Vivien Dugan; Yvette Unoarumhi
EPI_ISL_682323, EPI_ISL_682324, EPI_ISL_682325, EPI_ISL_682335, EPI_ISL_682341, EPI_ISL_682347, EPI_ISL_682351, EPI_ISL_912535			
see above	NHLS Universitas Academic	UFS Virology	D Goedhals; MM Nyaga; MT Mogotsi; P Nthiga; PA Bester; T de Oliveira
EPI_ISL_467437, EPI_ISL_467493, EPI_ISL_467507, EPI_ISL_467508, EPI_ISL_467518, EPI_ISL_467519, EPI_ISL_482710, EPI_ISL_482712, EPI_ISL_482726, EPI_ISL_487277, EPI_ISL_487288, EPI_ISL_487297, EPI_ISL_487304, EPI_ISL_487308, EPI_ISL_487314, EPI_ISL_487316, EPI_ISL_487319, EPI_ISL_487320, EPI_ISL_487321, EPI_ISL_487324, EPI_ISL_495529, EPI_ISL_495530, EPI_ISL_495534, EPI_ISL_495546, EPI_ISL_495550, EPI_ISL_495557, EPI_ISL_495562, EPI_ISL_498055, EPI_ISL_498061, EPI_ISL_498062, EPI_ISL_498065, EPI_ISL_498066, EPI_ISL_498067, EPI_ISL_498069, EPI_ISL_498071, EPI_ISL_498072, EPI_ISL_498073, EPI_ISL_498074, EPI_ISL_498075, EPI_ISL_498077, EPI_ISL_498078, EPI_ISL_498079, EPI_ISL_498081, EPI_ISL_498082, EPI_ISL_498084, EPI_ISL_498086, EPI_ISL_498087, EPI_ISL_498088, EPI_ISL_498089, EPI_ISL_498090, EPI_ISL_498094, EPI_ISL_498095, EPI_ISL_498096, EPI_ISL_498098, EPI_ISL_498099, EPI_ISL_498104, EPI_ISL_498106, EPI_ISL_498108, EPI_ISL_498109, EPI_ISL_498110, EPI_ISL_498111, EPI_ISL_498112, EPI_ISL_498113, EPI_ISL_498115, EPI_ISL_498117, EPI_ISL_498118, EPI_ISL_498119, EPI_ISL_498122, EPI_ISL_498123, EPI_ISL_498124, EPI_ISL_498125, EPI_ISL_498126, EPI_ISL_509224, EPI_ISL_509225, EPI_ISL_509226, EPI_ISL_509228, EPI_ISL_509229, EPI_ISL_509230, EPI_ISL_509231, EPI_ISL_509232, EPI_ISL_509235, EPI_ISL_509236, EPI_ISL_509237, EPI_ISL_509238, EPI_ISL_509239, EPI_ISL_509240, EPI_ISL_509241, EPI_ISL_509242, EPI_ISL_509243, EPI_ISL_509244, EPI_ISL_509247, EPI_ISL_509248, EPI_ISL_509249, EPI_ISL_509250, EPI_ISL_509251, EPI_ISL_509252, EPI_ISL_509253, EPI_ISL_509254, EPI_ISL_509255, EPI_ISL_509256, EPI_ISL_509257, EPI_ISL_509260, EPI_ISL_509264, EPI_ISL_509265, EPI_ISL_509267, EPI_ISL_509269, EPI_ISL_509275, EPI_ISL_509283, EPI_ISL_509285, EPI_ISL_509287, EPI_ISL_509289, EPI_ISL_509290, EPI_ISL_509293, EPI_ISL_509294, EPI_ISL_509296, EPI_ISL_509299, EPI_ISL_509300, EPI_ISL_509302, EPI_ISL_509303, EPI_ISL_509304, EPI_ISL_509305, EPI_ISL_509306, EPI_ISL_509307, EPI_ISL_509308, EPI_ISL_509309, EPI_ISL_509310, EPI_ISL_509313, EPI_ISL_509314, EPI_ISL_509316, EPI_ISL_509317, EPI_ISL_509318, EPI_ISL_509319, EPI_ISL_509320, EPI_ISL_509321, EPI_ISL_509323, EPI_ISL_509324, EPI_ISL_509325, EPI_ISL_509326, EPI_ISL_509327, EPI_ISL_509328, EPI_ISL_509329, EPI_ISL_509330, EPI_ISL_509331, EPI_ISL_509332, EPI_ISL_509333, EPI_ISL_509334, EPI_ISL_509335, EPI_ISL_509337, EPI_ISL_509338, EPI_ISL_509339, EPI_ISL_509340, EPI_ISL_509342, EPI_ISL_509345, EPI_ISL_509346, EPI_ISL_509347, EPI_ISL_509348, EPI_ISL_509350, EPI_ISL_509352, EPI_ISL_509353, EPI_ISL_509354, EPI_ISL_509357, EPI_ISL_509358, EPI_ISL_509361, EPI_ISL_509362, EPI_ISL_509363, EPI_ISL_509364, EPI_ISL_509365, EPI_ISL_509367, EPI_ISL_509368, EPI_ISL_509369, EPI_ISL_515568, EPI_ISL_515573, EPI_ISL_515574, EPI_ISL_515575, EPI_ISL_515580, EPI_ISL_515584, EPI_ISL_515585, EPI_ISL_515586, EPI_ISL_515592, EPI_ISL_515593, EPI_ISL_515594, EPI_ISL_515596, EPI_ISL_515601, EPI_ISL_515604, EPI_ISL_515606, EPI_ISL_515607, EPI_ISL_515610, EPI_ISL_515619, EPI_ISL_515622, EPI_ISL_515623, EPI_ISL_515627, EPI_ISL_515628, EPI_ISL_515631, EPI_ISL_515634, EPI_ISL_515639, EPI_ISL_515641, EPI_ISL_515642, EPI_ISL_515644, EPI_ISL_515645, EPI_ISL_515647, EPI_ISL_515650, EPI_ISL_515651, EPI_ISL_515654, EPI_ISL_515655, EPI_ISL_515658, EPI_ISL_515659, EPI_ISL_515660, EPI_ISL_515661, EPI_ISL_515662, EPI_ISL_515665, EPI_ISL_515666, EPI_ISL_515668, EPI_ISL_515669, EPI_ISL_515671, EPI_ISL_515674, EPI_ISL_515675, EPI_ISL_515676, EPI_ISL_515678, EPI_ISL_515680, EPI_ISL_515681, EPI_ISL_515682, EPI_ISL_515683, EPI_ISL_515684, EPI_ISL_515687, EPI_ISL_515689, EPI_ISL_515691, EPI_ISL_515694, EPI_ISL_515697, EPI_ISL_515699, EPI_ISL_515701, EPI_ISL_515702, EPI_ISL_515703, EPI_ISL_515705, EPI_ISL_515707, EPI_ISL_515708, EPI_ISL_515710, EPI_ISL_515711, EPI_ISL_515714, EPI_ISL_515716, EPI_ISL_515719, EPI_ISL_515720, EPI_ISL_515721, EPI_ISL_515722, EPI_ISL_515724, EPI_ISL_515725, EPI_ISL_515728, EPI_ISL_515729, EPI_ISL_515730, EPI_ISL_515731, EPI_ISL_515732, EPI_ISL_515733, EPI_ISL_515737, EPI_ISL_515739, EPI_ISL_515740, EPI_ISL_515743, EPI_ISL_515744, EPI_ISL_515748, EPI_ISL_515749, EPI_ISL_515752, EPI_ISL_515753, EPI_ISL_515754, EPI_ISL_515757, EPI_ISL_515760, EPI_ISL_515762, EPI_ISL_515763, EPI_ISL_515764, EPI_ISL_515767, EPI_ISL_515769, EPI_ISL_515771, EPI_ISL_515773, EPI_ISL_515775, EPI_ISL_515777, EPI_ISL_515778, EPI_ISL_515780, EPI_ISL_515781, EPI_ISL_515790, EPI_ISL_515791, EPI_ISL_515794, EPI_ISL_518034, EPI_ISL_518035, EPI_ISL_518042, EPI_ISL_518043, EPI_ISL_518044, EPI_ISL_518045, EPI_ISL_518047, EPI_ISL_518048, EPI_ISL_518049, EPI_ISL_518050, EPI_ISL_518051, EPI_ISL_518052, EPI_ISL_529720, EPI_ISL_529721, EPI_ISL_529723, EPI_ISL_529724, EPI_ISL_529726, EPI_ISL_529728, EPI_ISL_529730, EPI_ISL_529731, EPI_ISL_529732, EPI_ISL_529734, EPI_ISL_529735, EPI_ISL_529736, EPI_ISL_529737, EPI_ISL_529739, EPI_ISL_529740, EPI_ISL_529741, EPI_ISL_529742, EPI_ISL_529743, EPI_ISL_529744, EPI_ISL_529747, EPI_ISL_529748, EPI_ISL_529749, EPI_ISL_529750, EPI_ISL_529752, EPI_ISL_529755, EPI_ISL_529757, EPI_ISL_529758, EPI_ISL_529760, EPI_ISL_529763, EPI_ISL_529765, EPI_ISL_529766, EPI_ISL_529767, EPI_ISL_529769, EPI_ISL_529770, EPI_ISL_529771, EPI_ISL_529772, EPI_ISL_529774, EPI_ISL_529776, EPI_ISL_529777, EPI_ISL_529778, EPI_ISL_529779, EPI_ISL_529782, EPI_ISL_529783, EPI_ISL_529784, EPI_ISL_529786, EPI_ISL_529787, EPI_ISL_529788, EPI_ISL_529789, EPI_ISL_529790, EPI_ISL_529791, EPI_ISL_529792, EPI_ISL_529794, EPI_ISL_529796, EPI_ISL_529797, EPI_ISL_529798, EPI_ISL_529799, EPI_ISL_529801, EPI_ISL_529802, EPI_ISL_535390, EPI_ISL_535392, EPI_ISL_535393, EPI_ISL_535394, EPI_ISL_535395, EPI_ISL_535396, EPI_ISL_535397, EPI_ISL_535398, EPI_ISL_535399, EPI_ISL_535400, EPI_ISL_535403, EPI_ISL_535404, EPI_ISL_535405, EPI_ISL_535406, EPI_ISL_535408, EPI_ISL_535410, EPI_ISL_535412, EPI_ISL_535413, EPI_ISL_535414, EPI_ISL_535415, EPI_ISL_535416, EPI_ISL_535420, EPI_ISL_535421, EPI_ISL_535422, EPI_ISL_535426, EPI_ISL_535427, EPI_ISL_535428, EPI_ISL_535430, EPI_ISL_535431, EPI_ISL_535433, EPI_ISL_535434, EPI_ISL_535436, EPI_ISL_535438, EPI_ISL_535440, EPI_ISL_535442, EPI_ISL_535443, EPI_ISL_535444, EPI_ISL_535445, EPI_ISL_535446, EPI_ISL_535447, EPI_ISL_535449, EPI_ISL_535450, EPI_ISL_535451, EPI_ISL_535452, EPI_ISL_535456, EPI_ISL_535457, EPI_ISL_535459, EPI_ISL_535460, EPI_ISL_535461, EPI_ISL_535462, EPI_ISL_535463, EPI_ISL_535465, EPI_ISL_535466, EPI_ISL_535468, EPI_ISL_535469, EPI_ISL_535470, EPI_ISL_535472, EPI_ISL_535473, EPI_ISL_535475, EPI_ISL_535476, EPI_ISL_535477, EPI_ISL_535478, EPI_ISL_535479, EPI_ISL_535481, EPI_ISL_535483, EPI_ISL_535484, EPI_ISL_535485, EPI_ISL_535487, EPI_ISL_535488, EPI_ISL_535496, EPI_ISL_535498, EPI_ISL_535500, EPI_ISL_535501, EPI_ISL_535503, EPI_ISL_535504, EPI_ISL_535505, EPI_ISL_535506, EPI_ISL_535510, EPI_ISL_535511, EPI_ISL_535512, EPI_ISL_535513, EPI_ISL_535514, EPI_ISL_535516, EPI_ISL_535518, EPI_ISL_535520, EPI_ISL_535522, EPI_ISL_535523, EPI_ISL_535529, EPI_ISL_535531, EPI_ISL_535533, EPI_ISL_535536, EPI_ISL_535537, EPI_ISL_535539, EPI_ISL_535541, EPI_ISL_535542, EPI_ISL_535543, EPI_ISL_535544, EPI_ISL_535545, EPI_ISL_535546, EPI_ISL_535551, EPI_ISL_535552, EPI_ISL_535553, EPI_ISL_535554, EPI_ISL_535555, EPI_ISL_535556, EPI_ISL_535557, EPI_ISL_535558, EPI_ISL_535559, EPI_ISL_535562, EPI_ISL_535563, EPI_ISL_535565, EPI_ISL_535566, EPI_ISL_535567, EPI_ISL_535568, EPI_ISL_535569, EPI_ISL_535570, EPI_ISL_602632, EPI_ISL_602633, EPI_ISL_602634, EPI_ISL_602635, EPI_ISL_602636, EPI_ISL_602637, EPI_ISL_602639, EPI_ISL_602640, EPI_ISL_602641, EPI_ISL_602644, EPI_ISL_602646, EPI_ISL_602647, EPI_ISL_602648, EPI_ISL_602650, EPI_ISL_602651, EPI_ISL_602652, EPI_ISL_602653, EPI_ISL_602654, EPI_ISL_602656, EPI_ISL_602657, EPI_ISL_602658, EPI_ISL_602660, EPI_ISL_602661, EPI_ISL_602663, EPI_ISL_602664, EPI_ISL_602665, EPI_ISL_602666, EPI_ISL_602667, EPI_ISL_602668, EPI_ISL_602670, EPI_ISL_602672, EPI_ISL_602673, EPI_ISL_602674, EPI_ISL_602675, EPI_ISL_602676, EPI_ISL_602678, EPI_ISL_602679, EPI_ISL_602680, EPI_ISL_602681, EPI_ISL_602682, EPI_ISL_602683, EPI_ISL_602685, EPI_ISL_602686, EPI_ISL_602687, EPI_ISL_602690, EPI_ISL_602692, EPI_ISL_602693, EPI_ISL_602694, EPI_ISL_602695, EPI_ISL_602696, EPI_ISL_602697, EPI_ISL_602698, EPI_ISL_602700, EPI_ISL_602701, EPI_ISL_602703, EPI_ISL_602704, EPI_ISL_602706, EPI_ISL_602707, EPI_ISL_602708, EPI_ISL_602709, EPI_ISL_602710, EPI_ISL_602711, EPI_ISL_602712, EPI_ISL_602713, EPI_ISL_602714, EPI_ISL_602715, EPI_ISL_602716, EPI_ISL_602717, EPI_ISL_602718, EPI_ISL_602719, EPI_ISL_602720, EPI_ISL_602721, EPI_ISL_602722, EPI_ISL_602724, EPI_ISL_602725, EPI_ISL_602726, EPI_ISL_602727, EPI_ISL_602728, EPI_ISL_602729, EPI_ISL_602730, EPI_ISL_602731, EPI_ISL_602732, EPI_ISL_602733, EPI_ISL_602735, EPI_ISL_602737, EPI_ISL_602739, EPI_ISL_602740, EPI_ISL_602741, EPI_ISL_602742, EPI_ISL_602743, EPI_ISL_602744, EPI_ISL_602745, EPI_ISL_602747, EPI_ISL_602748, EPI_ISL_602749, EPI_ISL_602750, EPI_ISL_602751, EPI_ISL_602752, EPI_ISL_602753, EPI_ISL_602754, EPI_ISL_602755, EPI_ISL_602760, EPI_ISL_602761, EPI_ISL_602762, EPI_ISL_602763, EPI_ISL_602766, EPI_ISL_602767, EPI_ISL_602768, EPI_ISL_602769, EPI_ISL_602770, EPI_ISL_602771, EPI_ISL_602772, EPI_ISL_602775, EPI_ISL_602776, EPI_ISL_602778, EPI_ISL_602779, EPI_ISL_602780, EPI_ISL_602783, EPI_ISL_602784, EPI_ISL_602787, EPI_ISL_602788, EPI_ISL_602789, EPI_ISL_602790, EPI_ISL_602796, EPI_ISL_602797, EPI_ISL_602798, EPI_ISL_602799, EPI_ISL_602801, EPI_ISL_602803, EPI_ISL_602804, EPI_ISL_602806, EPI_ISL_602807, EPI_ISL_602808, EPI_ISL_602809, EPI_ISL_602810, EPI_ISL_602811, EPI_ISL_602813, EPI_ISL_602817, EPI_ISL_602818, EPI_ISL_602820, EPI_ISL_602822, EPI_ISL_602824, EPI_ISL_602826, EPI_ISL_602831, EPI_ISL_602832, EPI_ISL_602834, EPI_ISL_602836, EPI_ISL_602837, EPI_ISL_602838, EPI_ISL_602839, EPI_ISL_602840, EPI_ISL_602841, EPI_ISL_602842, EPI_ISL_602843, EPI_ISL_602844, EPI_ISL_602848, EPI_ISL_602849, EPI_ISL_602850, EPI_ISL_602851, EPI_ISL_602853, EPI_ISL_602854, EPI_ISL_602855, EPI_ISL_602860, EPI_ISL_602861, EPI_ISL_602863, EPI_ISL_602864, EPI_ISL_602866, EPI_ISL_602867, EPI_ISL_602870, EPI_ISL_602871, EPI_ISL_602875, EPI_ISL_602877, EPI_ISL_602879, EPI_ISL_602881, EPI_ISL_602883, EPI_ISL_602887, EPI_ISL_602890, EPI_ISL_602892, EPI_ISL_602896, EPI_ISL_602899, EPI_ISL_602900, EPI_ISL_602902, EPI_ISL_602906, EPI_ISL_602907, EPI_ISL_602908, EPI_ISL_602911, EPI_ISL_602913, EPI_ISL_602916, EPI_ISL_602917, EPI_ISL_602919, EPI_ISL_602920, EPI_ISL_602923, EPI_ISL_602928, EPI_ISL_602929, EPI_ISL_605785, EPI_ISL_605786, EPI_ISL_605787, EPI_ISL_660164, EPI_ISL_660165, EPI_ISL_660166, EPI_ISL_660168, EPI_ISL_660171, EPI_ISL_660172, EPI_ISL_660174, EPI_ISL_660175, EPI_ISL_660189, EPI_ISL_660190, EPI_ISL_660191, EPI_ISL_660192, EPI_ISL_660194, EPI_ISL_660198, EPI_ISL_660199, EPI_ISL_660202, EPI_ISL_660203, EPI_ISL_660204, EPI_ISL_660205, EPI_ISL_660206, EPI_ISL_660207, EPI_ISL_660209, EPI_ISL_660210, EPI_ISL_660212, EPI_ISL_660214, EPI_ISL_660215, EPI_ISL_660216, EPI_ISL_660219, EPI_ISL_660220, EPI_ISL_660224, EPI_ISL_660227, EPI_ISL_660234, EPI_ISL_660235, EPI_ISL_660236, EPI_ISL_660237, EPI_ISL_660238, EPI_ISL_660239, EPI_ISL_660240, EPI_ISL_660241, EPI_ISL_660242, EPI_ISL_660244, EPI_ISL_660245, EPI_ISL_660246, EPI_ISL_660248, EPI_ISL_660249, EPI_ISL_660250, EPI_ISL_660251, EPI_ISL_660252, EPI_ISL_660254, EPI_ISL_660255, EPI_ISL_660613, EPI_ISL_660618, EPI_ISL_660619, EPI_ISL_660621, EPI_ISL_660622, EPI_ISL_660623, EPI_ISL_660624, EPI_ISL_660626, EPI_ISL_660627, EPI_ISL_660628, EPI_ISL_660629, EPI_ISL_660630, EPI_ISL_660631, EPI_ISL_660632, EPI_ISL_660634, EPI_ISL_660635, EPI_ISL_660638, EPI_ISL_660639, EPI_ISL_660640, EPI_ISL_660641, EPI_ISL_660642, EPI_ISL_660643, EPI_ISL_660645, EPI_ISL_660650, EPI_ISL_660651, EPI_ISL_660654, EPI_ISL_660655, EPI_ISL_660662, EPI_ISL_660663, EPI_ISL_678598, EPI_ISL_678601, EPI_ISL_678602, EPI_ISL_678603, EPI_ISL_678605, EPI_ISL_678606, EPI_ISL_678607, EPI_ISL_678608, EPI_ISL_678609, EPI_ISL_678610, EPI_ISL_678612, EPI_ISL_678616, EPI_ISL_678618, EPI_ISL_678619, EPI_ISL_678622, EPI_ISL_678623, EPI_ISL_678624, EPI_ISL_678629, EPI_ISL_678640, EPI_ISL_736926, EPI_ISL_736932, EPI_ISL_736935, EPI_ISL_736940, EPI_ISL_736941, EPI_ISL_736944, EPI_ISL_736953, EPI_ISL_736954, EPI_ISL_736956, EPI_ISL_736971, EPI_ISL_736973, EPI_ISL_736974, EPI_ISL_736978, EPI_ISL_736980, EPI_ISL_736985, EPI_ISL_736988, EPI_ISL_736991, EPI_ISL_736992, EPI_ISL_736993, EPI_ISL_736994, EPI_ISL_825096, EPI_ISL_825097, EPI_ISL_825098, EPI_ISL_825100, EPI_ISL_825101, EPI_ISL_825103, EPI_ISL_825104, EPI_ISL_825106, EPI_ISL_825109, EPI_ISL_825110, EPI_ISL_825111, EPI_ISL_825113, EPI_ISL_825114, EPI_ISL_825117, EPI_ISL_825119, EPI_ISL_825120, EPI_ISL_825121, EPI_ISL_825125, EPI_ISL_825130, EPI_ISL_825131, EPI_ISL_825133, EPI_ISL_825446, EPI_ISL_825452			
see above	NHLS-IALCH	KRISP, KZN Research Innovation and Sequencing Platform	Cele S; Chimukangara B; ChimukangaraB; Gazy I; Giandhari J; Karim F; Khan S; Lessells R; Mdlalose K; Pillay S; Sigal A; Tegally H; Wilkinson E; York D; de Oliveira T
EPI_ISL_860582, EPI_ISL_860595	NHLS-IALCH	KRISP, KZn Research Innovation and Sequencing Platform	Giandhari J; Khan S; Lessells R; Mdlalose K; Pillay S; Tegally H; Wilkinson E; York D; de Oliveira T
EPI_ISL_418241, EPI_ISL_418242, EPI_ISL_420037, EPI_ISL_766861, EPI_ISL_766862, EPI_ISL_766863, EPI_ISL_766864, EPI_ISL_766865, EPI_ISL_766866, EPI_ISL_766867, EPI_ISL_766869, EPI_ISL_766871, EPI_ISL_766872, EPI_ISL_766873, EPI_ISL_766874, EPI_ISL_766875			

see above	NIC Viral Respiratory Unit - Institut Pasteur of Algeria	National Reference Center for Viruses of Respiratory Infections, Institut Pasteur, Paris	Angela Brisebarre; Etienne Simon-Lorière; Fawzi Derrar; Flora Donati; Marion Barbet; Maud Vanpeene; Mélanie Albert; Méline Bizard; Sylvie Behillil; Sylvie van der Werf; Vincent Enouf
EPI_ISL_1034177	NIV Influenza	NIV Influenza	Potdar V
EPI_ISL_1278136, EPI_ISL_1278160	NL-Dr. Leonard A. Miller Centre for Health Services	National Microbiology Laboratory (NML)	Adel Malek; Anna Majer; Anneliese Landgraff; CanCOGeN's metadata curation team; Darian Hole; Elsie Grudeski; Gary Van Domselaar; George Zahariadis; Grace Seo; Jennifer Tanner; Kerri Smith; Kirsten Biggar; Laura Gilbert; Madison Chapel; Morag Graham; Natalie Knox; Nathalie Bastien; Philip Mabon; Public Health Agency of Canada CanCOGeN team; Rhiannon Huzarewich; Robert Needle; Russell Mandes; Shari Tyson; Timothy Booth; Yan Li; Yang Yu
EPI_ISL_960304	NLZOH, Laboratory for Virology	NLZOH, Laboratory for Virology	Cesare Camma (IZSAM); Erik Alm (ECDC); Katarina Prosenc (Laboratory for Virology)
EPI_ISL_1655987	NMVRVI	National Public Health Surveillance Laboratory	Ana Steponkiene; Danas Baksa; Jelena Razmuk; Lukas Vasionis; Lukas Zemaitis; Migle Gabrielaite; Svajune Muralyte
EPI_ISL_903721	NV State Public Health Laboratory	Genomics and Discovery, Respiratory Viruses Branch, Division of Viral Diseases, Centers for Disease Control and Prevention	Anna Montmayeur; Anna Uehara; Ben L. Rambo-Martin; Clinton R. Paden; Dhwani Batra; Haibin Wang; Jasmine Padilla; Jing Zhang; Justin Lee; Krista Queen; Lori Rowe; Mark Burroughs; Mili Sheth; Peter W. Cook; Rachel Marine; Sarah Nobles; Suxiang Tong; Yan Li; Ying Tao
EPI_ISL_1657234	NVSPL	National Public Health Surveillance Laboratory	Ana Steponkiene; Danas Baksa; Jelena Razmuk; Lukas Vasionis; Lukas Zemaitis; Migle Gabrielaite; Svajune Muralyte
EPI_ISL_451427, EPI_ISL_1654612	NYU Langone Health	Departments of Pathology and Medicine, New York University School of Medicine	Adriana Heguy; Alireza Khodadadi-Jamayran; Amy Rapkiewicz; Andre M. Ribeiro-dos-Santos; Andrew Lytle; Antonio Serrano; Brendan Belovarac; Christian Marier; Dacia Dimartino; Emily Guzman; Emily Huang; Gael Westby; George Jour; Guiqing Wang; Guomiao Shen; Iman Osman; Jared Pinnell; John Cadley; John Chen; Lawrence H. Lin; Ludovic Boytard; Margaret Black; Maria Aguero-Rosenfeld; Marie Samanovic-Golden; Mark J. Mulligan; Matija Snuderl; Matthew T. Maurano; Megan Hogan; Nick Vulpescu; Paolo Cotzia; Paul Zappile; Peter Meyn; Raquel Ordonez Ciriza; Raven Luther; Sitharam Ramaswami; Tatyana Gindin; Theodore Vougiouklakis; Vanessa Raabe; Xiaojun Feng; Yutong Zhang
EPI_ISL_745188	Nababeep Hospital	National Health Laboratory Service (NHLS), Tygerberg	Bronwyn Kleinhans; Eduan Wilkindon; Gert van Zyl; Houriiyah Tegally; Kayla Delaney; Susan Engelbrecht; Tulio de Oliveira; Wolfgang Preiser
EPI_ISL_1233479	National Center for Infectious and Parasitic Diseases (NCIPD)	National Center for Infectious and Parasitic Diseases (NCIPD)	Alexiev et al
EPI_ISL_1231550	National Center for Infectious and Parasitic Diseases (NCIPD)	National Center for Infectious and Parasitic Diseases (NCIPD)	Alexiev et al
EPI_ISL_1139149, EPI_ISL_1139150, EPI_ISL_1139151, EPI_ISL_1139153	National Center of Disease Control and Prevention of the Republic of Armenia	Institute of Molecular Biology NAS RA, Republic of Armenia, Department of Bioengineering, BioinformaticsInstitute and Molecular Biology IBMPh RAU, Republic of Armenia	Andranik Chavushyan; Arsen Arakelyan; Diana Avetyan; Gayane Melik-Pashayan; Gisane Khachatyan; Hovsep Ghazaryan; Maria Nikoghosyan; Nelli Muradyan; Roksana Zakharyan; Shushan Sargrsyan; Siras Hakobyan; Tamara Sirunyan
EPI_ISL_454572	National Center of Expertise	National Center for Expertise, Kazakhstan National Center for Biotechnology, Kazakhstan	; Abdaliyev Askar; Akhmetollayev Ilyas; Amirgazin Asylulan; Aushakhmetova Zabira; Kalendar Ruslan; Lutsay Viktoriya; Rakhmetova Akbota; Ramankulov Yerlan; Shevtsov Alexandr
EPI_ISL_1302118, EPI_ISL_1302132, EPI_ISL_1302141, EPI_ISL_1302143	National Center of Infectious and Parasitic Diseases	National Center of Infectious and Parasitic Diseases	Alexiev et al
EPI_ISL_496525	National Centre For Cell Science	National Centre For Cell Science	Arvind Sahu; DBT's PAN-INDIA 1000 SARS-CoV2 RNA genome sequencing consortium; Dhiraj Paul; Girdhari Lal; Janesh Kumar; Kavita Bala Anand; Kunal Jani; Maharashtra COVID-19 Study Group; Manoj Kumar Bhat; Murlidhar Tambe; Radha Chauhan; Rajesh Karyakarte; Rajiv Mohan Gupta; Santosh Karade; Shelinder Pal Singh Shergill; Sourav Sen; Suvarna Joshi; Vasudevan Seshadri; Yogesh S Shouche
EPI_ISL_1384876, EPI_ISL_1384880	National Centre For Cell Science	National Centre For Cell Science – INSACOG	Ajay Pillai; Dhiraj Paul; INSACOG Consortium team; Manoj Kumar Bhat; Mitali Inamdar; Mohak P Gujare; Shivang P. Bhanushali; Sonal Manik Chavan; Yogesh Shouche.
EPI_ISL_407988	National Centre for Infectious Diseases	Programme in Emerging Infectious Diseases, Duke-NUS Medical School	Barnaby E Young; Danielle E Anderson; David CB Lye; Gavin JD Smith; Jayanthi Jayakumar; Linfa Wang; Martin Linster; Yan Zhuang; Yee Sin Leo; Yvonne CF Su
EPI_ISL_1407107, EPI_ISL_1407137	National HIV Reference Laboratory, Ministry of Health, Public Health Institute of Malawi	KRISP, KZN Research Innovation and Sequencing Platform	Auld A; Chilima B; Chiwaula M; Emmanuel SJ; Giandhari J; Kaba M; Kampira E; Kasambara W; Kim L; Lessells R; Maida A; Mvula B; Mwangomba W; Naidoo Y; Panja L; Pillay S; Tegally H; Wadonda N; Wilkinson E; de Oliveira T
EPI_ISL_464112, EPI_ISL_464113, EPI_ISL_464118, EPI_ISL_464121, EPI_ISL_464123, EPI_ISL_464129, EPI_ISL_464133, EPI_ISL_464138, EPI_ISL_464155			
see above	National Health Laboratory Service (NHLS), Tygerberg	Division of Medical Virology, Stellenbosch University and National Health Laboratory Service (NHLS)	Bronwyn Kleinhans; Eduan Wilkindon; Gert van Zyl; Houriiyah Tegally; Kayla Delaney; Susan Engelbrecht; Tulio de Oliveira; Wolfgang Preiser
EPI_ISL_634981, EPI_ISL_634983, EPI_ISL_634984, EPI_ISL_634985, EPI_ISL_634986, EPI_ISL_634987, EPI_ISL_634989, EPI_ISL_634994, EPI_ISL_634996, EPI_ISL_634997, EPI_ISL_634998, EPI_ISL_634999, EPI_ISL_635000, EPI_ISL_635001, EPI_ISL_635002, EPI_ISL_635004, EPI_ISL_635005, EPI_ISL_635006, EPI_ISL_635007, EPI_ISL_635008, EPI_ISL_635009, EPI_ISL_635010, EPI_ISL_635011, EPI_ISL_635014, EPI_ISL_635015, EPI_ISL_635016, EPI_ISL_635017, EPI_ISL_635018, EPI_ISL_635021, EPI_ISL_635022, EPI_ISL_635024, EPI_ISL_635025, EPI_ISL_635030, EPI_ISL_635031, EPI_ISL_635032, EPI_ISL_635034, EPI_ISL_635035, EPI_ISL_635045, EPI_ISL_635047, EPI_ISL_635048, EPI_ISL_635049, EPI_ISL_635050, EPI_ISL_635052, EPI_ISL_635054, EPI_ISL_635055, EPI_ISL_635056, EPI_ISL_635058			
see above	National Health Laboratory Service - Inkosi Albert Luthuli Central Hospital (NHLS-IALCH)	KRISP, KZN Research Innovation and Sequencing Platform	Giandhari J; Khan S; Lessells R; Mdlalose K; Pillay S; Tegally H; Wilkinson E; York D; de Oliveira T
EPI_ISL_1250430, EPI_ISL_1250436, EPI_ISL_1250442, EPI_ISL_1366782, EPI_ISL_1366785, EPI_ISL_1366787, EPI_ISL_1366795			
see above	National Health Laboratory Service, South Africa	KRISP, KZN Research Innovation and Sequencing Platform	Emmanuel SJ; Giandhari J; Khan S; Laguda-Akingba O; Lessells R; Maslo C; Mdlalose K; Pillay S; Sitharam L; Tegally H; Wilkinson E; York D; de Oliveira T
EPI_ISL_1048527	National Health Laboratory Service, South Africa	KRISP, KZn Research Innovation and Sequencing Platform	Emmanuel SJ; Giandhari J; Khan S; Lessells R; Mdlalose K; Pillay S; Tegally H; Wilkinson E; York D; de Oliveira T
EPI_ISL_421662	National Influenza Center, Indian Council of Medical Research - National Institute of Virology	Indian Council of Medical Research-National Institute of Virology, Microbial Containment Complex	Amita Jain; Anita Shete-Aich; Atanu Basu; Bharti Malhotra; Deepika Chaudhary; Dimpal A. Nyayanit; Gajanan Sapkal; Gururaj Deshpande; Hitesh Dighe; Manohar. L. Chaudhary; Padinjaremattathil Thankappan Ullas; Pragya D. Yadav; Priya Abraham; Sarah Cherian; Savita Patil; Sreelekshmy Mohandas; Triparna Majumdar; Varsha Potdar
EPI_ISL_416429	National Influenza Center, National Institute of Hygiene and Epidemiology (NIHE)	National Influenza Center, National Institute of Hygiene and Epidemiology (NIHE)	Dang Duc Anh; Futoshi Hasebe; Hoang Vu Mai Phuong; Kouichi Morita; Le Quynh Mai; Le Thi Thanh; Meng Ling Moi; Nguyen Le Khanh Hang; Nguyen Phuong Anh; Nguyen Vu Son; Pham Hong Quynh Anh; Pham Thi Hien; Taichiro Takemura; Takeshi Nabeshima; Tran Thu Huong; Ung Thi Hong Trang; Vuong Duc Cuong
EPI_ISL_862077, EPI_ISL_862080, EPI_ISL_1014685	National Influenza Center, Virology Department	National Influenza Center	A Nejati; F Ajaminejad; F Ajaminejad and T Mokhtari Azad; J Yavarian; K Sadeghi; N Ghavvami; N Ghavvami and T Mokhtari Azad; NZ Shafiei Jandaghi; V Salimi
EPI_ISL_2285862	National Influenza Centre	National Influenza Centre	; Benjamiin B. Lindsey; Benjamin H. Foulkes; Dennis Laryea; Ernest Asiedu; Franklin Asiedu-Bekoe; Gordon Awandare; Ivy A. Asante; Joseph Oliver-Commey; Joyce Ngoi; Linda Boatemaa; Lorreta Kwasah; Mathew D. Parker; Michael Marks; Mildred Adusei-Poku; Sharon Hsu; Thushan I de Silva; William K. Ampofo
EPI_ISL_417186, EPI_ISL_514382, EPI_ISL_515166, EPI_ISL_515172, EPI_ISL_622934, EPI_ISL_622937, EPI_ISL_622941			
see above	National Institute for Communicable Diseases of the National Health Laboratory Service	National Institute for Communicable Diseases of the National Health Laboratory Service	A; Allam M; Bhiman JN; Ismail A; Khumalo Z; Kwenda S; Mnyameni F; Mohale T; Mtshali P; Subramoney K; van Heusden P; von Gottberg
EPI_ISL_469254	National Institute for Viral Disease Control and Prevention, China CDC	Institute of Viral Disease Control and Prevention, China CDC	Chun Huang; Dayan Wang; George Fu Gao; Guizhen Wu; Li Zhao; Lijuan Chen; Peihua Niu , Baoying Huang; Roujian Lu; Wenbo Xu; Wenjie Tan; Wenling Wang; Yubai Bi
EPI_ISL_591279, EPI_ISL_850947, EPI_ISL_850949	National Institute for Viral Disease Control and Prevention, China CDC	National Institute for Viral Disease Control and Prevention, China CDC	Cao Chen; Dayan Wang; George F.Gao; Hong Wang; Huilai Ma; Ji Wang; Jingdong Song; Jun Han; Kai Nie; Ruqin Gao; Shiwen Wang; Weimin Zhou; Wenbo Xu; Wenjie Tan; Xiang Zhao; Yang Song; Yanhai Wang; Yao Meng; Yenan Feng; Yong Zhang; Yuchao Wu; Zhaoguo Wang; Zhixiao Chen
EPI_ISL_1391017	National Institute of Health Research and Development	National Institute of Health Research and Development	Agustiningsih; Arie Ardiansyah Nugraha; Fauzul Muna; Hana Apsari Pawestri; Hartanti Dian Ikawati; Herna; Holy Arif Wibowo; Irene Lorinda Indalao; Kartika Dewi Puspa; Kindi Adam; Krisna Nur Andriana Pangesti; Natalie Laurencia Kipuw; Nelly Puspandari; Ni Ketut Susilarini; Nike Susanti; Nurika Hariastuti; Reni Herman; Ririn Ramadhany; Subangkit; Tati Febriyanti; Triyani Soekarso; Ully Alfi Nikmah; Vivi Setiawaty.; Yuni Rukminiati
EPI_ISL_979799, EPI_ISL_1116455, EPI_ISL_1258447, EPI_ISL_1258457, EPI_ISL_1418329	National Institute of Infectious Diseases-Prof. Dr. Matei Bals Molecular Diagnostics Laboratory	National Institute of Infectious Diseases-Prof. Dr. Matei Bals Molecular Diagnostics Laboratory	Andreea Tudor; Corina Casangiu; Dan Otelea; Ionelia Nicolae; Leontina Banica; Marius Surleac; Ovidiu Vlaicu; Petre Milu; Simona Paraschiv
EPI_ISL_483636, EPI_ISL_1035809, EPI_ISL_1040485, EPI_ISL_1508999, EPI_ISL_1524771	National Institute of Laboratory Medicine and Referral Center	Genomic Research Lab, BCSIR	A. K. M. Shamsuzzaman; A. K. M.Shamsuzzaman; Abu Sayeed Mohammad Mahmud; Arifa Akram; Asish Kumar Ghosh; Barna Goswami; Eshrar Osman; Iffat Jahan; Mahmuda Yeasmin; Md. Ahasan Habib; Md. Ahashan Habib; Md. Maruf Ahmed Molla; Md. Murshed Hasan Sarkar; Md. Saddam Hossain; Md. Salim Khan; Mohammad Samir Uzzaman; Salek Ahmed Sajib; Shahina Akter; Sheikh Md. Selim Al Din; Tanjina Akhtar Banu; Tanjina Akhter Banu; Tanjina Akhter Banu; Tasnim Nafisa; Utpal Chandra Ray
EPI_ISL_1492330, EPI_ISL_1492332, EPI_ISL_1492389, EPI_ISL_1588439	National Institute of Public Health	National Institute of Public Health	Alexander Nagy; Dusan Trnka; Helena Jirincova; Jaromira Vecerova; Timotej Suri
EPI_ISL_1321358	National Institute of Public Health	National Reference Laboratory for Influenza and Respiratory Viruses CZE	Alexander Nagy; Dusan Trnka; Helena Jirincova; Jaromira Vecerova; Timotej Suri
EPI_ISL_1510304, EPI_ISL_1510388	National Institute of Public Health	State Veterinary Institute Prague	A; D; H; J; Jirincova; Nagy; Suri; T; Trnka; Vecerova
EPI_ISL_434558	National Institutes of Health, University of the Philippines Manila	Philippine Genome Center	Alessandra C. Sanchez; Benedict A. Maralit; Bernard Demot; Carlo M. Lapid; Christina Tan; El King D. Morado; Eva Maria Cutiongco-de la Paz; Francis A. Tablizo; Haifa L.Gaza; Jan Michael C. Yap; Jarvin E. Nipales; Jo-Hannah S. Llames; John Mark Velasco; Joshua Gregor A. Dizon; Joy Ann Petronio-Santos; Julius Aaron Mejia; Kris P. Punayan; Kristianne Arielle D. Gabriel; Maribell Dollete; Marissa M. Alejandria; Raul V. Destura; Shana F. Genavia; Shebna Rose D. Fabilloren; Shiela Mae M. Araiza; Sonia Salamat; and Cynthia P. Saloma
EPI_ISL_1266589	National Laboratory for Health, Environment and Food, OMM, Celje	CISLD (Clinical Institute of Special Laboratory Diagnostics), University Children's Hospital, University Medical Center Ljubljana	Ana Grom; Barbara Jenko Bizjan; Jernej Kovač; Katarina Kozmos; Marko Pokorn; Maruša Debeljak; Robert Šket; Tadej Battelino; Tine Tesovnik
EPI_ISL_1266502	National Laboratory for Health, Environment and Food, OMM, Koper	CISLD (Clinical Institute of Special Laboratory Diagnostics), University Children's Hospital, University Medical Center Ljubljana	Ana Grom; Barbara Jenko Bizjan; Jernej Kovač; Katarina Kozmos; Marko Pokorn; Maruša Debeljak; Robert Šket; Tadej Battelino; Tine Tesovnik
EPI_ISL_1111821	National Laboratory for Health, Environment and Food, OMM, Maribor	CISLD (Clinical Institute of Special Laboratory Diagnostics), University Children's Hospital, University Medical Center Ljubljana	Ana Grom; Barbara Jenko Bizjan; Jernej Kovač; Katarina Kozmos; Marko Pokorn; Maruša Debeljak; Robert Šket; Tadej Battelino; Tine Tesovnik
EPI_ISL_512636	National Laboratory for Influenza/Virology reference laboratory, Public Health Center of the Ministry of	Respiratory Virus Unit, Microbiology Services Colindale, Public Health	Dr. Iryna Demchyshyna; PHE Covid Sequencing Team

	Health of Ukraine	England	
EPI_ISL_1392640	National Medical Research Center for Obstetrics, Gynecology and Perinatology named after Academician V.I.Kulakov of the Ministry of Healthcare of Russian Federation	National Medical Research Center for Obstetrics, Gynecology and Perinatology named after Academician V.I.Kulakov of the Ministry of Healthcare of Russian Federation	Andrey Donnikov; Andrey Goltsov; Denis Rebrikov; Dmitry Abramov; Dmitry Korostin; Dmitry Trofimov; Gennady Sukhikh; Jekaterina Shubina; Tatiana Priputnevich; Yegor Botsmanov
EPI_ISL_644795, EPI_ISL_644798, EPI_ISL_644803, EPI_ISL_644804, EPI_ISL_644805, EPI_ISL_644806, EPI_ISL_644807, EPI_ISL_644808, EPI_ISL_644811, EPI_ISL_644812, EPI_ISL_644816, EPI_ISL_644818, EPI_ISL_1191782, EPI_ISL_1191783, EPI_ISL_1191784, EPI_ISL_1191786, EPI_ISL_1191787, EPI_ISL_1191788, EPI_ISL_1191789, EPI_ISL_1191790, EPI_ISL_1191791, EPI_ISL_1191792, EPI_ISL_1191793, EPI_ISL_1191794, EPI_ISL_1191795, EPI_ISL_1191799, EPI_ISL_1191801, EPI_ISL_1191802, EPI_ISL_1191803, EPI_ISL_1191804, EPI_ISL_1191805, EPI_ISL_1191806, EPI_ISL_1191807, EPI_ISL_1191809, EPI_ISL_1191812, EPI_ISL_1191815, EPI_ISL_1191818, EPI_ISL_1191822, EPI_ISL_1191824, EPI_ISL_1191828, EPI_ISL_1191831, EPI_ISL_1191832, EPI_ISL_1191833, EPI_ISL_1191843, EPI_ISL_1191866, EPI_ISL_1191875, EPI_ISL_1191876, EPI_ISL_1191883, EPI_ISL_1191888, EPI_ISL_1191909, EPI_ISL_1191927, EPI_ISL_1191936, EPI_ISL_1191937, EPI_ISL_1191939, EPI_ISL_1191947, EPI_ISL_1191950, EPI_ISL_1191959, EPI_ISL_1191965, EPI_ISL_1192019, EPI_ISL_1192023, EPI_ISL_1192030, EPI_ISL_1192040			
see above	National Microbiology Reference Laboratory	Quadram Institute Bioscience	Agnes Juru; Alexander Goredema; Ana-Victoria Gutierrez; Andrew J. Page; Andrew Tarupiwa; Barbra Murwira; Beuty Makamure; Charles Nyagupe; David Baker; Faustinos T Takawira; Gaetan Thilliez; Gemma Kay; Gibson Mhlanga; Hlanai Gumbo; Isaac Phiri; Justin O'Grady; Kenneth K Maeka; Leonardo de Oliveira Martins; Muchaneta Mugabe; Portia Manangazira; Raiva Simbi; Robert Kingsley; Sekesai Zinyowera; Tapfumanei Mashe; Tatenda Takawira; Thanh Le Viet
EPI_ISL_1195200, EPI_ISL_1447299, EPI_ISL_1447316	National Public Health Center, COVID Laboratory	National Public Health Center, National Biosafety Laboratory	Bernadett Pályi; Dániel Déri; Judit Henczkó; Norbert Solymosi; Nóra Magyar; Zoltán Kis
EPI_ISL_457833, EPI_ISL_457843	National Public Health Laboratory	KEMRI-Wellcome Trust Research Programme/KEMRI-CGMR-C Kilifi	Githinji G. et al 2020
EPI_ISL_845548, EPI_ISL_845549, EPI_ISL_845550, EPI_ISL_845552, EPI_ISL_845553, EPI_ISL_845558, EPI_ISL_845562			
see above	National Public Health Laboratory, Cameroon	African Centre of Excellence for Genomics of Infectious Diseases (ACEGID), Redeemer's University	Oluniyi P.E. et al
EPI_ISL_428849, EPI_ISL_462358, EPI_ISL_462412, EPI_ISL_483610, EPI_ISL_498567, EPI_ISL_596460, EPI_ISL_626640, EPI_ISL_645128, EPI_ISL_693315, EPI_ISL_693321, EPI_ISL_728185, EPI_ISL_803987, EPI_ISL_803988, EPI_ISL_825069, EPI_ISL_937519, EPI_ISL_981008, EPI_ISL_1081922, EPI_ISL_1442955, EPI_ISL_1476999, EPI_ISL_1519381, EPI_ISL_1524785, EPI_ISL_1543949, EPI_ISL_1543956			
see above	National Public Health Laboratory, National Centre for Infectious Diseases	National Public Health Laboratory, National Centre for Infectious Diseases	Chavatte JM; Cui L; Grace Jie Yin Ngan; Lin Cui; Lin RTP; Mak TM; Octavia S; Raymond Tzer Pin Lin; Royce Ang; Sophie Octavia; Tze Minn Mak; Zhenyang Zhou; Zhou Z
EPI_ISL_1122419, EPI_ISL_1122420	National Public Health Laboratory, National Centre for Infectious Diseases	National Virology Reference Laboratory	Lin Cui; Raymond Tzer Pin Lin; Surita Taib; Tze Minn Mak; Zainun Zaini; Zhenyang Zhou
EPI_ISL_1497324, EPI_ISL_1497443, EPI_ISL_1497451, EPI_ISL_1497499, EPI_ISL_1497500	National Public Health Organization	National Public Health Organization	Kyriaki Tryfinopoulou et al
EPI_ISL_480224	National Reference Laboratory "Influenza and acute respiratory diseases"	NRL-HIV	Ivailo Alexiev; Ivan Ivanov; Ivva Philipova
EPI_ISL_962877	National Virology Reference Laboratory	National Public Health Laboratory, National Centre for Infectious Diseases	Lin Cui; Raymond Tzer Pin Lin; Taib Surita; Tze Minn Mak; Zaini Zainun; Zhenyang Zhou
EPI_ISL_848159	National Virus Reference Laboratory	Irish Coronavirus Sequencing Consortium - National University of Ireland Galway	Grainne Mc Andrew; Kate Reddington; Simone Coughlan
EPI_ISL_767744	National Virus Reference Laboratory	Irish Coronavirus Sequencing Consortium - Teagasc Moorepark	Calum Walsh; Genuity Ireland
EPI_ISL_732474, EPI_ISL_837399, EPI_ISL_909859, EPI_ISL_1055137, EPI_ISL_1092522, EPI_ISL_1092925, EPI_ISL_1358154, EPI_ISL_1396997, EPI_ISL_1620380, EPI_ISL_1657521			
see above	National Virus Reference Laboratory	National Virus Reference Laboratory	Charlene Bennet; Charlene Bennett; Cillian F De Gascun; Daniel Hare; Gabriel Gonzalez; Guerrino Macori; Jonathan Dean; Michael Carr; Seamus Fanning; Zoe Yandle
EPI_ISL_1121113	Nebraska Public Health Laboratory	NPHL COVID-19 Response Team	NPHL COVID-19 Response Team
EPI_ISL_678597	Netcare	KRISP, KZN Research Innovation and Sequencing Platform	ChimukangaraB; Giandhari J; Khan S; Lessells R; Mdlalose K; Pillay S; Tegally H; Wilkinson E; York D; de Oliveira T
EPI_ISL_1136786, EPI_ISL_1136897, EPI_ISL_1220869	Nevada State Public Health Laboratory	Nevada State Public Health Laboratory	Andrew Gorzalski; Mark Pandori
EPI_ISL_696468	New Horizon Clinic wc NZC & NHLS/UCT	KRISP, KZN Research Innovation and Sequencing Platform	Arash Iranzadeh; Bruna Galvao; Carolyn Williamson; Deelan Doolabh; Diana Hardie; Emanuel James San; Houriiyah Tegally; Innocent Mudau; Jennifer Giandhari; Kruger Marais; Lynn Tyers; Marvin Hsiao; Stephen Korsman; Sureshnee Pillay; Tulio de Oliveira
EPI_ISL_1197964	New Mexico Department of Health Scientific Laboratory	Center for Global Health, University of New Mexico Health Sciences Center	Anastacia Griego; Darrell Dinwiddie; Daryl Domman; Joseph Hicks; Kurt Schwalm; Michael Edwards; Twila Kunde
EPI_ISL_1038928, EPI_ISL_1114672	New Mexico Department of Health Scientific Laboratory	New Mexico Department of Health Scientific Laboratory	Anastacia Griego-Fisher; D'eldra Malone; Ellie Johnson; Jennifer Benoit
EPI_ISL_1010699, EPI_ISL_1121989, EPI_ISL_1315071	New South Wales Health Pathology Royal Prince Alfred Hospital	Microbiology RPAH	Au, J.; Bull, R.; Deveson, I.; Foster, C.; Rawlinson, W.; Ruiz Silva, M.; Van Hal, S.
EPI_ISL_455422	Nigeria Centre for Disease Control	African Centre of Excellence for Genomics of Infectious Diseases (ACEGID), Redeemer's University, Ede, Osun State, Nigeria	Ajogbasile F.V.; Folarin O.A.; Happi C.T.; Ihekweazu C.; Kayode A.; Oguzie J.; Olawoye I.; Olumade T.; Oluniyi P.E.; Uwanibe J.
EPI_ISL_1173208, EPI_ISL_1173221, EPI_ISL_1173227, EPI_ISL_1173230, EPI_ISL_1173244	Nigeria Centre for Disease Control (NCDC)	African Centre for Excellence for Genomics of Infectious Diseases (ACEGID), Redeemer's University	I.B.; Olawoye; et al
EPI_ISL_872601, EPI_ISL_872602, EPI_ISL_872603, EPI_ISL_872604, EPI_ISL_872607, EPI_ISL_872611, EPI_ISL_872613, EPI_ISL_872615, EPI_ISL_872616, EPI_ISL_872618, EPI_ISL_872622, EPI_ISL_872623, EPI_ISL_872625, EPI_ISL_906281, EPI_ISL_906283, EPI_ISL_906285, EPI_ISL_906286, EPI_ISL_906287, EPI_ISL_906291, EPI_ISL_906299, EPI_ISL_906301, EPI_ISL_906302, EPI_ISL_906305, EPI_ISL_941276, EPI_ISL_941277, EPI_ISL_941278, EPI_ISL_941284, EPI_ISL_941285, EPI_ISL_941286, EPI_ISL_941292, EPI_ISL_941293, EPI_ISL_941926, EPI_ISL_1242010, EPI_ISL_1242013, EPI_ISL_1242025, EPI_ISL_1242026, EPI_ISL_1242028			
see above	Nigeria Centre for Disease Control (NCDC)	African Centre of Excellence for Genomics of Infectious Diseases (ACEGID), Redeemer's University	Oluniyi P.E. et al
EPI_ISL_455362, EPI_ISL_455412, EPI_ISL_455424, EPI_ISL_455431, EPI_ISL_487091, EPI_ISL_487099, EPI_ISL_487101, EPI_ISL_487102, EPI_ISL_487105, EPI_ISL_487107, EPI_ISL_487109, EPI_ISL_487110, EPI_ISL_487111, EPI_ISL_487112, EPI_ISL_527873, EPI_ISL_527874, EPI_ISL_527878, EPI_ISL_527879, EPI_ISL_527881, EPI_ISL_527882, EPI_ISL_527884, EPI_ISL_527887, EPI_ISL_527888, EPI_ISL_527889, EPI_ISL_527890, EPI_ISL_527892, EPI_ISL_527898, EPI_ISL_527901, EPI_ISL_527904, EPI_ISL_527911, EPI_ISL_527912, EPI_ISL_527914, EPI_ISL_527915, EPI_ISL_729920, EPI_ISL_729923, EPI_ISL_729924, EPI_ISL_729925, EPI_ISL_729926, EPI_ISL_729928, EPI_ISL_729929, EPI_ISL_729930, EPI_ISL_729931, EPI_ISL_729932, EPI_ISL_729935, EPI_ISL_729937, EPI_ISL_729938, EPI_ISL_729939, EPI_ISL_729941, EPI_ISL_729943, EPI_ISL_729946, EPI_ISL_729947, EPI_ISL_729948, EPI_ISL_729950, EPI_ISL_729952, EPI_ISL_729953, EPI_ISL_729954, EPI_ISL_729955, EPI_ISL_729956, EPI_ISL_729957, EPI_ISL_729958, EPI_ISL_729960, EPI_ISL_729961, EPI_ISL_729962, EPI_ISL_729964, EPI_ISL_729966, EPI_ISL_729968, EPI_ISL_729969, EPI_ISL_729970, EPI_ISL_729972, EPI_ISL_729973, EPI_ISL_729974, EPI_ISL_729975, EPI_ISL_729976, EPI_ISL_729977, EPI_ISL_729978, EPI_ISL_729979, EPI_ISL_729982, EPI_ISL_729985, EPI_ISL_729986, EPI_ISL_729992, EPI_ISL_729993, EPI_ISL_729994, EPI_ISL_729995, EPI_ISL_729999, EPI_ISL_730004, EPI_ISL_730007, EPI_ISL_730008, EPI_ISL_730010, EPI_ISL_730012, EPI_ISL_730013, EPI_ISL_730015, EPI_ISL_730019, EPI_ISL_730020, EPI_ISL_730021, EPI_ISL_730024, EPI_ISL_730028, EPI_ISL_730029, EPI_ISL_730032, EPI_ISL_730033, EPI_ISL_730035, EPI_ISL_730036, EPI_ISL_730038, EPI_ISL_730039, EPI_ISL_730042			
see above	Nigeria Centre for Disease Control (NCDC)	African Centre of Excellence for Genomics of Infectious Diseases (ACEGID), Redeemer's University, Ede, Osun State, Nigeria	Ajogbasile F.V.; Folarin O.A.; Happi C.T.; Ihekweazu C.; Kayode A.; Oguzie J.; Olawoye I.; Olumade T.; Oluniyi P.E.; Oluniyi P.E. et al; Uwanibe J.
EPI_ISL_487113	Nigeria Centre for Disease Control (NCDC)	Redeemer's University, ACEGID	Ajogbasile F.V.; Folarin O.A.; Happi C.T.; Ihekweazu C.; Kayode A.; Oguzie J.; Olawoye I.; Olumade T.; Oluniyi P.E.; Uwanibe J.
EPI_ISL_977549, EPI_ISL_977551, EPI_ISL_977553, EPI_ISL_977554, EPI_ISL_977555, EPI_ISL_977556, EPI_ISL_977557, EPI_ISL_977558, EPI_ISL_977559, EPI_ISL_977565			
see above	Nigeria Centre of Disease Control (NCDC)	African Centre of Excellence for Genomics of Infectious Diseases (ACEGID), Redeemer's University	Olawoye I. B. et al
EPI_ISL_1093435, EPI_ISL_1093437, EPI_ISL_1093440, EPI_ISL_1093441, EPI_ISL_1093443, EPI_ISL_1093444, EPI_ISL_1093445, EPI_ISL_1093447, EPI_ISL_1093448, EPI_ISL_1093475, EPI_ISL_1235652, EPI_ISL_1235657, EPI_ISL_1235658, EPI_ISL_1235659, EPI_ISL_1235660, EPI_ISL_1235661, EPI_ISL_1235662, EPI_ISL_1235663			
see above	Nigerian Centre for Disease Control (NCDC)	African Centre for Excellence for Genomics of Infectious Diseases (ACEGID), Redeemer's University	I.B.; Olawoye; et al; et.al
EPI_ISL_1035812, EPI_ISL_1035813, EPI_ISL_1035815, EPI_ISL_1035818, EPI_ISL_1035820, EPI_ISL_1035824, EPI_ISL_1035826, EPI_ISL_1035827			
see above	Nigerian Centre for Disease Control (NCDC)	African Centre for Excellence for Genomics of Infectious Diseases (ACEGID), Redeemer's University, Ede	I.B.; Olawoye; et al
EPI_ISL_745142	Nomangesi Jayiya Clinic	National Health Laboratory Service (NHLS), Tygerberg	Bronwyn Kleinhans; Eduan Wilkindon; Gert van Zyl; Houriiyah Tegally; Kayla Delaney; Susan Engelbrecht; Tulio de Oliveira; Wolfgang Preiser
EPI_ISL_456396	North Shore Hospital	Institute of Environmental Science and Research (ESR)	Anja Werno; Antje van der Linden; Arlo Upton; Chris Mansell; David Hammer; Dragana Drinkovic; Erasmus Smit; Gary McAuliffe; Hana Sofia Andersson; James Ussher; Jill Sherwood; Joep de Ligt; Josh Freeman; Julia Howard; Juliet Elvy; Lauren Jelly; Mary DeAlmeida; Matt Blakiston; Matt Storey; Matthew Rogers; Max Bloomfield; Michael Addidle; Michelle Balm; Sally Roberts; Sarah Jefferies; Sharmini Muttaiyah; Susan Morpeth; Susan Taylor; Timothy Blackmore; Vani Sathyendran; Veronica Playle; Virginia Hope; Xiaoyun Ren
EPI_ISL_936571	Northwestern Memorial Hospital	Ozer Lab	Chad J. Achenbach; Egon A. Ozer; Judd F. Hultquist; Lacy M. Simons; Lawrence J. Jennings; Michael G. Ison; Ramon Lorenzo-Redondo
EPI_ISL_1118541, EPI_ISL_1317721	Norwegian Institute of Public Health, Department of Virology	Norwegian Institute of Public Health, Department of Virology	Atiya R Ali; Debech Nadia; Engebretsen Serina Beate; Garcia Llorente Ignacio; Hilde Elshaug; Hilde Vollan; Jon Bråte; Kamilla Heddeland Instefjord; Karoline Bragstad; Kathrine Stene-Johansen; Marie Paulsen Madsen; Olav Hungnes; Pedersen Benedikte Nevjen; Rasmus Riis Kopperud
EPI_ISL_735438	Nuclei Acid Testing - Rwanda National Reference Laboratory	GIGA Medical Genomics	Bouchra Boujemla; Esperence Umumararungu; Jacob Souopgui; Keith Durkin; Léon Mutesa; Maria Artesi; Marie-Pierre Hayette; Patrick Tuyisenge; Robert Rutayisire; Sabin Nsanzimana; Swaibu Gatare; Sébastien Bontems; Vincent Bours; Yvan Butera
EPI_ISL_735436, EPI_ISL_735437, EPI_ISL_735444, EPI_ISL_735446,	Nucleic Acid Testing - Rwanda National Reference Laboratory	GIGA Medical Genomics	Bouchra Boujemla; Esperence Umumararungu; Jacob Souopgui; Keith Durkin; Léon Mutesa; Maria Artesi; Marie-Pierre Hayette; Patrick Tuyisenge; Robert Rutayisire; Sabin Nsanzimana; Swaibu Gatare; Sébastien Bontems; Vincent Bours; Yvan Butera

EPI_ISL_735447, EPI_ISL_735448			
EPI_ISL_925847, EPI_ISL_925849, EPI_ISL_925850, EPI_ISL_925851, EPI_ISL_925852, EPI_ISL_925853, EPI_ISL_925854, EPI_ISL_925856, EPI_ISL_925857, EPI_ISL_925858, EPI_ISL_925859, EPI_ISL_925861, EPI_ISL_925862, EPI_ISL_925863, EPI_ISL_925864, EPI_ISL_925865, EPI_ISL_925866, EPI_ISL_925867, EPI_ISL_925870, EPI_ISL_925872, EPI_ISL_925873, EPI_ISL_925874, EPI_ISL_925876, EPI_ISL_925881, EPI_ISL_925882, EPI_ISL_925883, EPI_ISL_925884, EPI_ISL_925885, EPI_ISL_925886, EPI_ISL_925887, EPI_ISL_925888, EPI_ISL_925890, EPI_ISL_925891, EPI_ISL_925893, EPI_ISL_925895, EPI_ISL_925896, EPI_ISL_925897, EPI_ISL_925898, EPI_ISL_925899, EPI_ISL_925908, EPI_ISL_925909, EPI_ISL_925910, EPI_ISL_925912, EPI_ISL_925914, EPI_ISL_925915, EPI_ISL_930567, EPI_ISL_930634, EPI_ISL_930853, EPI_ISL_960227, EPI_ISL_960228, EPI_ISL_960229, EPI_ISL_960230, EPI_ISL_960231, EPI_ISL_960232, EPI_ISL_960233, EPI_ISL_960234, EPI_ISL_960235, EPI_ISL_960236, EPI_ISL_960237, EPI_ISL_960239, EPI_ISL_960240, EPI_ISL_960241, EPI_ISL_960242, EPI_ISL_960243, EPI_ISL_960244, EPI_ISL_960245, EPI_ISL_960246, EPI_ISL_960247, EPI_ISL_960248, EPI_ISL_960249, EPI_ISL_960250, EPI_ISL_960251, EPI_ISL_960253, EPI_ISL_960254, EPI_ISL_960255, EPI_ISL_960256, EPI_ISL_960257, EPI_ISL_960258, EPI_ISL_960259, EPI_ISL_960260, EPI_ISL_960261, EPI_ISL_960262, EPI_ISL_960263, EPI_ISL_960264, EPI_ISL_960265, EPI_ISL_960266, EPI_ISL_960268, EPI_ISL_960269, EPI_ISL_960270, EPI_ISL_960271, EPI_ISL_960272, EPI_ISL_960274, EPI_ISL_960275, EPI_ISL_960276, EPI_ISL_960277, EPI_ISL_960278, EPI_ISL_960279, EPI_ISL_960280, EPI_ISL_960281, EPI_ISL_960282, EPI_ISL_960283, EPI_ISL_960284, EPI_ISL_960285, EPI_ISL_960286, EPI_ISL_960287, EPI_ISL_960288, EPI_ISL_960289, EPI_ISL_960290, EPI_ISL_960291, EPI_ISL_960292, EPI_ISL_960293, EPI_ISL_960294, EPI_ISL_960295, EPI_ISL_960296, EPI_ISL_960297, EPI_ISL_960298, EPI_ISL_960299, EPI_ISL_960300, EPI_ISL_960301, EPI_ISL_960302, EPI_ISL_1300662, EPI_ISL_1301743, EPI_ISL_1301744, EPI_ISL_1301745, EPI_ISL_1301746, EPI_ISL_1301749, EPI_ISL_1301755, EPI_ISL_1301756, EPI_ISL_1301757, EPI_ISL_1301758, EPI_ISL_1301759, EPI_ISL_1301760, EPI_ISL_1301761, EPI_ISL_1301762, EPI_ISL_1301763, EPI_ISL_1301764, EPI_ISL_1301765, EPI_ISL_1301767, EPI_ISL_1301768, EPI_ISL_1301769, EPI_ISL_1301771, EPI_ISL_1301772, EPI_ISL_1301773, EPI_ISL_1301774, EPI_ISL_1301775, EPI_ISL_1301777, EPI_ISL_1301778, EPI_ISL_1301779, EPI_ISL_1301780, EPI_ISL_1301781, EPI_ISL_1301782, EPI_ISL_1301783, EPI_ISL_1301784, EPI_ISL_1301785, EPI_ISL_1301786, EPI_ISL_1301787, EPI_ISL_1301788, EPI_ISL_1301789, EPI_ISL_1301790, EPI_ISL_1301792, EPI_ISL_1301794, EPI_ISL_1301795, EPI_ISL_1301796, EPI_ISL_1301797, EPI_ISL_1301803, EPI_ISL_1302666, EPI_ISL_1302667, EPI_ISL_1302675, EPI_ISL_1302676, EPI_ISL_1302677, EPI_ISL_1302678, EPI_ISL_1302679, EPI_ISL_1302680, EPI_ISL_1302681			
see above	Nucleic Acid Testing, National Reference Laboratory	GIGA Medical Genomics	Bouchra Boujemla; Corinne Fasquelle; Esperence Umumararungu; Jacob Souopgui; Keith Durkin; Léon Mutesa; Maria Artesi; Marie-Pierre Hayette; Nathalie Renotte; Patrick Tuyisenge; Robert Rutayisire; Sabin Nsanzimana; Swaibu Gatare; Sébastien Bontems; Vincent Bours; Yvan Butera
EPI_ISL_700471, EPI_ISL_700472, EPI_ISL_700486, EPI_ISL_700511, EPI_ISL_700580	Nyanga CDC wc NGC	NHLS/UCT	Arash Iranzadeh; Bruna Galvao; Carolyn Williamson; Deelan Doolabh; Diana Hardie; Innocent Mudau; Kruger Marais; Lynn Tyers; Marvin Hsiao; Stephen Korsman
EPI_ISL_667124, EPI_ISL_667441, EPI_ISL_1541554	OHSU Lab Services Molecular Microbiology Lab	Oregon SARS-CoV-2 Genome Sequencing Center	Alec J. Hirsch; Andrew C. Adey; Benjamin N. Bimber; Brendan L. O'Connell; Brian J. O'Roak; Daniel N. Streblow; Donna Hansel; Guang Fan; Kayla Carter; Ruth V. Nichols; Sally Grindstaff; Sonia Acharya; William B. Messer; Xuan Qin
EPI_ISL_1415421	OSPEDALE CIVILE SANT'OMERO - PRONTO SOCCORSO SANT'OMERO(TERAMO)	Istituto Zooprofilattico Sperimentale dell'Abruzzo e Molise "G. Caporale"	Ancora M; Calistri P; Cammà C; Caporale M; Curini V; Delli Compagni E; Di Domenico M; Di Lollo Valeria; Di Pasquale A; Lorusso A; Mangone I; Marcacci M; Puglia I; Rinaldi A; Savini G; Scialabba S
EPI_ISL_889397	Olomouc University Hospital	Institute of Applied Biotechnologies a.s.	Kateřina Kvapilová; Marián Hajdúch; Petr Kašný; Ondřej Brzoň; Petr Klempt; Petr Kvapil
EPI_ISL_491978	Oman-NIC	Department of Microbiology and Immunology-SQUH	Abdulla Balkhair; Ahlam Al-Amri; Aisha Al-Amri; Aisha Al-Busaidi; Amina Al Jardani; Fahad Zadjali; Fatma BaAlawi; Hamida AL Barwani; Hanan Al-kindi; Intisar Al-Shukri; Khulood Al-Mammary; Mohammed Al-Tobi; Samiha Al Kharusi; Samira Al-Maruqi; Zeyana AL-Dahmani
EPI_ISL_457701, EPI_ISL_457704, EPI_ISL_457994	Oman-NIC	Oman-NIC	Abdulla Balkhair; Ahlam Al-Amri; Aisha Al-Amri; Aisha Al-Busaidi; Amina Al Jardani; Fahad Zadjali; Fatma BaAlawi; Hamida AL Barwani; Hanan Al-kindi; Intisar Al-Shukri; Khulood Al-Mammary; Mohammed Al-Tobi; Samiha Al Kharusi; Samira Al-Maruqi; Zeyana AL-Dahmani
EPI_ISL_491116, EPI_ISL_491167, EPI_ISL_1532283, EPI_ISL_1532285, EPI_ISL_1532303	Oman-National Influenza Center	Biotechnology & OMICs Laboratory	Abdul Latif Khan; Adil Al-Wahaibi; Adil Khan; Ahlam Al-Amri; Ahmed Al Harrasi; Ahmed Al-Harrasi; Ahmed Al-Rawahi; Ahmed N Al-Rawahi; Aisha Al-Amri; Aisha Al-Busaidi; Amal Al-Maani; Amina Al-Jardani; Amina Al-Jardani.; Bilal Hussain; Hanan Al-Kindi; Intisar Al-Shukri; Sajjad Asaf; Samiha Al-Kharusi; Samira Al-Mahruqi; Samiya Al-Zadjali; Saqib Bilal; Seif Al-Abri; Seif Al-Abri.
EPI_ISL_518823, EPI_ISL_518837	Oman-National Influenza Center	Biotechnology & OMICs Laboratory, Natural & Medical Sciences Research Center, University of Nizwa	Abdul Latif Khan; Adil Al-Wahaibi; Adil Khan; Ahlam Al-Amri; Ahmed Al-Harrasi; Ahmed Al-Rawahi; Aisha Al-Amri; Aisha Al-Busaidi; Amina Al-Jardani; Hanan Al-Kindi; Intisar Al-Shukri; Sajjad Asaf; Samiha Al-Kharusi; Samira Al-Mahruqi; Seif Al-Abri
EPI_ISL_766569	Oman-National Influenza Center	Oman-National Influenza Center	Aisha Al-Busaidi; Amina Al-Jardani; Hamida Al-Barwani; Hanan Al-Kindi; Intisar Al-Shukri; Laila Al-Balushi; Samiha Al-Kharusi; Samira Al-Mahruqi
EPI_ISL_1381300	Omics Sciences Laboratory	Omics Sciences Laboratory	Darlyn Amaya; Derly Andrade Molina; Emily Sulay Saltos Montalvo; Gabriel Morey León; Juan Carlos Fernández Cadena; Katheryn Sacheri Viteri; Paula Juliana Gavilanes Jarrín; Rubén Armas González
EPI_ISL_1623698, EPI_ISL_1623699	Oregon State Public Health Laboratory	Oregon State Public Health Laboratory	Eugene Yeboah; John Fontana and Shane Sevey; Laura Tsaknaridis; Rafia Razzaque; Vanda Makris
EPI_ISL_1179484, EPI_ISL_1546610	Originating lab: Wales Specialist Virology Centre Sequencing lab: Pathogen Genomics Unit	Public Health Wales Microbiology Cardiff Wales Specialist Virology Centre	Alec Birchley; Alexander Adams; Amy Gaskin; Angela Marchbank; Bree Gatica-Wilcox; Catherine Moore; Jason Coombes; Joanne Watkins; Joel Southgate; Johnathan Evans; Laura Gifford; Lauren Gilbert; Lee Graham; Malorie Perry; Matthew Bull; Nicole Pacchiarini; Sally Corden; Sara Kumziene-Summerhayes; Sara Rey; Sarah Taylor; Simon Cottrell; Sophie Jones; Tom Connor
EPI_ISL_833257	Ospedale Civile Atri	Istituto Zooprofilattico Sperimentale dell'Abruzzo e Molise "G. Caporale"	Ancora M; Calistri P; Cammà C; Curini V; Di Domenico M; Di Pasquale A; Lorusso A; Mangone I; Marcacci M; Puglia I; Rinaldi A; Savini G
EPI_ISL_640017, EPI_ISL_700447, EPI_ISL_700575	Oudtshoorn Hospital wc OUD	NHLS/UCT	Arash Iranzadeh; Bruna Galvao; Carolyn Williamson; Deelan Doolabh; Diana Hardie; Innocent Mudau; Kruger Marais; Lynn Tyers; Marvin Hsiao; Stephen Korsman
EPI_ISL_995331, EPI_ISL_995340, EPI_ISL_995342, EPI_ISL_995348, EPI_ISL_995353, EPI_ISL_995359, EPI_ISL_995363, EPI_ISL_995365, EPI_ISL_995372, EPI_ISL_995378, EPI_ISL_995381, EPI_ISL_995384, EPI_ISL_995387, EPI_ISL_995388, EPI_ISL_995389, EPI_ISL_995392, EPI_ISL_995393, EPI_ISL_995394, EPI_ISL_995398, EPI_ISL_995400, EPI_ISL_995401, EPI_ISL_995402, EPI_ISL_995403, EPI_ISL_995405, EPI_ISL_995411, EPI_ISL_995412, EPI_ISL_995414, EPI_ISL_995421, EPI_ISL_995428, EPI_ISL_995437, EPI_ISL_995439, EPI_ISL_1201516, EPI_ISL_1301899, EPI_ISL_1416972, EPI_ISL_1416988			
see above	Outre mer	National Reference Center for Viruses of Respiratory Infections, Institut Pasteur, Paris	Angela Brisebarre; Camille Capel; Christophe Malabat; Combe Patrice; Corinne Maufrais; Esther Gyde; Etienne Simon-Lorière; Frédéric Lemoine; GréGoire Potiron; Louise Lefrançois; Marion Barbet; Maud Vanpeene; Méline Bizard; Sylvie Behillil; Sylvie van der Werf; Vincent Enouf
EPI_ISL_999318	Oxford Viromics, NDM, University of Oxford; Oxford University Hospitals; Basingstoke and North Hampshire Hospital	COVID-19 Genomics UK (COG-UK) Consortium	Alex Mobbs; Amy Trebes; Anita Justice; Catrin Moore; Christophe Fraser; David Bonsall; David Buck; Emma Wise; George Macintyre; Jessica Lynch; John Todd; Mariateresa de Cesare; Matilde Mori; Monique Andersson; Nathan Moore; Nick Cortes; Robert Shaw; Stephen Kidd; Tanya Golubchik; Timothy Peto
EPI_ISL_1445207	PPA MARCIO VALDEVINO BATISTA	Instituto Butantan / Mendelics	Antonio Jorge Martins; Bibiana Santos; Claudia Renata dos Santos Barros; David Schlesinger; Debora Botequio Moretti; Dimas Tadeu Covas; Elaine Cristina Marqueze; Elaine Vieira dos Santos; Erika Freitas; Evandra Strazza Rodrigues; Flavia Aburjaile; José Salvatore Leister Patané; João Paulo Kitajima; Luiz Carlos Junior de Alcantara; Maria Carolina Elias; Marta Giovanetti; Rafael dos Santos Bezerra; Raul Machado Neto; Ricardo Haddad; Rodrigo Tocantins Calado.; Sandra Coccuzzo Sampaio; Simone Kashima; Svetoslav Nanev Slavov; Vagner Fonseca; Vincent Louis Viala
EPI_ISL_700451, EPI_ISL_700586, EPI_ISL_1040799	Pacaltsdorp Clinic wc PAC	NHLS/UCT	Arash Iranzadeh; Bruna Galvao; Carolyn Williamson; Deelan Doolabh; Diana Hardie; Houriiyah Tegally; Innocent Mudau; Kruger Marais; Lynn Tyers; Marvin Hsiao; Stephen Korsman
EPI_ISL_596507, EPI_ISL_596517, EPI_ISL_596544, EPI_ISL_596547, EPI_ISL_596551	Palestinian Ministry of Health	Molecular Genetics Lab	Damien Richard; Dana Najjar; Francois Balloux; Hisham Darwish; Husam Sallam; Issa Shtayeh; Lucy van Dorp; Mahmoud Ruzayqat; Nouar Qutob; Osama Najjar; Zaidoun Salah
EPI_ISL_955905	Pamela Youde Nethersole Eastern Hospital	Hong Kong Department of Health	Alan K.L. Tsang; Dominic N.C. Tsang; Edman T.K. Lam; Peter C.W. Yip; Rickjason C.W. Chan
EPI_ISL_1098056	Pandemic Response Lab - NYC	Pandemic Response Lab, R&D	Cybill del Castillo; Haiping Hao; Henry Lee; Jon Laurent; Melissa Hopkins; Michael Hammerling; Pradeep Bugga; Shinyoung Clair Kang; William Ward
EPI_ISL_812922	Pasteur Institute in Ho Chi Minh city	Department of Microbiology and Immunology - Pasteur Institute in Ho Chi Minh city	Anh Hoang Nguyen; Hang Thi Thu Pham; Hieu Minh Le; Hieu Trung Nguyen; Lan Trong Phan; Loan Thi Kim Huynh; Long Thanh Nguyen; Manh Huy Dao; Nhung Pham Hong Vu; Quang Chan Luong; Quang Duy Pham; Thang Minh Cao; Thao Thi Ngoc Nguyen; Thinh Viet Nguyen; Thuong Vu Nguyen.
EPI_ISL_660148, EPI_ISL_660150	PathCare	National Health Laboratory Service (NHLS), Tygerberg	Bronwyn Kleinhans; Davis M-A; Draper C; Eduan Wilkindon; Gert van Zyl; Houriiyah Tegally; Hsiao M; Kayla Delaney; Siegfried N; Susan Engelbrecht; Tulio de Oliveira; Williamson C; Wolfgang Preiser
EPI_ISL_456171, EPI_ISL_456224, EPI_ISL_582019, EPI_ISL_622805, EPI_ISL_622806	PathLab Bay of Plenty	Institute of Environmental Science and Research (ESR)	Anja Werno; Antje van der Linden; Arlo Upton; Chris Mansell; David Hammer; Dragana Drinkovic; Erasmus Smit; Gary McAuliffe; Hana Sofia Andersson; Hermes Perez; James Ussher; Jill Sherwood; Jing Wang; Joep de Ligt; Josh Freeman; Julia Howard; Juliet Elvy; Lauren Jelly; Mary DeAlmeida; Matt Blakiston; Matt Storey; Matthew Rogers; Max Bloomfield; Michael Addidle; Michelle Balm; Muhammad Faisal; Nikki Freed; Olin Silander; Sally Roberts; Sarah Jefferies; Sharmini Muttaiyah; Susan Morpeth; Susan Taylor; Timothy Blackmore; Vani Sathyendran; Veronica Playle; Virginia Hope; Xiaoyun Ren
EPI_ISL_420456, EPI_ISL_470833, EPI_ISL_470843, EPI_ISL_470851	PathWest Laboratory Medicine WA	PathWest Laboratory Medicine WA	Avram Levy; Chisha Sikazwe; David Smith and David Speers; Jurissa Lang
EPI_ISL_512716, EPI_ISL_594167, EPI_ISL_596698, EPI_ISL_672621, EPI_ISL_857487, EPI_ISL_933788, EPI_ISL_933793, EPI_ISL_933799, EPI_ISL_1069391, EPI_ISL_1069392, EPI_ISL_1069393, EPI_ISL_1198800, EPI_ISL_1295930, EPI_ISL_1295935, EPI_ISL_1384812, EPI_ISL_1416322, EPI_ISL_1508992			
see above	PathWest Laboratory Medicine WA	PathWest Laboratory Medicine WA Microbial Surveillance Unit	PathWest Laboratory Medicine WA Microbial Surveillance Unit
EPI_ISL_667620, EPI_ISL_684717, EPI_ISL_686161, EPI_ISL_686461, EPI_ISL_687985, EPI_ISL_691254, EPI_ISL_691273, EPI_ISL_691974, EPI_ISL_768703, EPI_ISL_894451, EPI_ISL_901057, EPI_ISL_1123676, EPI_ISL_1128678, EPI_ISL_1430001, EPI_ISL_1430887, EPI_ISL_1432780			
see above	Pathogen Genomics Center, National Institute of Infectious Diseases	Pathogen Genomics Center, National Institute of Infectious Diseases	Kentaro Itokawa; Makoto Kuroda; Masanori Hashino; Rina Tanaka; Tsuyoshi Sekizuka
EPI_ISL_437692, EPI_ISL_437748, EPI_ISL_512948, EPI_ISL_513039, EPI_ISL_513046, EPI_ISL_513188, EPI_ISL_513193, EPI_ISL_678214			
see above	Pathogen Genomics Lab King Abdullah University of Science and Technology(KAUST)	Pathogen Genomics Lab King Abdullah University of Science and Technology(KAUST)	Abbas Al Mutair; Abdulaziz Alahmadi; Afrah Alsomali; Ahmad Bakur Mahmoud; Amanda Ooi; Amit Kumar Subudhi; Anwar Hashem; Arnab Pain; Asim Khogeer; Awad Al-Omari; Fadwa Alofi; Fathia Ben Rached; Fathia Ben-Rached; Jumana Taha; Kahled Algithami; Khaled Algithami; Luke Esau; Muhammad Shuaib; Naif Almontashiri; Nashwa Al-khotani; Olga Douvropoulou; Raeece Naeem; Rahul P Salunke; Rahul Salunke; Raushan Nugmanova; Samer Salih; Sara Mfarrej; Sharif Hala
EPI_ISL_1627576, EPI_ISL_1627577	Pathogenic Microorganisms Variability Laboratory	WHO National Influenza Centre Russian Federation	Alexander Gintsburg; Alexey Shchetinin; Alina Odintsova; Andrei Siniavin; Andrey Komissarov; Andrey Pochtovyy; Artem Fadeev; Artem Tkachuk; Daria Danilenko; Denis Logunov; Dmitry Lioznov; Elena Nabieva; Elena Shidlovskaya; Elizaveta Divisenko; Evgeny Usachev; Georgii Bazykin; Ksenia Safina; Kseniya Komissarova; Ludmila Kolobukhina; Lyudmila Vasilchenko; Maria Nikiforova; Nadezhda Kuznetsova; Olga Burgasova; Svetlana Smetanina; Vladimir Gushchin
EPI_ISL_806719	Pathology North - Royal North Shore Hospital - NSW Health Pathology	NSW Health Pathology - Institute of Clinical Pathology and Medical Research; Westmead Hospital; University of Sydney	CIDM-PH et al.
EPI_ISL_407894, EPI_ISL_414414	Pathology Queensland	Public Health Virology Laboratory	Alyssa Pyke; Amanda De Jong; Andrew Van Den Hurk; Ben Huang; Bixing Huang; Carmel Taylor; David Warrilow; Doris Genge; Elisabeth Gamez; Glen Hewitson; Ian Maxwell Mackay; Inga Sultana; Jamie McMahon; Jean Barcelon; Judy Northill; Mitchell Finger; Natalie Simpson; Neelima Nair; Peter Burtonclay; Peter Moore; Sarah Wheatley; Sean Moody; Sonja Hall-Mendelin; Timothy Gardam; and Frederick Moore; and Frederick Moore.
EPI_ISL_455071	Pathology Sydney South West - NSW Health Pathology	NSW Health Pathology - Institute of Clinical Pathology and Medical	CIDM-PH et al.

		Research; Westmead Hospital; University of Sydney	
EPI_ISL_455033, EPI_ISL_545006	Pathology West - NSW Health Pathology	NSW Health Pathology - Institute of Clinical Pathology and Medical Research; Westmead Hospital; University of Sydney	CIDM-PH et al.
EPI_ISL_1600064	Platform BIS UZA/UAntwerpen	Labo Klinische Biologie, UZA	Basil Britto Xavier; Christine Lammens; Herman Goossens; Jasmine Coppens; Marie Le Mercier; Veerle Matheeussen
EPI_ISL_522547	Platforme CYROI	UMR PIMIT Université de La Réunion	Camille Lebarbenchon; David Wilkinson; Patrick Mavingui
EPI_ISL_700518, EPI_ISL_700528, EPI_ISL_700560	Plettenberg Bay Clinic wc PLC	NHLS/UCT	Arash Iranzadeh; Bruna Galvao; Carolyn Williamson; Deelan Doolabh; Diana Hardie; Innocent Mudau; Kruger Marais; Lynn Tyers; Marvin Hsiao; Stephen Korsman
EPI_ISL_745187	Port Nolloth Hospital	National Health Laboratory Service (NHLS), Tygerberg	Bronwyn Kleinhans; Eduan Wilkindon; Gert van Zyl; Houriiyah Tegally; Kayla Delaney; Susan Engelbrecht; Tulio de Oliveira; Wolfgang Preiser
EPI_ISL_404895	Providence Regional Medical Center	Division of Viral Diseases, Centers for Disease Control and Prevention	C.R.; Gerber; Li, Y.; Lindstrom, S.; Lu, X.; Paden; Queen, K.; S.I.; Tao, Y.; Tong, S.; Zhang, J.
EPI_ISL_1180682	Public Health Authority of the Slovak Republic	Bergthaler laboratory, CeMM Research Center for Molecular Medicine of the Austrian Academy of Sciences	Andreas Bergthaler; Anna Schedl; Bekir Erguner; Benedikt Agerer; Christoph Bock; Fabian Amman; Jan Laine; Lukas Endler; Maelle Le Moing; Martin Senekowitsch; Michael Schuster; Thomas Penz
EPI_ISL_1647630, EPI_ISL_1647695, EPI_ISL_1656821	Public Health Authority of the Slovak Republic	Laboratory of Genomics and Bioinformatics, Comenius University Science Park	Anna Gičová; Diana Rusňáková; Jaroslav Budiš; Miroslav Böhmer; Tatiana Sedláčková; Tomáš Szemes
EPI_ISL_1112317, EPI_ISL_1112321	Public Health Center of Ukraine	Charité Universitätsmedizin Berlin, Institute of Virology	Barbara Mühlemann; Christian Drosten; Ihor Kuzin; Iryna Demchyshyna; Julia Schneider; Jörn Beheim-Schwarzbach; Liudmyla Chernenko; Roman Rodyna; Talitha Veith; Terry Jones; Victor M Corman
EPI_ISL_636973	Public Health Lab	Public Health Lab	Alwasti; H
EPI_ISL_985235, EPI_ISL_1061310, EPI_ISL_1137505	Public Health Virology-Forensic and Scientific Services	Public Health Virology-Forensic and Scientific Services	Son Nguyen; Son Nguyen et al
EPI_ISL_1495192	Public Health Authority of the Slovak Republic	Bergthaler laboratory, CeMM Research Center for Molecular Medicine of the Austrian Academy of Sciences	Andreas Bergthaler; Anna Schedl; Bekir Erguner; Benedikt Agerer; Christoph Bock; Fabian Amman; Jan Laine; Lukas Endler; Maelle Le Moing; Martin Senekowitsch; Michael Schuster; Petr Triska; Thomas Penz
EPI_ISL_1055494, EPI_ISL_1055545, EPI_ISL_1055675, EPI_ISL_1055688	QEII Health Sciences Centre	National Microbiology Laboratory (NML)	Anna Majer; Anneliese Landgraff; CanCOGeN's metadata curation team; Dan Gaston; Darian Hole; Elsie Grudeski; Gary Van Domselaar; Grace Seo; Janice Pettipas; Jason LeBlanc; Jennifer Tanner; Kirsten Biggar; Madison Chapel; Morag Graham; Natalie Knox; Nathalie Bastien; Philip Mabon; Public Health Agency of Canada CanCOGeN team; Rhiannon Huzarewich; Russell Mandes; Shari Tyson; Timothy Booth; Todd Hatchette; Yan Li
EPI_ISL_639759, EPI_ISL_639818, EPI_ISL_639819, EPI_ISL_693269, EPI_ISL_693282, EPI_ISL_693290, EPI_ISL_693292, EPI_ISL_849683, EPI_ISL_849685, EPI_ISL_849686, EPI_ISL_849691, EPI_ISL_849692, EPI_ISL_849693, EPI_ISL_849731, EPI_ISL_849744, EPI_ISL_849755, EPI_ISL_944737, EPI_ISL_944742, EPI_ISL_962180, EPI_ISL_968212, EPI_ISL_1159380, EPI_ISL_1300524, EPI_ISL_1300529, EPI_ISL_1300533, EPI_ISL_1340874, EPI_ISL_1396522, EPI_ISL_1404920, EPI_ISL_1465885, EPI_ISL_1465892, EPI_ISL_1483027, EPI_ISL_1483028, EPI_ISL_1483030			
see above	Queensland Health Forensic and Scientific Services	Queensland Health Forensic and Scientific Services	Son Nguyen; Son Nguyen et al
EPI_ISL_530240, EPI_ISL_530245	Queensland Health Forensic and Scientific Services, Public Health Virology	Public Health Virology Laboratory, Forensic and Scientific Services, Queensland Health	Son Nguyen et al
EPI_ISL_1424519, EPI_ISL_1424628	Queensland Medical Laboratories	Victorian Infectious Diseases Reference Laboratory (VIDRL) and the Melbourne Diagnostic Unit Public Health Laboratory (MDU-PHL)	N.L.; Palou, T.; Seemann, T.; Sherry; Vaccher, S.
EPI_ISL_571980, EPI_ISL_604232, EPI_ISL_937094	Quest Diagnostics	Quest Diagnostics	Anderson, B.; D.F.; Gerasimova, A.; Grover, D.; Hua, M.; K.E.; Kagan; Lacbawan, F.; Liu Y.; Livingston; Owen, R.; R.M.; Rosenthal; S.H.; Shalhout
EPI_ISL_1254034, EPI_ISL_1367240, EPI_ISL_1479708, EPI_ISL_1479760, EPI_ISL_1480801, EPI_ISL_1494003, EPI_ISL_1552260, EPI_ISL_1582137, EPI_ISL_1621839, EPI_ISL_1621960, EPI_ISL_1648615, EPI_ISL_1648884			
see above	Quest Diagnostics Incorporated	Centers for Disease Control and Prevention Division of Viral Diseases, Pathogen Discovery	A. Gerasimova; A. Perez; Adrian Paskey; B. Anderson; Ben L. Rambo-Martin; Benjamin Rambo-Martin; Christopher Gulvick; Clinton R. Paden; Dakota Howard; Darlene Wagner; Dhwani Batra; Duncan MacCannell; F. Lacbawan; I. A. Shlyakhter; Jason Caravas; K.E. Livingston; Kara Moser; L.E. Bernstein; M. Hua; Matthew Schmerer; P. Tanpaiboon; Peter W. Cook; R. M. Kagan; R. Owen; R. V. Rolando; S. H. Rosenthal; Scott Sammons; Shatavia Morrison; Suxiang Tong; Y. Liu; Yvette Unoarumhi
EPI_ISL_1086925, EPI_ISL_1087190, EPI_ISL_1090931	Quest Diagnostics Incorporated	Respiratory Viruses Branch, Division of Viral Diseases, Centers for Disease Control and Prevention	A. Gerasimova; A. Perez; B. Anderson; Ben L. Rambo-Martin; Clinton R. Paden; Dakota Howard; Dhwani Batra; Duncan MacCannell; F. Lacbawan; I. A. Shlyakhter; K.E. Livingston; L.E. Bernstein; M. Hua; P. Tanpaiboon; Peter W. Cook; R. M. Kagan; R. Owen; R. V. Rolando; S. H. Rosenthal; Suxiang Tong; Y. Liu
EPI_ISL_415641, EPI_ISL_416477, EPI_ISL_416480	R. G. Lugar Center for Public Health Research, National Center for Disease Control and Public Health (NCDC) of Georgia.	R. G. Lugar Center for Public Health Research, National Center for Disease Control and Public Health (NCDC) of Georgia.	Adam Kotorashvili; Amiran Gamkrelidze.; Ana Papkiauri; Ann Machablishvili; Anna Kasradze; Davit Tsaguria; Ekaterine Khmaladze; Ekaterine Zangaladze; Ekaterine Zhghenti; Giorgi Tomashvili; Gvantsa Brachveli; Gvantsa Chanturia; Irma Burjanadze; Ketevan Sidamonidze; Khatuna Zakhashvili; Lela Sabadze; Lela Urushadze; Magda Dgebuadze; Maia Alkhazashvili; Mari Gavashelidze; Mariam Zakalashvili; Marine Murtskhvaladze; Meri Pantsulaia; Nato Kotaria; Nino Berishvili; Paata Imnadze; Roena Sukhiashvili; Tamar Jashiashvili; Tata Imnadze; Tea Tevdoradze
EPI_ISL_1063843	REGIONAL VRDL, ICMR-RMRC BBSR	Immunogenomics lab, Institute of Life Sciences, Bhubaneswar	Ajay Parida; Amol M. Kanampalliwar; Arup Ghosh; Atimukta Jha; INSACOG Consortium; Kirtal Hansdah; Punit Prasad; Rajeeb Swain; Rupesh Dash; Safal Walia; Shifu Aggarwal; Sunil K. Raghav
EPI_ISL_1628700, EPI_ISL_1628701, EPI_ISL_1628702, EPI_ISL_1628707, EPI_ISL_1628728, EPI_ISL_1628729, EPI_ISL_1628732, EPI_ISL_1628752, EPI_ISL_1628765, EPI_ISL_1628767, EPI_ISL_1628779, EPI_ISL_1628780, EPI_ISL_1628781, EPI_ISL_1628782, EPI_ISL_1628861, EPI_ISL_1628864, EPI_ISL_1628867, EPI_ISL_1628896, EPI_ISL_1628903, EPI_ISL_1628910, EPI_ISL_1628912, EPI_ISL_1629201, EPI_ISL_1629202, EPI_ISL_1629203, EPI_ISL_1629206, EPI_ISL_1629207, EPI_ISL_1629222, EPI_ISL_1629272, EPI_ISL_1629310, EPI_ISL_1629315, EPI_ISL_1629325, EPI_ISL_1629326, EPI_ISL_1629333, EPI_ISL_1629345, EPI_ISL_1629346, EPI_ISL_1629400, EPI_ISL_1629401, EPI_ISL_1629404, EPI_ISL_1629406, EPI_ISL_1629408, EPI_ISL_1629446, EPI_ISL_1629451, EPI_ISL_1629473, EPI_ISL_1629475, EPI_ISL_1629480			
see above	REUNILAB	UMR PIMIT	Dr Camille Lebarbenchon; Dr David A Wilkinson; Dr Patrick Mavingui; Magali Turpin
EPI_ISL_419553	RI State Health Laboratories	Pathogen Discovery, Respiratory Viruses Branch, Division of Viral Diseases, Centers for Disease Control and Prevention	Anna Uehara; Clinton R. Paden; Haibin Wang; Jasmine Padilla; Jing Zhang; Justin Lee; Krista Queen; Suxiang Tong; Yan Li; Ying Tao
EPI_ISL_420795	RI State Health Laboratory	Pathogen Discovery, Respiratory Viruses Branch, Division of Viral Diseases, Centers for Disease Control and Prevention	Alison S. Laufer Halpin; Anne Uehara; Christopher A. Elkins; Clinton R. Paden; Haibin Wang; Jasmine Padilla; Jing Zhang; Justin Lee; Krista Queen; Mary S. Keckler; Rachel Marine; Suxiang Tong; Yan Li; Ying Tao
EPI_ISL_889017	RS Hermina Tangerang	Eijkman Institute for Molecular Biology, Ministry of Research and Technology/National Agency for Research and Innovation	Amin Soebandrio; Edison Johar; Frilasita A Yudhaputri; Hidayat Trimarsanto; Iskandar Adnan; Khin Saw Myint; Lydia V. Panggalo; Safarina G Malik; Sukma Oktavianthi; Willy Agustine
EPI_ISL_1469285	RS Khusus Paru Karawang	National Institute of Health Research and Development	Arie Ardiansyah Nugraha; Hana Apsari Pawestri; Hartanti Dian Ikawati; Kartika Dewi Puspa; Krisna Pangesti; Nelly Puspandari; Subangkit; Vivi Setiawaty
EPI_ISL_538499	RS Lavallete Malang East Java	National Institute of Health Research and Development	AA; HA; HD; Ikawati; KD; KNA; Nugraha; Paisal; Pangesti; Pawestri; Puspa; Setiawaty, V.; Soekarso; Subangkit; T
EPI_ISL_888992	RS Mitra Keluarga Gading Serpong	Eijkman Institute for Molecular Biology, Ministry of Research and Technology/National Agency for Research and Innovation	Amin Soebandrio; Edison Johar; Frilasita A Yudhaputri; Hidayat Trimarsanto; Iskandar Adnan; Khin Saw Myint; Lydia V. Panggalo; Safarina G Malik; Sukma Oktavianthi; Willy Agustine
EPI_ISL_1622427	RS OMNI Pulomas	Eijkman Institute for Molecular Biology, Ministry of Research and Technology/National Agency for Research and Innovation	Amin Soebandrio; Edison Johar; Frilasita A Yudhaputri; Hidayat Trimarsanto; Iskandar Adnan; Khin Saw Myint; Lydia V. Panggalo; Muhammad Rezki Rasyak; Safarina G Malik; Sukma Oktavianthi; Willy Agustine
EPI_ISL_454516	RSE "National Center for Biotechnology"	RSE "National Center for Biotechnology"	Akbota Rakhmetova; Alexandr Shevtsov; Askar Abdaliyev; Asylulan Amirgazin; Ilyas Akhmetollayev; Ruslan Kalendar; Viktoriya Lutsay; Yerlan Ramankulov; Zabira Aushakhmetova
EPI_ISL_574609	RSJPD Harapan Kita	Eijkman Institute for Molecular Biology, Ministry of Research and Technology/National Agency for Research and Innovation	Amin Soebandrio; David H Muljono; Edison Johar; Frilasita A Yudhaputri; Herawati Sudoyo; Hidayat Trimarsanto; Iskandar A Adnan; Khin Saw Myint; Safarina G Malik; Willy Agustine
EPI_ISL_568686	RSUD Prof. DR. Margono Soekarjo	Eijkman Institute for Molecular Biology, Ministry of Research and Technology/National Agency for Research and Innovation	Amin Soebandrio; David H Muljono; Edison Johar; Frilasita A Yudhaputri; Herawati Sudoyo; Hidayat Trimarsanto; Iskandar A Adnan; Khin Saw Myint; Safarina G Malik; Willy Agustine
EPI_ISL_791983	RSUD dr. Kanujoso Djatiwibowo Balikpapan	National Institute of Health Research and Development	AA; HA; HD; Ikawati; Ivanna; KD; KNA; N; Nugraha; Pangesti; Pawestri; Puspa; Puspandari; Setiawaty; Soekarso; Subangkit; T; V
EPI_ISL_1169047	RSUP Dr. Mohammad Hoesin Palembang Sumatera Selatan	National Institute of Health Research and Development	Arie Ardiansyah Nugraha; Hana Apsari Pawestri; Hartanti Dian Ikawati; Kartika Dewi Puspa; Nelly Puspandari; Subangkit; Vivi Setiawaty
EPI_ISL_862040	RSUP Dr. Sardjito	Genetics Working Group (Pokja Genetik) Faculty of Medicine, Public Health and Nursing Universitas Gadjah Mada (FK-KMK UGM); Disease Investigation Center Wates Ministry of Agriculture Indonesia; Department of Microbiology FK-KMK UGM; Laboratorium Diagnostik Yayasan Tahija World Mosquito Program (WMP) Yogyakarta Center for Tropical Medicine FK-KMK UGM; Integrated Research Center FK-KMK UGM; Department of Computer Science and Electronics FMIPA UGM; RSUP Dr. Sardjito	. Marcellus; Afiahayati; Alvin Santoso Kalim; Bambang Sigit Riyanto; Dwi AA Nugrahaningsih; Edwin W. Daniwijaya; Eggi Arguni; Eko Budiono; Endah Supriyati; Gunadi; Hendra Wibawa; Heni Retnowulan; Ika Trisnawati; Ira Puspitawati; Kristy Iskandar; Ludhang P. Rizki; Mohamad S. Hakim; Munawar Gani; Nungki Anggorowati; Nur Imma Fatimah Harahap; Nur Rahmi Ananda; Osman Sianipar; Riat El Khair; Satria Maulana; Siswanto; Sumardi; Titik Nuryastuti; Tri Wibawa; Umi Solekhah Intansari; Untung Riawan; Yunika Puspadewi; Elizabeth Henny Herningtiyas
EPI_ISL_450512, EPI_ISL_450514	Rafik Hariri University Hospital	Rafik Hariri University Hospital	Rita Feghali
EPI_ISL_640136	Red Cross Children's Hospital wc RXH	NHLS/UCT	Arash Iranzadeh; Bruna Galvao; Carolyn Williamson; Deelan Doolabh; Diana Hardie; Innocent Mudau; Kruger Marais; Lynn Tyers; Marvin Hsiao; Stephen Korsman
EPI_ISL_1415415, EPI_ISL_1415417, EPI_ISL_1415418	Reference Laboratory of the Ministry of Health	Laboratory of Respiratory Viruses and Measles, Oswaldo Cruz Institute, FIOCRUZ	Alice Sampaio Rocha; Ana Carolina Mendonca; Anna Carolina Paixao; Fernando Motta; Indira Martins; Jessica Edwards; Luciana Appolinario; Marilda Siqueira on behalf of the Fiocruz COVID-19 Genomic Surveillance Network; Paola Resende; Renata Serrano Lopes
EPI_ISL_768530	Regional Medical Sciences Center 5 Samut Songkhram	National Institute of Health, Department of Medical Sciences, Ministry of Public Health, Thailand	; Natchaya Khiadsang; Pakorn Piromtong; Pilailuk Okada; Ratana Tacharoenmuang; Siripaporn Phuygun; Sittiporn Parnmen; Sunthareeya Waicharoen; Thanutsapa Thanadachakul; Warawan Wongboot; sirikanda wimol
EPI_ISL_708800	Regional medical sciences center 6 chonburi	National Institute of Health, Department of Medical Sciences, Ministry of Public Health, Thailand	Malinee Chittaganpitch; Pilailuk Okada; Siripaporn Phuygun; Sittiporn Parnmen; Sunthareeya Waicharoen; Thanutsapa Thanadachakul; Warawan Wongboot
EPI_ISL_906851	Respiratory Viruses Branch, Centers for Disease Control and Prevention	Respiratory Viruses Branch, Centers for Disease Control and Prevention	C.R.; Cook, P.; Li, Y.; Paden; Queen, K.; Tao, Y.; Tong, S.; Uehara, A.; Wang, H.; Zhang, J.

EPI_ISL_1394781	Respublikine Siaulie ligonine	Lithuanian University of Health Sciences Hospital, Department of Genetics and Molecular Medicine	Astra Vitkauskiene; Darius Cereskevicius; Inga Nasvytiene; Mantas Sarauskas; Marius Sukys; Rasa Ugenskiene; Renaldas Jurkevicius; Rima Vainoriene; Zivile Zemeckiene
EPI_ISL_1040778	Retreat CHC wc RHC	NHLS/UCT	Arash Iranzadeh; Bruna Galvao; Carolyn Williamson; Deelan Doolabh; Diana Hardie; Innocent Mudau; Kruger Marais; Lynn Tyers; Marvin Hsiao; Stephen Korsman
EPI_ISL_1590501, EPI_ISL_1590805	Riga East University Hospital-National Microbiology Reference Laboratory; Eurofins Genomics Europe Sequencing GmbH	Riga East University Hospital-National Microbiology Reference Laboratory; Eurofins Genomics Europe Sequencing GmbH	Arzu Algulieva; Diāna Dusacka; Dārta Pūpola; Ilva Pole; Reinis Vangravs; Reinis Zeltmatis; Sergejs Nikisins; Ģirts Šķenders
EPI_ISL_700465	Riversdale Clinic wc RAV	NHLS/UCT	Arash Iranzadeh; Bruna Galvao; Carolyn Williamson; Deelan Doolabh; Diana Hardie; Innocent Mudau; Kruger Marais; Lynn Tyers; Marvin Hsiao; Stephen Korsman
EPI_ISL_812423	Royal Darwin Hospital Pathology	MDU-PHL	Caly L.; Druce J.; M.L.; Meumann, E.; N.L.; Sait; Seemann T.; Sherry
EPI_ISL_522705	Royal Hobart Hospital Microbiology Department	MDU-PHL	Cooley L.; M.B.; Sait M.; Schultz; Seemann T.; Sherry N.; van Haeften R.
EPI_ISL_857395	S.M.S.Medical College, Jaipur, Rajasthan	S.M.S.Medical College, Jaipur, Rajasthan	Dr. Bharti Malhotra; Dr. Swati Gautam
EPI_ISL_468032, EPI_ISL_510544, EPI_ISL_516551, EPI_ISL_732960, EPI_ISL_752598, EPI_ISL_755568, EPI_ISL_855344			
see above	SA Pathology	SA Pathology	Chuan Kok Lim; Geoff Higgins; Ivan Bastian; Julien Soubrier; Karin Kassahn; Lex Leong; Luke Walters; Mark Turra; Song Gao
EPI_ISL_1138512, EPI_ISL_1138513, EPI_ISL_1138514, EPI_ISL_1138515, EPI_ISL_1138517	SA Pärnu Hospital Laboratory	1. Laboratory of Communicable Diseases (Estonia); 2. Eurofins Genomics Europe Sequencing GmbH	Liidia Dotsenko
EPI_ISL_1628725, EPI_ISL_1628726, EPI_ISL_1628730, EPI_ISL_1628745, EPI_ISL_1629398	SAINT BENOIT	UMR PIMIT	Dr Camille Lebarbenchon; Dr David A Wilkinson; Dr Patrick Mavingui; Magali Turpin
EPI_ISL_1094374	SD Public Health Laboratory	Respiratory Viruses Branch, Division of Viral Diseases, Centers for Disease Control and Prevention	Anna Montmayeur; Anna Uehara; Ben L. Rambo-Martin; Clinton R. Paden; Dhwani Batra; Haibin Wang; Jasmine Padilla; Jing Zhang; Justin Lee; Krista Queen; Lori Rowe; Mark Burroughs; Mili Sheth; Peter W. Cook; Rachel Marine; Sarah Nobles; Suxiang Tong; Yan Li; Ying Tao
EPI_ISL_1445240	SECAO CENTRO DE DIAGNOSTICO SECEDI	Instituto Butantan / Mendelics	Antonio Jorge Martins; Bibiana Santos; Claudia Renata dos Santos Barros; David Schlesinger; Debora Botequio Moretti; Dimas Tadeu Covas; Elaine Cristina Marqueze; Elaine Vieira dos Santos; Erika Freitas; Evandra Strazza Rodrigues; Flavia Aburjaile; José Salvatore Leister Patané; João Paulo Kitajima; Luiz Carlos Junior de Alcantara; Maria Carolina Elias; Marta Giovanetti; Rafael dos Santos Bezerra; Raul Machado Neto; Ricardo Haddad; Rodrigo Tocantins Calado.; Sandra Coccuzzo Sampaio; Simone Kashima; Svetoslav Nanev Slavov; Vagner Fonseca; Vincent Louis Viala
EPI_ISL_1580596	SIESP CHIETI - DRIVE IN ORTONA ORTONA(CHIETI)	Istituto Zooprofilattico Sperimentale dell'Abruzzo e Molise "G. Caporale"	Ancora M; Calistri P; Cammà C; Caporale M; Curini V; Delli Compagni E; Di Domenico M; Di Lollo Valeria; Di Pasquale A; Lorusso A; Mangone I; Marcacci M; Puglia I; Rinaldi A; Savini G; Scialabba S
EPI_ISL_1228017	SUNY UPSTATE MEDICAL UNIVERSITY	Wadsworth Center, New York State Department of Health	Alexis Russel; Daryl M. Lamson; Erasmus Schneider; Erica Lasek-Nesselquist; John Kelly; Jonathan Plitnick; Kirsten St. George; Matthew Shudt; Melissa A Leisner; Navjot Singh
EPI_ISL_1152647, EPI_ISL_1641674	SYNLAB MVZ Trier	Robert Koch Institute	
EPI_ISL_688372, EPI_ISL_1427368, EPI_ISL_1427544	Saitama Prefectural Institute of Public Health	Pathogen Genomics Center, National Institute of Infectious Diseases	Kentaro Itokawa; Makoto Kuroda; Masanori Hashino; Rina Tanaka; Tsuyoshi Sekizuka
EPI_ISL_901735	Sakai City Institute of Public Health	Pathogen Genomics Center, National Institute of Infectious Diseases	Kentaro Itokawa; Makoto Kuroda; Masanori Hashino; Rina Tanaka; Tsuyoshi Sekizuka
EPI_ISL_750170	Sanatorio Americano	Institut Pasteur de Montevideo	Ana Carolina Mendonça; Andrés Lizasoain; Camila Simoes; Cecilia Alonso; Cecilia Salazar; Daiana Mir; Fernando López-Tort; Fernando Motta; Gonzalo Bello; Ighor Arantes; Ignacio Ferrés; Jose Sotelo; Leticia Maya; Letícia Garay Martins; Luciana Appolinario; Lucía Spangenberg; Mailen Arleo; Mariana Brandes; Marilda Mendonça Siqueira; Marilda Tereza Mar da Rosa; María José Benitez-Galeano; Martín Graña; Matías Castells; Matías Victoria; Matías Salvo; Natalia Rego; Natalia Reyes; Pablo Smircich; Paola Cristina Resende; Rodney Colina; Tamara Fernandez-Calero; Tania Possi; Tatiana Schäffer Gregianini; Verónica Noya; Yasser Vega
EPI_ISL_1468424	Santa Casa de Aracatuba Hospital Sagrado Coracao de Jesus	Instituto Adolfo Lutz, Interdiciplinary Procedures Center, Strategic Laboratory	Caio Vinicius Dias Lopes; Claudia Regina Gonçalves; Claudio Tavares Sacchi; Erica Valessa Ramos Gomes; Karoline Rodrigues Campos
EPI_ISL_672433	Santa Clara County Public Health Laboratory	Chan-Zuckerberg Biohub	CZB Cliahub Consortium
EPI_ISL_479800	Sapporo City Institute of Public Health	Pathogen Genomics Center, National Institute of Infectious Diseases	Asami Ohnishi; Hajime Kamiya; Kentaro Itokawa; Makoto Kuroda; Masanori Hashino; Motoi Suzuki; Rina Tanaka; Tsuyoshi Sekizuka
EPI_ISL_510529	School of Veterinary Medicine, Disease Control	School of Veterinary Medicine, Disease Control	A.L.; A.N.; Bates, M.; C. and Zumla, A.; Chambaro, H.; Chanda, D.; Changula, K.; Chilufya; Chipimo; Chitanga, S.; Fwoloshi, S.; K.S.; Kapata; Kapata, N.; Kapaya, F.; Kapin'a, M.; Kayeyi, N.; Liwewe; M.M.; Malama, K.; Masahiro, K.; Monze, M.; Morales; Mubemba, B.; Mukonka, V.; Mulenga, L.; Muleya, W.; Mupeta, F.; Musonda, K.; Nalubamba; Ngosa, W.; P.C.; P.J.; Saasa, N.; Sawa, H.; Shibemba; Simulundu, E.; Sinyange, N.; Takada, A.; Tembo, J.; Zulu, P.
EPI_ISL_700588	Sedgefield Clinic wc SGE	NHLS/UCT	Arash Iranzadeh; Bruna Galvao; Carolyn Williamson; Deelan Doolabh; Diana Hardie; Innocent Mudau; Kruger Marais; Lynn Tyers; Marvin Hsiao; Stephen Korsman
EPI_ISL_696462	Sedgefield Clinic wc SGE & NHLS/UCT	KRISP, KZN Research Innovation and Sequencing Platform	Arash Iranzadeh; Bruna Galvao; Carolyn Williamson; Deelan Doolabh; Diana Hardie; Emanuel James San; Houriiyah Tegally; Innocent Mudau; Jennifer Giandhari; Kruger Marais; Lynn Tyers; Marvin Hsiao; Stephen Korsman; Sureshnee Pillay; Tulio de Oliveira
EPI_ISL_408977	Serology, Virology and OTDS Laboratories (SAViD), NSW Health Pathology Randwick	NSW Health Pathology - Institute of Clinical Pathology and Medical Research; Centre for Infectious Diseases and Microbiology Laboratory Services; Westmead Hospital; University of Sydney	Carter I; Chen SC; Eden J-S; Holmes EC; Kok J and Dwyer DE for the 2019-nCoV Study Group*; Maddocks S; O'Sullivan MV; Rahman H; Rawlinson W; Rockett R; Sintchenko V
EPI_ISL_856759	Servicio Virosis Respiratorias-Departamento Virología-INEI	Instituto Nacional Enfermedades Infecciosas C.G.Malbran	Avaro M.; Baumeister E.; Benedetti E.; Campos J.; Cisterna D.; Dattero ME; Lorenzo F.; Molina V.; Perandones C.; Poklepovich T.; Pontoriero A.; Russo M.; Tuduri E.
EPI_ISL_1393025	Servicio de Microbiología Clínica (Complejo Hospitalario de Navarra, Pamplona)	Centro de Secuenciación NASERTIC	Ana Miqueleiz; Ana Navascués; Carmen Ezpeleta Baquedano
EPI_ISL_1588913	Servicio de Microbiología Clínica (Complejo Hospitalario de Navarra, Pamplona), Instituto de Investigación Sanitaria de Navarra (IdiSNA)	SeqCOVID-SPAIN consortium/IBV(CSIC)	Ana Miqueleiz and SeqCOVID-SPAIN consortium; Ana Navascués; Carmen Ezpeleta Baquedano
EPI_ISL_510390	Servicio de Microbiología, Hospital Miguel Servet, Zaragoza	SeqCOVID-SPAIN consortium/IBV(CSIC)	Alexander Tristancho Baró; Ana Milagro; Antonio Rezusta López; Nieves Martínez Cameo and SeqCOVID-SPAIN consortium; Yolanda Gracia Grataloup
EPI_ISL_1046867	Servicio de Microbiología, Laboratori Clínic Metropolitana Nord. Hospital Universitari Germans Trias i Pujol. Institut d'Investigació en Ciències de la Salut Germans Trias i Pujol (IGTP)	IrsiCaixa - Can Ruti CovidSeq	Adrián Antuori; Ana Pérez; Anna Not; Antoni E. Bordoy; Bonaventura Clotet Elisa Martró; Cristina Casañ; Cristina Esteban; Francesc Catala-Moll; Ignacio Blanco; Marc Noguera-Julian; Maria Casadellà; Mariona Parera; Montserrat Giménez; Pilar Armengol; Roger Paredes; Verónica Saludes
EPI_ISL_871934	Servicio de Microbiología, Laboratori Clínic Metropolitana Nord. Hospital Universitari Germans Trias i Pujol. Institut d'Investigació en Ciències de la Salut Germans Trias i Pujol (IGTP)	SeqCOVID-SPAIN consortium/IBV(CSIC)	Adrián Antuori; Anabel Fernández; Anna Not; Antoni E. Bordoy; Cristina Casañ and SeqCOVID-SPAIN consortium; Elisa Martró; Nona Romaní; Verónica Saludes
EPI_ISL_1647336	Servizo de Microbioloxía. Complexo Hospitalario de Santiago de Compostela	Servizo de Microbioloxía. Complexo Hospitalario de Santiago de Compostela	Amparo Coira; Antonio Aguilera; Carlos García_Riestra; Daniel Navarro; Gema Barbeito; Iria Roca; José Llovo; Laura Millán; Manuela Hernández; María Luisa Pérez_del_Molino; Mercedes Treviño; Rocío trastoy; Teresa Lopez_Valiño; Xosé Costa
EPI_ISL_962662, EPI_ISL_1081544	Sharp HealthCare Laboratory	Andersen lab at Scripps Research	Art Mendoza; Cathy Woerle; Jacquelyn Berumen; Liam McGinnis; Omid Bakhtar; SEARCH Alliance San Diego with Aaron Harding
EPI_ISL_482680	Singapore General Hospital	Department of Microbiology	Chenhao Li; Karrie Ko; Kern Rei Chng; Kian Sing Chan; Kun Lee Lim; Lynette Oon; Niranjan Nagarajan; Nurdyana Abdul Rahman
EPI_ISL_956278	Siti Khodijah Hospital	Institute of Tropical Disease, Universitas Airlangga	Aldise M Nastri; Gatot Soegiarto; Jezzy R Dewantari; Kazufumi Shimizu; Krisnoadi Rahardjo; Laksmi Wulandari; Maria I Lusida; Muhammad Hamdan; Resti Yudhawati; Rima R Prasetya; Soetjipto; Yasuko Mori
EPI_ISL_1284678	Sonic - Bioscientia - MVZ Labor Saar GmbH	Robert Koch Institute	
EPI_ISL_529197	South Carolina Department of Health and Environmental Control	South Carolina Department of Health and Environmental Control	Haley V. Flores
EPI_ISL_1253689, EPI_ISL_1314860, EPI_ISL_1578046	South Dakota Public Health Laboratory	South Dakota Public Health Laboratory	Jacob Garfin and Chris Carlson
EPI_ISL_455090, EPI_ISL_455099	South Eastern Area Laboratory Services	NSW Health Pathology - Institute of Clinical Pathology and Medical Research; Westmead Hospital; University of Sydney	CIDM-PH et al.
EPI_ISL_490042, EPI_ISL_593691, EPI_ISL_593704, EPI_ISL_629005, EPI_ISL_740874, EPI_ISL_872575, EPI_ISL_872584			
see above	South Eastern Area Laboratory Services (SEALS)	NSW Health Pathology - Institute of Clinical Pathology and Medical Research; Westmead Hospital; University of Sydney	CIDM-PH et al.
EPI_ISL_456238	Southern Community Labs Dunedin	Institute of Environmental Science and Research (ESR)	Anja Werno; Antje van der Linden; Arlo Upton; Chris Mansell; David Hammer; Dragana Drinkovic; Erasmus Smit; Gary McAuliffe; Hana Sofia Andersson; James Ussher; Jill Sherwood; Joep de Ligt; Josh Freeman; Julia Howard; Juliet Elvy; Lauren Jelly; Mary DeAlmeida; Matt Blakiston; Matt Storey; Matthew Rogers; Max Bloomfield; Michael Addidle; Michelle Balm; Sally Roberts; Sarah Jefferies; Sharmini Muttaiyah; Susan Morpeth; Susan Taylor; Timothy Blackmore; Vani Sathyendran; Veronica Playle; Virginia Hope; Xiaoyun Ren
EPI_ISL_849739, EPI_ISL_849740, EPI_ISL_849741, EPI_ISL_849742, EPI_ISL_849743	Special Operations Medical Research Division, Defence Services Medical Research Centre	Special Operations Medical Research Division, Defence Services Medical Research Centre	Aung; Htun; K.K.; K.Z.; Lwin; Myint, K.; N.M.; Oo; P.K.; Win; Z.W.; Zaw, T.
EPI_ISL_767927	St Vincent's Pathology (SydPath)	NSW Health Pathology - Institute of Clinical Pathology and Medical Research; Westmead Hospital; University of Sydney	CIDM-PH et al.
EPI_ISL_1261778	State Hygienic Laboratory at the University of Iowa	State Hygienic Laboratory at the University of Iowa	Alankar Kampoowale; Valerie Reeb; Wes Hottel
EPI_ISL_1495125, EPI_ISL_1495140	State Institution «Public Health Center of Ministry of Health of Ukraine»	Robert Koch Institute, ZBS1 Highly Pathogenic Viruses, Berlin, Germany	Andreas Nitsche; Annika Brinkmann; Iryna Demchyshyna; Janine Michel; Liudmyla Chernenko; Roman Rodyna; Steven Uddin

EPI_ISL_752619, EPI_ISL_752633, EPI_ISL_752952, EPI_ISL_967760, EPI_ISL_1292430, EPI_ISL_1292438, EPI_ISL_1515922			
see above	State Laboratories Division, Hawaii State Department of Health	State Laboratories Division, Hawaii State Department of Health	Ayana Garnet; Drew Kuwazaki; Edward Desmond; Pamela O'Brien; Razvan Sultana; Sabrina Diemert
EPI_ISL_882954	State Veterinary Institute Prague	State Veterinary Institute Prague	A; Cernikova; L; M; Nagy; Stara
EPI_ISL_640079	Stellenbosch Hospital wc STB	NHLS/UCT	Arash Iranzadeh; Bruna Galvao; Carolyn Williamson; Deelan Doolabh; Diana Hardie; Innocent Mudau; Kruger Marais; Lynn Tyers; Marvin Hsiao; Stephen Korsman
EPI_ISL_1534311	Still Bay Sat Clinic wc SFV	NHLS/UCT	Arash Iranzadeh; Bruna Galvao; Carolyn Williamson; Deelan Doolabh; Diana Hardie; Innocent Mudau; Kruger Marais; Lynn Tyers; Marvin Hsiao; Stephen Korsman
EPI_ISL_1615590	Subang Public Health	West Java Health Laboratory; School of Life Sciences and Technology, Institut Teknologi Bandung	Aulia Saraswati Wicaksono; Azzania Fibriani; Cut Nur Cinthia Alamanda; Ema Rahmawati; Karimatu Khoirunnisa; Miftahul Faridl; Rifky Waluyajati Rachman; Rini Robiani; Ryan Bayusantika Ristandi
EPI_ISL_491087	Suceava County Emergency Hospital	"Stefan cel Mare" University Metagenomics Lab	Antoniadis Panagiotis et al.; Lobiuc Andrei
EPI_ISL_1197741, EPI_ISL_1197758, EPI_ISL_1198065, EPI_ISL_1198331, EPI_ISL_1599662, EPI_ISL_1618069, EPI_ISL_1618243, EPI_ISL_1618272			
see above	Swedish national genomic surveillance program of SARS-CoV-2	The Public Health Agency of Sweden	Swedish national genomic surveillance program of SARS-CoV-2
EPI_ISL_478717	Sydney South West Pathology Service (SSWPS) - Concord Repatriation General Hospital - NSW Health Pathology	NSW Health Pathology - Institute of Clinical Pathology and Medical Research; Westmead Hospital; University of Sydney	CIDM-PH et al.
EPI_ISL_593755, EPI_ISL_639729, EPI_ISL_767904	Sydney South West Pathology Service (SSWPS) - Liverpool Hospital - NSW Health Pathology	NSW Health Pathology - Institute of Clinical Pathology and Medical Research; Westmead Hospital; University of Sydney	CIDM-PH et al.
EPI_ISL_767858, EPI_ISL_845806, EPI_ISL_872586	Sydney South West Pathology Service (SSWPS) - Royal Prince Alfred Hospital - NSW Health Pathology	NSW Health Pathology - Institute of Clinical Pathology and Medical Research; Westmead Hospital; University of Sydney	CIDM-PH et al.
EPI_ISL_1319281, EPI_ISL_1470275	Synlab Eesti OÜ	1. Laboratory of Communicable Diseases (Estonia); 2. Eurofins Genomics Europe Sequencing GmbH	Liidia Dotsenko et al.
EPI_ISL_914797, EPI_ISL_914813	TAMIZAJE COMUNITARIO - PASO CANOAS	Inciensa, Instituto Costarricense de Investigación y Enseñanza en Nutrición y Salud	Adriana Godínez; Claudio Soto-Garita; Estela Cordero; Francisco Duarte; Hebleen Porras; Melany Calderón & Mariel López
EPI_ISL_1094615	TN Division of Laboratory Services	Respiratory Viruses Branch, Division of Viral Diseases, Centers for Disease Control and Prevention	Anna Montmayeur; Anna Uehara; Ben L. Rambo-Martin; Clinton R. Paden; Dhwani Batra; Haibin Wang; Jasmine Padilla; Jing Zhang; Justin Lee; Krista Queen; Lori Rowe; Mark Burroughs; Mili Sheth; Peter W. Cook; Rachel Marine; Sarah Nobles; Suxiang Tong; Yan Li; Ying Tao
EPI_ISL_1527566, EPI_ISL_1607948	TXDSHS	TXDSHS	Anita Pokharel; Bonnie Oh; Chun Wang; Grace Kubin; Jenny Zhang; Lorraine Rodriguez; Maliha Rahman; Mayela Pedrueza; Myong Koag; Rachel Lee; Rashmi Tuladhar
EPI_ISL_411926	Taiwan Centers for Disease Control	Taiwan Centers for Disease Control	Ji-Rong Yang; Jung-Jung Mu; Ming-Tsan-Liu; Yu-Chi-Lin
EPI_ISL_1578466	Teaching Institute for Public Health of Split-Dalmatia County	Croatian Institute of Public Health	Irena Tabain; Ivana Ferenčak
EPI_ISL_1591266	Teaching Institute for Public Health of Varaždin County	Croatian Institute of Public Health	Irena Tabain; Ivana Ferenčak
EPI_ISL_966940	Technical Support Units for Scientific Research (UATRS), National Centre for Scientific and Technical Research (CNRST)	Technical Support Units for Scientific Research (UATRS), National Centre for Scientific and Technical Research (CNRST)	Alaoui; Elalaoui; Elannaz, H.; Elouanass, M.; Ennibi; H. and El Fahime, E.; Hemlali, M.; Lahlou; M.A.; Melloul, M.; Rfaki, A.; S.A.; Touil, N.; a.I.
EPI_ISL_877227, EPI_ISL_934971, EPI_ISL_937538, EPI_ISL_1117527	Thai Red Cross Emerging Infectious Diseases Health Science Centre, Chulalongkorn Hospital, Faculty of Medicine, Chulalongkorn University	Thai Red Cross Emerging Infectious Diseases Center and Faculty of Medicine, Chulalongkorn University	Apaporn Rodpan; Chaniya Jamsaim; Duangtip Singhakun; Gompol Suwanpimolkul; Krirk Asavametha; Leilani Paitoonpong; Opass Putcharoen; Panpit Suwangool; Pattama Torvorapanit; Ratchada Kitsommart; Rome Buathong; Sininat Petcharat; Sininat Petcharat; Sopon Iamsirithaworn; Supaporn Wacharapluesadee; Thiravat Hemachudha; Watsamon Jantarabenjakul; Weenassarin Ampoot; Yutthana Joyjinda; Yutthana Joyjinda
EPI_ISL_672114	The Ashley Laboratory, Stanford University	Chan-Zuckerberg Biohub	CZB Cliahub Consortium
EPI_ISL_541333, EPI_ISL_693678, EPI_ISL_850666, EPI_ISL_850669, EPI_ISL_850675	The National Institute of Public Health	State Veterinary Institute Prague	A; D; H; J; Jirincova; L; M; Nagy; Novakova; Trinklova; Trnka; Vecerova
EPI_ISL_491118	The National Institute of Public Health	The National Institute of Public Health and State Veterinary Institute Prague	A; D; H; J; Jirincova; L; Nagy; Novakova; Trnka; Vecerova
EPI_ISL_827878, EPI_ISL_1586170, EPI_ISL_1586769	The National University Hospital of Iceland	deCODE genetics	Agnar Helgason; Alma Moller; Arna B Agustsdottir; Arnaldur Gylfason; Asgeir Sigurdsson; Aslaug Jonasdottir; Berglind Eiriksdottir; Bjarni Thorbjornsson; Brynjar O Jensson; Daniel F Gudbjartsson; Droplaug N Magnusdottir; Elisabet E Gardarsdottir; Emil A Thorarensen; Gardar Sveinbjornsson; Gisli Masson; Gudmundur Georgsson; Gudmundur L Norddahl; Gudrun Sigmundsdottir; Hakon Jonsson; Hannes Eggertsson; Hilma Holm; Ingileif Jonsdottir; Jona Saemundsdottir; Kamilla S Josefsdottir; Kari Stefansson; Karl G Kristinsson; Kjartan R Gudmundsson; Kristin E Sveinsdottir; Louise le Roux; Maney Sveinsdottir; Olafia S Gretarsdottir; Olafur T Magnusson; Pall Melsted; Patrick Sulem; Run Fridriksdottir; Solvi Rognvaldsson; Thora R Gunnarsdottir; Thordur Kristjansson; Thorolfur Gudnason; Unnur Thorsteinsdottir
EPI_ISL_889334	The University Hospital Brno	Institute of Applied Biotechnologies a.s.	Kateřina Kvapilová; Martin Kašný; Martina Lengerová; Ondřej Brzoň; Petr Klempt; Petr Kvapil
EPI_ISL_640019, EPI_ISL_700428, EPI_ISL_700431, EPI_ISL_700438	Thembalethu CDC wc THC	NHLS/UCT	Arash Iranzadeh; Bruna Galvao; Carolyn Williamson; Deelan Doolabh; Diana Hardie; Houriiyah Tegally; Innocent Mudau; Kruger Marais; Lynn Tyers; Marvin Hsiao; Stephen Korsman
EPI_ISL_690803	Tokyo Metropolitan Institute of Public Health	Pathogen Genomics Center, National Institute of Infectious Diseases	Kentaro Itokawa; Makoto Kuroda; Masanori Hashino; Rina Tanaka; Tsuyoshi Sekizuka
EPI_ISL_792061, EPI_ISL_792085, EPI_ISL_961381, EPI_ISL_961400	Toronto Invasive Bacterial Diseases Network	McMaster University	Ahmed Draia; Allison McGeer; Andrew G. McArthur; Angel Li; David Richardson; Emily Panousis; Hooman Derakhshani; Jalees Nasir; Kuganya Nirmalarajah; Michael Surette; Patryk Aftanas; Samira Mubareka
EPI_ISL_745140, EPI_ISL_745148, EPI_ISL_745182, EPI_ISL_745183, EPI_ISL_745185	Tygerberg Hospital wc TBH	National Health Laboratory Service (NHLS), Tygerberg	Bronwyn Kleinhans; Eduan Wilkindon; Gert van Zyl; Houriiyah Tegally; Kayla Delaney; Susan Engelbrecht; Tulio de Oliveira; Wolfgang Preiser
EPI_ISL_1581474	UAB InMedica	Vilnius University Hospital Santaros Klinikos, Center of Laboratory Medicine	Daniel Naumovas; Dovile Ezerskyte; Gytis Dudas; Ingrida Olendraite; Laimonas Griskevicius; Ligita Raugaite; Mindaugas Stoskus; Monika Katenaite; Rimvydas Norvilas
EPI_ISL_941870	ULS Alto Minho	Instituto Nacional de Saude (INSA) and Instituto Gulbenkian de Ciencia (IGC)	Borges et al
EPI_ISL_1116963	ULS Litoral Alentejano	Instituto Nacional de Saude (INSA) and Instituto Gulbenkian de Ciencia (IGC)	Borges et al
EPI_ISL_941891	ULSM - Matosinhos	Instituto Nacional de Saude (INSA) and Instituto Gulbenkian de Ciencia (IGC)	Borges et al
EPI_ISL_977224	ULSS 1 Dolomiti	Istituto Zooprofilattico Sperimentale delle Venezie	Adelaide Milani; Alessia Schivo; Alice Fusaro; Ambra Pastori; Annalisa Salviato; Antonia Ricci; Bianca Zecchin; Calogero Terregino; Erika Giorgia Quaranta; Isabella Monne
EPI_ISL_1559375	UMC Groningen, Clinical Virology, Department of Medical Microbiology and Infection Prevention	UMC Groningen, Clinical Virology, Department of Medical Microbiology and Infection Prevention	Alexander Friedrich; Coretta Van Leer-Buter; Erley Lizarazo-Forero; Hubert Niesters; Lilli Gard; Marjolein Knoester; Monika Fliss; Sigrid Rosema; Xuewei Zhou
EPI_ISL_1479126	UNIDADE DE ATENDIMENTO DST AIDS TB E HAN	Epiclin	Ana Paula Muterle; Carolina Comerlato; Eliana Márcia Da Ros Wendland; Fernando Hayashi Sant'Anna; Janira Prichula; Juliana Comerlato
EPI_ISL_1445121	UNIDADE DE PRONTO ATENDIMENTO UPA DRA ANA OLIVIA BENTIVOGLIO	Instituto Butantan / Mendelics	Antonio Jorge Martins; Bibiana Santos; Claudia Renata dos Santos Barros; David Schlesinger; Debora Botequio Moretti; Dimas Tadeu Covas; Elaine Cristina Marqueze; Elaine Vieira dos Santos; Erika Freitas; Evandra Strazza Rodrigues; Flavia Aburjaile; José Salvatore Leister Patané; João Paulo Kitajima; Luiz Carlos Junior de Alcantara; Maria Carolina Elias; Marta Giovanetti; Rafael dos Santos Bezerra; Raul Machado Neto; Ricardo Haddad; Rodrigo Tocantins Calado.; Sandra Coccuzzo Sampaio; Simone Kashima; Svetoslav Nanev Slavov; Vagner Fonseca; Vincent Louis Viala
EPI_ISL_1445128	UNIDADE RESPIRATORIA NOVA HORTOLANDIA	Instituto Butantan / Mendelics	Antonio Jorge Martins; Bibiana Santos; Claudia Renata dos Santos Barros; David Schlesinger; Debora Botequio Moretti; Dimas Tadeu Covas; Elaine Cristina Marqueze; Elaine Vieira dos Santos; Erika Freitas; Evandra Strazza Rodrigues; Flavia Aburjaile; José Salvatore Leister Patané; João Paulo Kitajima; Luiz Carlos Junior de Alcantara; Maria Carolina Elias; Marta Giovanetti; Rafael dos Santos Bezerra; Raul Machado Neto; Ricardo Haddad; Rodrigo Tocantins Calado.; Sandra Coccuzzo Sampaio; Simone Kashima; Svetoslav Nanev Slavov; Vagner Fonseca; Vincent Louis Viala
EPI_ISL_1399878	UNILABS	Instituto Nacional de Saude (INSA)	Borges et al
EPI_ISL_861676	UPA Vila Santa Catarina	Instituto Adolfo Lutz, Interdiciplinary Procedures Center, Strategic Laboratory	Claudia Regina Gonçalves; Claudio Tavares Sacchi; Erica Valessa Ramos Gomes; Karoline Rodrigues Campos
EPI_ISL_1137251, EPI_ISL_1373716	UPMC Clinical Microbiology Laboratory	Microbial Genome Sequencing Center; Microbial Genomic Epidemiology Laboratory, University of Pittsburgh	Daniel J. Snyder; Jane W. Marsh; Kady D. Waggle; Lee H. Harrison; Marissa P. Griffith; Stephanie L. Mitchell; Vatsala R. Srinivasa; Vaughn S. Cooper
EPI_ISL_681988, EPI_ISL_682035, EPI_ISL_682037	UPMC Clinical Microbiology Laboratory	Microbial Genomic Epidemiology Laboratory, University of Pittsburgh	Chinelo Ezeonwuku; Dan Snyder; Jane W. Marsh; Kady D. Waggle; Lee H. Harrison; Marissa P. Griffith; Mustapha M. Mustapha; Stephanie L. Mitchell; Vatsala R. Srinivasa; Vaughn S. Cooper
EPI_ISL_1499395	URMC LABS	Wadsworth Center, New York State Department of Health	Alexis Russel; Daryl M. Lamson; Erasmus Schneider; Erica Lasek-Nesselquist; John Kelly; Jonathan Plitnick; Kirsten St. George; Matthew Shudt; Melissa A Leisner; Navjot Singh

EPI_ISL_1531477	US Air Force School of Aerospace Medicine	US Air Force School of Aerospace Medicine	Amanda Javorina; Anthony Fries; Clarise Starr; Elizabeth Macias; Jennifer Meyer; Sarah Purves; William Buggele; William Gruner
EPI_ISL_427209, EPI_ISL_461417, EPI_ISL_824881, EPI_ISL_1324121, EPI_ISL_1324122, EPI_ISL_1324123, EPI_ISL_1324124, EPI_ISL_1324126, EPI_ISL_1324127, EPI_ISL_1324128, EPI_ISL_1324129, EPI_ISL_1324130, EPI_ISL_1324131			
see above	UW Virology Lab	UW Virology Lab	Alexander Greninger; Amin Addetia; Hong Xie; Keith Jerome; Keith R Jerome; Lasata Shrestha; Margaret Mills; Meei-Li Huang; Michelle Lin; Noah Baker; Pavitra Roychoudhury; Saraswathi Sathees; Sean Ellis; Shah Mohamed Bakhash; Truong Nguyen
EPI_ISL_734725, EPI_ISL_734818	UZ Leuven, National Reference Laboratory for Coronaviruses, Laboratory Medicine, Leuven, Belgium	KU Leuven, Rega Institute, Clinical and Epidemiological Virology	Bert Vanmechelen; Joan Marti-Carerras; Piet Maes; Tony Wawina-Bokalanga
EPI_ISL_737935, EPI_ISL_737939, EPI_ISL_737940, EPI_ISL_737941, EPI_ISL_737942, EPI_ISL_737945, EPI_ISL_737946, EPI_ISL_737947, EPI_ISL_737949, EPI_ISL_737950, EPI_ISL_737951, EPI_ISL_737952, EPI_ISL_737953, EPI_ISL_737954, EPI_ISL_737955, EPI_ISL_737956, EPI_ISL_737957, EPI_ISL_737958, EPI_ISL_737959, EPI_ISL_737960, EPI_ISL_737961, EPI_ISL_737963, EPI_ISL_737965, EPI_ISL_737970, EPI_ISL_737971, EPI_ISL_737972, EPI_ISL_737973, EPI_ISL_737974, EPI_ISL_737975, EPI_ISL_737976, EPI_ISL_737977, EPI_ISL_737978, EPI_ISL_737980, EPI_ISL_737981, EPI_ISL_737982, EPI_ISL_737983, EPI_ISL_737985, EPI_ISL_737987, EPI_ISL_737988, EPI_ISL_737990, EPI_ISL_737991, EPI_ISL_737992, EPI_ISL_737993, EPI_ISL_737995, EPI_ISL_737997, EPI_ISL_737998, EPI_ISL_737999, EPI_ISL_738000, EPI_ISL_738001, EPI_ISL_738002, EPI_ISL_738005, EPI_ISL_738006, EPI_ISL_738007, EPI_ISL_738008, EPI_ISL_738009, EPI_ISL_738010, EPI_ISL_738011, EPI_ISL_738012, EPI_ISL_738013, EPI_ISL_738014, EPI_ISL_738015, EPI_ISL_738016, EPI_ISL_738017, EPI_ISL_738019, EPI_ISL_738020, EPI_ISL_738021, EPI_ISL_738022, EPI_ISL_738023, EPI_ISL_738026, EPI_ISL_738038			
see above	Uganda Central Public Health Lab and Uganda Virus Research Institute	MRC/UVRI & LSHTM Uganda Research Unit	Dan Lule Bugembe; Matthew Cotten; My V.T. Phan; Pontiano Kaleebu et al.
EPI_ISL_451183, EPI_ISL_451184, EPI_ISL_451186, EPI_ISL_451189, EPI_ISL_451190, EPI_ISL_451193, EPI_ISL_451195, EPI_ISL_451198, EPI_ISL_451199, EPI_ISL_451201, EPI_ISL_451202			
see above	Uganda Virus Research Institute	MRC/UVRI & LSHTM Uganda Research Unit	Beatrice Dhaala; Dan Lule Bugembe; Deogratius Ssemwanga; Henry Kyobe; Henry Mwebesa; Jane Aceng; John Kayiwa; Jonas Lexow; Julius Lutwama; Matthew Cotten; My V.T Phan; Phionah Tushabe; Pontiano Kaleebu; Stephen Balinandi
EPI_ISL_1351494	Unidad de InvestigaciÃ³n MÃ©dica de YucatÃ¡n (UIMY)	Unidad de Genomica Avanzada	Alejandro Sanchez-Flores; Alfredo Herrera-Estrella; Alicia Ocana-Mondragon; Angel Gustavo Salas-Lais; Bernardo Martinez-Miguel; Blanca Taboada; Brenda Irasema Maldonado-Meza; Carla Ivon Herrera-Najera; Carlos F. Arias; Celia Boukadida; Clara Esperanza Santacruz-Tinoco; Concepcion Grajales-Muniz; Consorcio Mexicano de Vigilancia Genomica (CoViGen-Mex). Authors (in alphabetical order): Julio Elias Alvarado-Yaah; Fernando Fontove-Herrera; Francisco Pulido; Gloria Elena Espinoza-Ayala; Gloria Maria Molina-Salinas; Gloria Vazquez; Hector Esteban Paz-Juarez; Hector Montoya-Fuentes; Helen Haydee Fernanda Ramirez-Plascencia; Jorge Ivan Salinal-Nevarez; Jose Antonio Enciso-Moreno; Jose Esteban Munoz-Medina; Jose de Jesus Nunez-Contreras; Juan Bautista Chale-Dzul; Luis Alberto Ochoa-Carrera; Margarita Matias-Florentino; Maria Guadalupe Santiago-Mauricio; Maria Guadalupe de Jesus Mireles-Rivera; Nelly Selem-Mojica; Pavel Isa; Ricardo Grande; Santiago Avila-Rios; Victor Hugo Borja-Aburto
EPI_ISL_1279322	Unidad de Investigación Biomédica de Zacatecas (UIBZ)	Instituto Nacional de Enfermedades Respiratorias (INER): Centro de Investigación en Enfermedades Infecciosas (CIENI)	Alejandro Sanchez-Flores; Alfredo Herrera-Estrella; Alicia Ocaña-Mondragón; Angel Gustavo Salas-Lais; Bernardo Martínez-Miguel; Blanca Taboada; Brenda Irasema Maldonado-Meza; Carla Ivón Herrera-Najera; Carlos F. Arias; Celia Boukadida; Clara Esperanza Santacruz-Tinoco; Concepción Grajales-Muñiz; Consorcio Mexicano de Vigilancia Genómica (CoViGen-Mex). Authors (in alphabetical order): Julio Elias Alvarado-Yaah; Fernando Fontove-Herrera; Francisco Pulido; Gloria Elena Espinoza-Ayala; Gloria María Molina-Salinas; Gloria Vazquez; Hector Esteban Paz-Juárez; Hector Montoya-Fuentes; Helen Haydee Fernanda Ramírez-Plascencia; Jorge Ivan Salinal-Nevarez; José Antonio Enciso-Moreno; José Esteban Muñoz-Medina; José de Jesús Nuñez-Contreras; Juan Bautista Chale-Dzul; Luis Alberto Ochoa-Carrera; Margarita Matías-Florentino; María Guadalupe Santiago-Mauricio; María Guadalupe de Jesús Mireles-Rivera; Nelly Sélem-Mojica; Pavel Isa; Ricardo Grande; Santiago Ávila-Ríos; Víctor Hugo Borja-Aburto
EPI_ISL_1299856, EPI_ISL_1299860, EPI_ISL_1299861, EPI_ISL_1299866, EPI_ISL_1299880	Unit of lab surveillance of viral emerging diseases, National Lab of Influenza	Respiratory Virus Unit, National Infection Service, Public Health England	Iris Hasibra; PHE Covid Sequencing Team; Prof Albana Fico; Prof Silvia Bino
EPI_ISL_812310, EPI_ISL_812555, EPI_ISL_812569, EPI_ISL_812589, EPI_ISL_831760, EPI_ISL_831839	United States Air Force School of Aerospace Medicine	United States Air Force School of Aerospace Medicine	Amanda Javorina; Anthony Fries; Clarise Starr; Elizabeth Macias; Jennifer Meyer; Sarah Purves; William Gruner
EPI_ISL_1490686, EPI_ISL_1490689	Unity Health Toronto	Ontario Institute for Cancer Research	Bernard Lam; Felicia Vincelli; Ilinca Lungu; Jared T. Simpson; Jeremy Johns; Karel Boissinot; Larissa M. Matukas; Le Luu; Mark Downing; Paul Krzyzanowski; Philip Zuzarte; Ramzi Fattouh; Richard de Borja; Samira Mubareka; TIBDN; Trina Otterman; Yan Chen
EPI_ISL_1540707	Universidad Tecnica Particular de Loja	Institute of Microbiology, Universidad San Francisco de Quito	Belén Prado-Vivar; Bernardo Gutiérrez; David Zuñiga; Fernando Serrano; Gabriel Trueba; Juan José Guadalupe; Katherine Ojeda; Luis Flores; Melissa Ortega; Michelle Grunauer; Monica Becerra-Wong; Paola Dalgo; Patricio Rojas-Silva; Paúl Cárdenas; Raiza Briceño; Sully Márquez; Verónica Barragán
EPI_ISL_1020131	University Clinic of Nephrology- Skopje	Research Center for Genetic Engineering and Biotechnology "Georgi D. Efremov" , Macedonian Academy of Sciences and Arts	Aleksandar J. Dimovski; Dijana Plasheska-Karanfilska; Gjorgji Bozinovski; Milena Jakimovska; Predrag Noveski
EPI_ISL_812966, EPI_ISL_812967	University Clinical Research Center, University of Sciences	University Clinical Research Center, University of Sciences	A.A.; Bane, S.; Dao, S.; Diakite, M.; Diarra, B.; Doumbia, S.; Guindo, I.; Iknane; Kone, A.
EPI_ISL_1272020, EPI_ISL_1272024	University Hospital Centre Zagreb	Croatian Institute of Public Health	Irena Tabain; Ivana Ferenčak
EPI_ISL_710548, EPI_ISL_710555	University Hospital Dubrava	Ruđer Boškovic Institute; Forensic Science Centre Ivan Vučetić; University of Zagreb Faculty of Science	Ana Livun; Antonela Blažeković; Boris Maček; Danilo Licastro; Dunja Glavaš; Fran Borovečki; Fuad Ćosović; Gordana Maravić Vlahoviček; Ivan Šamija; Ivana Ćelap; Jasna Kašman; Josipa Skelin; Katarina Marija Tupek; Kristian Vlahoviček; Kristina Gotovac Jereić; Lidija Cvetko-Krajinović; Lucija Basić; Lucija Markulin; Maja Kuzman; Marina Korolija; Mario Štefanović; Mirjana Domazet-Lošo; Paula Štancl; Petra Vrabec; Robert Belužić; Rosa Karlić; Sanja Tadinac; Senčica Pejša; Tomislav Domazet-Lošo; Valentina Đumlijan-Combaj; Vjekoslav Tomaić; Vladimir Krajinović; Željka Mačak Šafranko
EPI_ISL_1166683	University Hospitals of Geneva, Laboratory of Virology	HUG, Laboratory of Virology and the Health2030 Genome Center	Ana Rita Goncalves; Deborah Penet; Emmanouil Dermitzakis; Henri Pegeot; Ioannis Xenarios; Keith Harshman; Laurent Kaiser; Lorenzo Cerutti; Melyssa Elies; Samuel Cordey
EPI_ISL_671428, EPI_ISL_671475	University of Debrecen, Department of Medical Microbiology	National Laboratory of Virology, Szentágothai Research Centre	Balázs Somogyi; Brigitta Zana; Endre Gábor Tóth; Eszter Csoma; Ferenc Jakab; Gábor Kemenesi
EPI_ISL_1040497	University of Liège COVID-19 testing center	GIGA Medical Genomics	Bouchra Boujemla; Cécile Meex; Fabrice Bureau; Keith Durkin; Laurent Gillet; Maria Artesi; Marie-Pierre Hayette; Nathalie Renotte; Sébastien Bontems; Vincent Bours; Wouter Coppieters
EPI_ISL_955150, EPI_ISL_955156, EPI_ISL_955169, EPI_ISL_955171, EPI_ISL_955173, EPI_ISL_955185, EPI_ISL_955188, EPI_ISL_955210, EPI_ISL_1016508, EPI_ISL_1016690, EPI_ISL_1016968			
see above	University of Sarajevo, Veterinary Faculty, Laboratory for Molecular Diagnostic and Research Laboratory	University of Sarajevo, Veterinary Faculty, Laboratory for Molecular Diagnostic and Research Laboratory	Alic-Seho A.; Alic-Šeho A.; Alić-Šeho A.; Goletic S.; Goletic T.; Goletic Š.; Goletić T.; Goletić Š.; Hodzic A.; Hodžic A.; Hodžić A.; Jazic A.; Jažic A.; Jažić A.; Nicevic M.; Nicević M.; Sabic E.; Softic A.; Softić A.; Terzic I.; Terzić I.; Šabic E.; Šabić E.
EPI_ISL_477618	University of Szeged, Institute of Clinical Microbiology	National Laboratory of Virology, Szentágothai Research Centre	Balázs Somogyi; Brigitta Zana; Endre Gábor Tóth; Ferenc Jakab; Gábor Kemenesi; Terhes Gabriella
EPI_ISL_428255, EPI_ISL_516514, EPI_ISL_605499, EPI_ISL_759733	University of Wisconsin-Madison AIDS Vaccine Research Laboratories	University of Wisconsin-Madison AIDS Vaccine Research Laboratories	Gage Moreno; Katarina Braun; et al. AIDS Vaccine Research Laboratories
EPI_ISL_977293, EPI_ISL_977294, EPI_ISL_977312, EPI_ISL_977313, EPI_ISL_977314, EPI_ISL_977316, EPI_ISL_977317, EPI_ISL_977318, EPI_ISL_977319, EPI_ISL_977320, EPI_ISL_977321, EPI_ISL_977322, EPI_ISL_977323, EPI_ISL_977324, EPI_ISL_977325, EPI_ISL_977326, EPI_ISL_977327, EPI_ISL_977328, EPI_ISL_977329, EPI_ISL_977330, EPI_ISL_977331, EPI_ISL_977332, EPI_ISL_977334, EPI_ISL_977335, EPI_ISL_977336, EPI_ISL_977337, EPI_ISL_977338, EPI_ISL_977339, EPI_ISL_977340, EPI_ISL_977341, EPI_ISL_977342, EPI_ISL_977343, EPI_ISL_977344, EPI_ISL_977345, EPI_ISL_977346, EPI_ISL_977347, EPI_ISL_977349, EPI_ISL_977350, EPI_ISL_977351, EPI_ISL_977353, EPI_ISL_977354, EPI_ISL_977355, EPI_ISL_977357, EPI_ISL_977359, EPI_ISL_977360, EPI_ISL_977361, EPI_ISL_977362, EPI_ISL_977363, EPI_ISL_977364, EPI_ISL_977365, EPI_ISL_977366, EPI_ISL_977367, EPI_ISL_977368, EPI_ISL_977370, EPI_ISL_977371, EPI_ISL_977372, EPI_ISL_977373, EPI_ISL_977374, EPI_ISL_977375, EPI_ISL_977376, EPI_ISL_977377, EPI_ISL_977378, EPI_ISL_977379, EPI_ISL_977380, EPI_ISL_977381, EPI_ISL_977382, EPI_ISL_977383, EPI_ISL_977384, EPI_ISL_977386, EPI_ISL_977387, EPI_ISL_977388, EPI_ISL_977389, EPI_ISL_977390, EPI_ISL_977391, EPI_ISL_977392, EPI_ISL_977394, EPI_ISL_977395, EPI_ISL_977396, EPI_ISL_977397, EPI_ISL_977399, EPI_ISL_977400, EPI_ISL_977401, EPI_ISL_977402, EPI_ISL_977403, EPI_ISL_977404, EPI_ISL_977405, EPI_ISL_977406, EPI_ISL_977407, EPI_ISL_977408, EPI_ISL_977409, EPI_ISL_977410, EPI_ISL_977411, EPI_ISL_977412, EPI_ISL_977413, EPI_ISL_977414, EPI_ISL_977415, EPI_ISL_977416, EPI_ISL_977418, EPI_ISL_977419, EPI_ISL_977420, EPI_ISL_977421, EPI_ISL_977423, EPI_ISL_977424, EPI_ISL_977425, EPI_ISL_977426, EPI_ISL_977427, EPI_ISL_977428, EPI_ISL_977429, EPI_ISL_977430, EPI_ISL_977431, EPI_ISL_977432, EPI_ISL_977433, EPI_ISL_977434, EPI_ISL_977435, EPI_ISL_977437, EPI_ISL_977438, EPI_ISL_977439, EPI_ISL_977440, EPI_ISL_977441, EPI_ISL_977442, EPI_ISL_977443, EPI_ISL_977444, EPI_ISL_977445, EPI_ISL_977446, EPI_ISL_977448, EPI_ISL_977449, EPI_ISL_977452, EPI_ISL_977455, EPI_ISL_977456, EPI_ISL_977458, EPI_ISL_977460, EPI_ISL_977461, EPI_ISL_977462, EPI_ISL_977463, EPI_ISL_977465, EPI_ISL_977466, EPI_ISL_977469, EPI_ISL_977470			
see above	University of Zambia, School of Veterinary Medicine	UNZAVET and PATH	Daniel Bridges; Mulenga Mwenda-Chimfwembe; Ngonda Saasa
EPI_ISL_931569	Utah Public Health Laboratory	Utah Public Health Laboratory	Erin L. Young; Kelly F. Oakeson; Tara Gallagher
EPI_ISL_940862, EPI_ISL_940877, EPI_ISL_940880, EPI_ISL_940883, EPI_ISL_940886, EPI_ISL_940889, EPI_ISL_940890, EPI_ISL_940891			
see above	Vaccines and Infectious Diseases Analytics Research Unit (VIDA)	KRISP, KZN Research Innovation and Sequencing Platform	Baillie Vicky; Giandhari Jennifer; Madhi Shabir; Naidoo Yeshnee; Pillay Sureshnee; Tegally Houriiyah; de Oliveira Tulio; du Plessis Jeanine
EPI_ISL_1132671, EPI_ISL_1132672, EPI_ISL_1132720, EPI_ISL_1132721, EPI_ISL_1132728	Vaccines and Infectious Diseases Analytics Research Unit (VIDA)	KRISP, KZn Research Innovation and Sequencing Platform	Baillie Vicky; Giandhari Jennifer; Madhi Shabir; Naidoo Yeshnee; Pillay Sureshnee; Tegally Houriiyah; de Oliveira Tulio; du Plessis Jeanine
EPI_ISL_708820	Vajira Hospital	National Institute of Health, Department of Medical Sciences, Ministry of Public Health, Thailand	Malinee Chittaganpitch; Pakorn Piromtong; Pilailuk Okada; Siripaporn Phuygun; Sittiporn Parnmen; Sunthareeya Waicharoen; Thanutsapa Thanadachakul; Warawan Wongboot
EPI_ISL_1373903	Vanderbilt University Medical Center	Pathogen Discovery, Respiratory Viruses Branch, Division of Viral Diseases, Centers for Disease Control and Prevention	Anna Kelleher; Anna Uehara; Brian Lynch; Clinton R. Paden; Haibin Wang; Han Jia Justin Ng; Jing Zhang; Krista Queen; Peter Cook; Suxiang Tong; Yan Li; Ying Tao
EPI_ISL_960159	Vanguard CHC wc VGC	National Health Laboratory Service/UCT	Arash Iranzadeh; Bruna Galvao; Carolyn Williamson; Deelan Doolabh; Diana Hardie; Innocent Mudau; Kruger Marais; Lynn Tyers; Marvin Hsiao; Stephen Korsman
EPI_ISL_904010, EPI_ISL_904011	Veterinary Specialized Institute Kraljevo	Veterinary Specialized Institute "Kraljevo", Serbia	Afonso, C.; Banovic Djeri, B.; Jankovic, M.; Jovanovic, T.; Knezevic, A.; Petrovic, T.; Sekler, M.; Tesovic, B.; Vidanovic, D.; Volkening, J.
EPI_ISL_833514	Veterinary Specialized Instute "Nis"	Veterinary Specialized Institute "Kraljevo", Serbia	Afonso, C.; Banovic Djeri, B.; Jankovic, M.; Jovanovic, T.; Knezevic, A.; Manic, M.; Petrovic, M.; Petrovic, T.; Sekler, M.; Tesovic, B.; Vidanovic, D.; Volkening, J.
EPI_ISL_640108, EPI_ISL_640113, EPI_ISL_700421, EPI_ISL_700546, EPI_ISL_1040774, EPI_ISL_1040784, EPI_ISL_1040814			
see above	Victoria Hospital wc VHW	NHLS/UCT	Arash Iranzadeh; Bruna Galvao; Carolyn Williamson; Deelan Doolabh; Diana Hardie; Innocent Mudau; Kruger Marais; Lynn Tyers; Marvin Hsiao; Stephen Korsman
EPI_ISL_521880, EPI_ISL_640588, EPI_ISL_779632, EPI_ISL_812441, EPI_ISL_854752, EPI_ISL_979362, EPI_ISL_1033155			
see above	Victorian Infectious Diseases Reference Laboratory (VIDRL)	VIDRL and MDU-PHL	Caly L.; Druce J.; M.L.; N.L.; Sait; Sait, M.; Schultz M.; Schultz M.B.; Seemann T.; Sherry; Sherry, N.
EPI_ISL_416415	Victorian Infectious Diseases Reference Laboratory (VIDRL)	Victorian Infectious Diseases Reference Laboratory and Microbiological Diagnostic Unit Public Health Laboratory, Doherty Institute	Caly L.; Druce J.; Schultz M.; Seemann T.; Taiaroa, G.
EPI_ISL_1585807	Vilnius University Hospital Santaros Klinikos	Vilnius University Hospital Santaros Klinikos, Center of Laboratory	Daniel Naumovas; Dovile Ezerskyte; Gytis Dudas; Ingrida Olendraite; Laimonas Griskevicius; Ligita Raugaite; Mindaugas Stoskus; Monika Katenaite; Rimvydas Norvilas

EPI_ISL_451708, EPI_ISL_500901, EPI_ISL_535631, EPI_ISL_541532, EPI_ISL_1003993, EPI_ISL_1004928, EPI_ISL_1408371, EPI_ISL_1598655			
see above	Viollier AG	Department of Biosystems Science and Engineering, ETH Zürich	Andrea Patrignani; Andreia Cabral de Gouvea; Catharine Aquino; Chaoran Chen; Christian Beisel; Christiane Beckmann; Christoph Noppen; David Dreifuss; Deborah Penet; Doris Popovic; Elodie Burcklen; Emmanouil Dermitzakis; Griffin White; Henri Pegeot; Ina Nissen; Ioannis Xenarios; Ivan Topolsky; Jay Tracy; Katharina Jahn; Keith Harshman; Lara Fuhrmann; Laura Neff; Lennart Opitz; Lorenzo Cerutti; Maria Domenica Moccia; Maurice Redondo; Natascha Santacroce; Niko Beerenwinkel; Noemie Santamaria de Souza; Olivier Kobel; Pedro Ferreira; Philipp Jablonski; Ralph Schlapbach; Rebecca Denes; Sarah Nadeau; Simon Grüter; Sophie Seidel; Susana Posada-Céspedes; Tanja Stadler; Timothy Sykes; Tobias Schär
EPI_ISL_1388419	Viollier AG	University Hospital Basel, Clinical Bacteriology	Adrian Egli; Alfredo Mari; Hans Hirsch; Helena MB Seth-Smith; Julia Bielicki; Karoline Leuzinger; Lukas Fenner; Madlen Stange; Manuel Battegay; Tim Roloff
EPI_ISL_417437, EPI_ISL_417942, EPI_ISL_417946, EPI_ISL_417947, EPI_ISL_417948, EPI_ISL_417950, EPI_ISL_420030, EPI_ISL_420032, EPI_ISL_420033, EPI_ISL_420845, EPI_ISL_420847, EPI_ISL_435032, EPI_ISL_437337, EPI_ISL_437338, EPI_ISL_437339, EPI_ISL_437340, EPI_ISL_437343, EPI_ISL_437346, EPI_ISL_437348, EPI_ISL_437350, EPI_ISL_437352, EPI_ISL_447231, EPI_ISL_447232, EPI_ISL_471396, EPI_ISL_471397, EPI_ISL_471398, EPI_ISL_471400, EPI_ISL_471402, EPI_ISL_471403, EPI_ISL_471407, EPI_ISL_471408, EPI_ISL_471409, EPI_ISL_471410, EPI_ISL_471414, EPI_ISL_471415, EPI_ISL_513598, EPI_ISL_513600, EPI_ISL_513601, EPI_ISL_513605, EPI_ISL_513606, EPI_ISL_513607, EPI_ISL_513608, EPI_ISL_513610, EPI_ISL_513617, EPI_ISL_513618, EPI_ISL_513630, EPI_ISL_591087, EPI_ISL_961013			
see above	Viral Respiratory Lab, National Institute for Biomedical Research (INRB)	Pathogen Sequencing Lab, National Institute for Biomedical Research (INRB)	Adrienne Amuri Aziza; Allison Black; Amuri Aziza; Andrew Rambaut; Catherine Pratt; Eddy Kinganda-Lusamaki; Edith Nkwembe; Emmanuel Lokilo Lofiko; Francisca Muyembe Mawete; Ian Goodfellow; James Hadfield; Jean Claude Makangara; Jean-Claude Makangara Cigolo; Jean-Jacques Muyembe Tamfum; Josh Quick; Kristian Andersen; Matthias Pauthner; Michael Wiley; Nick Loman; Placide Mbala-Kingebeni; Steve Ahuka-Mundeke; Trevor Bedford
EPI_ISL_419260, EPI_ISL_572249, EPI_ISL_942402	Virginia DCLS	Virginia DCLS	Virginia DCLS
EPI_ISL_1632700	Virginia Division of Consolidated Laboratory Services	Virginia Division of Consolidated Laboratory Services	Virginia DCLS
EPI_ISL_636982, EPI_ISL_636983, EPI_ISL_636984, EPI_ISL_636985, EPI_ISL_636986, EPI_ISL_636988	Virology Lab, National Institute for Biomedical Research (INRB)	Project group Epidemology of Highly Pathogenic Microorganisms, Robert Koch-Institute	Eddy Kinganda-Lusamaki; Essia Belarbi; Fabian Leendertz; Gabriel Mbunsu; Grit Schubert; Jasmin Schlotterbeck; Jean-Jacques Muyembe Tamfum; Sheila Makiala; Steve Ahuka-Mundeke
EPI_ISL_508862, EPI_ISL_677634, EPI_ISL_677635, EPI_ISL_677636, EPI_ISL_1660226, EPI_ISL_1660228, EPI_ISL_1660230, EPI_ISL_1660233, EPI_ISL_1660236, EPI_ISL_1660239, EPI_ISL_1660241, EPI_ISL_1660246, EPI_ISL_1660247, EPI_ISL_1660248, EPI_ISL_1660249, EPI_ISL_1660250, EPI_ISL_1660251, EPI_ISL_1660252, EPI_ISL_1660254, EPI_ISL_1660255, EPI_ISL_1660257, EPI_ISL_1660259, EPI_ISL_1660260, EPI_ISL_1660261, EPI_ISL_1660262, EPI_ISL_1660265, EPI_ISL_1660266, EPI_ISL_1660267, EPI_ISL_1660276, EPI_ISL_1660277, EPI_ISL_1660281, EPI_ISL_1660289, EPI_ISL_1660292, EPI_ISL_1660293, EPI_ISL_1660294, EPI_ISL_1660295, EPI_ISL_1660296, EPI_ISL_1660297, EPI_ISL_1660298, EPI_ISL_1660299, EPI_ISL_1660300, EPI_ISL_1660301, EPI_ISL_1660302, EPI_ISL_1660306, EPI_ISL_1660307, EPI_ISL_1660309, EPI_ISL_1660310, EPI_ISL_1660311, EPI_ISL_1660314, EPI_ISL_1660316, EPI_ISL_1660317, EPI_ISL_1660319, EPI_ISL_1660320, EPI_ISL_1660321, EPI_ISL_1660322, EPI_ISL_1660323, EPI_ISL_1660324, EPI_ISL_1660327, EPI_ISL_1660329, EPI_ISL_1660330, EPI_ISL_1660331, EPI_ISL_1660332, EPI_ISL_1660335			
see above	Virology Unit, Institut Pasteur de Madagascar	Virology Unit, Institut Pasteur de Madagascar	Angela Brisebarre; Camille Capel; Cara Brook; Cara E. Brook; Christian Ranaivoson; Christophe Malabat; Corinne Maufrais; Cristina M. Tato; Emmanuelle Permal; Etienne Simon-Lorière; Frédéric Lemoine; Helisoa Razafimanjato; Jean-Michel Heraud; Joseph L. DeRisi; Louise Lefrançois; Marion Barbet; Maud Vanpeene; Michelle Tan; Méline Bizard; Norosoa Razanajatovo; Philippe Dussart; Soa Fy Andriamandimby; Sylvie Behillil; Sylvie van der Werf; Tsiry Randriambolamanantsoa; Vida Ahyong; Vincent Enouf; Vololoniaina Raharinosy
EPI_ISL_918372, EPI_ISL_933781, EPI_ISL_933783, EPI_ISL_1098606, EPI_ISL_1532804, EPI_ISL_1532810, EPI_ISL_1532811, EPI_ISL_1532815, EPI_ISL_1534528, EPI_ISL_1534530, EPI_ISL_1534531, EPI_ISL_1534537, EPI_ISL_1534538			
see above	Virology Unit, Institut Pasteur du Cambodge	Virology Unit, Institut Pasteur du Cambodge	Chau Darapheak; Chin Savuth; Erik A Karlsson; Etienne Simon-Loriere; Kraing Sidonn; Leakhena Pum; Ly Sovann; Sokhoun Yann; Teyputita Ou; Veasna Duong; Yi Sengdoeurn
EPI_ISL_722201	Vitalis Mostar	Alea Genetic Center	Konjhodzic R.; Pecar D.; Salihefendic L.
EPI_ISL_745189	Vredenburg Hospital wc VBG	National Health Laboratory Service (NHLS), Tygerberg	Bronwyn Kleinhans; Eduan Wilkindon; Gert van Zyl; Houriiyah Tegally; Kayla Delaney; Susan Engelbrecht; Tulio de Oliveira; Wolfgang Preiser
EPI_ISL_1372296	WHO National Influenza Centre Russian Federation	WHO National Influenza Centre Russian Federation	Andrey Komissarov; Anna Ivanova; Artem Fadeev; Daria Danilenko; Dmitry Bazhenov; Dmitry Lioznov; Elena Nabieva; Georgii Bazykin; Ksenia Safina; Kseniya Komissarova; Maria Pisareva; Maria Timofeeva; Tamila Musaeva; Veronika Eder
EPI_ISL_860804	WHO/Minsk	Charité Universitätsmedizin Berlin, Institut für Virologie	Barbara Mühlemann; Christian Drosten; Julia Schneider; Julia Tesch; Jörn Beheim-Schwarzbach; Shmialiova Natallia; Sivets Natallia; Talitha Veith; Terry Jones; Tobias Bleicker; Victor M Corman
EPI_ISL_1659566	WSSE Gorzów Wlkp.	1. Academic Center for Pathomorphological and Genetic-Molecular Diagnostics ltd, Bialystok, Poland 2. National Institute of Public Health - National Institute of Hygiene, Warsaw, Poland	Anetta Sulewska; Jacek Nikliński; Janusz Dzięcioł; Joanna Kiśluk; Katarzyna Zacharczuk; Konrad Raczkowski; Magdalena Nowakowska; Małgorzata Sadkowska-Todys; Piotr Karabowicz; Piotr Majewski; Przemysław Biecek. Joanna Reszeć; Radosław Charkiewicz; Tomasz Wołkowicz
EPI_ISL_1239639	WSSE Olsztyn	1. National Institute of Public Health - National Institute of Hygiene; 2. Eurofins Genomics Europe Sequencing GmbH	ECDC COVID-19 WGS support team; Eurofins Genomics Europe Sequencing Team; Gierczyńki Rafał; Sadkowska-Todys Małgorzata; Wołkowicz Tomasz; Zacharczuk Katarzyna
EPI_ISL_1651917	WSSE w Gdańsku	1. Academic Center for Pathomorphological and Genetic-Molecular Diagnostics ltd, Bialystok, Poland 2. National Institute of Public Health - National Institute of Hygiene, Warsaw, Poland	Anetta Sulewska; Jacek Nikliński; Janusz Dzięcioł; Joanna Kiśluk; Katarzyna Zacharczuk; Konrad Raczkowski; Magdalena Nowakowska; Małgorzata Sadkowska-Todys; Piotr Karabowicz; Piotr Majewski; Przemysław Biecek. Joanna Reszeć; Radosław Charkiewicz; Tomasz Wołkowicz
EPI_ISL_903786	WVDHHR - Office of Laboratory Services	Genomics and Discovery, Respiratory Viruses Branch, Division of Viral Diseases, Centers for Disease Control and Prevention	Anna Montmayeur; Anna Uehara; Ben L. Rambo-Martin; Clinton R. Paden; Dhwani Batra; Haibin Wang; Jasmine Padilla; Jing Zhang; Justin Lee; Krista Queen; Lori Rowe; Mark Burroughs; Mili Sheth; Peter W. Cook; Rachel Marine; Sarah Nobles; Suxiang Tong; Yan Li; Ying Tao
EPI_ISL_734403	Wadsworth Center, New York State Department.of Health	Wadsworth Center, New York State Department.of Health	Alexis Russel; Daryl M. Lamson; Erasmus Schneider; Erica Lasek-Nesselquist; John Kelly; Jonathan Plitnick; Kirsten St. George; Navjot Singh; Sara Griesemer
EPI_ISL_548064	Waikato Hospital	Institute of Environmental Science and Research (ESR)	Anja Werno; Antje van der Linden; Arlo Upton; Chris Mansell; David Hammer; Dragana Drinkovic; Erasmus Smit; Gary McAuliffe; Hana Sofia Andersson; Hermes Perez; James Ussher; Jill Sherwood; Jing Wang; Joep de Ligt; Josh Freeman; Julia Howard; Juliet Elvy; Lauren Jelly; Mary DeAlmeida; Matt Blakiston; Matt Storey; Matthew Rogers; Max Bloomfield; Michael Addidle; Michelle Balm; Muhammad Faisal; Nikki Freed; Olin Silander; Sally Roberts; Sarah Jefferies; Sharmini Muttaiyah; Susan Morpeth; Susan Taylor; Timothy Blackmore; Vani Sathyendran; Veronica Playle; Virginia Hope; Xiaoyun Ren
EPI_ISL_456170, EPI_ISL_456185	Wellington SCL	Institute of Environmental Science and Research (ESR)	Anja Werno; Antje van der Linden; Arlo Upton; Chris Mansell; David Hammer; Dragana Drinkovic; Erasmus Smit; Gary McAuliffe; Hana Sofia Andersson; James Ussher; Jill Sherwood; Joep de Ligt; Josh Freeman; Julia Howard; Juliet Elvy; Lauren Jelly; Mary DeAlmeida; Matt Blakiston; Matt Storey; Matthew Rogers; Max Bloomfield; Michael Addidle; Michelle Balm; Sally Roberts; Sarah Jefferies; Sharmini Muttaiyah; Susan Morpeth; Susan Taylor; Timothy Blackmore; Vani Sathyendran; Veronica Playle; Virginia Hope; Xiaoyun Ren
EPI_ISL_637085, EPI_ISL_649125, EPI_ISL_1016878	Wellington SCL (WN)	Institute of Environmental Science and Research (ESR)	Anja Werno; Antje van der Linden; Arlo Upton; Chris Mansell; David Hammer; Dragana Drinkovic; Erasmus Smit; Gary McAuliffe; Hana Sofia Andersson; Hermes Perez; James Ussher; Jill Sherwood; Jing Wang; Joep de Ligt; Josh Freeman; Julia Howard; Juliet Elvy; Lauren Jelly; Mary DeAlmeida; Matt Blakiston; Matt Storey; Matthew Rogers; Max Bloomfield; Michael Addidle; Michelle Balm; Muhammad Faisal; Nikki Freed; Olin Silander; Sally Roberts; Sarah Jefferies; Sharmini Muttaiyah; Susan Morpeth; Susan Taylor; Timothy Blackmore; Vani Sathyendran; Veronica Playle; Virginia Hope; Xiaoyun Ren
EPI_ISL_1255106, EPI_ISL_1255107, EPI_ISL_1255109, EPI_ISL_1255110, EPI_ISL_1255111, EPI_ISL_1255115, EPI_ISL_1255116, EPI_ISL_1255117, EPI_ISL_1255119, EPI_ISL_1255120, EPI_ISL_1255121, EPI_ISL_1255122, EPI_ISL_1255123, EPI_ISL_1255124, EPI_ISL_1255125, EPI_ISL_1255126, EPI_ISL_1255127, EPI_ISL_1255128, EPI_ISL_1255129, EPI_ISL_1255131, EPI_ISL_1255132, EPI_ISL_1255133, EPI_ISL_1255134, EPI_ISL_1255135, EPI_ISL_1255136, EPI_ISL_1255137, EPI_ISL_1255139, EPI_ISL_1255140, EPI_ISL_1255144, EPI_ISL_1255145, EPI_ISL_1255147, EPI_ISL_1255150, EPI_ISL_1255151, EPI_ISL_1255154, EPI_ISL_1255155, EPI_ISL_1255156, EPI_ISL_1255160, EPI_ISL_1255161, EPI_ISL_1255167, EPI_ISL_1255170, EPI_ISL_1255174, EPI_ISL_1255176, EPI_ISL_1255183, EPI_ISL_1255184, EPI_ISL_1255185, EPI_ISL_1255207, EPI_ISL_1255210, EPI_ISL_1255215, EPI_ISL_1255223, EPI_ISL_1255225, EPI_ISL_1255228, EPI_ISL_1255232, EPI_ISL_1255235, EPI_ISL_1255238, EPI_ISL_1255242, EPI_ISL_1255245, EPI_ISL_1255246, EPI_ISL_1255247, EPI_ISL_1255248, EPI_ISL_1255252, EPI_ISL_1255253, EPI_ISL_1255264, EPI_ISL_1255265, EPI_ISL_1255266, EPI_ISL_1255272, EPI_ISL_1255273, EPI_ISL_1255276, EPI_ISL_1255277			
see above	West African Centre for Cell Biology of Infectious Pathogens (WACCBIP), University of Ghana, Accra, Ghana	West African Centre for Cell Biology of Infectious Pathogens (WACCBIP), University of Ghana, Volta Road, Legon-Accra, Ghana	; Abdoulaye B Diallo; Abdul-Karim Abass; Aisha Mohammed; Benjamin Demah Nuertey; Collins M. Morang'a; Dam Kenneth Mibut; Dominic S.Y. Amuzu; Emmanuella Amoako4; Evelyn B. Quansah; Frederick Kumi-Ansah; Frederick Tei-Maya; Gordon A Awandare; Joyce M. Ngoi; Kesego Tapela; Lucas N. Amenga-Etego; Nelson Kibinge; Oliver D Boakye5; Peter K Quashie; Philip M. Soglo; Samirah Saiid; Samuel Kaba Akoriyea; Theophilus Odoom; Vanessa Magnussen; Vincent Appiah; Yaw Bediako
EPI_ISL_1255105	West African Centre for Cell Biology of Infectious Pathogens (WACCBIP), University of Ghana, Volta Road, Legon-Accra, Ghana	West African Centre for Cell Biology of Infectious Pathogens (WACCBIP), University of Ghana, Volta Road, Legon-Accra, Ghana	; Abdoulaye B Diallo; Abdul-Karim Abass; Aisha Mohammed; Benjamin Demah Nuertey; Collins M. Morang'a; Dam Kenneth Mibut; Dominic S.Y. Amuzu; Emmanuella Amoako4; Evelyn B. Quansah; Frederick Kumi-Ansah; Frederick Tei-Maya; Gordon A Awandare; Joyce M. Ngoi; Kesego Tapela; Lucas N. Amenga-Etego; Nelson Kibinge; Oliver D Boakye5; Peter K Quashie; Philip M. Soglo; Samirah Saiid; Samuel Kaba Akoriyea; Theophilus Odoom; Vanessa Magnussen; Vincent Appiah; Yaw Bediako
EPI_ISL_1627236	Wojewodzka Stacja Sanitarno-Epidemiologiczna w Olsztynie, Laboratorium Badan Epidemiologiczno-Klinicznych	Wojewodzka Stacja Sanitarno-Epidemiologiczna w Olsztynie, Laboratorium Badan Epidemiologiczno-Klinicznych	Aleksandra Kobiatko; Barbara Dolinska; Emilia Tarabasz; Ewa Liszewska; Marta Lukian; Monika Czerminska; Patryk Bielecki; Paulina Rozycka; Sylwia Krzetowska; Tomasz Jakubczak
EPI_ISL_766787, EPI_ISL_1054879	Wyoming Public Health Laboratory	Wyoming Public Health Laboratory	Ashley Norberg; Bailey Bowcutt; Channing Weber; Lynette Gumbleton; Noah Hull; Taylor Fearing; and Wanda Manley
EPI_ISL_1595853	Yunan Center for Disease Control and Prevention	National Institute for Viral Disease Control and Prevention, China CDC	Jienan Zhou; Meiling Zhang; Senquan Jia; Xiang Zhao; Xiaoqing Fu; Yenan Feng; Yuchao Wu; Zhixiao Chen
EPI_ISL_681703, EPI_ISL_1014238	Zurita & Zurita Laboratorios	Zurita & Zurita Laboratorios	Camilo Zurita-Salinas; Gabriela Sevillano; Jeannete Zurita
EPI_ISL_826727, EPI_ISL_826740, EPI_ISL_827488, EPI_ISL_828809, EPI_ISL_829326	deCODE genetics	deCODE genetics	Agnar Helgason; Alma Moller; Arna B Agustsdottir; Arnaldur Gylfason; Asgeir Sigurdsson; Aslaug Jonasdottir; Berglind Eiriksdottir; Bjarni Thorbjornsson; Brynjar O Jensson; Daniel F Gudbjartsson; Dropplaug N Magnusdottir; Elisabet E Gardarsdottir; Emil A Thorarensen; Gardar Sveinbjornsson; Gisli Masson; Gudmundur Georgsson; Gudmundur L Norddahl; Gudrun Sigmundsdottir; Hakon Jonsson; Hannes Eggertsson; Hilma Holm; Ingileif Jonsdottir; Jona Saemundsdottir; Kamilla S Josefsdottir; Kari Stefansson; Karl G Kristinsson; Kjartan R Gudmundsson; Kristin E Sveinsdottir; Louise le Roux; Maney Sveinsdottir; Olafia S Gretarsdottir; Olafur T Magnusson; Pall Melsted; Patrick Sulem; Run Fridriksdottir; Solvi Rognvaldsson; Thora R Gunnarsdottir; Thordur Kristjansson; Thorolfur Gudnason; Unnur Thorsteinsdottir
EPI_ISL_1007972	genXone SA, Molecular Diagnostics Laboratory / NZOZ	genXone SA, Research & Development Laboratory	Aleksandra Gidlewicz; Anna Brylak-Błaszków; Grzegorz Nowicki; Jakub Grabowski; Karol Szeszko; Maciej Sykulski; Michał Kaszuba; Natalia Drwęska-Matelska; Łukasz Krych
EPI_ISL_872222, EPI_ISL_872243, EPI_ISL_872247, EPI_ISL_872328, EPI_ISL_872331, EPI_ISL_884006	hopital	National Reference Center for Viruses of Respiratory Infections, Institut Pasteur, Paris	Angela Brisebarre; Camille Capel; Combe Patrice; Etienne Simon-Lorière; Marion Barbet; Maud Vanpeene; Méline Bizard; Sylvie Behillil; Sylvie van der Werf; Vincent Enouf
EPI_ISL_458285, EPI_ISL_458286	unknown	Bundeswehr Institute of Microbiology	Antwerpen; Bestehorn-Willmann; Eckstein, S.; Handrick, S.; M.C.; M.H.; M.S.; Naija, H.; R. and Ben Moussa, M.; Rehn, A.; Stoecker, K.; Walter; Woelfel
EPI_ISL_463001, EPI_ISL_463003	unknown	Clinical virology	Fares, W.; Triki, H.
EPI_ISL_437615, EPI_ISL_437618	unknown	Faculty of Medicine	Buathong, R.; Bunprakob, S.; Ghai, S.; Joyjinda, Y.; Mungaomklang, A.; Petcharat, S.; Plipat; Prasithsirikul, W.; Rodpan, A.; Sirichan, N.; T. and Hemachudha, T.; Wacharapluesadee, S.
EPI_ISL_1527027	unknown	Inciensa, Instituto Costarricense de Investigación y Enseñanza en Nutrición y Salud	Barboza-Arguedas E & Cerdas-Quesada C; Pérez-Corrales C
EPI_ISL_476559	unknown	Laboratoire Sciences et Technologies de la Santé (STS) Institut	Abderraouf Hilali; Amal Souiri; Hajar Lemriss; Jawad Bouzid; Mustapha Mouallif; Narjis Amar; Sanaâ Lemriss; Saâd EL Kabbaj; Touria Essayagh

		Supérieur des Sciences de la Santé Université Hassan 1er, Settat, Morocco	
EPI_ISL_450409	unknown	Microbiology	K.-Y.; K.K.W.; To; Yuen
EPI_ISL_848601	Evandro Chagas Institute	Evandro Chagas Institute	A.M.; Barbagelata; E.C.; E.M.A.; Ferreira; J.A.; Junior; K.C.; L.C.; L.S.; M.C.; P.S.; Pinheiro; Santos; Silva; Sousa; Sousa Junior; W.D.C.; da Silva
